# Structural Effects on the Temperature Dependence of Hydride Kinetic Isotope Effects of the NADH/NAD^+^ Model Reactions in Acetonitrile: Charge-Transfer Complex Tightness Is a Key

**DOI:** 10.1021/acs.joc.3c02562

**Published:** 2024-02-16

**Authors:** Amanda Beach, Pratichhya Adhikari, Grishma Singh, Meimei Song, Nicholas DeGroot, Yun Lu

**Affiliations:** Department of Chemistry, Southern Illinois University Edwardsville, Edwardsville, Illinois 62026, United States

## Abstract

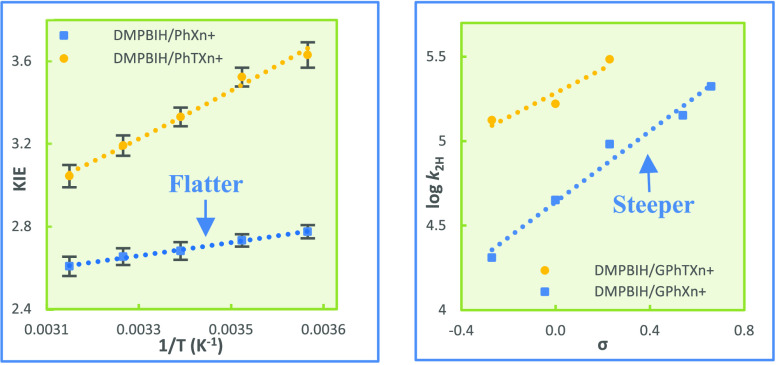

It has recently frequently been found that the kinetic isotope effect (KIE) is independent of temperature (*T*) in H-tunneling reactions in enzymes but becomes dependent on *T* in their mutants. Many enzymologists found that the trend is related to different donor–acceptor distances (DADs) at tunneling-ready states (TRSs), which could be sampled by protein dynamics. That is, a more rigid system of densely populated short DADs gives rise to a weaker *T* dependence of KIEs. Theoreticians have attempted to develop H-tunneling theories to explain the observations, but none have been universally accepted. It is reasonable to assume that the DAD sampling concept, if it exists, applies to the H-transfer reactions in solution, as well. In this work, we designed NADH/NAD^+^ model reactions to investigate their structural effects on the *T* dependence of hydride KIEs in acetonitrile. Hammett correlations together with N-CH_3_/CD_3_ secondary KIEs were used to provide the electronic structure of the TRSs and thus the rigidity of their charge-transfer complexation vibrations. In all three pairs of reactions, a weaker *T* dependence of KIEs always corresponds to a steeper Hammett slope on the substituted hydride acceptors. It was found that a tighter/rigid charge-transfer complexation system corresponds with a weaker *T* dependence of KIEs, consistent with the observations in enzymes.

## Introduction

The primary (1°) kinetic isotope effect (KIE) and its temperature (*T*) dependence, represented by the isotopic activation energy difference (i.e., Δ*E*_a_ = *E*_aD_ – *E*_aH_), have been used to study quantum H-tunneling mechanisms. Within the traditional Bell tunneling model that adds a one-dimensional tunnel correction to the classical transition state (TS) theory, a KIE greater than the classical limit of 7 and a Δ*E*_a_ outside of the classical range of 1.0–1.2 kcal/mol are indicators of a H-tunneling mechanism.^1^ Furthermore, the conner-cutting tunneling model within the variational TS theory indicates that a Δ*E*_a_ of >1.2 kcal/mol can be indicative of significant tunneling, irrespective of the size of the KIEs.^2^ To date, many researchers have used the Bell criteria to suggest a H-tunneling mechanism, but how H tunnels and why tunneling happens differently in different systems have not been thoroughly discussed.^3^

In the past >20 years, it has frequently been found that KIE is independent of *T* (Δ*E*_a_ ∼ 0) in wild-type enzymes but becomes dependent on *T* in their mutants (Δ*E*_a_ > 0).^4−23^ In the latter case, Δ*E*_a_ exceeds the classical range and very often becomes much larger than 1.2 kcal/mol. The seemingly regular enzyme structural effects on the Δ*E*_a_’s cannot be explained by the Bell model as in this model a Δ*E*_a_ of ∼0 can be observed only at extremely low temperatures when *E*_aH_ and *E*_aD_ are close to 0 and KIEs are huge.^24^ Many researchers have used the newly developed vibration-assisted activated H-tunneling (VA-AHT) model to explain the observed trend in Δ*E*_a_’s.^4,6,8,21−23,25−27^ In that ground state full tunneling phenomenological model, there are two orthogonal activation processes.^4,6,7,28,29^ In one process, heavy atom motions bring the donor reactant (D-H) and acceptor product (H-A) to an activated degenerate state where H-wave functions from both could overlap ([D-H ↔ H-A]^⧧^), i.e., H tunneling. Therefore, the degenerate energy state is a tunneling-ready state (TRS). In the second activation process, heavy atom motions sample the short donor–acceptor distances (DADs) for efficient H tunneling to occur. The first (1) activation process is almost insensitive to the isotope [*E*_aH_(1) = *E*_aD_(1)], whereas the second (2) process is sensitive to the isotope. The latter is due to the fact that the heavier isotope vibration possesses a shorter wavelength; thus, a higher energy is required to sample shorter DADs so that the D isotope can effectively tunnel [*E*_aD_(2) > *E*_aH_(2)]. When the system is sufficiently rigid that DADs are very narrowly distributed, their sampling is not possible, and *E*_aD_(2) and *E*_aH_(2) are close, making Δ*E*_a_ ∼ 0 [=*E*_aD_(2) – *E*_aH_(2)]. Therefore, Δ*E*_a_ reflects the *T* dependence of DADs. Within this model, the *T* independence of KIEs in wild-type enzymes is explained in terms of strong protein vibrations that press the donor and acceptor close to each other so that a thermal DAD sampling is almost not needed, whereas in enzyme mutants, the natural vibrations are impaired and DADs become longer so that the thermal energy required to sample shorter DADs for D tunneling becomes larger, leading to a larger Δ*E*_a_. The discussion has been used to support the recently proposed role of protein dynamics in enzyme catalysis.^5,30−33^

The DAD sampling concept in the VA-AHT model was initially proposed to explain the change in the *T* dependence of KIEs for general H-tunneling reactions from enzymes to mutants.^4,6,11,34^ It was subsequently argued that the model may not be used for the adiabatic proton or hydride-transfer reactions in which tunneling may take place from the ground state of the reactant to the excited state of the product at the TRS.^35−37^ The fact, however, is that the hydride-transfer reactions (as well as proton-transfer reactions^13,38^) in enzymes versus mutants frequently demonstrate the same trend of the change in Δ*E*_a_’s as predicted from the VA-AHT model. Several groups did find that Δ*E*_a_’s for the hydride-transfer reactions are correlated to the DAD distributions at the TRS in the same way as described in the model.^18,20−22,25^ In the meantime, γ-secondary (2°) KIEs for hydride-transfer reactions in enzymes and solution were found to be fitted to this ground state tunneling model, as well.^39−43^ While the commonly accepted tunneling model for hydride-transfer reactions has not been established, these studies indicate that an assumed model somehow contains a DAD−Δ*E*_a_ relationship. On the other hand, simulations using the ensemble averaged variational TS theory, including multidimensional H tunneling and the empirical valence bond approach, show that the small Δ*E*_a_ observed in the hydride-tunneling process catalyzed by a dihydrofolate dehydrogenase results from the insensitivity of DADs to temperature.^44,45^ Nonetheless, study of the said relationship in hydride-transfer reactions will help in the development of hydride-tunneling models and provide insight into the proposed DAD sampling activation in enzyme-catalyzed hydride-transfer reactions.

To examine the DAD−Δ*E*_a_ relationship in hydride-transfer reactions, we have started a project to study the structural and solvent effects on the Δ*E*_a_’s for the reactions of NADH/NAD^+^ models in solution.^42,43,46−49^ Our hypothesis following the enzymatic observations and explanations described above is that a more rigid H-tunneling system of narrowly distributed DADs gives rise to a smaller Δ*E*_a_ value, and this should be applicable to all kinds of H-transfer reactions. One reason that we chose hydride-transfer reactions of NADH/NAD^+^ models for the study is that all of the hydride-transfer enzymes for DAD−Δ*E*_a_ relationship studies in the literature use NADH/NAD^+^ coenzymes, so our study can be directly compared to the enzymes to provide insight into the explanations about the role of protein dynamics in enzyme catalysis.^46,49^ The other reason is that all of the reactions take place in charge-transfer (CT) complexes so experimental design to modulate the DADs to study their relationship with Δ*E*_a_’s could use the electronic effect considerations. For one example, we have reported the *T* dependence of KIEs for hydride-transfer reactions from 1,3-dimethyl-2-phenylbenzimidazoline (DMPBIH) and 10-methylacridine (MAH) to 9-[*p*-substituted(*G*)]phenylxanthylium ion [*G*PhXn^+^, counterion BF_4_^–^ (same below)] in acetonitrile.^48^ We found that a stronger hydride donor and/or acceptor, which favors tighter CT complexation, gives rise to a smaller Δ*E*_a_. This supports our hypothesis.

**Figure 1:**
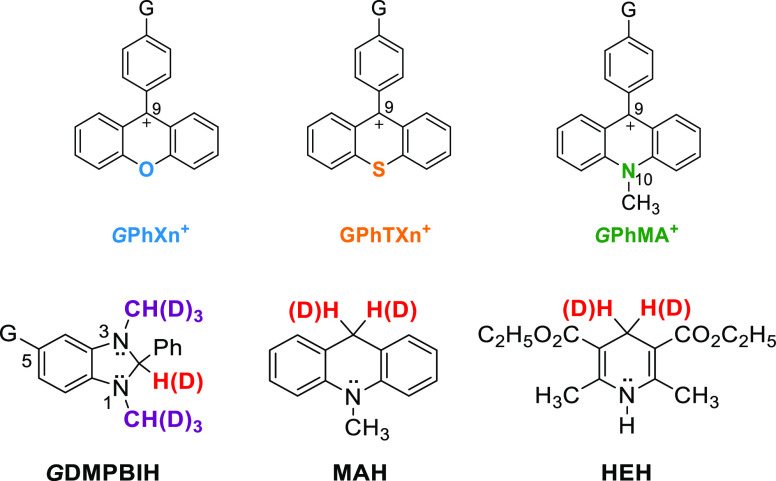
Hydride acceptors (top) and donors (bottom) used.

**Figure 2:**
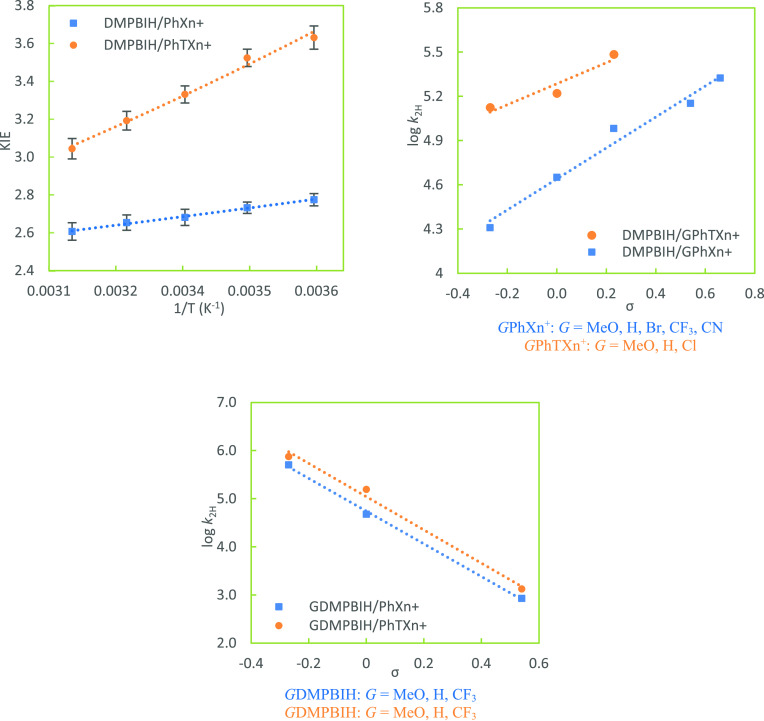
Arrhenius plot of KIEs for hydride-transfer reactions from DMPBIH to Ph(T)Xn^+^ (reaction pair I) (top left, temperatures of 5, 15, 25, 35, and 45 °C; lines are nonlinear exponential fits) as well as Hammett correlations of *G*Ph(T)Xn^+^ (top right) and *G*DMPBIH (bottom) at 25 °C.

**Figure 3:**
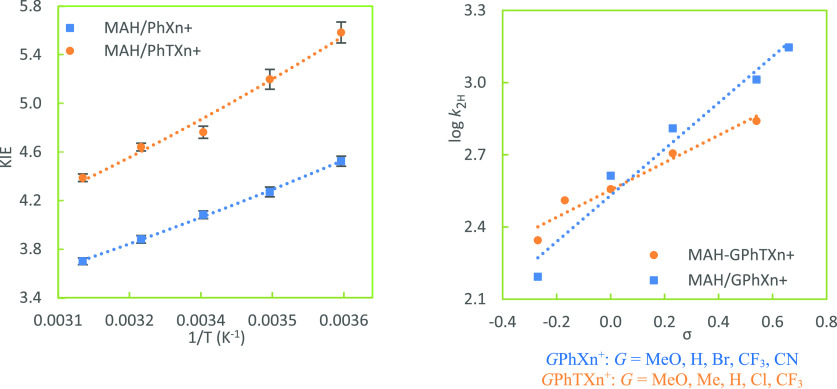
Arrhenius plot of KIEs for hydride-transfer reactions from MAH to Ph(T)Xn^+^ (reaction pair II) (left, temperatures of 5, 15, 25, 35, and 45 °C; lines are nonlinear exponential fits) as well as Hammett correlations of *G*Ph(T)Xn^+^ (right) at 25 °C.

**Figure 4:**
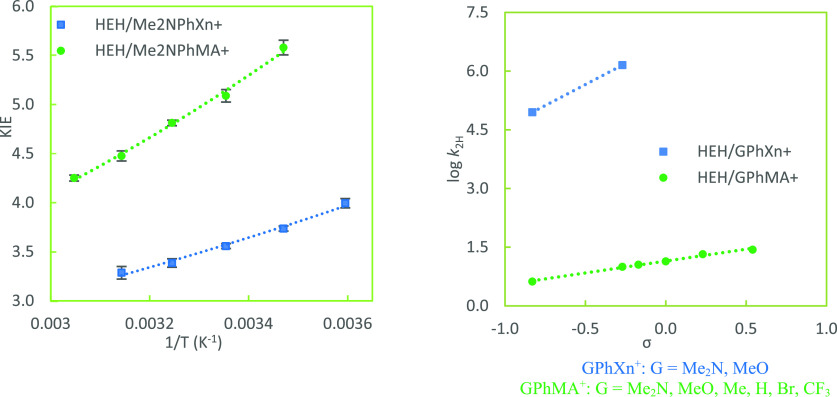
Arrhenius plot of KIEs for hydride-transfer reactions from HEH to Me_2_NPhXn^+^ and Me_2_NPhMA^+^ (reaction pair III) [left, temperatures of 5, 15, 25, 35, and 45 °C (or from 15 to 55 °C); lines are nonlinear exponential fits] as well as Hammett correlations of *G*PhXn^+^ and *G*PhMA^+^ (right) at 25 °C.

To further study the rigidity−Δ*E*_a_ relationship between the tightness of the CT complexes and Δ*E*_a_, in this paper, we change the O in PhXn^+^ to S [for the 9-phenylthioxanthylium ion (PhTXn^+^)] and N [for the 9-phenylacridinium ion (PhMA^+^)] and vary the *para* -group (*G*) in the *G*PhMA^+^, to attempt to regulate the CT complex rigidities and link the rigidities to the Δ*E*_a_ values for their hydride-transfer reactions with various hydride donors in acetonitrile (Figure 1; *G*’s are indicated in Figures 2–4). The major reasons behind our design are as follows. (i) By comparison with O, S decreases the positive charge delocalization to the central ring due to the longer C–S bond and mismatching p orbitals, weakening the ability of PhTXn^+^ to form a π–π interaction with the donor and thus the CT complex rigidity. (ii) N has a lower electronegativity, and the positive charge is more stable, which also decreases the degree of CT complexation. (iii) The electron-withdrawing group at *G*PhMA^+^ would facilitate a stronger CT complexation. To demonstrate the electronic structures of the CT complexation in the TRSs, we constructed the Hammett correlations for the hydride transfers from 5-substituted *G*DMPBIH, MAH, and Hantzsch ester (HEH) to *G*PhXn^+^, *G*PhTXn^+^, and *G*PhMA^+^ (Figure 1). For the same purpose, we also determined the 1,3-N,N-2CH_3_/2CD_3_ γ2° KIEs on DMPBIH to evaluate the change in electron density on N during the reaction. We found that the *G*PhXn^+^ systems indeed form stronger CT complexes than the *G*PhTXn^+^ and *G*PhMA^+^ systems. Together with the Δ*E*_a_ values we determined, our results show that a more rigid system gives rise to a smaller Δ*E*_a_ value, supporting our hypothesis.

## Results

It is well-known that hydride transfer in the NADH/NAD^+^ model reactions takes place within a CT complex of reactants.^47,50−52^ In this paper, we call these complexes productive reactant complexes (PRCs). We have reported the spectroscopic evidence of CT complex formation for similar reactions.^47,53^ In this work, we also demonstrated the same evidence of the formation of CT complexes for the selected reaction between MAH and PhXn^+^ (Figure S1). The PRCs are believed to form at a diffusion-controlled rate. Theoretically, the hydride transfer could be classical through a TS or nonclassical through a TRS. This mechanism is described in eq 1 (Don-H and Acc refer to the donor and acceptor, respectively).^49^ The observed KIEs are derived from second-order rate constants (*k*_2_). They correspond to the hydride-transfer step (*k*_H_). That is, KIE = *k*_2H_/*k*_2D_ = *k*_H_/*k*_D_.
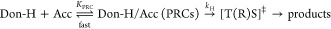
1

Below, we will present the *T* dependence of KIEs and the use of Hammett correlations and 2° N-CH_3_/CD_3_ KIEs to determine the electronic structures of the T(R)S complexes.

### *T* Dependence of KIEs

The *T* dependence of KIEs of the hydride-transfer reactions was determined in acetonitrile over a 40 °C temperature range of 5–45 or 15–55 °C. Three pairs of hydride-transfer reactions include that from DMPBIH to PhXn^+^ versus PhTXn^+^ (reaction pair I), that from MAH to PhXn^+^ versus PhTXn^+^ (reaction pair II), and that from HEH to (CH_3_)_2_NPhXn^+^ versus (CH_3_)_2_NPhMA^+^ (reaction pair III). In the reactions of HEH, the least reactive (CH_3_)_2_N acceptors were used. This is due to the rate measurement limitation of the stopped-flow instrument for the very fast reaction of *G*PhXn^+^. Additionally, the *T* dependence of KIEs was determined for the reactions of HEH with various *G*PhMA^+^ ions. This study focuses on how the substituent effect affects the *T* dependence of KIEs in this series of reactions. Representative second-order rate constants (*k*_2H_) and KIEs at 25 °C and Δ*E*_a_ values are listed in Table 1. The left panels of Figures 2–4 show the Arrhenius plots of KIEs for each pair of reactions for a direct comparison of the two systems. As expected, the Δ*E*_a_ is smaller for the reaction of PhXn^+^ than those for the reactions of PhTXn^+^ (Figures 2 and 3) and PhMA^+^ (Figure 4), whereas Δ*E*_a_ appears not to change significantly with substitutions in the reactions of *G*PhMA^+^ (group IV reactions in Table 1).

**Table 1:** Structural Effects on the Temperature Dependence of 1° KIEs (in acetonitrile)

classification	donor	acceptor	*k*_2H_^25 ^°^C^ (M^–1^ s^–1^)	1° KIE^25 ^°^C^^a^	Δ*E*_a(D-H)_ (kcal/mol)^a^
pair I	DMPBIH	PhXn^+^	(4.54 ± 0.05) × 10^4^^b^	2.68 (0.04)^b^	0.27 (0.06)^b^
	DMPBIH	PhTXn^+^	(1.66 ± 0.02) × 10^5^	3.33 (0.05)	0.79 (0.12)
pair II	MAH	PhXn^+^	(4.10 ± 0.03) × 10^2^^b^	4.08 (0.03)^b^	0.88 (0.05)^b^
	MAH	PhTXn^+^	(3.69 ± 0.03) × 10^2^	4.79 (0.05)	1.08 (0.14)
pair III	HEH	(CH_3_)_2_NPhXn^+^	(8.87 ± 0.05) × 10^4^	3.56 (0.02)	0.86 (0.08)
	HEH	(CH_3_)_2_NPhMA^+^	4.19 ± 0.03	5.09 (0.06)	1.27 (0.14)
group IV^c^ (a series of reactions)					
	HEH	CH_3_OPhMA^+^	(1.00 ± 0.01) × 10	5.11 (0.08)	1.31 (0.10)
	HEH	CH_3_PhMA^+^	(1.13 ± 0.02) × 10	5.30 (0.10)	1.33 (0.10)
	HEH	PhMA^+^	(1.37 ± 0.08) × 10	5.31 (0.04)	1.19 (0.07)
	HEH	BrPhMA^+^	(2.09 ± 0.03) × 10	5.20 (0.08)	1.32 (0.09)
	HEH	CF_3_PhMA^+^	(2.74 ± 0.03) × 10	5.23 (0.05)	1.13 (0.19)

a Numbers in parentheses are standard deviations.

b From ref (48).

c (CH_3_)_2_NPhMA^+^ in pair III acceptors belongs to this group, as well.

### Hammett Correlations

Hammett correlations with substituent constants (σ) of *G*PhXn^+^, *G*PhTXn^+^, and *G*PhMA^+^ for their reactions with various hydride donors were constructed using the corresponding *k*_2H_’s at 25 °C. Hammett plots for each pair of the reactions are put side by side with the corresponding Arrhenius plots of KIEs for a direct comparison (right panels in Figures 2–4). Due to the rate measurement limitation for the reactions of DMPBIH with *G*PhTXn^+^ and the reactions of HEH with *G*PhXn^+^, fewer rate data are available for their Hammett plots. The Hammett constants (ρ) for the three pairs of reactions (I–III) were obtained and are listed in Table 2 and Figure S2. The observed positive ρ values of the three series of substituted hydride acceptors indicate that the acceptor loses positive charge during the reactions. In the three reaction pairs, the reactions of *G*PhXn^+^ with larger ρ values are more sensitive to the substituent effects than the reactions with *G*PhTXn^+^ and *G*PhMA^+^. To estimate the charge carried by the acceptor moiety at the activated reactive complexes, a comparison of the Hammett correlations on the equilibrium constants (*K*_H_-) for the corresponding carbocations to accept a full hydride ion is needed. These latter correlations were constructed, as well (Figure S4). Because the ρ value reflects the partial loss of positive charge from the carbocations during the activation process and the ρ(*K*_H_-) value reflects a full loss of positive charge from the same for the overall reaction, the estimated partial positive charge carried by the acceptor moieties at the T(R)S can be calculated as ξ = 1 – ρ/ρ(*K*_H_-).^54^ It should be noted that the corresponding Hammett correlations of the σ^+^ values were also constructed (Figures S3 and S5). In general, the correlations with σ were found to be slightly better. The corresponding ρ^+^ and ρ^+^(*K*_H_-) and hence the charge (ξ) derived are also listed in Table 2 for comparison with the results from the correlations with the σ values. The ξ values obtained from the correlations with σ^+^ are close to those obtained with σ values.

**Table 2:** Hammett Constants (ρ) of *k*_2H_ and *K*_H_- and Estimated Charge (ξ) of the Reactive Complexes for the Three Pairs of Reactions^a^

classification	reaction system	ρ (ρ^+^)^b^	ρ(*K*_H_-) [ρ^+^(*K*_H_-)]^b^,^c^	estimated charge (ξ)^d^
pair I	DMPBIH/*G*PhXn^+^	1.05 (0.68)	4.59 (2.84)	+0.77 (+0.76) on PhXn
	DMPBIH/*G*PhTXn^+^	0.71 (0.30)	6.12 (3.73)	+0.88 (+0.92) on PhTXn
pair I	*G*DMPBIH/PhXn^+^	–3.40 (1.93)^e^	N/A	φ = +0.42 on DMPBIH^f^
	*G*DMPBIH/PhTXn^+^	–3.45 (1.98)^e^	N/A	φ = +0.42 on DMPBIH^f^
pair II	MAH/*G*PhXn^+^	0.96 (0.64)	4.59 (2.84)	+0.79 (+0.77) on PhXn
	MAH/*G*PhTXn^+^	0.57 (0.36)	6.12 (3.73)	+0.91 (+0.90) on PhTXn
pair III	HEH/*G*PhXn^+^	2.15 (1.31)	4.59 (2.84)	+0.53 (+0.54) on PhXn
	HEH/*G*PhMA^+^	0.61 (0.34)	5.93 (3.73)	+0.90 (+0.91) on PhMA

a In acetonitrile.

b From the correlations with σ or σ^+^. See the fitting parameters in Figures S2 and S3.

c *K*_H_- is for the R^+^ + H^–^ → R-H equilibrium, converted from the hydride affinities of R^+^ from the literature.^55^

d Calculated from 1 – ρ/ρ(*K*_H_-) or 1 – ρ^+^/ρ^+^(*K*_H_-) for the numbers in parentheses, unless otherwise noted.

e Less reliable (see the text).

f See Table 3; calculated from (1 – 2° KIE)/(1 – 2° EIE) (cf. refs (49) and (56)).

Hammett correlations of *G*DMPBIH were also constructed to determine the electronic structure of the donor moiety of the activated reactive complexes for the reactions of DMPBIH with PhXn^+^ versus PhTXn^+^. The two Hammett plots are shown in Figure 2 (bottom) to allow a direct comparison with the same for the *G*Ph(T)Xn^+^ acceptors. The observed negative ρ values indicate that the donor gains a positive charge during the reactions (Table 2). It is interesting that the Hammett constants [ρ (ρ^+^)] are the same within experimental error. Because the rates for the MeODMPBIH reactions are too fast beyond our ability to accurately measure them (especially under our concentration conditions), the use of the corresponding ρ (ρ^+^) to estimate the amount of partial positive charge (ξ) carried by the donor DMPBIH moiety in the reactive complexes may not be proper. Also, because we do not have the ρ(*K*_H_-) value for the corresponding oxidized structures (*G*DMPBI^+^), we are not able to derive the amount of partial positive charge (ξ) carried by the donor DMPBIH moiety in the reactive complexes, either. For these reasons, we compare the 1,3-N,N-2CH_3_/2CD_3_ 2° KIEs on DMPBIH with the corresponding 2° equilibrium isotope effect (EIE) for DMPBIH to accept a full hydride ion to derive the charges carried by the DMPBIH moiety of the two reactions.

### 1,3-N,N-2CH_3_/2CD_3_ γ-2° KIEs on DMPBIH for Its Reactions with Ph(T)Xn^+^

The N-CH/CD 2° KIE originates from the isotopic difference in negative hyperconjugation between the lone pair of electrons on N and the empty σ* orbital of the C–H/D bond.^47,57^ It resulted from the loss or gain of electron density on N during the reaction. The electron density loss tightens the C–H/D bonds, leading to an inverse 2° KIE, whereas the electron density gain loosens the C–H/D bonds, leading to a normal 2° KIE.^57,58^ According to this analysis, for the reactions of DMPBIH with PhXn^+^ and PhTXn^+^, the 1,3-N,N-2CH_3_/2CD_3_ γ-2° KIEs on DMPBIH should be the inverse. Table 3 lists the 1,3-N,N-2CH_3_/CD_3_ γ-2° KIEs on DMPBIH that we determined for the two reactions. The two 2° KIE values are indeed inverse and interestingly the same, implying that the DMPBIH moieties in the two T(R)S complexes carry the same amount of positive charge. This appears to be consistent with the same Hammett constant values of *G*DMPBIH for their reactions with PhXn^+^ and PhTXn^+^ (see Figure 2 and Table 2). While the rate data for the reaction of MeODMPBIH are less accurate for the Hammett correlations, it is interesting that the electronic structure information about the TRS derived from both methods is consistent.

**Table 3:** CH_3_/2CD_3_ γ-2° KIEs and 2° EIE on DMPBIH for It to Release a Hydride Ion^a^

γ-2CH_3_/2CD_3_ 2° KIE^b^		
with PhXn^+^	with PhTXn^+^	γ-2CH_3_/2CD_3_ 2° EIE^c^
0.89 (0.01)	0.89 (0.01)	0.74

a In acetonitrile, numbers in parentheses are standard deviations.

b The 2° KIE of 0.91 for PhXn^+^ was reported previously;^48^ here we used the new batch of isotopologues and same isotopologue solutions for the kinetics of both reactions for a back-to-back comparison.

c From ref (49).

A comparison of the γ-2° KIEs with the corresponding 2° EIE we reported^49^ (Table 3) gives the partial positive charge on DMPBIH as φ = +0.42 [=1 – (2° KIE)/(2° EIE)] for both reactions.^49,56^ This is also listed in Table 2 for comparison with the amount of positive charge borne by the PhXn and PhTXn moieties in the two activated complexes [T(R)Ss]. For the pair I reactions, the PhXn moiety receives −0.23 charge whereas the PhTXn moiety receives −0.12 charge at the T(R)S. Note that to balance the +1 charge of both systems, in the two T(R)Ss, the in-flight nucleus (H) carries charges of −0.19 and −0.30, respectively.

## Discussion

For the reactions in this paper, KIEs are small (<7), but Δ*E*_a_’s range from 0.27 to 1.33 kcal/mol, some of which are within and some of which are outside of the semiclassical range of 1.0–1.2 kcal/mol. While such hydride-transfer reactions of NADH/NAD^+^ analogues usually have small KIEs, both this and other works of ours as well as a few sporadic works from others showed that they have Δ*E*_a_’s spanning a wide range from well below the semiclassical limit (∼0 kcal/mol), through the semiclassical range, to well above the semiclassical limit (≲1.8 kcal/mol).^46−48,53,59^ Furthermore, it has been shown that small KIEs from such hydride-transfer reactions also fit to the Marcus theory of atom transfer that involves a H-tunneling component.^60−62^ In the meantime, the small KIEs and similar Δ*E*_a_’s were also found in the hydride-transfer reactions of NADH/NAD^+^ in enzymes and mutants.^6,17,18,20,25,63,64^ As described in the Introduction, the latter observations have been explained following various contemporary H-tunneling models.

The ultimate goal of this study is to correlate Δ*E*_a_ with DADs at TRSs in a H-tunneling mechanism. Our hypothesis is that a more rigid system of narrowly distributed DADs gives rise to a smaller Δ*E*_a_. In the literature study of the DAD−Δ*E*_a_ relationship for enzyme-catalyzed H-tunneling reactions, the DAD distribution density or enzyme active site rigidity was evaluated from molecular dynamics calculations,^18,20−22^ secondary (2°) KIEs on the α-C-H(D) groups,^25^ the information from the two-dimensional IR measurements^20^ of the enzyme–coenzyme–inhibitor ternary complex structures, and the stable structures that mimic the productive reactant complexes of the enzymatic reactions.^20,65^ In our study of the relationship for the hydride-transfer reactions of NADH/NAD^+^ model reactions in solution, we have used the computed distributions of DADs in the productive reactant complexes (PRCs; cf. eq 1) as well as the 2° KIEs on N-CH_3_/CD_3_ of the reactants to evaluate the tightness of the TRS complexes.^42,43,47−49^ Here in this work, we use the Hammett correlations (along with the N-CH_3_/CD_3_ 2° KIEs) to evaluate the electronic structure and tightness of TRS complexes. The most important discovery is that in all three pairs of reactions (I–III) with substituted acceptors in the same steric environments, a system with a steeper Hammett slope always corresponds with a smaller slope of the Arrhenius plot of KIEs for the reaction of a certain acceptor, i.e., a smaller Δ*E*_a_ value (see side-by-side comparisons of the two plots in Figures 2–4)!

### Correlation of DADs with Δ*E*_a_ for the Reactions of Me_2_NPhXn^+^ versus Me_2_NPhMA^+^ (pair III reactions)

It is expected that a stronger hydride donor/acceptor would form a stronger CT complexation and thus a more rigid system. It has been reported that the hydride affinities [−Δ*G*_H_-(R^+^)] of PhXn^+^ and PhMA^+^ in acetonitrile are 91.6 and 71.4 kcal/mol, respectively.^55^ Therefore, PhMA^+^ is a weaker hydride acceptor and is expected to form a weaker CT complex with a donor. The Hammett correlation results do show that the PhMA moiety receives less negative charge than PhXn in the TRSs of their reactions with HEH [−0.10 vs −0.47 (Table 2)]. This is consistent with our expectation that the *G*PhMA^+^ system is looser than the *G*PhXn^+^ system in their CT complexation with a donor due to the N/O electronegativity difference. Meanwhile, we are aware that the change in charge during the reaction is not merely from CT complexation; rather, the extent of hydride transfer (bond cleavage and formation) is also responsible for the charge distributions at the TRS. In this regard, according to Hammond’s postulate, the TRS is later on the reaction coordinate for the exergonic reaction of *G*PhMA^+^ than that of *G*PhXn^+^ (the hydride affinity of the oxidized form of HEH in acetonitrile is 64.4 kcal/mol, so the reactions are exergonic^55^). Therefore, the hydride transfer would result in a larger loss of positive charge from *G*PhMA^+^ than from *G*PhXn^+^, but the observed Hammett plot has a much smaller slope in the former system. This suggests that the observed greater loss of positive charge from *G*PhXn^+^ results mainly from a tighter CT complexation at the TRS.

A comparison of Δ*E*_a_’s for the reactions of HEH with (CH_3_)_2_NPhMA^+^ (1.27 kcal/mol) versus (CH_3_)_2_NPhXn^+^ (0.86 kcal/mol) from Table 1 indicates that the less rigid system of PhMA^+^ corresponds with a larger Δ*E*_a_, which is consistent with our hypothesis.

### Correlation of DADs with Δ*E*_a_ for the Reactions of PhXn^+^ and PhTXn^+^ (pair I and pair II reactions, respectively)

Compared to PhXn^+^, because of the smaller amount of positive charge delocalization to S of a larger size, PhTXn^+^ is a slightly stronger hydride acceptor (the hydride affinities of PhXn^+^ and PhTXn^+^ in acetonitrile are 91.6 and 96.0 kcal/mol, respectively^55^). This would, however, decrease the degree of π–π CT complexation with a donor and thus the rigidity of the TRS. The Hammett correlations of the two systems show that during the reaction, the PhTXn moiety receives less negative charge density from both DMPBIH and MAH (Table 2). Specifically, in the TRSs of the reactions of DMPBIH (reaction pair I), PhTXn receives a charge of −0.12 but PhXn receives a charge of −0.23, and in the reactions of MAH (reaction pair II), PhTXn receives a charge of −0.09 whereas PhXn receives a charge of −0.21. This appears to show that the CT complexation in the TRS of the reaction of PhTXn^+^ is looser than that of the PhXn^+^, but the charge distribution resulted from the C–H → C bond changes should also be considered in the rigidity order analysis. According to Hammond’s postulate, the PhTXn^+^ reaction should be earlier in the reaction process and thus have a smaller Hammett slope or less loss of positive charge upon reaching the TRS. We found that in the two exergonic reactions, the observed loss of positive charge from the reaction of PhTXn^+^ is ∼2 times smaller than that from the reaction of PhXn^+^ (the hydride affinities of the oxidized forms of DMPBIH and MAH are 49.2 and 76.2 kcal/mol, respectively, so the reactions are exergonic^55^). While the reaction of PhTXn^+^ is only 4.2 kcal/mol more exergonic than the reaction of PhXn^+^, the observed relatively large charge loss difference in the two reactions is not likely to have resulted from only the C–H bond breaking and forming at the TRS. This is likely true because the free energy changes of the overall reactions are so large that the TRSs are very early on the reaction coordinate, making the bond changes less sensitive to the structural change. For example, according to the hydride affinities of the reactant and product, the Δ*G*°’s for the reactions of DMPBIH with PhXn^+^ and PhTXn^+^ are as high as −42.4 and −46.8 kcal/mol, respectively.

In both pairs of reactions (I and II), the observed Δ*E*_a_ is larger in the PhTXn^+^ systems than in the PhXn^+^ systems: 0.79 and 0.27 kcal/mol, respectively for the reactions with DMPBIH, and 1.08 and 0.88 kcal/mol, respectively for the reactions with MAH (Table 1). This shows that a looser system gives rise to a larger Δ*E*_a_ value, which is also consistent with our hypothesis.

### Further Evidence for the Correlation of DADs with Δ*E*_a_

From the hydride affinities of the oxidized forms of the donor compounds, DMPBIH is 25 kcal/mol more reactive than MAH,^55^ indicating that DMPBIH is a much stronger hydride/electron donor and thus would form a stronger CT complexation with an acceptor. In their reactions with both PhXn^+^ and PhTXn^+^, DMPBIH always gives smaller Δ*E*_a_ value [pair I vs pair II (Table 1)]. For example, with PhXn^+^, Δ*E*_a_ is 0.61 kcal/mol (=0.88 kcal/mol – 0.27 kcal/mol) smaller for the reaction of DMPBIH than that of MAH, and in the reactions of PhTXn^+^, it is 0.29 kcal/mol (=1.08 kcal/mol – 0.79 kcal/mol) smaller. This appears to support our hypothesis of relating Δ*E*_a_ to the system rigidity, as well.

The analysis presented above also suggests that Δ*E*_a_ is less sensitive to the change in the donor structure from DMPBIH to MAH for the loose systems with PhTXn^+^ (ΔΔ*E*_a_ = 0.29 kcal/mol) than the tight systems with PhXn^+^ (ΔΔ*E*_a_ = 0.61 kcal/mol). In fact, this is the same for the change in the acceptor structure from PhXn^+^ to PhTXn^+^. Their reactions for the loose systems that use the MAH donor give a ΔΔ*E*_a_ of 0.20 kcal/mol (=1.08 kcal/mol – 0.88 kcal/mol), whereas for the tight system with DMPBIH, the ΔΔ*E*_a_ is larger [0.52 kcal/mol (=0.79 kcal/mol – 0.27 kcal/mol)]. Therefore, Δ*E*_a_ appears to be less sensitive to structural changes in the looser systems.

The fact that the Δ*E*_a_ is less sensitive to structural change in the looser systems was seen in the systems with a series of less reactive *G*PhMA^+^ acceptors, as well (Table 1). It is expected that the electron-withdrawing group (EWG) increases the positive charge density on the MA ring, thereby increasing the strength of the CT complexation with the donor and thus the system rigidity, but the Δ*E*_a_ for their reactions with HEH appears not to show an apparent increasing trend with a changing *G* in a wide range from CF_3_ (EWG) to N(CH_3_)_2_ (EDG) groups [Δ*E*_a_ ∼ 1.3 kcal/mol (Table 1)]. This is consistent with our reported observations that Δ*E*_a_ is less sensitive to substituents in the reactions of *G*PhXn^+^ with a weaker electron donor (MAH) than those with a stronger donor (DMPBIH). In that work, Δ*E*_a_ changes from 0.89 kcal/mol (*G* = CF_3_) to 0.96 kcal/mol [*G* = N(CH_3_)_2_] for the reactions of MAH (ΔΔ*E*_a_ = 0.07 kcal/mol only), but a relatively large change (ΔΔ*E*_a_ = 0.46 kcal/mol) was found for the reactions of the stronger electron/hydride donor (DMPBIH) {Δ*E*_a_ from 0.04 kcal/mol (*G* = CF_3_) to 0.50 kcal/mol [N(CH_3_)_2_]}.^48^

## Conclusions

Structural effects on the temperature dependence of KIEs (represented by Δ*E*_a_) were studied by using hydride-transfer reactions of NADH/NAD^+^ analogues in acetonitrile. The hydride donors and acceptors were designed according to their π-electron/charge donating/accepting abilities to form a CT complex. It appears that a system with a stronger CT complexation gives rise to a smaller Δ*E*_a_ value. In enzymes, a smaller Δ*E*_a_ has been frequently observed in wild-type enzymes, and Δ*E*_a_ increases when enzymes are mutated. Many researchers have found that DADs are more densely populated in the wild-type enzymes and their size and population range increase as enzymes are mutated. Therefore, the structural effects on Δ*E*_a_ are the same in solution as in enzymes with respect to their links to system rigidities. That is, a H-tunneling system with more rigid reaction centers gives rise to a lower Δ*E*_a_ value. Theoreticians have been attempting to develop a tunneling model for enzyme-catalyzed hydride and H atom-transfer reactions to explain the DAD−Δ*E*_a_ relationship, but a universally accepted model is still lacking. Our results from the study in solutions will certainly be valuable additions to the current debates about the appropriateness of models to describe general H-transfer reactions and provide insight into the contentious role of protein dynamics in enzyme catalysis.

## Experimental Section

Syntheses of hydride donors^47,48,56^ DMPBIH, MAH, and HEH and their deuterated analogues, as well as hydride acceptors *G*PhXn^+^BF_4_^–^^48,66^ and *G*PhTXn^+^BF_4_^–^,^67^ can be found from our previous work. The 5-substituted *G*DMPBIHs were prepared according to the procedure described in the literature.^68^ The 9-aryl-substituted acridinium ions (*G*PhMA^+^BF_4_^–^) were prepared via the transfer of a hydride from the corresponding reduced forms (*G*PhMAH) to the tropylium ion (Tr^+^BF_4_^–^) in acetonitrile. The *G*PhMAHs were synthesized by the reaction of acridinium salt (MA^+^I^–^) with the corresponding Grignard reagents of *G*PhMgBr in dry tetrahydrofuran. The latter synthesis procedures are from Fukuzumi’s work.^69^ All of the compounds are known. They were purified carefully and characterized by NMR and melting points. The HPLC grade acetonitrile solvent was redistilled twice, using KMnO_4_/K_2_CO_3_ to remove the reducing impurities and P_2_O_5_ to remove water, in order under nitrogen. Kinetic solutions were prepared using the freshly distilled solvents and kept in the refrigerator (4 °C) or freezer (−20 °C) before use under each kinetic temperature condition.

Kinetics were determined on an SF-61DX2 Hi-Tech KinetAsyst double-mixing stopped-flow instrument. The same kinetic procedures used in our publications were followed.^47−49^ From our experiments and the literature, the types of reactions strictly follow the second-order rate law.^46−48,55,56,60,70^ Each KIE was derived from the second-order rate constants of the isotopic reactions (=*k*_2H_/*k*_2D_). Experimentally, the pseudo-first-order rate constants (*k*^pfo^’s) were determined spectroscopically (by ultraviolet–visible) and the observed *k*_2_ was calculated by dividing *k*^pfo^ by the concentration of the large excess reactant (for example, R–H or R–D), i.e., *k*_2_ = *k*^pfo^/[R–H(D)]. Then
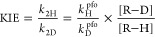
2Usually, the same concentrations of R–H and R–D solutions were used, but to eliminate the errors in the preparation of the two solutions at a certain concentration, we corrected the concentration ratio by measuring the absorbance (Abs) of each isotopic solution at an appropriate wavelength (this is especially necessary for the measurements of small 2° KIEs). Assuming R–H and R–D have the same extinction coefficient at the wavelength (ε_H_ = ε_D_), we have
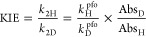
3For the measurements of N-CH_3_/CD_3_ 2° KIEs on DMPBIH for its reactions with PhXn^+^ and PhTXn^+^, the same isotopic solutions were used to reduce the weighing errors and hence to allow a direct comparison of the 2° KIEs in the two systems.

Six measurements of *k*^pfo^’s for the reactions of two isotopologues for 1° and 2° KIE derivations at different temperatures were taken on the same day and repeated on the other day(s). For a Δ*E*_a_ determination, kinetics was determined over a temperature range of 40 °C, and *E*_aH_ and *E*_aD_ were derived. A typical kinetic procedure at a certain temperature is as follows. Six kinetic runs of 12 half-lives of the reaction were measured for each isotopic reaction in a back-to-back manner. The procedure was then repeated at other temperatures as quickly as possible (5, 15, 25, 35, and 45 °C, in order) so that the instrument settings were kept the same and the aging of the reaction solutions was minimized (while the solutions are already stable, they were wrapped with aluminum foil and kept in a refrigerator between temperatures to eliminate any possible source of error). Repetitions or kinetic measurements of the reactions of the same series of substituted substrates on different days sometimes used different batches of substrates and solvents and sometimes were done by different workers. That was to eliminate the effect of a possible different impurity from unknown sources or human errors in the KIE measurements. Therefore, one KIE value was obtained from at least 12 (mostly 18) repetitions. Pooled standard deviations were reported. All of the kinetic results [for extents of reaction of close to 1% to 99.98% (corresponding to 12 half-lives)] were fitted very well or excellently to the first-order rate law for the derivation of *k*^pfo^ and to the Arrhenius correlations for the derivation of *E*_a_, both with *R*^2^ = 0.9990–1.0000, mostly closer or sometimes even equal to 1.0000! Other details about the kinetic measurements as well as the raw data can be found in Tables S1–S13.

## Data Availability

The data underlying this study are available in the published article and its Supporting Information.

## Supporting Information Available

Rate constants, charge-transfer spectra, Hammett plots of *K*_H_- and *k*_2H_ (with σ^+^), and primary kinetic data (PDF)

## References

[ref1] Bell R. P. The tunnel effect in chemistry; Chapman & Hall: London, 1980.

[ref2] Kim Y.; Kreevoy M. M. The experimental manifestations of corner-cutting tunneling. J. Am. Chem. Soc. 1992, 114, 7116–7123. 10.1021/ja00044a024.

[ref3] Bae S. H.; Li X.-X.; Seo M. S.; Lee Y.-M.; Fukuzumi S.; Nam W. Tunneling Controls the Reaction Pathway in the Deformylation of Aldehydes by a Nonheme Iron(III)–Hydroperoxo Complex: Hydrogen Atom Abstraction versus Nucleophilic Addition. J. Am. Chem. Soc. 2019, 141, 7675–7679. 10.1021/jacs.9b02272. 31034219

[ref4] Nagel Z. D.; Klinman J. P. Update 1 of: Tunneling and dynamics in enzymatic hydride transfer. Chem. Rev. 2010, 110, PR41–PR67. 10.1021/cr1001035. 21141912 PMC4067601

[ref5] Hay S.; Scrutton N. S. Good vibrations in enzyme-catalysed reactions. Nat. Chem. 2012, 4, 161–168. 10.1038/nchem.1223. 22354429

[ref6] Kohen A. Role of Dynamics in Enzyme Catalysis: Substantial vs. Semantic Controversies. Acc. Chem. Res. 2015, 48, 466–473. 10.1021/ar500322s. 25539442 PMC4334245

[ref7] Klinman J. P.; Offenbacher A. R. Understanding Biological Hydrogen Transfer Through the Lens of Temperature Dependent Kinetic Isotope Effects. Acc. Chem. Res. 2018, 51, 1966–1974. 10.1021/acs.accounts.8b00226. 30152685 PMC6258190

[ref8] Wang Z.; Singh P.; Czekster C. M.; Kohen A.; Schramm V. L. Protein Mass-Modulated Effects in the Catalytic Mechanism of Dihydrofolate Reductase: Beyond Promoting Vibrations. J. Am. Chem. Soc. 2014, 136, 8333–8341. 10.1021/ja501936d. 24820793 PMC4063187

[ref9] Kohen A.; Cannio R.; Bartolucci S.; Klinman J. P. Enzyme dynamics and hydrogen tunnelling in a thermophilic alcohol dehydrogenase. Nature 1999, 399, 496–499. 10.1038/20981. 10365965

[ref10] Scrutton N. S.; Basran J.; Sutcliffe M. J. Review A new conceptual framework for enzyme catalysis. Eur. J. Biochem. 1999, 264, 666–671. 10.1046/j.1432-1327.1999.00645.x. 10491112

[ref11] Knapp M. J.; Rickert K.; Klinman J. P. Temperature-dependent isotope effects in soybean lipoxygenase-1: Correlating hydrogen tunneling with protein dynamics. J. Am. Chem. Soc. 2002, 124, 3865–3874. 10.1021/ja012205t. 11942823

[ref12] Sikorski R. S.; Wang L.; Markham K. A.; Rajagopalan P. T. R.; Benkovic S. J.; Kohen A. Tunneling and coupled motion in the *E. coli* dihydrofolate reductase catalysis. J. Am. Chem. Soc. 2004, 126, 4778–4779. 10.1021/ja031683w. 15080672

[ref13] Basran J.; Sutcliffe M. J.; Scrutton N. S. Enzymatic H-Transfer Requires Vibration-Driven Extreme Tunneling. Biochemistry 1999, 38, 3218–3222. 10.1021/bi982719d. 10074378

[ref14] Harris R. J.; Meskys R.; Sutcliffe M. J.; Scrutton N. S. Kinetic Studies of the Mechanism of Carbon–Hydrogen Bond Breakage by the Heterotetrameric Sarcosine Oxidase of Arthrobacter sp. 1-IN. Biochemistry 2000, 39, 1189–1198. 10.1021/bi991941v. 10684595

[ref15] Basran J.; Sutcliffe M. J.; Scrutton N. S. Deuterium Isotope Effects during Carbon–Hydrogen Bond Cleavage by Trimethylamine Dehydrogenase: Implications for mechanism and vibrationally assisted hydrogen tunneling in wild-type and mutant enzymes. J. Biol. Chem. 2001, 276, 24581–24587. 10.1074/jbc.M101178200. 11304539

[ref16] Knapp M. J.; Klinman J. P. Environmentally coupled hydrogen tunneling. Linking catalysis to dynamics. Eur. J. Biochem. 2002, 269, 3113–3121. 10.1046/j.1432-1033.2002.03022.x. 12084051

[ref17] Wang Z.; Kohen A. Thymidylate synthase catalyzed H-transfers: Two chapters in one tale. J. Am. Chem. Soc. 2010, 132, 9820–9825. 10.1021/ja103010b. 20575541 PMC2912445

[ref18] Stojkoviç V.; Perissinotti L.; Willmer D.; Benkovic S.; Kohen A. Effects of the donor acceptor distance and dynamics on hydride tunneling in the dihydrofolate reductase catalyzed reaction. J. Am. Chem. Soc. 2012, 134, 1738–1745. 10.1021/ja209425w. 22171795 PMC4341912

[ref19] Hu S.; Soudackov A. V.; Hammes-Schiffer S.; Klinman J. P. Enhanced Rigidification within a Double Mutant of Soybean Lipoxygenase Provides Experimental Support for Vibronically Nonadiabatic Proton-Coupled Electron Transfer Models. ACS Catal. 2017, 7, 3569–3574. 10.1021/acscatal.7b00688. 29250456 PMC5724529

[ref20] Pagano P.; Guo Q.; Ranasinghe C.; Schroeder E.; Robben K.; Háse F.; Ye H.; Wickersham K.; Aspuru-Guzik A.; Major D. T.; Gakhar L.; Kohen A.; Cheatum C. M. Oscillatory Active-Site Motions Correlate with Kinetic Isotope Effects in Formate Dehydrogenase. ACS Catal. 2019, 9, 11199–11206. 10.1021/acscatal.9b03345. 33996196 PMC8118594

[ref21] Ranasinghe C.; Pagano P.; Sapienza P. J.; Lee A. L.; Kohen A.; Cheatum C. M. Isotopic Labeling of Formate Dehydrogenase Perturbs the Protein Dynamics. J. Phys. Chem. B 2019, 123, 10403–10409. 10.1021/acs.jpcb.9b08426. 31696711

[ref22] Singh P.; Vandemeulebroucke A.; Li J.; Schulenburg C.; Fortunato G.; Kohen A.; Hilvert D.; Cheatum C. M. Evolution of the Chemical Step in Enzyme Catalysis. ACS Catal. 2021, 11, 6726–6732. 10.1021/acscatal.1c00442.

[ref23] Howe G. W.; van der Donk W. A. Temperature-Independent Kinetic Isotope Effects as Evidence for a Marcus-like Model of Hydride Tunneling in Phosphite Dehydrogenase. Biochemistry 2019, 58, 4260–4268. 10.1021/acs.biochem.9b00732. 31535852 PMC6852621

[ref24] Stojković V.; Kohen A. Enzymatic H-transfer: Quantum tunneling and coupled motion from kinetic isotope effects. Isr. J. Chem. 2009, 49, 163–173. 10.1560/IJC.49.2.163.

[ref25] Pudney C. R.; Johannissen L. O.; Sutcliffe M. J.; Hay S.; Scrutton N. S. Direct Analysis of Donor-Acceptor Distance and Relationship to Isotope Effects and the Force Constant for Barrier Compression in Enzymatic H-Tunneling Reactions. J. Am. Chem. Soc. 2010, 132, 11329–11335. 10.1021/ja1048048. 20698699

[ref26] Loveridge E. J.; Tey L.-H.; Allemann R. K. Solvent Effects on Catalysis by Escherichia coli Dihydrofolate Reductase. J. Am. Chem. Soc. 2010, 132, 1137–1143. 10.1021/ja909353c. 20047317

[ref27] Romero E.; Ladani S. T.; Hamelberg D.; Gadda G. Solvent-Slaved Motions in the Hydride Tunneling Reaction Catalyzed by Human Glycolate Oxidase. ACS Catal. 2016, 6, 2113–2120. 10.1021/acscatal.5b02889.

[ref28] Hay S.; Sutcliffe M. J.; Scruton N. S. Probing Coupled Motions in Enzymatic Hyfrogen Tunneling Reactions: Beyond Temperature-Dependence Studies of Kinetic Isotope Effects. In Quantum Tunnelling in Enzyme-Catalyzed Reactions; Scrutton N. S., Allemann R. K., Eds.; RSC Publishing, 2009; pp 199–218.

[ref29] Loveridge E. J.; Allemann R. Direct Methods for the Analysis of Quantum-Mechanical Tunneling: Dihydrofolate Reductase. In Quantum Tunnelling in Enzyme-Catalyzed Reactions; Scrutton N. S., Allemann R. K., Eds.; RSC Publishing, 2009; pp 179–198.

[ref30] Benkovic S. J.; Hammes-Schiffer S. A perspective on enzyme catalysis. Science 2003, 301, 1196–1202. 10.1126/science.1085515. 12947189

[ref31] Klinman J. P.; Kohen A. Hydrogen Tunneling Links Protein Dynamics to Enzyme Catalysis. Annu. Rev. Biochem. 2013, 82, 471–496. 10.1146/annurev-biochem-051710-133623. 23746260 PMC4066974

[ref32] Schramm V. L.; Schwartz S. D. Promoting vibrations and the function of enzymes. Emerging theoretical and experimental convergence. Biochemistry 2018, 57, 3299–3308. 10.1021/acs.biochem.8b00201. 29608286 PMC6008225

[ref33] Schwartz S. D. Protein Dynamics and Enzymatic Catalysis. J. Phys. Chem. B 2023, 127, 2649–2660. 10.1021/acs.jpcb.3c00477. 36944023 PMC10072970

[ref34] Kuznetsov A. M.; Ulstrup J. Proton and hydrogen atom tunneling in hydrolytic and redox enzyme catalysis. Can. J. Chem. 1999, 77, 1085–1096. 10.1139/v99-099.

[ref35] Klinman J. P. A new model for the origin of kinetic hydrogen isotope effects. J. Phy. Org. Chem. 2010, 23, 606–612. 10.1002/poc.1661.

[ref36] Layfield J. P.; Hammes-Schiffer S. Hydrogen Tunneling in Enzymes and Biomimetic Models. Chem. Rev. 2014, 114, 3466–3494. 10.1021/cr400400p. 24359189 PMC3981923

[ref37] Ludlow M. K.; Soudackov A. V.; Hammes-Schiffer S. Theoretical Analysis of the Unusual Temperature Dependence of the Kinetic Isotope Effect in Quinol Oxidation. J. Am. Chem. Soc. 2009, 131, 7094–7102. 10.1021/ja9001184. 19351186 PMC2710000

[ref38] Hothi P.; Hay S.; Roujeinikova A.; Sutcliffe M. J.; Lee M.; Leys D.; Cullis P. M.; Scrutton N. S. Driving Force Analysis of Proton Tunnelling Across aReactivity Series for an Enzyme-Substrate Complex. Chem. Bio. Chem. 2008, 9, 2839–2845. 10.1002/cbic.200800408. 19012287

[ref39] Roston D.; Kohen A. Elusive transition state of alcohol dehydrogenase unveiled. Proc. Nat. Acad. Sci. USA 2010, 107, 9572–9577. 10.1073/pnas.1000931107. 20457944 PMC2906880

[ref40] Kashefolgheta S.; Razzaghi M.; Hammann B.; Eilers J.; Roston D.; Lu Y. Computational Replication of the Abnormal Secondary Kinetic Isotope Effects in a Hydride Transfer Reaction in Solution with a Motion Assisted H-Tunneling Model. J. Org. Chem. 2014, 79, 1989–1994. 10.1021/jo402650a. 24498946 PMC3985929

[ref41] Derakhshani-Molayousefi M.; Kashefolgheta S.; Eilers J. E.; Lu Y. Computational Replication of the Primary Isotope Dependence of Secondary Kinetic Isotope Effects in Solution Hydride-Transfer Reactions: Supporting the Isotopically Different Tunneling Ready State Conformations. J. Phys. Chem. A 2016, 120, 4277–4284. 10.1021/acs.jpca.6b03571. 27232375

[ref42] Bai M.; Koirala S.; Lu Y. Direct Correlation Between Donor-Acceptor Distance and Temperature Dependence of Kinetic Isotope Effects in Hydride-Tunneling Reactions of NADH/NAD+ Analogues. J. Org. Chem. 2021, 86, 7500–7507. 10.1021/acs.joc.1c00497. 34037396

[ref43] Bai M.; Pratap R.; Salarvand S.; Lu Y. Correlation of Temperature Dependence of Hydride Kinetic Isotope Effects with Donor-Acceptor Distances in Two Solvents of Different Polarities. Org. Biol. Chem. 2023, 21, 5090–5097. 10.1039/D3OB00718A. PMC10339711 37278324

[ref44] Pu J.; Ma S.; Gao J.; Truhlar D. G. Small temperature dependence of the kinetic isotope effect for the hydride transfer reaction catalyzed by Escherichia coli dihydrofolate reductase. J. Phys. Chem. B 2005, 109, 8551–8556. 10.1021/jp051184c. 16852008 PMC4476250

[ref45] Liu H.; Warshel A. Origin of the Temperature Dependence of Isotope Effects in Enzymatic Reactions: The Case of Dihydrofolate Reductase. J. Phys. Chem. B 2007, 111, 7852–7861. 10.1021/jp070938f. 17571875

[ref46] Liu Q.; Zhao Y.; Hammann B.; Eilers J.; Lu Y.; Kohen A. A Model Reaction Assesses Contribution of H-Tunneling and Coupled Motions to Enzyme Catalysis. J. Org. Chem. 2012, 77, 6825–6833. 10.1021/jo300879r. 22834675

[ref47] Lu Y.; Wilhelm S.; Bai M.; Maness P.; Ma L. Replication of the Enzymatic Temperature Dependency of the Primary Hydride Kinetic Isotope Effects in Solution: Caused by the Protein Controlled Rigidity of the Donor-Acceptor Centers?. Biochemistry 2019, 58, 4035–4046. 10.1021/acs.biochem.9b00574. 31478638

[ref48] Maness P.; Koirala S.; Adhikari P.; Salimraftar N.; Lu Y. Substituent Effects on Temperature Dependence of Kinetic Isotope Effects in Hydride-Transfer Reactions of NADH/NAD+ Analogues in Solution: Reaction Center Rigidity Is the Key. Org. Lett. 2020, 22, 5963–5967. 10.1021/acs.orglett.0c02049. 32662653

[ref49] Adhikari P.; Song M.; Bai M.; Rijal P.; DeGroot N.; Lu Y. Solvent Effects on the Temperature Dependence of Hydride Kinetic Isotope Effects: Correlation to the Donor–Acceptor Distances. J. Phys. Chem. A 2022, 126, 7675–7686. 10.1021/acs.jpca.2c06065. 36228057

[ref50] Fukuzumi S.; Ohkubo K.; Tokuda Y.; Suenobu T. Hydride Transfer from 9-Substituted 10-Methyl-9,10-dihydroacridines to Hydride Acceptors via Charge-Transfer Complexes and Sequential Electron-Proton-Electron Transfer. A Negative Temperature Dependence of the Rates. J. Am. Chem. Soc. 2000, 122, 4286–4294. 10.1021/ja9941375.

[ref51] Lu Y.; Zhao Y.; Parker V. D. Proton-transfer reactions of methylarene radical cations with pyridine bases under non-steady-state conditions. Real kinetic isotope effect evidence for extensive tunneling. J. Am. Chem. Soc. 2001, 123, 5900–5907. 10.1021/ja010271p. 11414822

[ref52] Zhu X. Q.; Zhang J. Y.; Cheng J.-P. Negative Kinetic Temperature Effect on the Hydride Transfer from NADH Analogue BNAH to the Radical Cation of N-Benzylphenothiazine in Acetonitrile”. J. Org. Chem. 2006, 71, 7007–7015. 10.1021/jo061145c. 16930056

[ref53] Lu Y.; Zhao Y.; Handoo K. L.; Parker V. D. Hydride-exchange reactions between NADH and NAD+ model compounds under non-steady-state conditions. Apparent and Real kinetic isotope effects. Org. Biomol. Chem. 2003, 1, 173–181. 10.1039/b208186e. 12929407

[ref54] Bunting J. W.; Sindhuatmadja S. Kinetics and mechanism of the reaction of 5-nitroisoquinolinium cations with 1,4-dihydronicotinamides. J. Org. Chem. 1981, 46, 4211–4219. 10.1021/jo00334a022.

[ref55] Zhu X. Q.; Deng F. H.; Yang J. D.; Li X. T.; Chen Q.; Lei N. P.; Meng F. K.; Zhao X. P.; Han S. H.; Hao E. J.; Mu Y. Y. A classical but new kinetic equation for hydride transfer reactions. Org. Biomol. Chem. 2013, 11, 6071–6089. 10.1039/c3ob40831k. 23917398

[ref56] Ma L.; Sakhaee N.; Jafari S.; Wilhelm S.; Rahmani P.; Lu Y. Imbalanced Transition States from α-H/D and Remote β-Type N-CH/D Secondary Kinetic Isotope Effects on the NADH/NAD+ Analogues in Their Hydride Tunneling Reactions in Solution. J. Org. Chem. 2019, 84, 5431–5439. 10.1021/acs.joc.9b00412. 30912443

[ref57] Perrin C. L.; Ohta B. K.; Kuperman J.; Liberman J.; Erdelyi M. Stereochemistry of Beta-Deuterium Isotope Effects on Amine Basicity. J. Am. Chem. Soc. 2005, 127, 9641–9647. 10.1021/ja0511927. 15984892

[ref58] Perrin C. L. The Logic behind a Physical–Organic Chemist’s Research Topics. J. Org. Chem. 2017, 82, 819–838. 10.1021/acs.joc.6b02390. 28103693

[ref59] Powell M. F.; Bruice T. C. Effect of isotope scrambling and tunneling on the kinetic and product isotope effects for reduced nicotinamide adenine dinucleotide model hydride transfer reactions. J. Am. Chem. Soc. 1983, 105, 7139–7149. 10.1021/ja00362a019.

[ref60] Lee I.-S. H.; Jeoung E. H.; Kreevoy M. M. Primary Kinetic Isotope Effects on Hydride Transfer from 1,3-Dimethyl-2-phenylbenzimidazoline to NAD+ Analogues. J. Am. Chem. Soc. 2001, 123, 7492–7496. 10.1021/ja004232+. 11480968

[ref61] Kil H. J.; Lee I.-S. H. Primary Kinetic Isotope Effects on Hydride Transfer from Heterocyclic Compounds to NAD + Analogues. J. Phs. Chem. A 2009, 113, 10704–10709. 10.1021/jp905937x. 19746950

[ref62] Kreevoy M. M.; Ostovic D.; Truhlar D. G.; Garrett B. C. Phenomenological manifestations of large-curvature tunneling in hydride-transfer reactions. J. Phys. Chem. 1986, 90, 3766–3774. 10.1021/j100407a052.

[ref63] Roston D.; Cheatum C. M.; Kohen A. Hydrogen Donor-Acceptor Fluctuations from Kinetic Isotope Effects: A Phenomenological Model. Biochemistry 2012, 51, 6860–6870. 10.1021/bi300613e. 22857146 PMC3448806

[ref64] Schowen R. L. The Strengths and Weaknesses of Model Reactions for the Assesement if Tunneling in Enzymic Reactions. In Quantum tunnelling in enzyme catalyzed reactions; Allemann R., Scrutton N., Eds.; Royal Society of Chemistry: London, 2009; Chapter 13, pp 291–313.

[ref65] Horitani M.; Offenbacher A. R.; Carr C. A. M.; Yu T.; Hoeke V.; Cutsail G. E.; Hammes-Schiffer S.; Klinman J. P.; Hoffman B. M. C-13 ENDOR Spectroscopy of Lipoxygenase-Substrate Complexes Reveals the Structural Basis for C-H Activation by Tunneling. J. Am. Chem. Soc. 2017, 139, 1984–1997. 10.1021/jacs.6b11856. 28121140 PMC5322796

[ref66] Lu Y.; Qu F.; Moore B.; Endicott D.; Kuester W. Hydride Reduction of NAD+ Analogues by Isopropyl Alcohol: Kinetics, Deuterium Isotope Effects and Mechanism. J. Org. Chem. 2008, 73, 4763–4770. 10.1021/jo800820u. 18543993

[ref67] Maharjan B.; Raghibi Boroujeni M.; Lefton J.; White O. R.; Razzaghi M.; Hammann B. A.; Derakhshani-Molayousefi M.; Eilers J. E.; Lu Y. Steric Effects on the Primary Isotope Dependence of Secondary Kinetic Isotope Effects in Hydride Transfer Reactions in Solution: Caused by the Isotopically Different Tunneling Ready State Conformations?. J. Am. Chem. Soc. 2015, 137, 6653–6661. 10.1021/jacs.5b03085. 25941865

[ref68] Zhu X.-Q.; Zhang M.-T.; Yu A.; Wang C.-H.; Cheng J.-P. Hydride, Hydrogen Atom, Proton, and Electron Transfer Driving Forces of Various Five-Membered Heterocyclic Organic Hydrides and Their Reaction Intermediates in Acetonitrile. J. Am. Chem. Soc. 2008, 130, 2501–2516. 10.1021/ja075523m. 18254624

[ref69] Fukuzumi S.; Tokuda Y.; Kitano T.; Okamoto T.; Otera J. Electron-transfer oxidation of 9-substituted 10-methyl-9,10-dihydroacridines. Cleavage of the carbon-hydrogen vs. carbon-carbon bond of the radical cations. J. Am. Chem. Soc. 1993, 115, 8960–8968. 10.1021/ja00073a010.

[ref70] Shen G. B.; Xia K.; Li X. T.; Li J. L.; Fu Y. H.; Yuan L.; Zhu X. Q. Prediction of Kinetic Isotope Effects for Various Hydride Transfer Reactions Using a New Kinetic Model. J. Phys. Chem. A 2016, 120, 1779–1799. 10.1021/acs.jpca.5b10135. 26938149

