# Supplementary material for: Structural Effects on the Temperature Dependence of Hydride Kinetic Isotope Effects of the NADH/NAD+ Model Reactions in Acetonitrile: Charge-Transfer Complex Tightness Is a Key
Source: J Org Chem. 2024 Feb 16;89(5):3184–93. doi: 10.1021/acs.joc.3c02562 (PMC10913049; doi:10.1021/acs.joc.3c02562)
Supplement: Supplementary file 1 — jo3c02562_si_001.pdf [file jo3c02562_si_001.pdf]

Supporting Information

Structural Effects on the Temperature Dependence of Hydride Kinetic Isotope Effects of the NADH/NAD^+ Model Reactions in Acetonitrile: Charge-Transfer Complex Tightness Is a Key

Amanda Beach, Pratichhya Adhikari, Grishma Singh, Meimei Song, Nicholas DeGroot, Yun Lu*

Department of Chemistry, Southern Illinois University Edwardsville, Edwardsville, Illinois 62026, United States

yulu@siue.edu

Spectroscopic evidence for charge-transfer complexes (Figure S1)…………………………………….. S2

Hammett correlations of reaction rates (Figures S2-5)…………………..…………..………………...…S3

Raw rate and equilibrium constants (Tables S1-13) …....……………..……………………………………. S6

Synthesis of *G*PhMA$^+$ ……………………………………………………………………………… S10

Synthesis of *G*DMPBIH ……………………………………………………………………………… S11

References ……………………………………………………………………………………………... S11

Data availability statement.……………………………………………………………………….. S12

Spectroscopic Evidence for Charge-Transfer Complexes

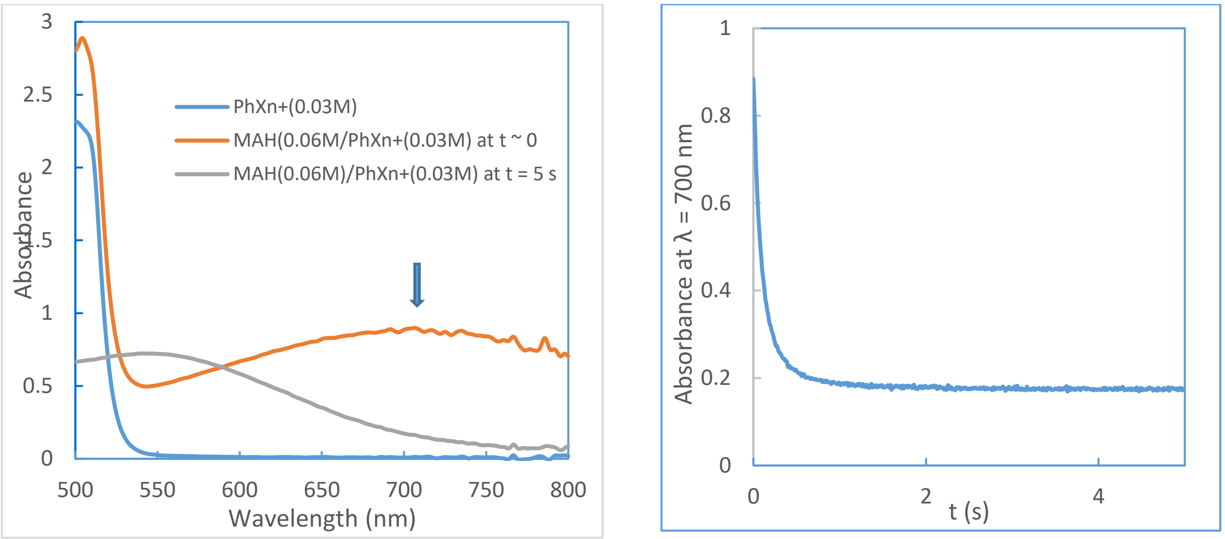

Figure S1. Left: The CT absorption bands of the hydride transfer reaction from MAH to $PhXn^+$ in acetonitrile at 5 °C, measured upon mixing of the reactant solutions (in orange color) and at the end of the reaction (at 5 s of the reaction, in grey color). The grey spectrum is the CT absorption band of the products complex. The blue spectrum is the $PhXn^+$ only. Right: The decay of the CT absorption at 700 nm with time for the reaction of MAH (0.06 M) with $PhXn^+$ (0.03 M) in acetonitrile at 5 °C.

Hammett Correlations with the Fitting Parameters

Note that in the main paper, it is noted that the Hammett correlations are generally better with σ than σ^+.

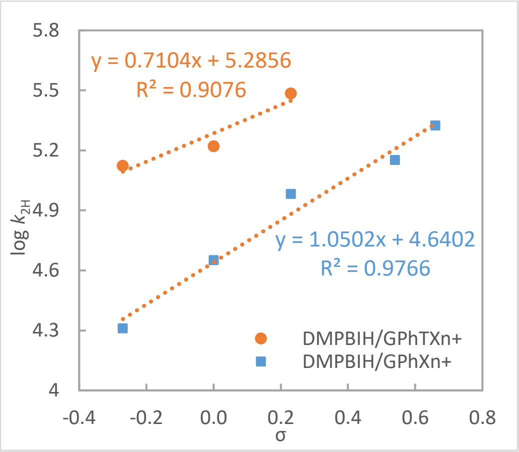

*G*PhXn$^+$: *G* = MeO, H, Br, CF_3, CN (*c.f.* Ref[1])
*G*PhTXn$^+$: *G* = MeO, H, Cl (*c.f.* Table S3)

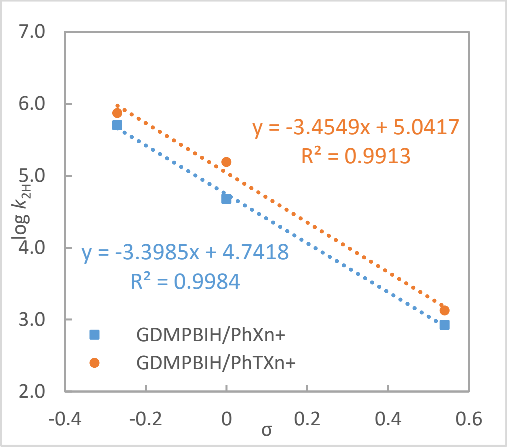

*G*DMPBIH: *G* = MeO, H, CF_3 (*c.f.* Table S4)
*G*DMPBIH: *G* = MeO, H, CF_3 (*c.f.* Table S4)

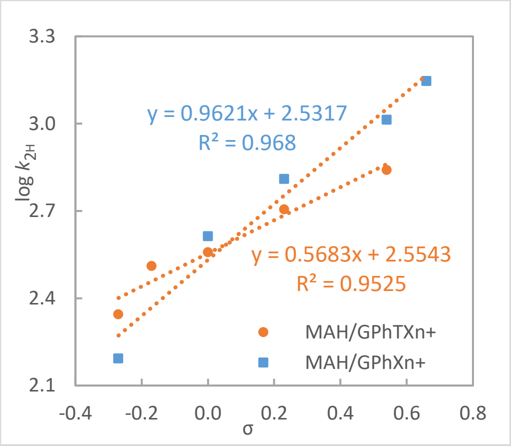

*G*PhXn$^+$: *G* = MeO, H, Br, CF_3, CN (*c.f.* Ref[1])
*G*PhTXn$^+$: *G* = MeO, Me, H, Cl, CF_3 (*c.f.* Ref[2])

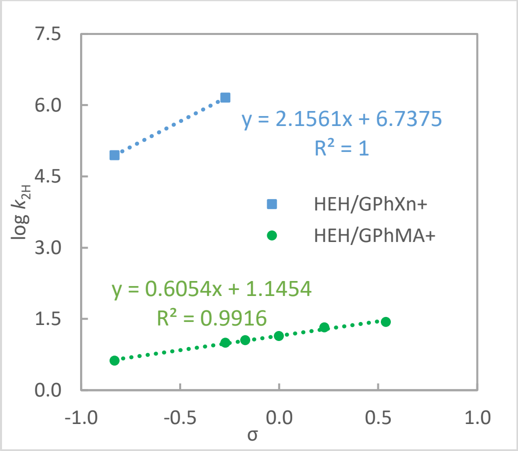

*G*PhXn$^+$: *G* = Me_2N (*c.f.* Table S5), MeO (*c.f.* Ref[3])
*G*PhMA$^+$: *G* = Me_2N, MeO, Me, H, Br, CF_3 (*c.f.* Tables S6-11)

Figure S2. Hammett correlations of k_{2H} with σ (Same as the Figures 2-4 in the paper, but this is to show the fitting parameters. The sources of the raw k_{2H} data is indicated below each plot.)

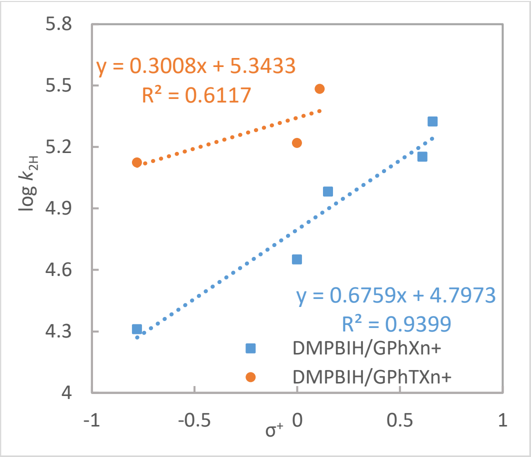

*G*PhXn$^+$: *G* = MeO, H, Br, CF_3, CN (*c.f.* Ref[1])
*G*PhTXn$^+$: *G* = MeO, H, Cl (*c.f.* Table S3)

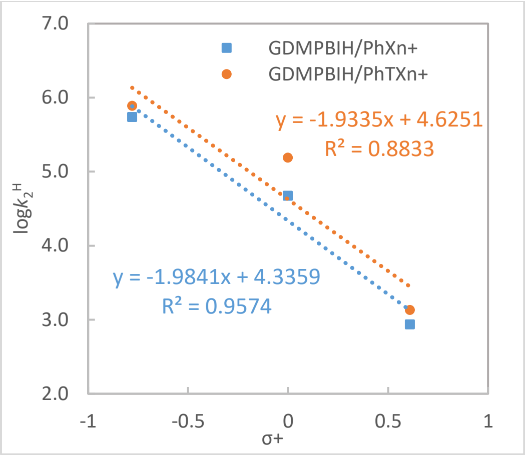

*G*DMPBIH: *G* = MeO, H, CF_3 (*c.f.* Table S4)
*G*DMPBIH: *G* = MeO, H, CF_3 (*c.f.* Table S4)

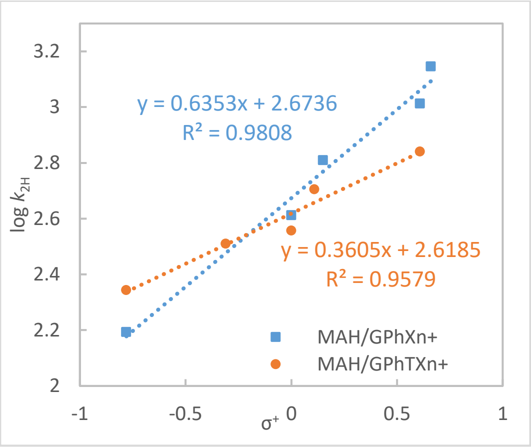

*G*PhXn$^+$: *G* = MeO, H, Br, CF_3, CN (*c.f.* Ref[1])
*G*PhTXn$^+$: *G* = MeO, Me, H, Cl, CF_3 (*c.f.* Ref[2])

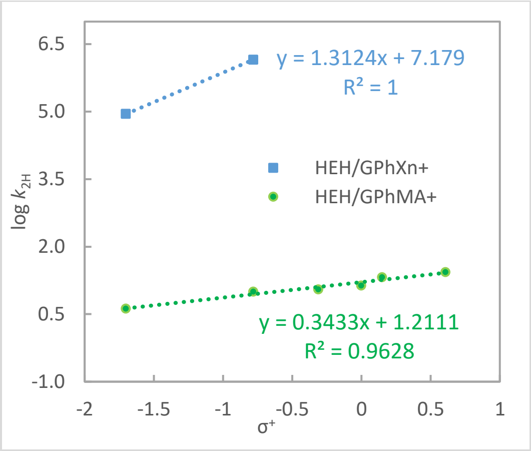

*G*PhXn$^+$: *G* = Me_2N (*c.f.* Table S5), MeO (*c.f.* Ref[3])
*G*PhMA$^+$: *G* = Me_2N, MeO, Me, H, Br, CF_3 (*c.f.* Tables S6-11)

Figure S3. Hammett correlations of k_{2H} with σ^+ (The sources of the raw k_{2H} data are indicated below each plot.)

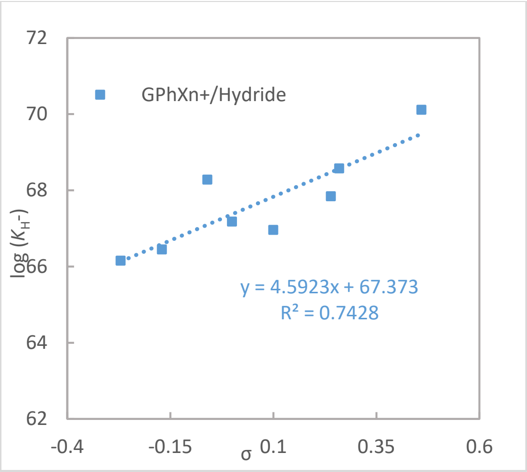

*G*PhXn⁺: *G* = MeO, Me, H, Cl, Br, m-MeO, m-Me, m-CF_3

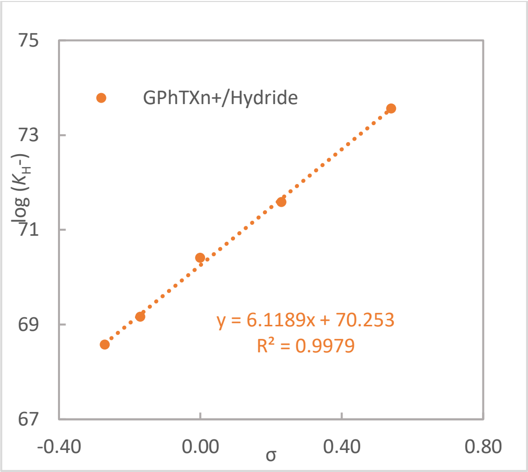

*G*PhTXn+: *G* = MeO, Me, H, Cl, CF_3

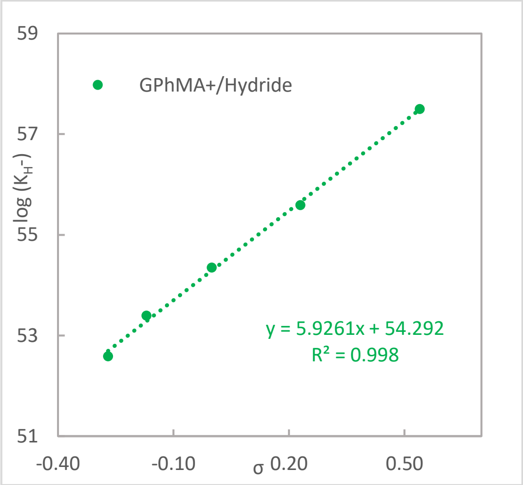

*G*PhMA⁺: *G* = MeO, Me, H, Cl, CF_3

Figure S4. Hammett correlations of K_{H^-} with σ (The raw K_{H^-} data are listed in Table S12.)

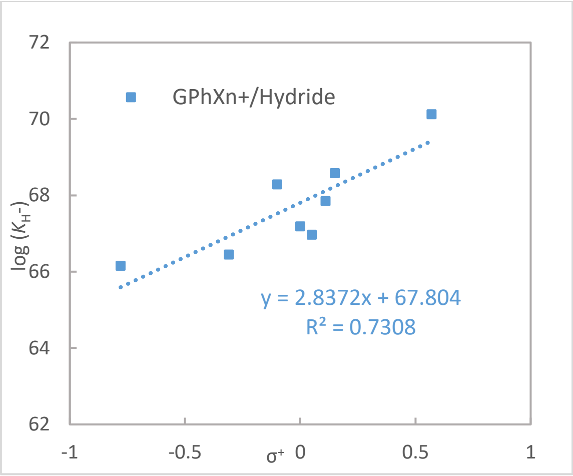

*G*PhXn⁺: *G* = MeO, Me, H, Cl, Br, m-MeO, m-Me, m-CF_3

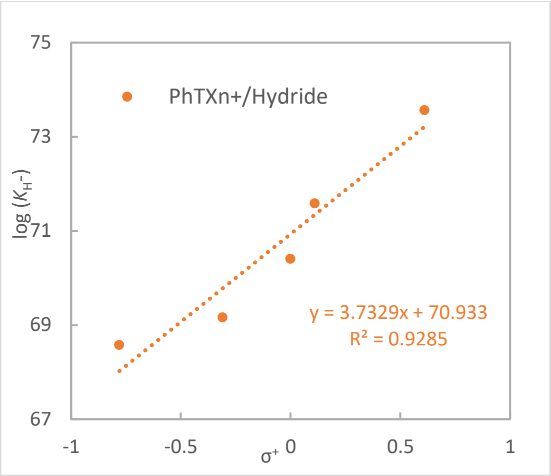

*G*DMPBIH: *G* = MeO, Me, H, Cl, CF_3

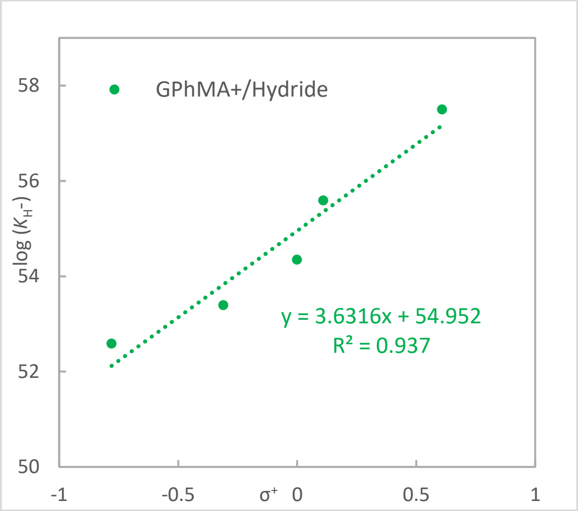

*G*PhMA⁺: *G* = MeO, Me, H, Cl, CF_3

Figure S5. Hammett correlations of K_{H^-} with σ^+ (The raw K_{H^-} data are listed in Table S12.)

Raw Kinetic/Thermodynamic Data

Table S1. The temperature effects on the rate constants and 1° KIEs of the hydride transfer reaction from DMPBIH to $PhTXn^+$ in acetonitrile [a,b]

**Temp (°C)**	k_{2H} **($M^{-1}s^{-1}$)**	k_{2D} **($M^{-1}s^{-1}$)**	**1° KIE**
45.0	2.19(0.03) x 10^5	7.19(0.06) x 10^4	3.04 (0.05)
35.0	1.93(0.02) x 10^5	6.05(0.06) x 10^4	3.19 (0.05)
25.0	1.66(0.02) x 10^5	4.97(0.05) x 10^4	3.33 (0.05)
15.0	1.37(0.01) x 10^5	3.90(0.04) x 10^4	3.52 (0.05)
5.0	1.15(0.02) x 10^5	3.16(0.03) x 10^4	3.63 (0.06)

[a] Repeated on three different days with 6 repetitions each day. Numbers in parentheses are the pooled standard deviations S(pooled). [b] [DMPBIH] = 1.625x10^{-3} M, [$PhTXn^+$] = 1.625x10^{-4} M. Absorbance decay at 493 nm due to $PhTXn^+$ was followed for kinetic measurements.

Table S2. The temperature effects on the rate constants and 1° KIEs of the hydride transfer reaction from MAH to $PhTXn^+$ in acetonitrile [a,b]

**Temp (°C)**	k_{2H} **($M^{-1}s^{-1}$)**	k_{2D} **($M^{-1}s^{-1}$)**	**1° KIE**
45.0	8.39(0.04) x 10^2	1.91(0.01) x 10^2	4.39 (0.03)
35.0	5.70(0.02) x 10^2	1.23(0.01) x 10^2	4.64 (0.03)
25.0	3.66(0.02) x 10^2	7.69(0.01) x 10	4.76 (0.05)
15.0	2.32(0.02) x 10^2	4.47(0.01) x 10	5.20 (0.08)
5.0	1.41(0.02) x 10^2	2.53(0.00$_3$) x 10	5.58 (0.09)

[a] Repeated on three different days with 6 repetitions each day. Numbers in parentheses are the pooled standard deviations S(pooled). [b] [MAH] = 3.75x10^{-3} M, [$PhTXn^+$] = 2.50x10^{-4} M. Absorbance decay at 493 nm due to $PhTXn^+$ was followed for kinetic measurements.

Table S3. The rate constants of the hydride transfer reaction from DMPBIH to *G*$PhTXn^+$ in acetonitrile at 25 °C [a]

G$PhTXn^+$	$k_{2H}^{25°C}$ **($M^{-1}s^{-1}$)** [b]
MeO	1.33(0.01) x 10^5
H	1.66(0.02) x 10^5
Cl	3.05(0.09) x 10^5

[a] Repeated on three different days with 6 repetitions each day. Numbers in parentheses are the pooled standard deviations S(pooled). [b] For G = Cl, [DMPBIH] = 1.50x10^{-3} M, [$ClPhTXn^+$] = 1.50x10^{-4} M; for G= H, [DMPBIH] = 1.625x10^{-3} M, [$MeOPhTXn^+$] = 1.625x10^{-4} M; G = MeO, [DMPBIH] = 1.00x10^{-3} M, [$MeOPhTXn^+$] = 1.00x10^{-4} M; Absorbance decay at 497nm, 493nm, 530nm due to Cl, H and MeO substituted $PhTXn^+$ were followed for kinetic measurements.

Table S4. The rate constants of the hydride transfer reaction from *G*DMPBIH to $PhXn^+$ and $PhTXn^+$ in acetonitrile at 25 °C [a]

*G*DMPBIH	$k_{2H}^{25°C}$ **($M^{-1}s^{-1}$)** [b]	*G*DMPBIH	$k_{2H}^{25°C}$ **($M^{-1}s^{-1}$)** [c]
	With $PhXn^+$		With $PhTXn^+$
5-MeO	5.04(0.19) x 10^5 [d]	5-MeO	7.48(0.33) x 10^5 [d]
H	4.76(0.03) x 10^4	H	166(0.02) x 10^5
5-CF_3	8.47(0.06) x 10^2	5-CF_3	1.34(0.07) x 10^3

[a] Repeated on two different days with 12 repetitions each day. Numbers in parentheses are the pooled standard deviations S(pooled). [b] [GDMPBIH] = 1.22x10^{-3} M, [$PhXn^+$] = 4.00x10^{-5} M. Absorbance decay at 373 nm due to $PhXn^+$ was followed for kinetic measurements; [b] [*G*DMPBIH] = 1.22x10^{-3} M, [$PhTXn^+$] = 1.22x10^{-4} M. Absorbance decay at 373 nm due to $PhTXn^+$ was followed for kinetic measurements; [d] These two rate constants are less reliable as they have reached the limit of rate measurement from our stopped-flow instrument (Use of the data for discussion has been limited, see the main paper.)

Table S5. The temperature effects on the rate constants and 1° KIEs of the hydride transfer reaction from HEH to *Me$_2$N*$PhXn^+$ in acetonitrile [a,b]

**Temp (°C)**	k_{2H} **($M^{-1}s^{-1}$)**	k_{2D} **($M^{-1}s^{-1}$)**	**1° KIE**
45.0	1.19(0.01) x 10^5	3.61(0.06) x 10^4	3.29 (0.06)
35.0	1.04(0.01) x 10^5	3.06(0.02) x 10^4	3.39 (0.04)
25.0	8.87(0.05) x 10^4	2.49(0.01) x 10^4	3.56 (0.03)
15.0	7.50(0.04) x 10^4	2.01(0.01) x 10^4	3.74 (0.03)
5.0	6.26(0.06) x 10^4	1.57(0.01) x 10^4	3.99 (0.05)

[a] Repeated on three different days with 6 repetitions each day. Numbers in parentheses are the pooled standard deviations S(pooled). [b] [HEH] = 4.125x10^{-4} M, [*Me$_2$N*$PhXn^+$] = 3.75x10^{-5} M. Absorbance decay at 660 nm due to *Me$_2$N*$PhMA^+$ was followed for kinetic measurements.

Table S6. The temperature effects on the rate constants and 1° KIEs of the hydride transfer reaction from HEH to *Me₂N*PhMA⁺ in acetonitrile [a,b]

Temp (°C)	k_{2H} ($M^{-1}s^{-1}$)	k_{2D} ($M^{-1}s^{-1}$)	1° KIE
55.0	1.32(0.01) x 10	3.10(0.02)	4.25 (0.03)
45.0	9.28(0.06)	2.07(0.02)	4.48 (0.05)
35.0	6.37(0.02)	1.32(0.01)	4.81 (0.03)
25.0	4.19(0.03)	8.23(0.01) x 10^{-1}	5.09 (0.06)
15.0	2.64(0.02)	4.72(0.01) x 10^{-1}	5.58 (0.07)

[a] Repeated on two different days with 6 repetitions each day. Numbers in parentheses are the pooled standard deviations S(pooled). [b] [HEH] = 6.00x10^{-3} M, [*Me_2N*$PhMA^+$] = 1.00x10^{-4} M. Absorbance decay at 560 nm due to *Me_2N*$PhMA^+$ was followed for kinetic measurements.

Table S7. The temperature effects on the rate constants and 1° KIEs of the hydride transfer reaction from HEH to *MeO*$PhMA^+$ in acetonitrile [a,b]

Temp (°C)	k_{2H} ($M^{-1}s^{-1}$)	k_{2D} ($M^{-1}s^{-1}$)	1° KIE
55.0	2.83(0.02) x 10	6.78(0.05)	4.18 (0.05)
45.0	2.03(0.01) x 10	4.63(0.05)	4.38 (0.05)
35.0	1.48(0.02) x 10	3.09(0.02)	4.79 (0.06)
25.0	1.00(0.01) x 10	1.96(0.02)	5.11 (0.08)
15.0	6.74(0.07)	1.23(0.01)	5.48 (0.08)

[a] Repeated on two different days with 6 repetitions each day. Numbers in parentheses are the pooled standard deviations S(pooled). [b] [HEH] = 6.00x10^{-3} M, [*MeO*$PhMA^+$] = 1.00x10^{-4} M. Absorbance decay at 434 nm due to *MeO*$PhMA^+$ was followed for kinetic measurements.

Table S8. The temperature effects on the rate constants and 1° KIEs of the hydride transfer reaction from HEH to *Me*$PhMA^+$ in acetonitrile [a,b]

Temp (°C)	k_{2H} ($M^{-1}s^{-1}$)	k_{2D} ($M^{-1}s^{-1}$)	1° KIE
55.0	3.16(0.03) x 10	7.50(0.06)	4.21 (0.05)
45.0	2.30(0.02) x 10	5.09(0.04)	4.52 (0.05)
35.0	1.66(0.02) x 10	3.39(0.05)	4.90 (0.08)
25.0	1.13(0.02) x 10	2.13(0.02)	5.30 (0.10)
15.0	7.51(0.05)	1.35(0.02)	5.55 (0.08)

[a] Repeated on two different days with 6 repetitions each day. Numbers in parentheses are the pooled standard deviations S(pooled). [b] [HEH] = 6.00x10^{-3} M, [*Me*$PhMA^+$] = 1.00x10^{-4} M. Absorbance decay at 426 nm due to *Me*$PhMA^+$ was followed for kinetic measurements.

Table S9. The temperature effects on the rate constants and 1° KIEs of the hydride transfer reaction from HEH to $PhMA^+$ in acetonitrile [a,b]

Temp (°C)	k_{2H} ($M^{-1}s^{-1}$)	k_{2D} ($M^{-1}s^{-1}$)	1° KIE
55.0	3.91(0.03) x 10	8.84(0.05)	4.42 (0.04)
45.0	2.83(0.02) x 10	6.00(0.06)	4.72 (0.06)
35.0	1.99(0.01) x 10	3.99(0.03)	4.98 (0.05)
25.0	1.37(0.08) x 10	2.58(0.01)	5.31 (0.04)
15.0	9.03(0.05)	1.58(0.01)	5.71 (0.04)

[a] Repeated on three different days with 6 repetitions each day. Numbers in parentheses are the pooled standard deviations S(pooled). [b] [HEH] = 6.00x10^{-3} M, [$PhMA^+$] = 1.50x10^{-4} M. Absorbance decay at 436 nm due to $PhMA^+$ was followed for kinetic measurements.

Table S10. The temperature effects on the rate constants and 1° KIEs of the hydride transfer reaction from HEH to *Br*PhMA$^+$ in acetonitrile [a,b]

Temp (°C)	k_{2H} ($M^{-1}s^{-1}$)	k_{2D} ($M^{-1}s^{-1}$)	1° KIE
55.0	5.59(0.04) x 10	1.34(0.01) x 10	4.10 (0.04)
45.0	4.09(0.01) x 10	9.22(0.08)	4.43 (0.04)
35.0	3.02(0.03) x 10	6.25(0.05)	4.82 (0.07)
25.0	2.09(0.03) x 10	4.02(0.03)	5.20 (0.08)
15.0	1.42(0.01) x 10	2.59(0.04)	5.50 (0.10)

[a] Repeated on two different days with 6 repetitions each day. Numbers in parentheses are the pooled standard deviations S(pooled). [b] [HEH] = 6.00x10^{-3} M, [*Br*PhMA$^+$] = 1.20x10^{-4} M. Absorbance decay at 425 nm due to *Br*PhMA$^+$ was followed for kinetic measurements.

Table S11. The temperature effects on the rate constants and 1° KIEs of the hydride transfer reaction from HEH to *CF₃*PhMA$^+$ in acetonitrile [a,b]

Temp (°C)	k_{2H} ($M^{-1}s^{-1}$)	k_{2D} ($M^{-1}s^{-1}$)	1° KIE
55.0	7.28(0.11) x 10	1.77(0.01) x 10	4.13 (0.06)
45.0	5.29(0.04) x 10	1.18(0.00$_4$) x 10	4.50 (0.03)
35.0	3.93(0.04) x 10	8.06(0.15)	4.88 (0.10)
25.0	2.74(0.03) x 10	5.23(0.02)	5.23 (0.05)
15.0	1.83(0.09) x 10	3.39(0.05)	5.39 (0.21)

[a] Repeated on two different days with 6 repetitions each day. Numbers in parentheses are the pooled standard deviations S(pooled). [b] [HEH] = 6.00x10^{-3} M, [CF_3PhMA$^+$] = 1.50x10^{-4} M. Absorbance decay at 424 nm due to CF_3PhMA$^+$ was followed for kinetic measurements.

Table S12. Hydride affinities ($-\Delta G^o_{H^-}$, kcal/mol) and equilibrium constants (K_{H^-}, M^{-1}) for hydride acceptors to accept a hydride ion in acetonitrile [a]

	$-\Delta G^o_{H^-}$	K_{H^-}		$-\Delta G^o_{H^-}$	K_{H^-}		$-\Delta G^o_{H^-}$	K_{H^-}
	*G*PhXn$^+$			*G*PhTXn$^+$			*G*PhMA$^+$	
Br	93.5	3.75E+68	CF_3	100.3	3.64E+73	CF_3	78.4	3.16E+57
Cl	92.5	6.93E+67	Cl	97.6	3.81E+71	Cl	75.8	3.92E+55
H	91.6	1.52E+67	H	96	2.56E+70	H	74.1	2.22E+54
Me	90.6	2.80E+66	Me	94.3	1.45E+69	Me	72.8	2.47E+53
MeO	90.2	1.43E+66	MeO	93.5	3.75E+68	MeO	71.7	3.85E+52
m-MeO	91.3	9.14E+66						
m-Me	93.1	1.91E+68						
m-CF3	95.6	1.30E+70						

[a] $-\Delta G^o_{H^-}$ at 25 °C are from ref[2]

Table S13. The 1,3-N,N-2CH_3/CD_3 γ-2° KIEs on DMPBIH for its reactions with PhXn$^+$ and PhTXn$^+$ in acetonitrile

Measurements	k^{pfo}(1,3-2CH_3) (s^{-1}) [a,b]	k^{pfo}(1,3-2CD_3) (s^{-1}) [a,b]	A_{343nm}(DMPBIH (γ,γ-2CH_3))	A_{343nm}(DMPBIH (γ,γ-2CD_3))	γ-2° KIE [c]
With PhXn$^+$					
1	56.959 (0.277)	60.624 (0.403)	1.11598	1.04573	0.880 (0.007)
2	57.234 (0.496)	66.199 (0.436)	1.0936	1.13395	0.896 (0.010)
3	55.685 (0.304)	48.650 (0.389)	1.08329	0.84115	0.889 (0.009)
					**Average** 0.889 (0.009) [d]
With PhTXn$^+$					
1	187.857 (2.158)	201.461 (2.588)	1.11598	1.04573	0.874 (0.015)
2	193.851 (3.190)	206.298 (1.611)	1.11598	1.04573	0.881 (0.016)
3	188.365 (2.158)	220.215 (3.273)	1.0936	1.13395	0.887 (0.014)

4	190.550 (1.511)	164.258 (2.550)	1.08329	0.84115	0.901 (0.016)
					**Average**
					0.885 (0.015) [d]

[a] At 25 °C, every rate constant is from 6 measurements, same solutions of DMPBIH isotopologues (~ 1.2 x 10^{-3} M) are used to react with $PhXn^+$ (4.00 x 10^{-5} M) and $PhTXn^+$ (1.219 x 10^{-4} M) in order to have a direct comparison of the 2° KIEs for both reactions, numbers in parentheses are standard deviations (SDs); [b] measurement times for the reactions of both acceptors should be the same (four times), but one 2° KIE (=0.848) for the reaction of $PhXn^+$ is an outlier; [c] calculated using eqn (3) of the main paper; numbers in parentheses are standard deviations (SDs), $SD = KIE\cdot((SD(CH_3)/k(CH_3))^2 + (SD(CD_3)/k(CD_3)^2)^{1/2}$; [d] numbers in parentheses are pooled standard deviations S(pooled).

Synthesis of 9-(4-*substituted*phenyl)-10-methylacridinium tetrafluoroborate (*G*$PhMA^+BF_4^-$)

General procedure

1.01 mmol 10-methyl-9-(4-*substituted*phenyl)-9,10-dihydroacridine was dissolved in 30 mL of HPLC grade acetonitrile, with gentle heating if needed. 0.988 mmol of tropylium tetrafluoroborate was added to the solution and stirred to dissolve. The flask was sealed with a septum and placed in a 60 °C hot water bath overnight. The solution was then gravity filtered, and the excess solvent was rotary evaporated to a volume of about 10 mL. Dry diethyl ether was added to precipitate the salt product. The product was recrystallized twice using acetonitrile/anhydrous diethyl ether. The yields of the products are from 60 – 85%.

9-phenyl-10-methylacridinium tetrafluoroborate ($PhMA^+$): ^1H NMR (400 MHz, CD_3CN) δ (ppm) 8.50-8.75 (d, 2H), 8.35-8.45 (m, 2H), 7.84-7.86 (d, 2H), 7.74-7.75 (m, 2H), 7.72-7.73 (m, 3H), 7.74-7.76 (d, 2H), 4.83 (s, 3H)

9-(4-(trifluoromethyl)phenyl)-10-methylacridinium tetrafluoroborate (CF_3PhMA^+): ^1H NMR (400 MHz, CD_3CN) δ (ppm) 8.64-8.66 (d, 2H), δ 8.30-8.40 (m, 2H), 7.86-7.88 (m, 2H), 7.84-7.86 (m, 2H), 7.81-7.83 (m, 2H), 7.69-7.71 (d, 2H), 4.85 ppm (s, 3H)

9-(4-bromophenyl)-10-methylacridinium tetrafluoroborate ($BrPhMA^+$): ^1H NMR (400 MHz, CD_3CN) δ (ppm) 8.50-8.52 (m, 2H), 8.41-8.43 (t, 2H), 7.87-7.89 (m, 2H), 7.84-7.86 (m, 2H), 7.82-7.84 (m, 2H), 7.42-7.44 (m, 2H), 4.83 ppm (s, 3H)

9-(p-tolyl)-10-methylacridinium tetrafluoroborate (CH_3PhMA^+): ^1H NMR (400 MHz, CD_3CN) δ(ppm) 8.59-8.65 (d, 2H), 8.40-8.45 (m, 2H), 8.04-8.06 (d, 2H), 7.82-7.86 (t, 2H), 7.56-7.58 (d, 2H), 7.40-7.42 (d, 2H), 4.81 (s, 3H), 2.56 (s, 3H)

9-(4-methoxyphenyl)-10-methylacridinium tetrafluoroborate (CH_3OPhMA^+): ^1H NMR (400 MHz, CD_3CN) δ (ppm) 8.60-8.70 (d, 2H), 8.34-8.36 (m, 2H), 8.10-8.13 (d, 2H), 7.84-7.87 (m, 2H), 7.81-7.83 (m, 2H), 7. 46-7.57 (d, 2H), 7.27-7.29 (d, 2H), 4.80 (s, 3H), 3.96 (s, 3H)

9-(4-(N,N-dimethyl)phenyl)-10-methylacridinium tetrafluoroborate ($(CH_3)_2NPhMA^+$): ^1H NMR (400 MHz, CD_3CN) δ (ppm) 8.51-8.52 (d, 2H), 8.33-8.35 (m, 2H), 8.26-8.28 (d, 2H), 7.81-7.83 (m, 2H), 7.43-7.45 (d, 2H), 7. 03-7.05 (d, 2H), 4.74 (s, 3H), 3.13 (s, 6H)

Synthesis of 9-(4-*substituted*phenyl)-10-methyl-9,10-dihydroacridine (*G*PhMAH)

General procedure

*G*PhMAH was synthesized by reacting 10-methylacridinium iodide with Grignard reagent made from 4-substitutedbromobenzene in dry THF following a procedure described in literature[4].

9-phenyl-10-methyl-9,10-dihydroacridine (PhMAH): M.p. 105-109 ºC; ^{1}H NMR (400 MHz, CD_3CN) δ (ppm) 7.11-7.15 (m, 4H), 7.09-7.10 (m, 3H), 7.05-7.08(m, 2H), 6.95-7.03 (m, 2H), 5.26 (s, 1H), 3.40 (s, 3H)

9-(4-trifluoromethylphenyl)-10-methyl-9,10-dihydroacridine (CF_3PhMAH): M.p. 125-128 °C; and ^{1}H NMR (400 MHz, $CDCl_3$) δ (ppm) 7.42-7.44 (d, 2H), 7.22-7.24 (m, 2H), 7.18-7.20 (m, 4H), 6.93-6.99 (m, 4H), 5.25 (s, 1H), 3.42 (s, 3H); and ^{19}F NMR (400 MHz, $CDCl_3$) δ (ppm) -62.40

9-(4-bromophenyl)- 10-methyl-9,10-dihydroacridine (BrPhMAH): M.p., 137-139 °C; ^{1}H NMR (400 MHz, CDCl3), δ (ppm) 7.30-7.31 (d, 2H), 7.17-7.18 (m, 2H), 7.15-7.16 (m, 2H), 6.93-6.96 (m, 6H), 5.14 (s, 1H), 3.40 (s, 3H)

9-(p-tolyl)-10-methyl-9,10-dihydroacridine (CH_3PhMAH): M.p., 140-142 °C; ^{1}H NMR (400 MHz, $CDCl_3$) δ (ppm) 7.16-7.24 (m, 4H), 6.90 (s, 4H), 6.70-6.73 (m, 4H), 5.14 (s, 1H), 3.40 (s, 3H), 2.25 (s, 3H)

9-(4-methoxyphenyl)-10-methyl-9,10-dihydroacridine (CH_3OPhMAH): M.p., 141-143 °C; ^{1}H NMR (400 MHz, $CDCl_3$) δ (ppm) 7.18-7.22 (m, 2H), 7.15-7.17 (m, 2H), 7.01-7.03 (m, 2H), 6.91-6.96 (m, 4H), 6.74-6.76 (m, 2H), 5.12 (s, 1H), 3.73 (s, 3H), 3.41 (s, 3H)

9-(4-(N,N-dimethyl)phenyl)-10-methyl-9,10-dihydroacridine ($(CH_3)_2N$PhMAH): M.p., 140-142 °C; ^{1}H NMR (400 MHz, $CDCl_3$) δ (ppm) 7.15-7.20 (m, 4H), 6.89-7.00 (m, 6H), 6.60-6.62 (m, 2H), 5.07 (s, 1H), 3.40 (s, 3H), 2.86 (s, 6H)

Synthesis of 5-*substituted*-1,3-dimethyl-2-phenyl-benzimidozoline (*G*DMPBIH)

Commercially available 5-*substituted*-2-phenylbenzimidozole was reacted with CH_3I in methanol in a sealed pressure reactor at 120 °C to obtain the *G*DMPBI^{+}I^{-} salt. *G*DMPBIH was synthesized by reducing *G*DMPBI^{+} with sodium borohydride in methanol. Both syntheses followed the procedures described in literature.[5]

5-trifluromethyl-1,3-dimethyl-2-phenylbenzimidozoline (CF_3DMPBIH): M.p., 85.0-87.0 °C; NMR (400 MHz, $CDCl_3$) δ (ppm) 7.52-7.54 (m, 2H), 7.45-7.47 (m, 3H), 6.95-6.97 (d, 1H), 6.57 (s, 1H), 6.40-6.42 (d, 1H), 5.26 (s, 1H), 2.56 (s, 6H)

5-methoxy-1,3-dimethyl-2-phenylbenzimidozoline (MeODMPBIH): M.p., 94.0-96.0 °C; NMR (400 MHz, $CDCl_3$) δ (ppm) 7.54-7.55 (m, 2H), 7.43-7.45 (m, 3H), 6.32-6.34 (d, 1H), 6.17-6.20 (d, 1H), 6.12-6.13 (d, 1H), 4.75 (s, 1H), 3.70 (s, 3H), 2.46-2.49 (d, 6H)

Reference

1. Maness, P.; Koirala, S.; Adhikari, P.; Salimraftar, N.; Lu, Y., Substituent Effects on Temperature Dependence of Kinetic Isotope Effects in Hydride-Transfer Reactions of NADH/NAD+ Analogues in Solution: Reaction Center Rigidity Is the Key. *Org. Lett.* **2020,** *22* (15), 5963–5967.
2. Zhu, X. Q.; Deng, F. H.; Yang, J. D.; Li, X. T.; Chen, Q.; Lei, N. P.; Meng, F. K.; Zhao, X. P.; Han, S. H.; Hao, E. J.; Mu, Y. Y., A classical but new kinetic equation for hydride transfer reactions. *Org. Biomol. Chem.* **2013,** *11*, 6071-6089.
3. Shen, G. B.; Xia, K.; Li, X. T.; Li, J. L.; Fu, Y. H.; Yuan, L.; Zhu, X. Q., Prediction of Kinetic Isotope Effects for Various Hydride Transfer Reactions Using a New Kinetic Model. *J. Phys. Chem. A* **2016,** *120*, 1779−1799.
4. Fukuzumi , S.; Tokuda, Y.; Kitano, T.; Okamoto, T.; Otera, J., Electron-transfer oxidation of 9-substituted 10-methyl-9,10-dihydroacridines. Cleavage of the carbon-hydrogen vs. carbon-carbon bond of the radical cations. *J. Am. Chem. Soc.* **1993,** *115*, 8960-8968.
5. Zhu, X., -Q.; Zhang, M.-T.; Yu, A.; Wang, C.-H.; Cheng, J.-P., Hydride, Hydrogen Atom, Proton, and Electron Transfer Driving Forces of Various Five-Membered Heterocyclic Organic Hydrides and Their Reaction Intermediates in Acetonitrile. *J. Am. Chem. Soc.* **2008,** *130* (8), 2501–2516.

Data Availability Statement

In the main paper, we have described ways to determine the rate constants. Here we provide more details about how the data is collected and fitted to derive the pseudo-first order rate constants (k^{pfo}). We will also present the primary kinetic data for all of the rate constants reported in the paper (listed in Tables S1 to S11 and Table S13). For the latter, we directly copy the original data from the corresponding excel data file. Due to the decimal point place difference in between the two places, data may slightly differ at the last digit of their numbers. Meanwhile, we provide the *Abs* – time (t) data for the measurements of k^{pfo}.

All of the kinetics were determined on the SF-61DX2 Hi-Tech KinetAsyst double-mixing stopped-flow instrument. At first, we estimate a rate constant according to our experience. We then collect *Abs* - t data around 15 half-lives to derive an estimated k^{pfo}. This may need a few attempts depending upon how accurate our estimate is. According to the k^{pfo} value, we calculate the half-life time, and run the formal kinetic measurements for about 15 half-lives (or more than 12 half-lives) for six times with one minute time interval between runs to allow equilibration of temperature of the reaction solutions. Using the software in the computer interfaced to the instrument, we fit the 12 half-lives (99.98% completion of the reaction) *Abs* - t data. For the Table S9 results only, we collected and fitted 3 half-lives data to derive the rate constants, but we also carried out a single run of more than 12 half-lives (“long time run (LR)”) to make sure that the reaction is complete and there is no absorption at the wavelength of measurements after 12 half-lives of the reaction. If a small spike of the absorbance change is observed at the very beginning due to mixing of solutions, the initial data selected for the fit would exclude that part of the data. This initial data point is usually close to 1% of the reactions. Figure S6 shows one example as to how the fit looks like (R^2 value) and how the k^{pfo} is generated.

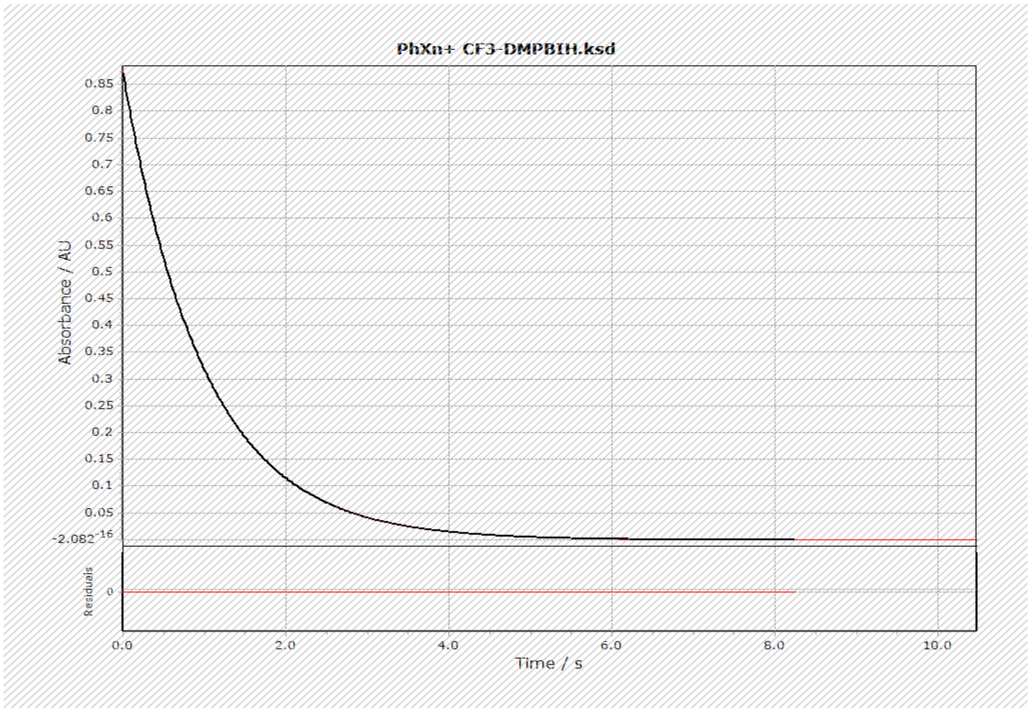

Figure S6. The fit to a kinetic run for the reaction between *CF3*DMPBIH (0.001219M) and $PhXn^+$ (0.00004M) in acetonitrile at 25°C at 373 nm due to $PhXn^+$ following the first-order kinetic law (Definition: Y = -A*exp(-R*X) + C; R is k^{pfo}). This is copied from the Kinetic Studio report generated from the fitting software in the instrument (k^{pfo} = 1.00909 (s^{-1}), linear regression coefficient R^2 = 0.99998).

Primary kinetic data for the rate constants in Table S1

Day 1 data (September 30, 2020)

Temp (°C)	Trial H1	Trial H2	Trial H3	Trial H4	Trial H5	Trial H6	Average k_H^{pfo} (s^{-1})	Stdev	k_{2H} ($M^{-1}s^{-1}$)	Stdev [a]
	Pseudo-first-order rate constants k_H^{pfo} (s^{-1})									
45	355.58	358.31	356.38	351.06	346.44	343.01	351.80	6.07	2.16E+05	3736.16
35	312.96	305.75	313.70	306.83	310.97	312.13	310.39	3.32	1.91E+05	2043.05
25	274.49	270.70	272.27	268.25	274.13	275.32	271.97	2.58	1.67E+05	1585.92
15	222.15	222.00	225.83	220.70	225.44	223.14	223.21	2.03	1.37E+05	1252.28
5	187.10	182.71	182.60	187.39	185.62	188.40	185.64	2.48	1.14E+05	1525.00

Temp (°C)	Trial D1	Trial D2	Trial D3	Trial D4	Trial D5	Trial D6	Average k_D^{pfo} (s^{-1})	Stdev	k_{2D} ($M^{-1}s^{-1}$)	Stdev [a]
45	114.51	117.20	115.19	117.58	116.40	117.41	116.38	1.27	7.16E+04	782.02
35	97.93	96.88	97.59	97.02	99.12	99.71	98.04	1.15	6.03E+04	705.29
25	80.21	80.69	80.11	80.79	81.65	81.05	80.75	0.57	4.97E+04	349.12
15	62.92	63.63	64.41	63.62	64.03	63.95	63.76	0.51	3.92E+04	311.05
5	51.67	51.17	50.81	51.74	52.06	52.07	51.57	0.56	3.17E+04	346.03

[a] = (Stdev(for k^{pfo})/k^{pfo})*k_{2H}

Six kinetic runs together with the averaged data	First-order kinetic fit of the averaged data as an example
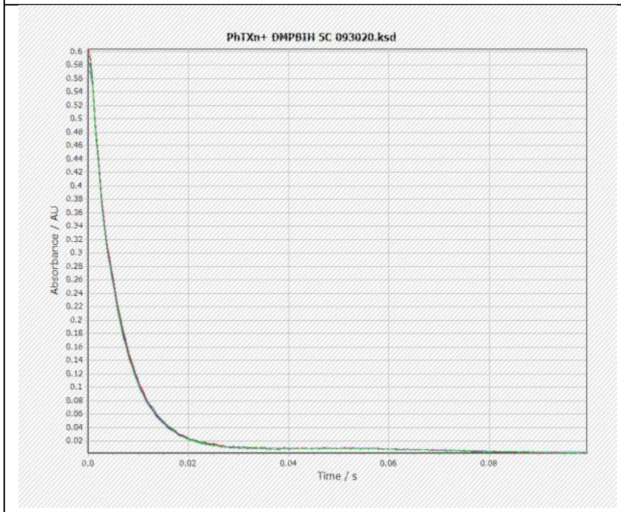	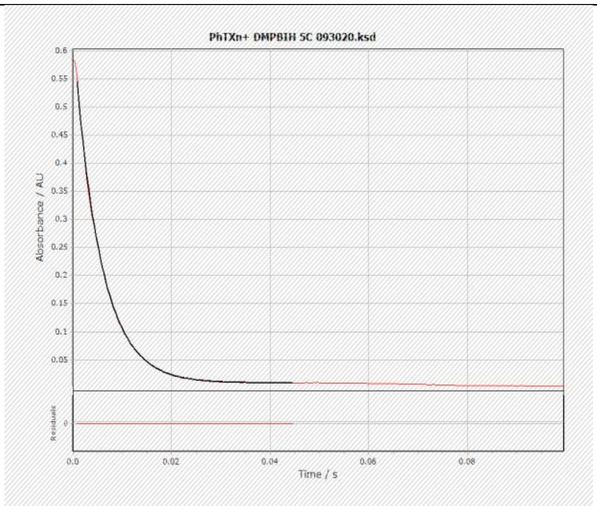
5℃ H	

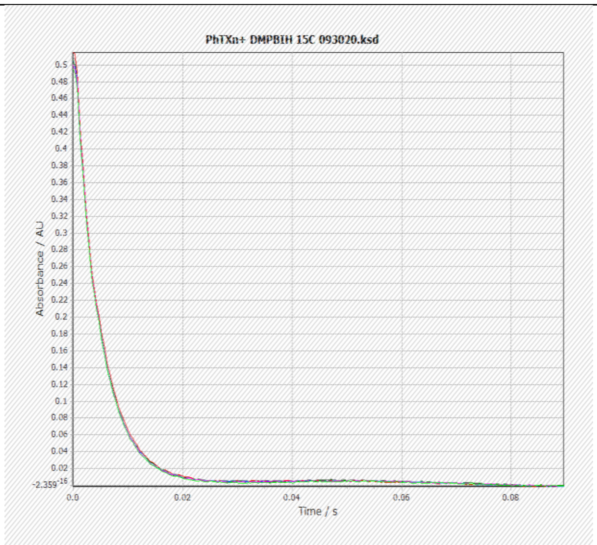
PhTXn+ DMPBIH 15C 093020.ksd
Absorbance / AU
Time / s

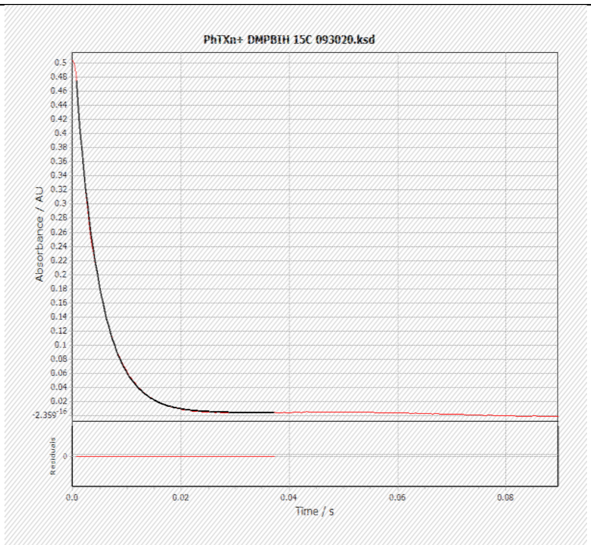
PhTXn+ DMPBIH 15C 093020.ksd
Absorbance / AU
Residuals
Time / s

15℃ H

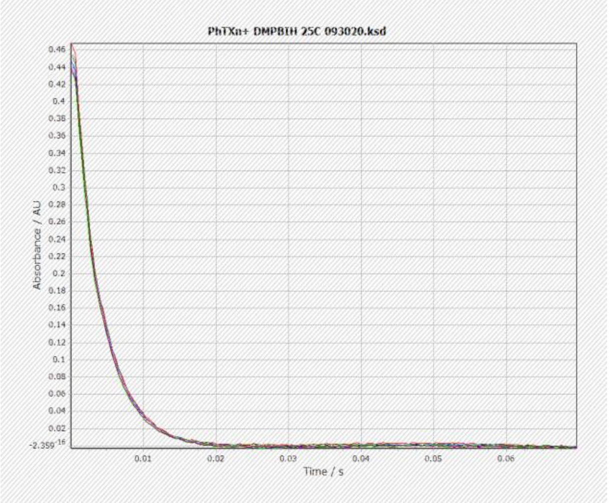
PhTXn+ DMPBIH 25C 093020.ksd
Absorbance / AU
Time / s

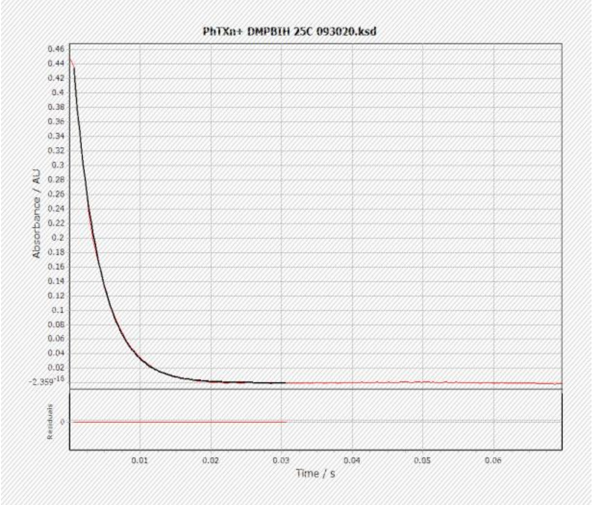
PhTXn+ DMPBIH 25C 093020.ksd
Absorbance / AU
Residuals
Time / s

25℃ H

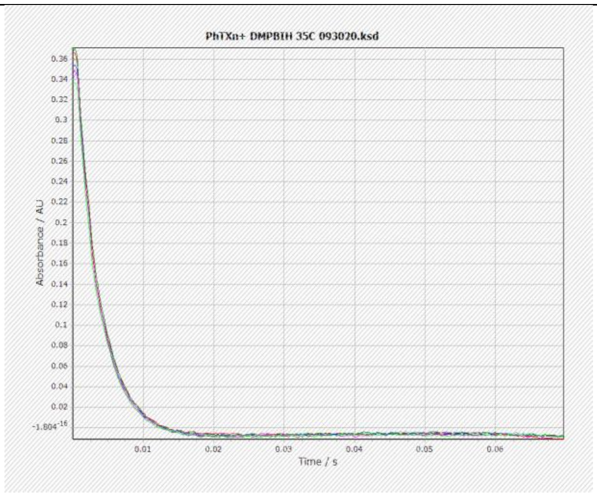
PhTXn+ DMPBIH 35C 093020.ksd
Absorbance / AU
Time / s

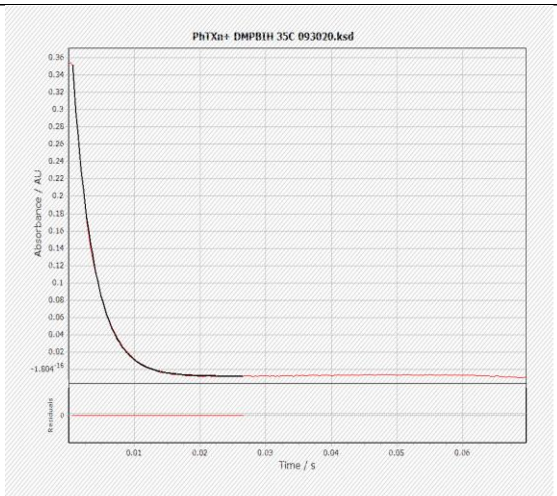
PhTXn+ DMPBIH 35C 093020.ksd
Absorbance / AU
Residuals
Time / s

35℃ H

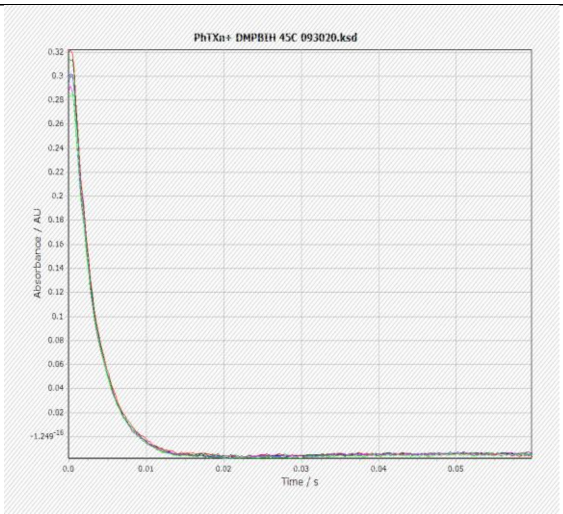
PhTXn+ DMPBIH 45C 093020.ksd
Absorbance / AU
Time / s

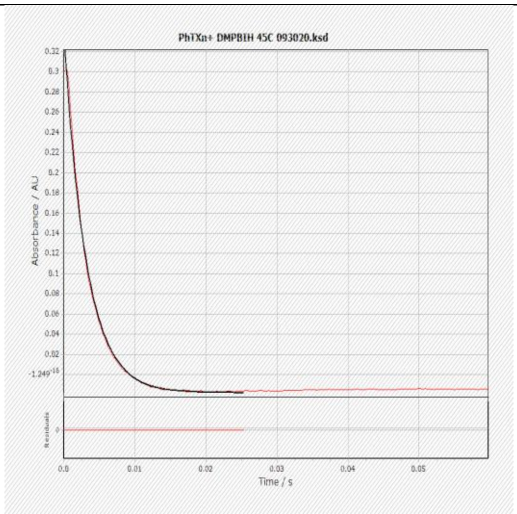
PhTXn+ DMPBIH 45C 093020.ksd
Absorbance / AU
Residuals
Time / s

45℃ H

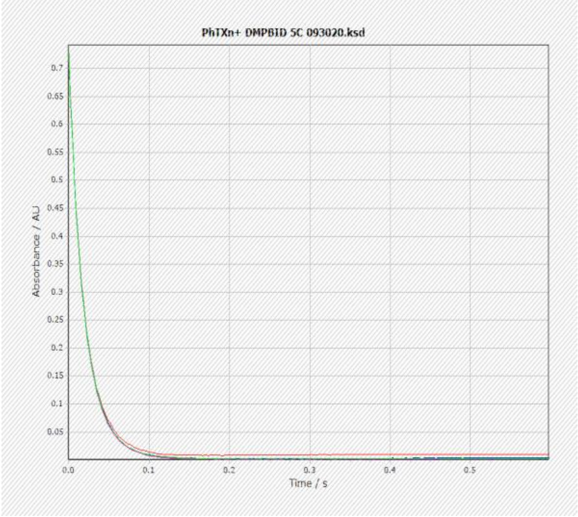
PhTXn+ DMPBID 5C 093020.ksd
Absorbance / AU
Time / s

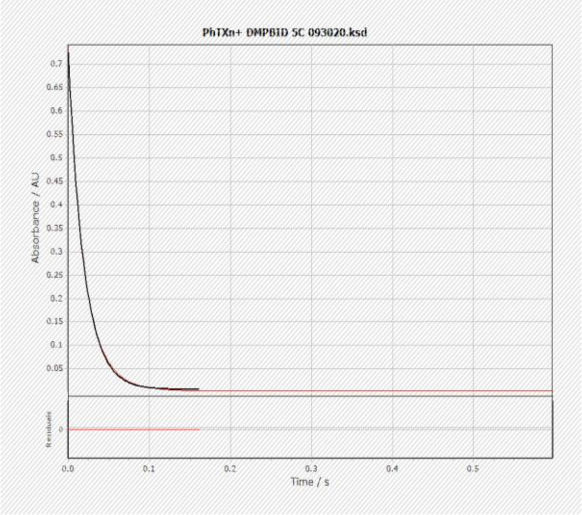
PhTXn+ DMPBID 5C 093020.ksd
Absorbance / AU
Residuals
Time / s

5℃ D

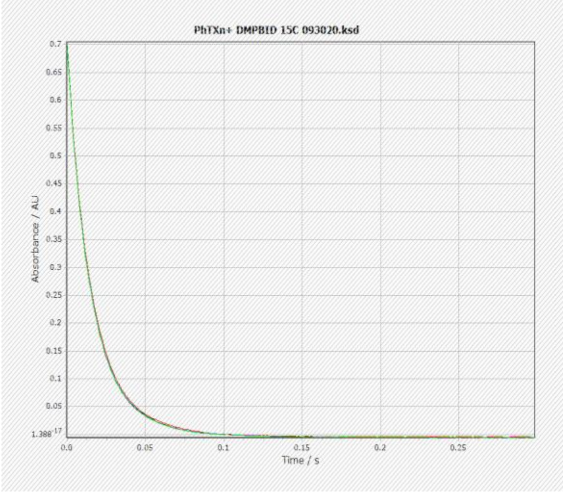
PhTXn+ DMPBID 15C 093020.ksd
Absorbance / AU
Time / s

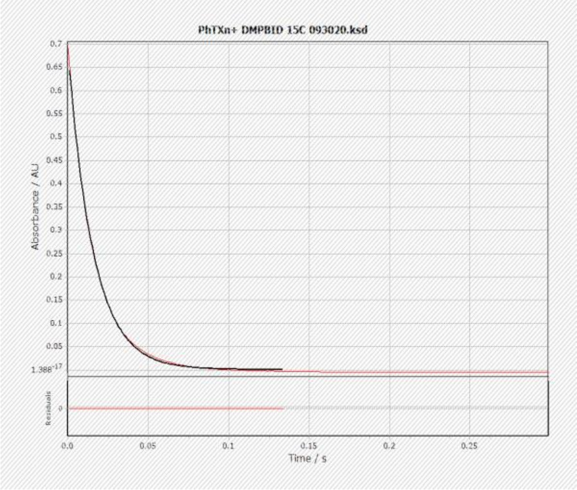
PhTXn+ DMPBID 15C 093020.ksd
Absorbance / AU
Residuals
Time / s

15℃ D

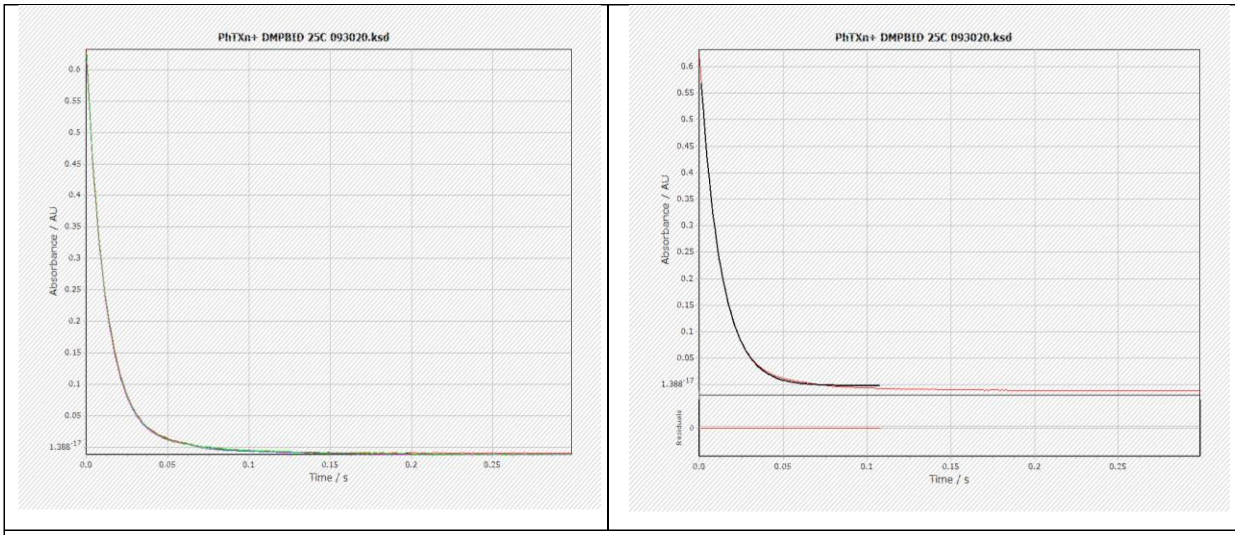
PhTXn+ DMPBID 25C 093020.ksd
Absorbance / AU
Time / s
PhTXn+ DMPBID 25C 093020.ksd
Absorbance / AU
Residuals
Time / s

25℃ D

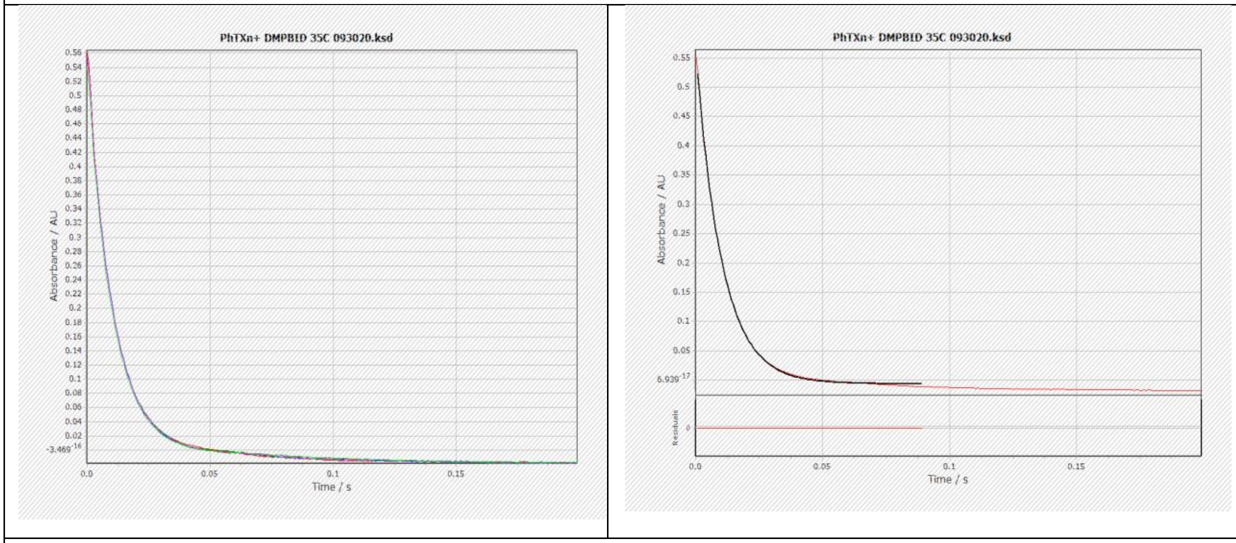
PhTXn+ DMPBID 35C 093020.ksd
Absorbance / AU
Time / s
PhTXn+ DMPBID 35C 093020.ksd
Absorbance / AU
Residuals
Time / s

35℃ D

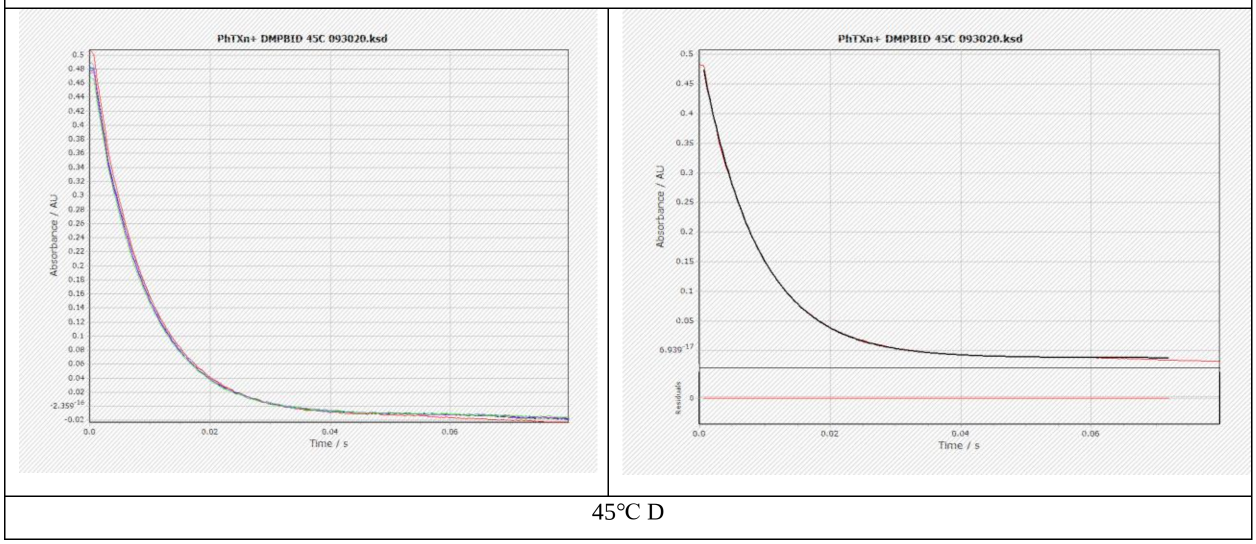
PhTXn+ DMPBID 45C 093020.ksd
Absorbance / AU
Time / s
PhTXn+ DMPBID 45C 093020.ksd
Absorbance / AU
Residuals
Time / s

45℃ D

Day 2 data (October 1, 2020)

	Pseudo-first-order rate constants k_H^{pfo} (s^{-1})									
**Temp (°C)**	**Trial H1**	**Trial H2**	**Trial H3**	**Trial H4**	**Trial H5**	**Trial H6**	**Average k_H^{pfo} (s^{-1})**	**Stdev**	**k_{2H} ($M^{-1}s^{-1}$)**	**Stdev [a]**
45	354.46	360.00	352.40	351.89	359.31	349.95	3.55E+02	4.13E+00	2.18E+05	2.54E+03
35	314.06	316.78	315.78	311.97	315.96	303.74	3.13E+02	4.87E+00	1.93E+05	3.00E+03
25	266.34	269.34	270.29	265.40	269.43	262.24	2.68E+02	2.15E+00	1.65E+05	1.32E+03
15	226.07	226.17	226.24	228.38	227.64	224.46	2.26E+02	1.37E+00	1.39E+05	8.41E+02
5	182.62	186.26	188.09	186.05	185.63	187.55	1.86E+02	1.92E+00	1.14E+05	1.18E+03

**Temp (°C)**	**Trial D1**	**Trial D2**	**Trial D3**	**Trial D4**	**Trial D5**	**Trial D6**	**Average k_D^{pfo} (s^{-1})**	**Stdev**	**k_{2D} ($M^{-1}s^{-1}$)**	**Stdev [a]**
45	115.31	117.76	116.29	117.34	118.08	117.71	1.17E+02	1.07E+00	7.21E+04	655.56
35	98.60	98.42	97.57	99.47	99.57	99.22	9.88E+01	7.62E-01	6.08E+04	469.21
25	79.46	81.13	81.00	80.66	81.31	82.68	8.10E+01	1.04E+00	4.99E+04	641.34
15	63.93	63.88	64.02	65.34	65.30	64.88	6.46E+01	6.92E-01	3.97E+04	425.86
5	51.32	51.24	52.04	51.48	51.53	52.61	5.18E+01	5.47E-01	3.19E+04	336.66

[a] = (Stdev(for k^{pfo})/k^{pfo})*k_{2H}

Six kinetic runs together with the averaged data	First-order kinetic fit of the averaged data as an example
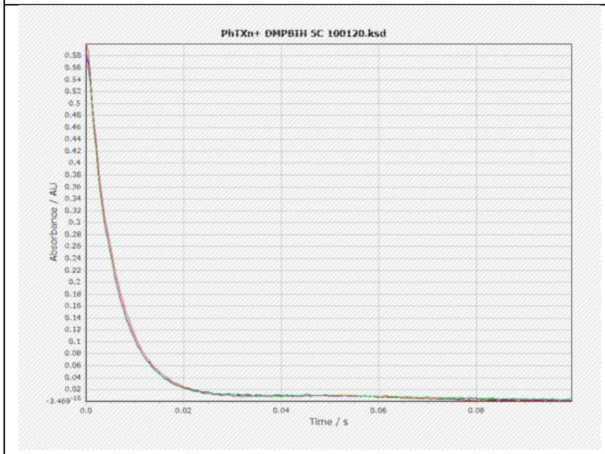	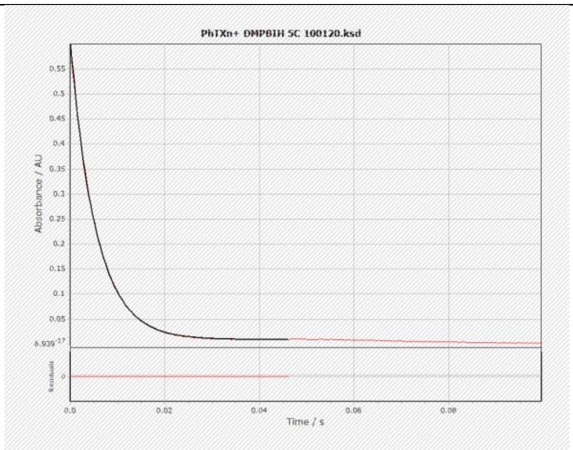

5°C H

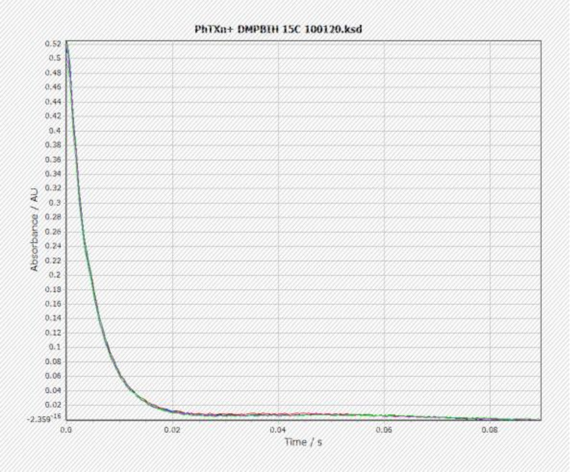

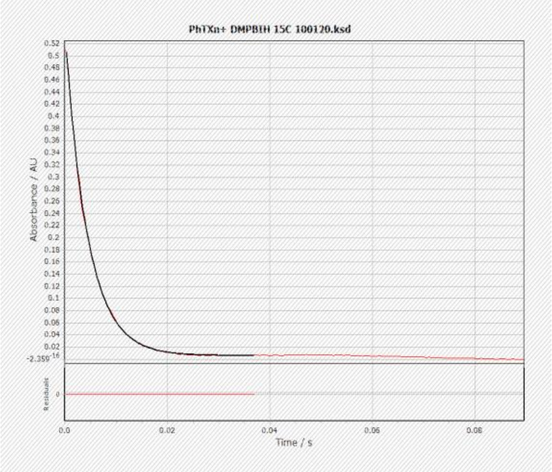

15°C H

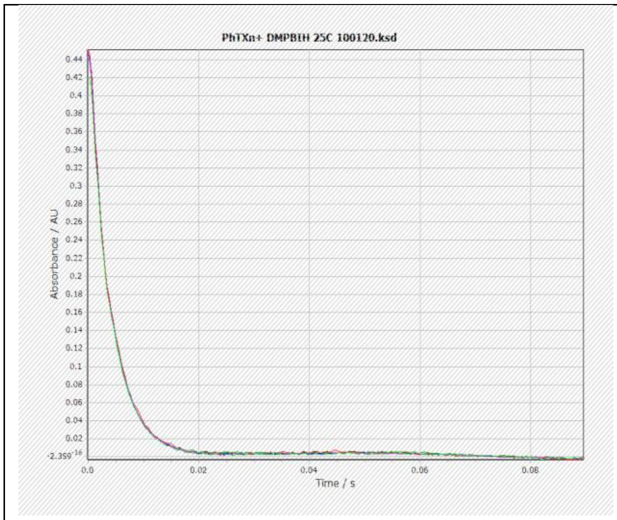

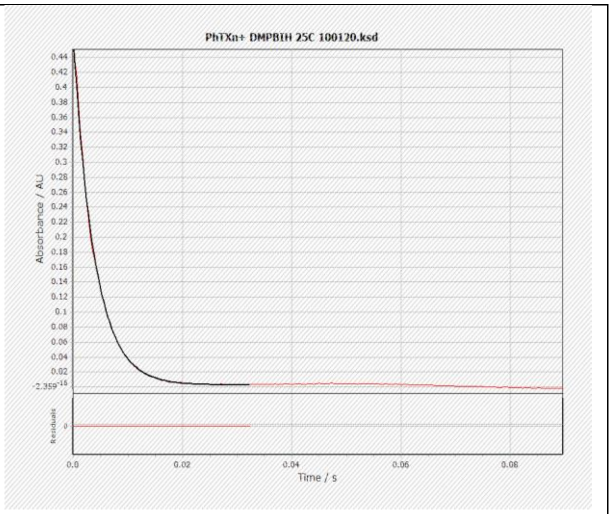

25°C H

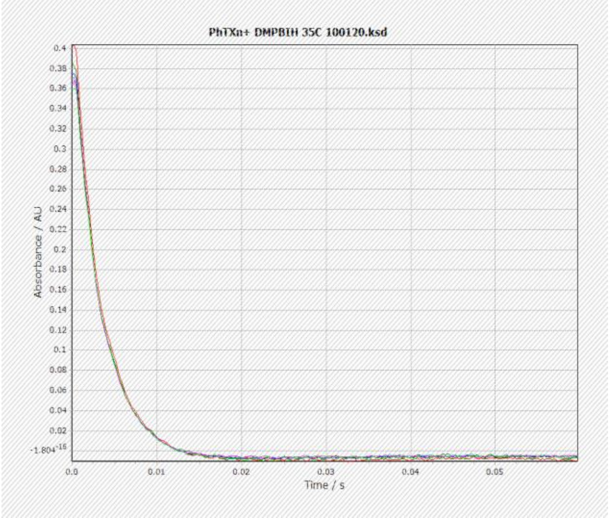

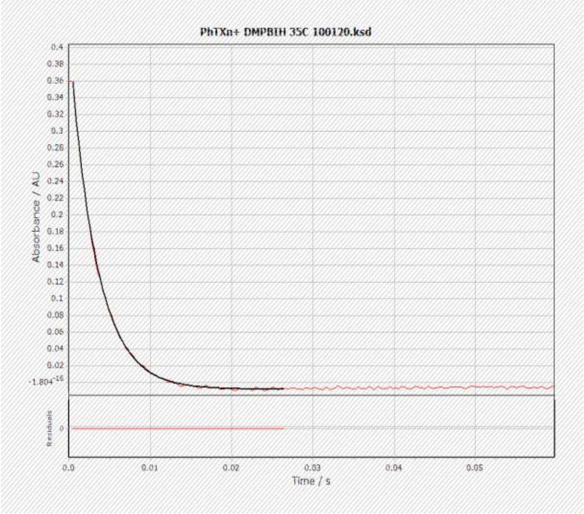

35°C H

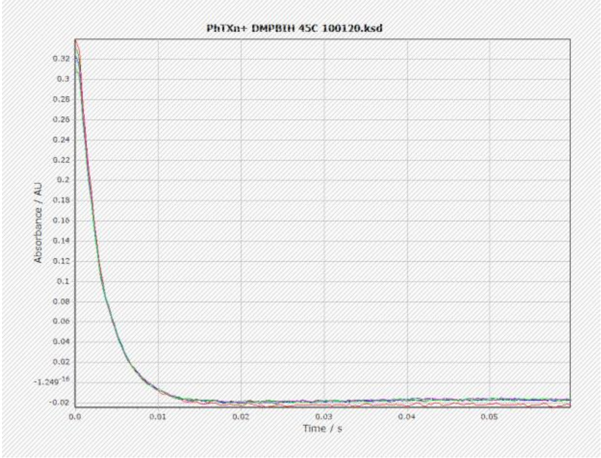

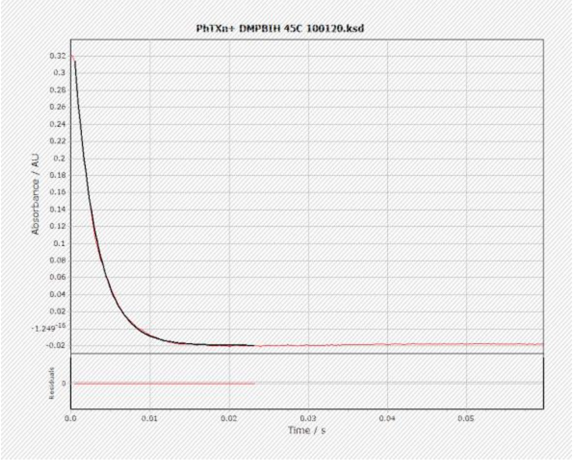

45°C H

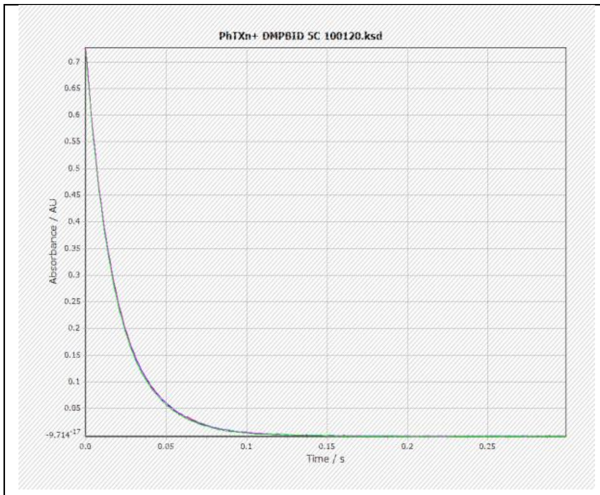

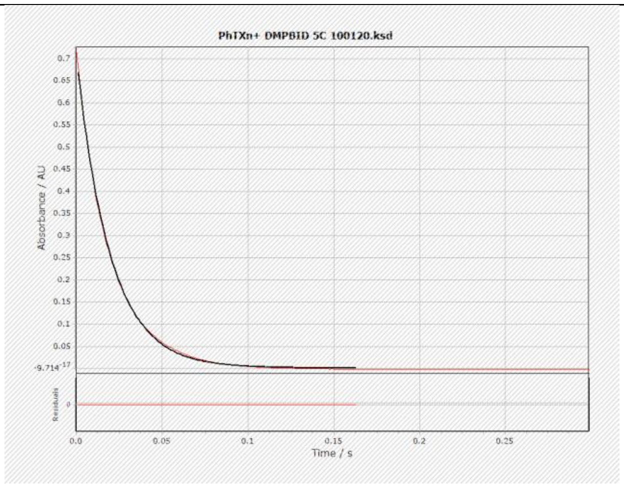

5°C D

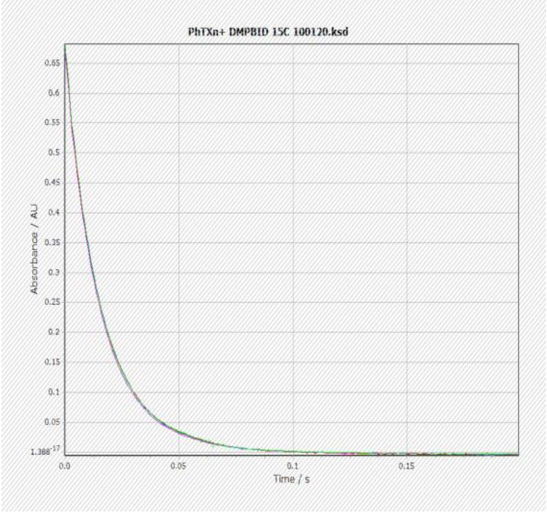

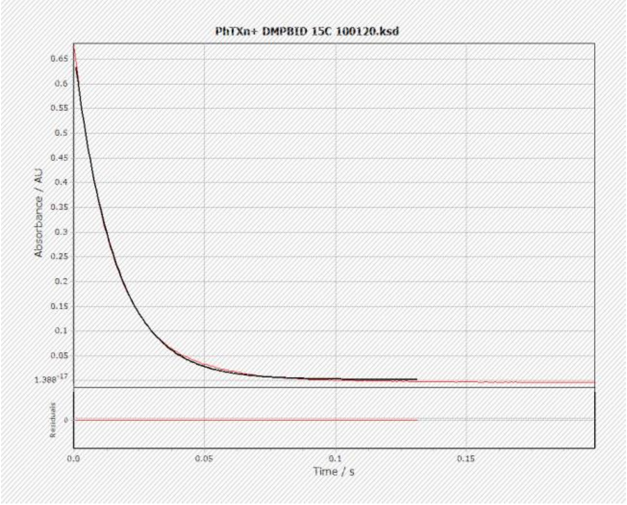

15°C D

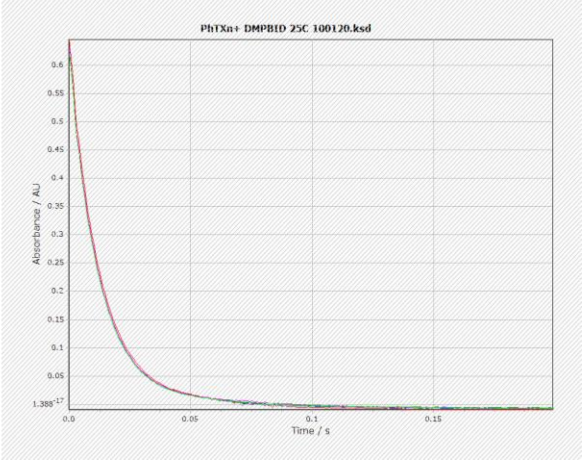

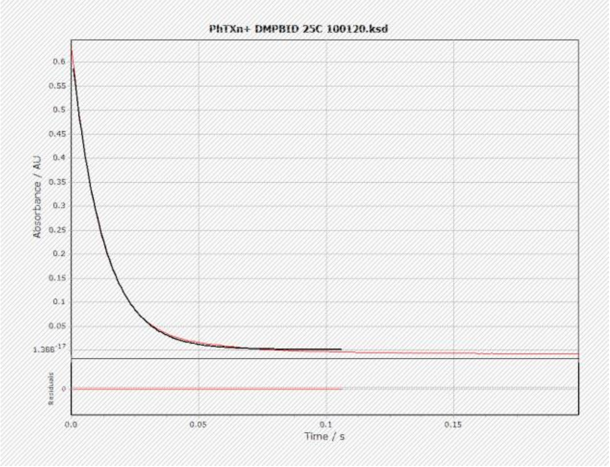

25°C D

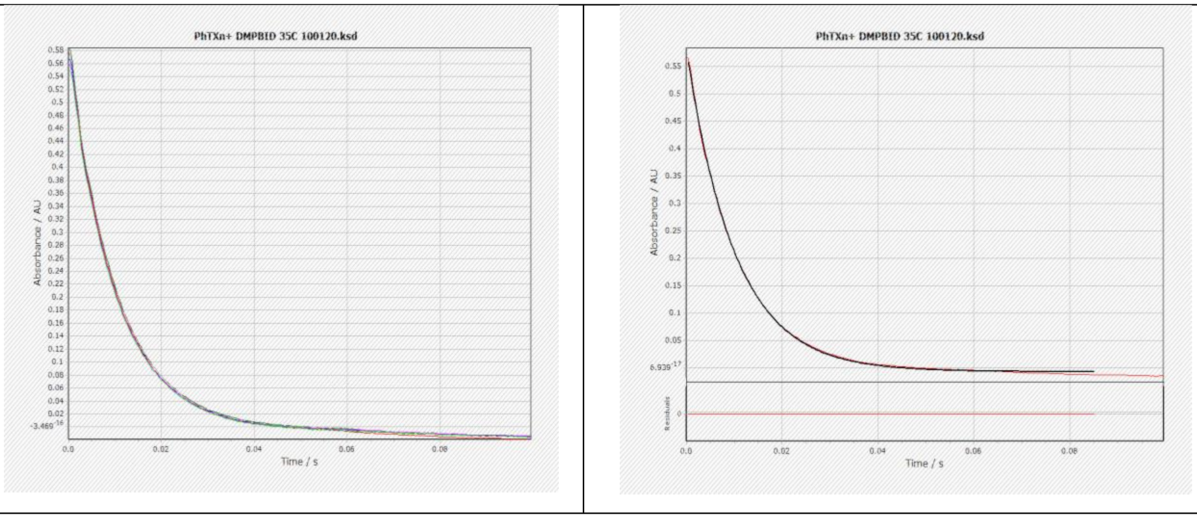

35°C D

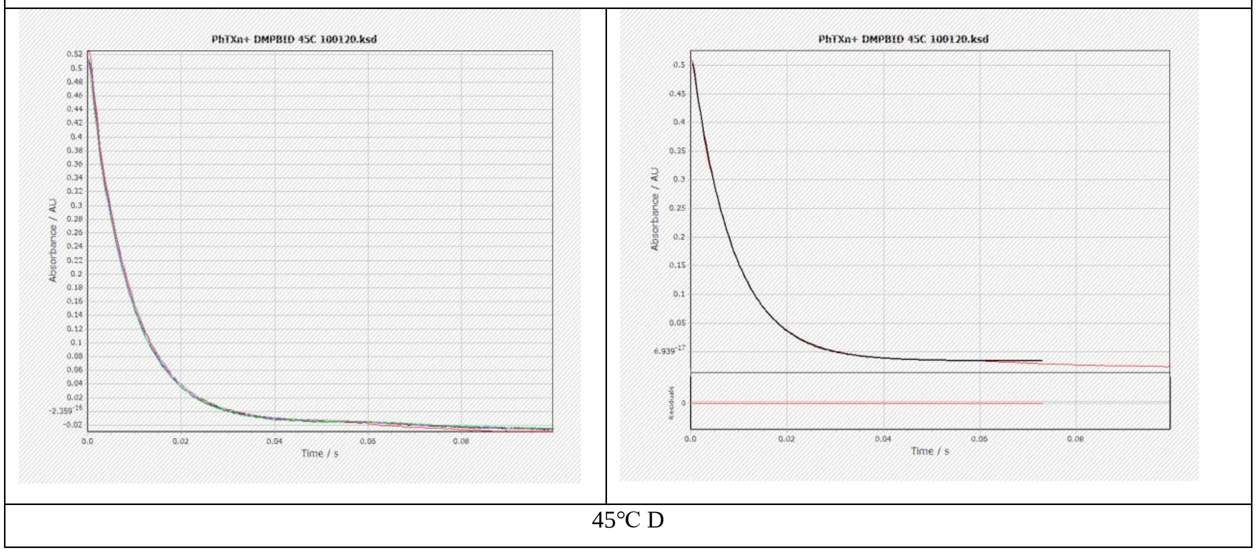

45°C D

Day 3 data (October 14, 2020)

	Pseudo-first-order rate constants k_H^{pfo} (s^{-1})									
**Temp (°C)**	**Trial H1**	**Trial H2**	**Trial H3**	**Trial H4**	**Trial H5**	**Trial H6**	**Average k_H^{pfo} (s^{-1})**	**Stdev**	**k_{2H} ($M^{-1}s^{-1}$)**	**Stdev [a]**
45	365.33	359.26	349.90	361.82	365.93	363.38	3.61E+02	5.93E+00	2.22E+05	3.65E+03
35	319.43	318.11	316.75	318.60	313.52	317.05	3.17E+02	2.07E+00	1.95E+05	1.28E+03
25	265.35	270.73	268.94	264.64	268.58	270.32	2.68E+02	2.57E+00	1.65E+05	1.58E+03
15	216.99	219.15	220.58	222.11	220.12	221.78	2.20E+02	1.88E+00	1.35E+05	1.16E+03
5	184.49	186.52	188.34	184.97	190.39	192.76	1.88E+02	3.23E+00	1.16E+05	1.99E+03
**Temp (°C)**	**Trial D1**	**Trial D2**	**Trial D3**	**Trial D4**	**Trial D5**	**Trial D6**	**Average k_D^{pfo} (s^{-1})**	**Stdev**	**k_{2D} ($M^{-1}s^{-1}$)**	**Stdev [a]**
45	117.14	116.01	117.69	117.38	116.91	117.96	1.17E+02	6.87E-01	7.21E+04	4.23E+02
35	96.93	97.37	96.77	99.61	98.52	97.88	9.78E+01	1.08E+00	6.02E+04	6.64E+02
25	79.81	79.95	80.79	81.27	80.77	81.78	8.07E+01	7.58E-01	4.97E+04	4.66E+02
15	60.93	61.96	60.95	62.38	62.69	61.83	6.18E+01	7.27E-01	3.80E+04	4.47E+02
5	50.24	50.67	50.67	51.12	50.32	51.12	5.08E+01	3.43E-01	3.12E+04	2.11E+02

[a] = (Stdev(for k^{pfo})/k^{pfo})*k_{2H}

<table>
<tr><td>Six kinetic runs together with the averaged data</td><td>First-order kinetic fit of the averaged data as an example</td></tr>
<tr><td>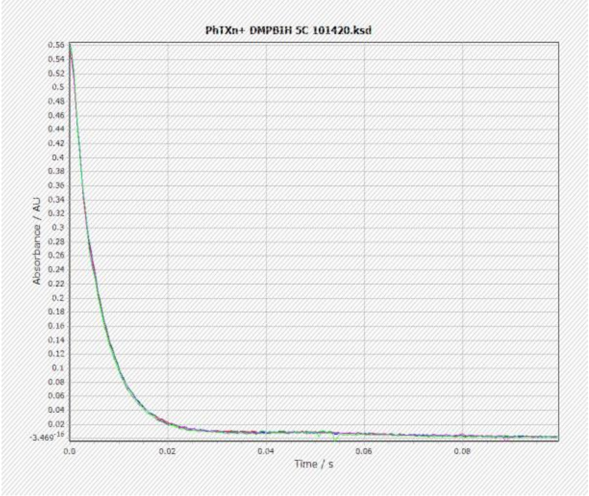
</td><td>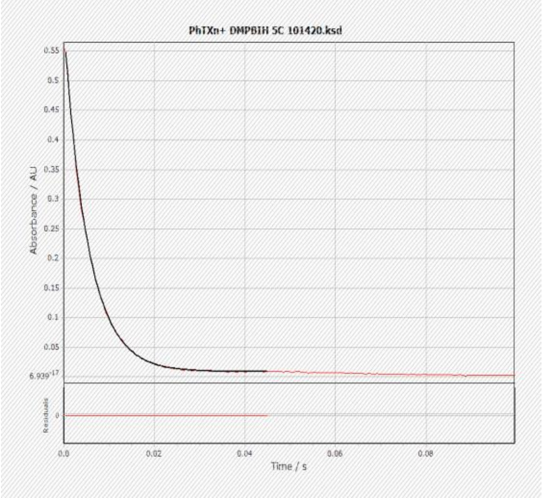
</td></tr>
<tr><td colspan="2">5℃ H</td></tr>
<tr><td>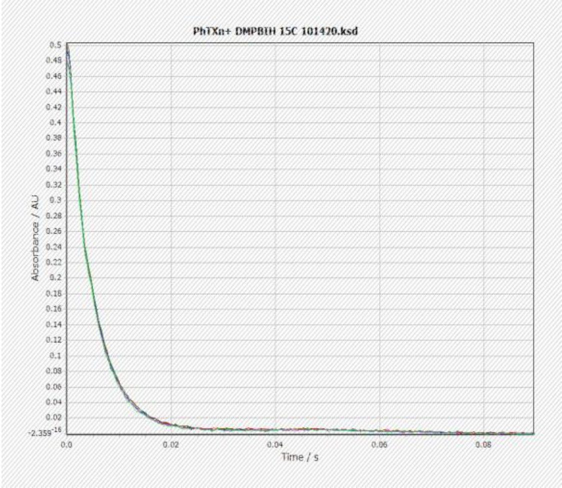
</td><td>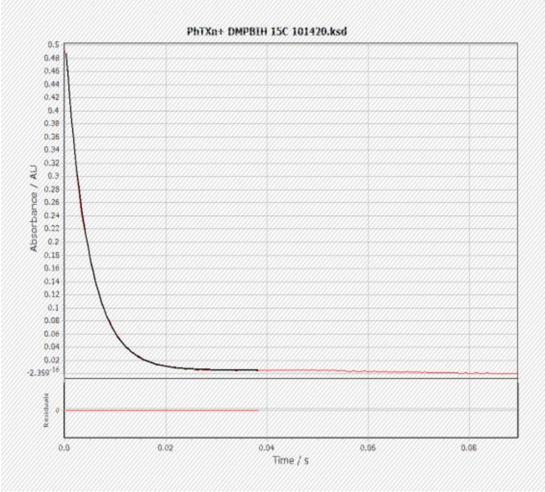
</td></tr>
<tr><td colspan="2">15℃ H</td></tr>
<tr><td>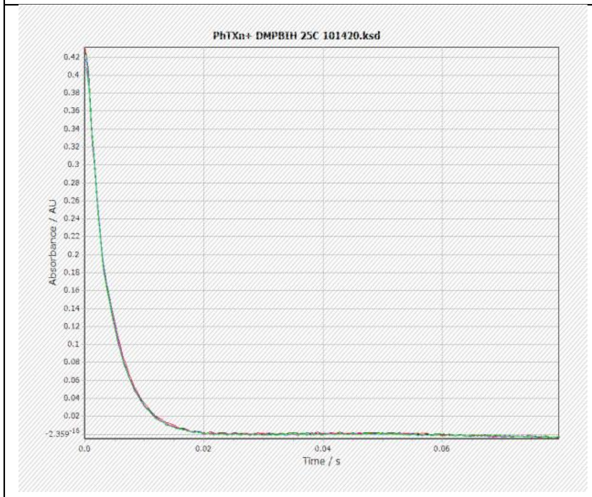
</td><td>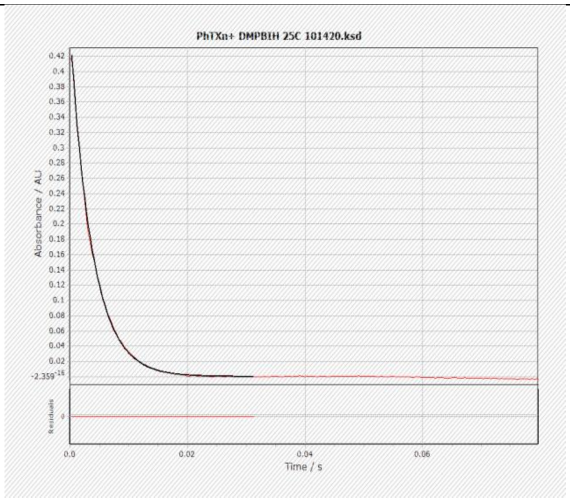
</td></tr>
<tr><td colspan="2">25℃ H</td></tr>
</table>

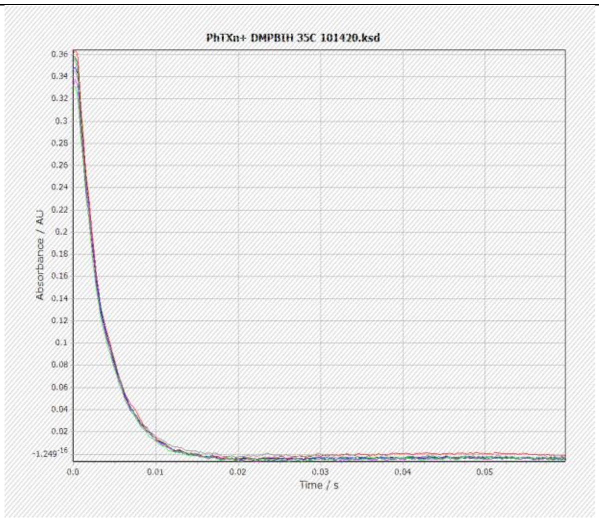

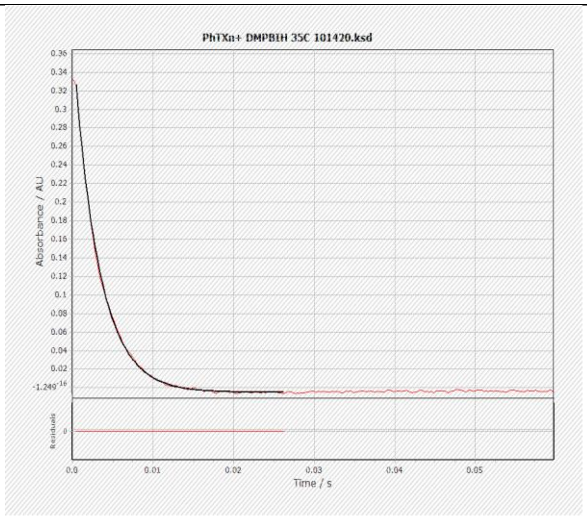

35°C H

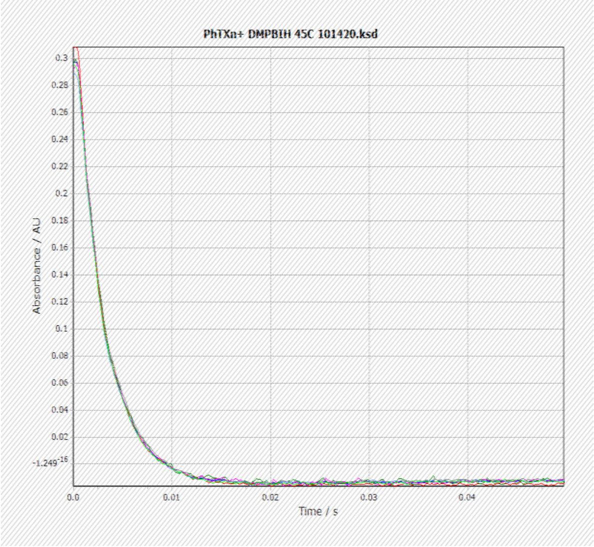

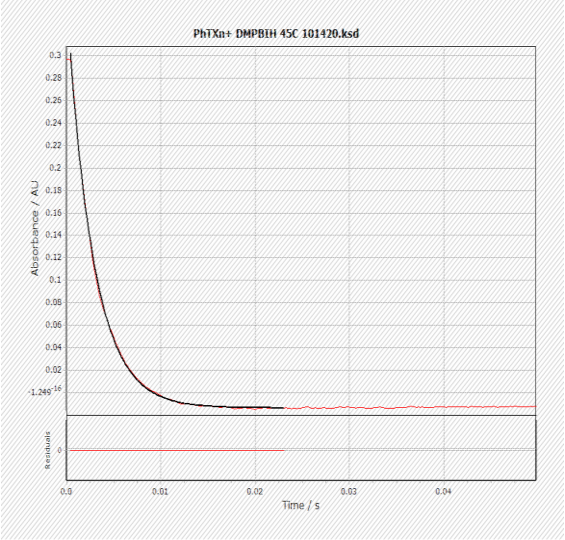

45°C H

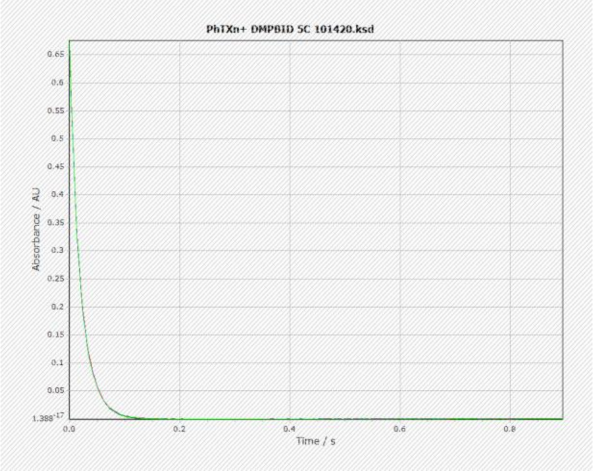

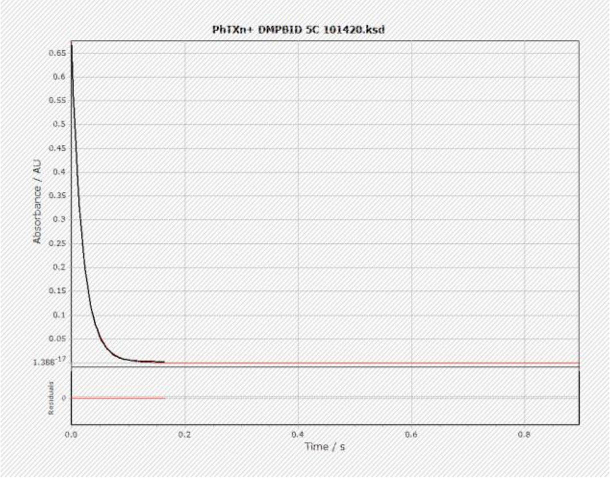

5°C D

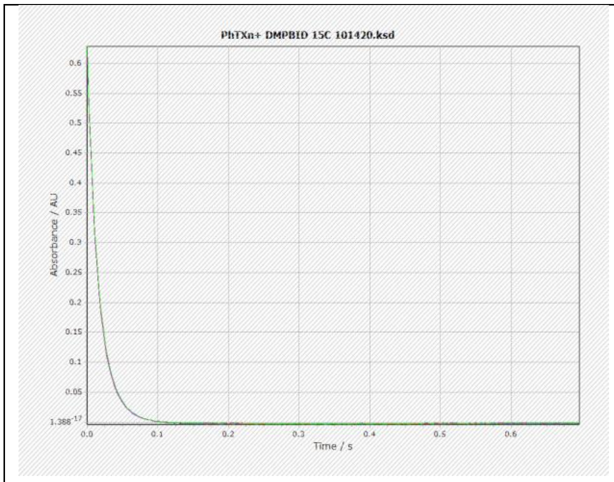
PhTXn+ DMPBID 15C 101420.ksd
Absorbance / AU
Time / s
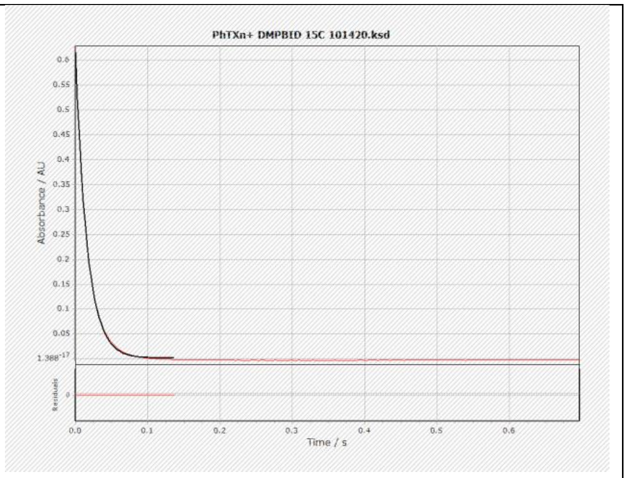
PhTXn+ DMPBID 15C 101420.ksd
Absorbance / AU
Residuals
Time / s

15℃ D

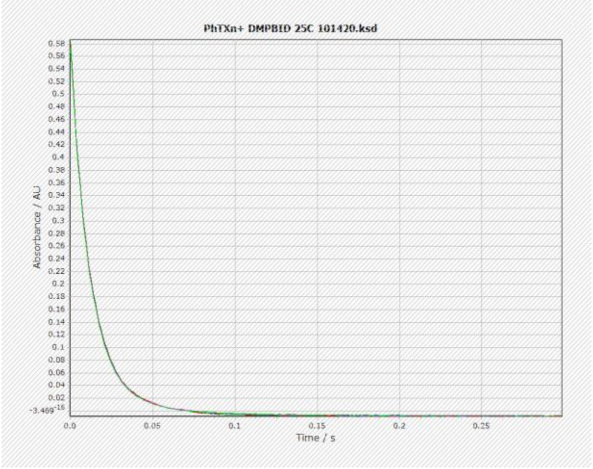
PhTXn+ DMPBID 25C 101420.ksd
Absorbance / AU
Time / s
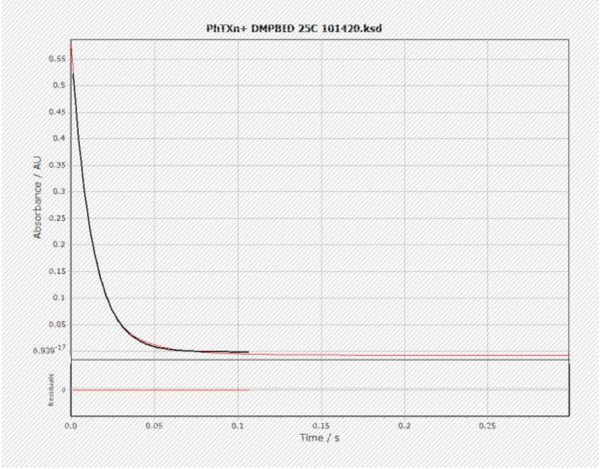
PhTXn+ DMPBID 25C 101420.ksd
Absorbance / AU
Residuals
Time / s

25℃ D

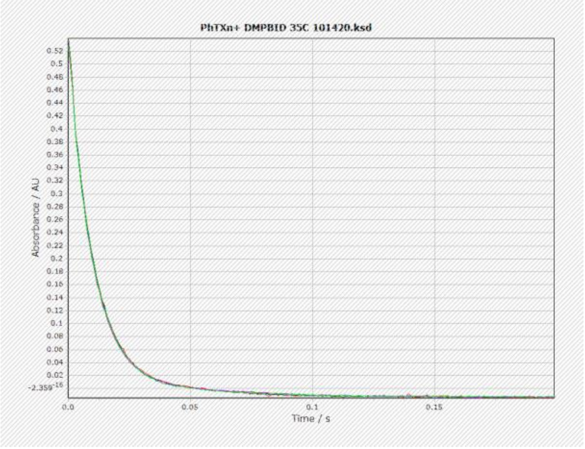
PhTXn+ DMPBID 35C 101420.ksd
Absorbance / AU
Time / s
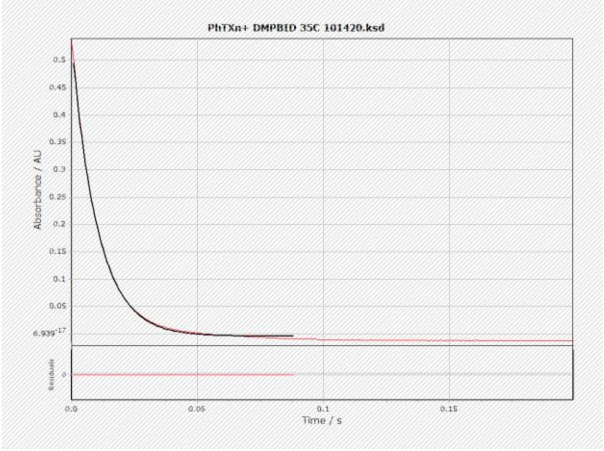
PhTXn+ DMPBID 35C 101420.ksd
Absorbance / AU
Residuals
Time / s

35℃ D

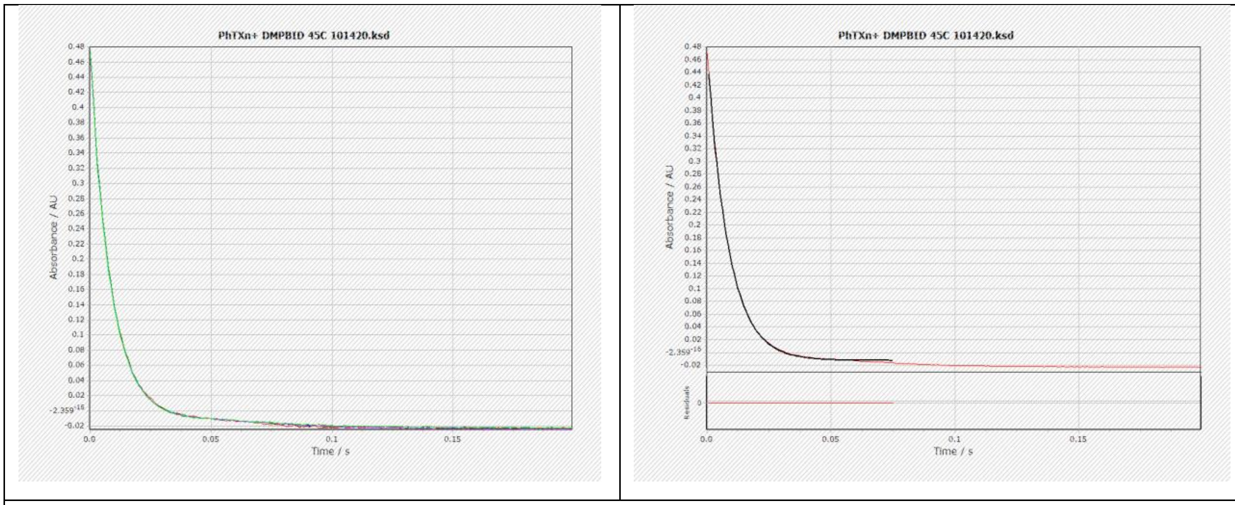

45°C D

Primary kinetic data for the rate constants in Table S2

Day 1 data (September 23, 2020)

	Pseudo-first-order rate constants k^{pfo} (s^{-1})									
**Temp (°C)**	**Trial H1**	**Trial H2**	**Trial H3**	**Trial H4**	**Trial H5**	**Trial H6**	**Average k_H^{pfo} (s^{-1})**	**Stdev**	**k_{2H} ($M^{-1}s^{-1}$)**	**Stdev [a]**
45	3.178	3.158	3.153	3.154	3.191	3.189	3.170	0.018	845.456	4.741
35	2.165	2.159	2.156	2.160	2.143	2.157	2.157	0.008	575.083	2.028
25	1.390	1.395	1.397	1.401	1.398	1.412	1.399	0.008	372.991	2.011
15	0.890	0.876	0.891	0.891	0.890	0.899	0.889	0.007	237.188	1.937
5	0.555	0.546	0.548	0.552	0.548	0.555	0.551	0.004	146.894	1.060
**Temp (°C)**	**Trial D1**	**Trial D2**	**Trial D3**	**Trial D4**	**Trial D5**	**Trial D6**	**Average k_D^{pfo} (s^{-1})**	**Stdev**	**k_{2D} ($M^{-1}s^{-1}$)**	**Stdev [a]**
45	0.709	0.714	0.718	0.708	0.718	0.718	0.714	0.005	190.444	1.248
35	0.457	0.461	0.460	0.462	0.464	0.465	0.462	0.003	123.081	0.755
25	0.287	0.288	0.288	0.291	0.293	0.292	0.290	0.002	77.318	0.597
15	0.171	0.174	0.176	0.175	0.175	0.176	0.175	0.002	46.534	0.502
5	0.096	0.095	0.095	0.095	0.095	0.094	0.095	0.001	25.347	0.159

[a] = (Stdev(for k^{pfo})/k^{pfo})*k_{2H}

<table>
<tr><th>Six kinetic runs together with the averaged data</th><th>First-order kinetic fit of the averaged data as an example</th></tr>
<tr><td>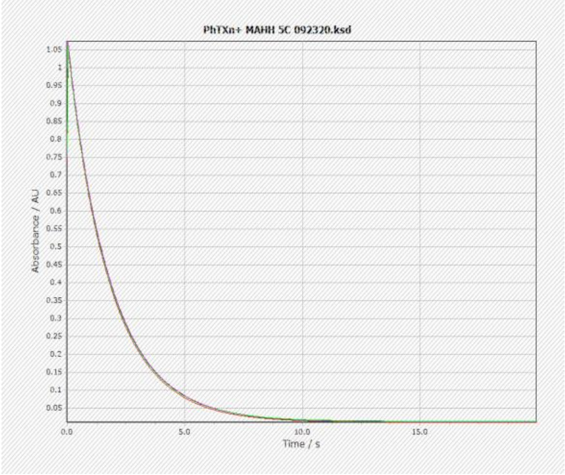
</td><td>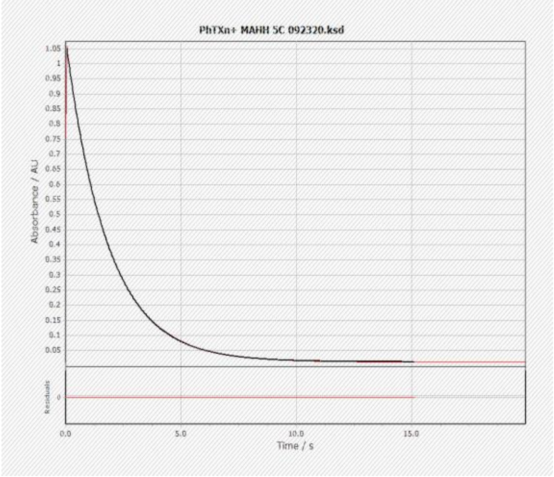
</td></tr>
<tr><td colspan="2">5℃ H</td></tr>
<tr><td>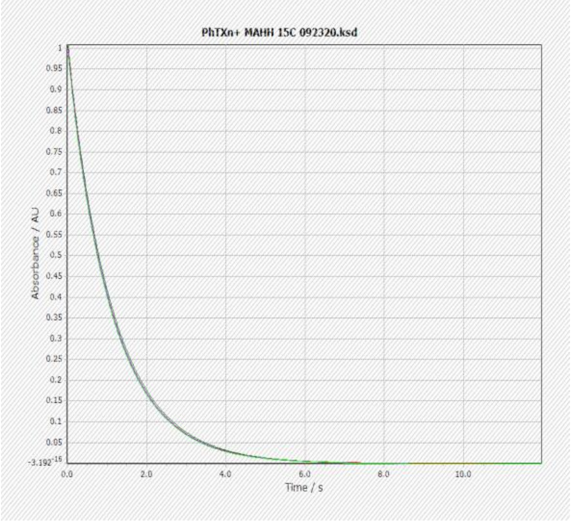
</td><td>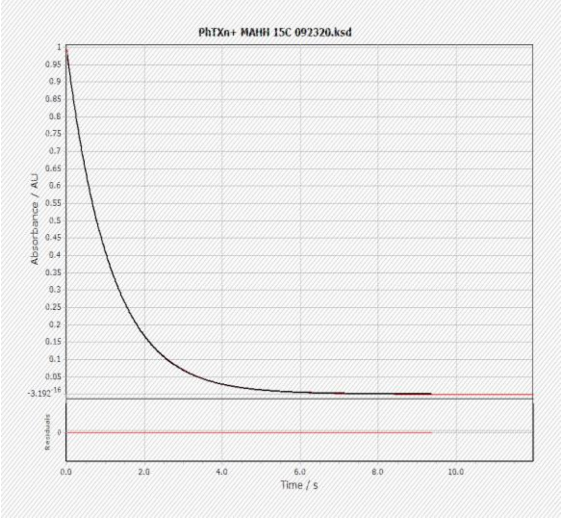
</td></tr>
<tr><td colspan="2">15℃ H</td></tr>
<tr><td>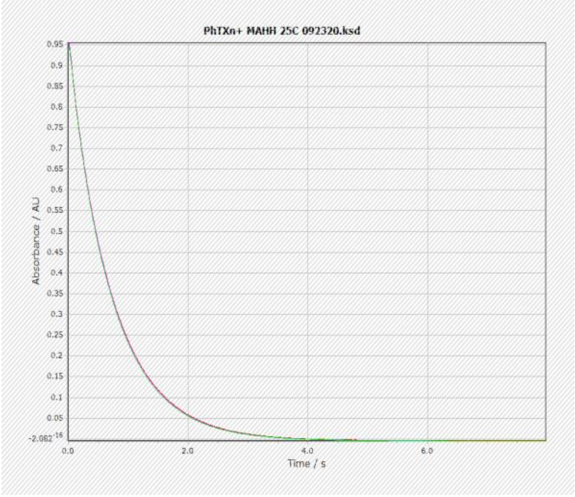
</td><td>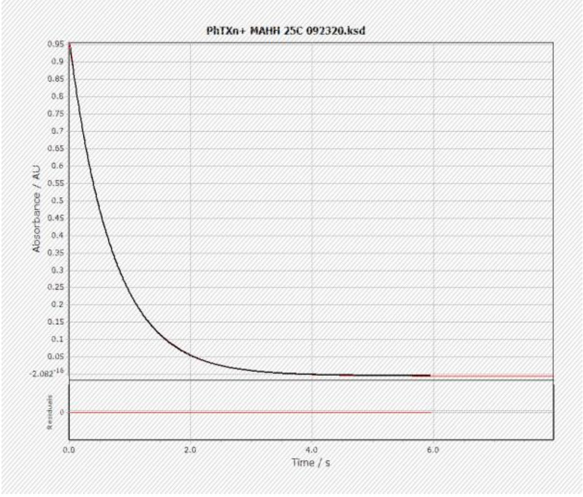
</td></tr>
<tr><td colspan="2">25℃ H</td></tr>
</table>

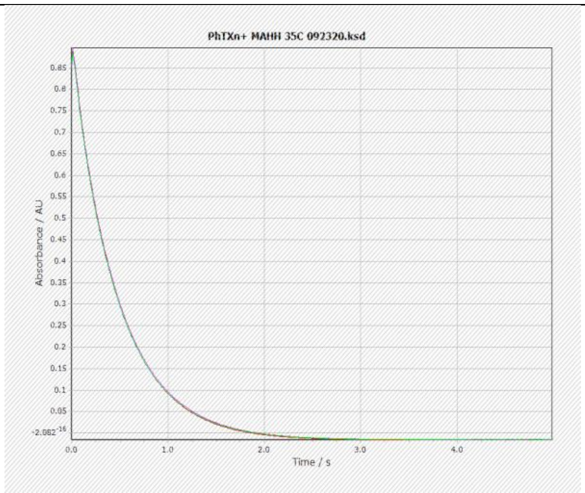 PhTXn+ MAHH 35C 092320.ksd<br>Absorbance / AU<br>Time / s	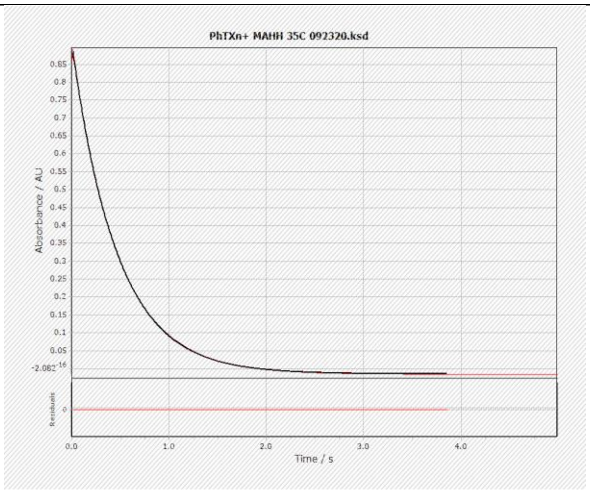 PhTXn+ MAHH 35C 092320.ksd<br>Absorbance / AU<br>Residuals<br>Time / s
35℃ H	
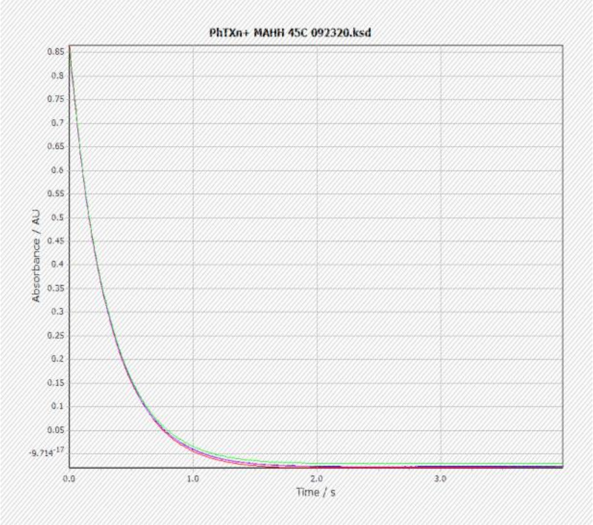 PhTXn+ MAHH 45C 092320.ksd<br>Absorbance / AU<br>Time / s	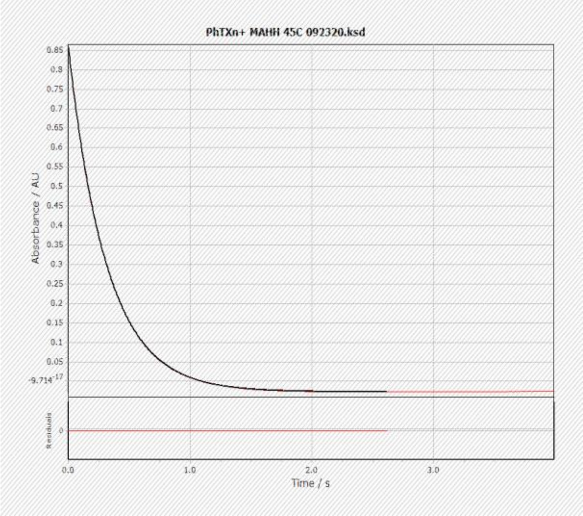 PhTXn+ MAHH 45C 092320.ksd<br>Absorbance / AU<br>Residuals<br>Time / s
45℃ H	
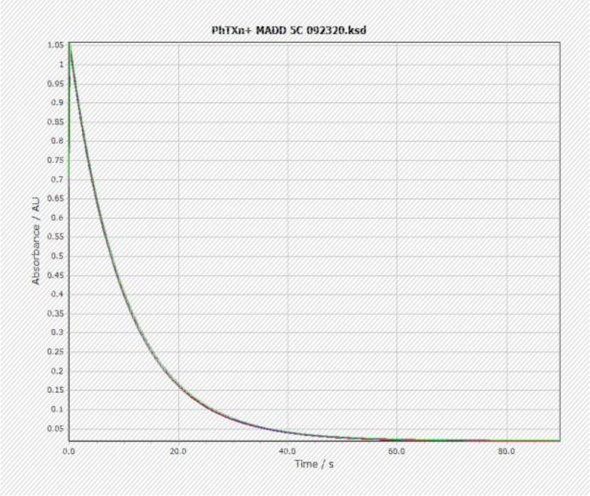 PhTXn+ MADD 5C 092320.ksd<br>Absorbance / AU<br>Time / s	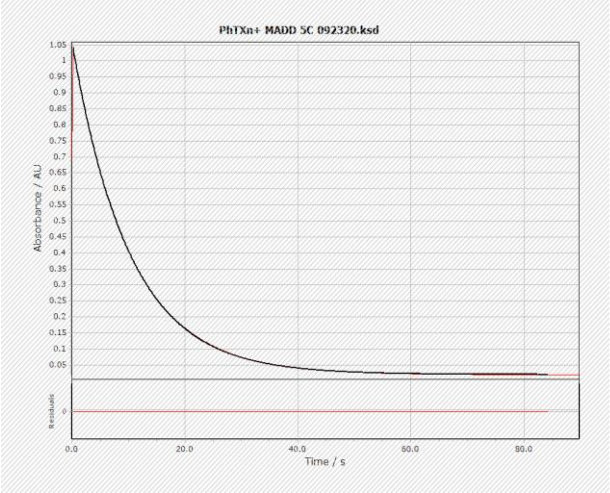 PhTXn+ MADD 5C 092320.ksd<br>Absorbance / AU<br>Residuals<br>Time / s
5℃ D	

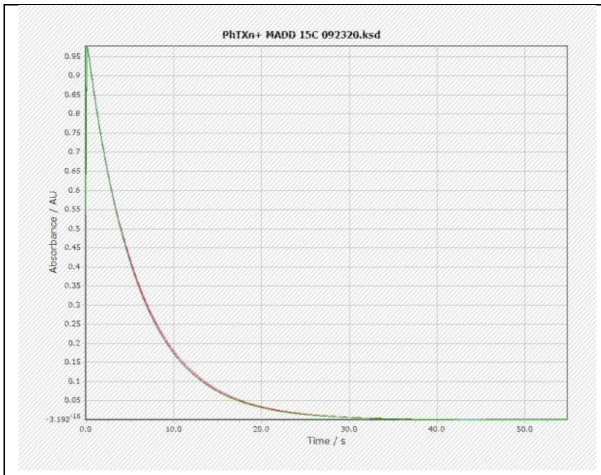

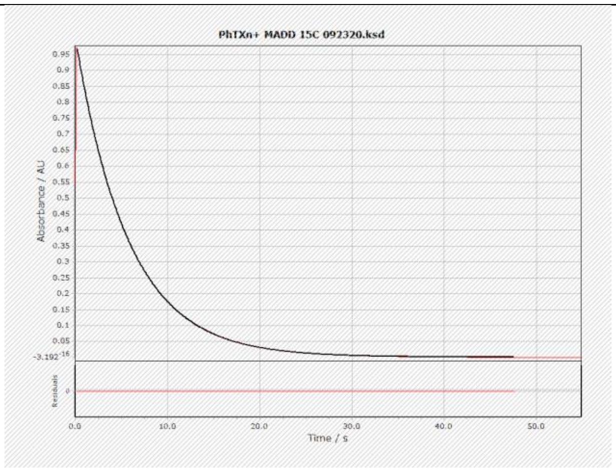

15°C D

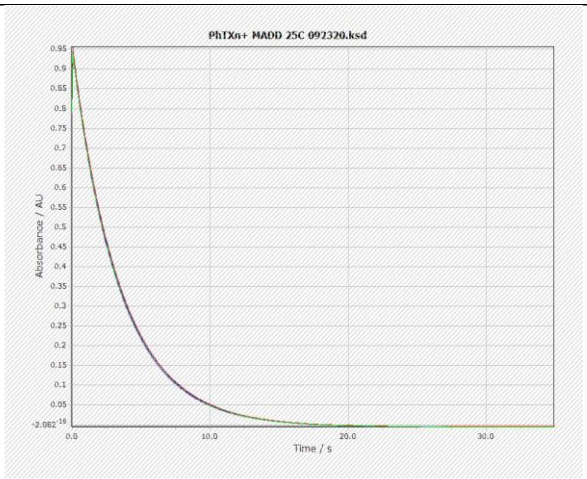

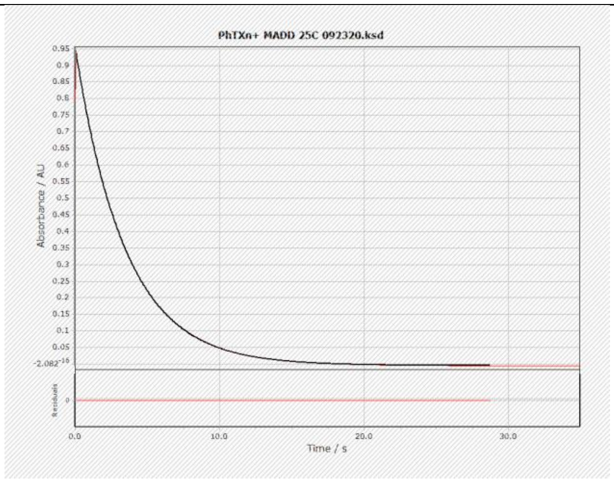

25°C D

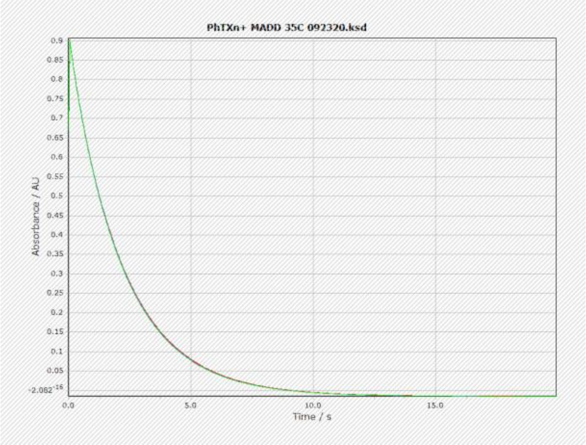

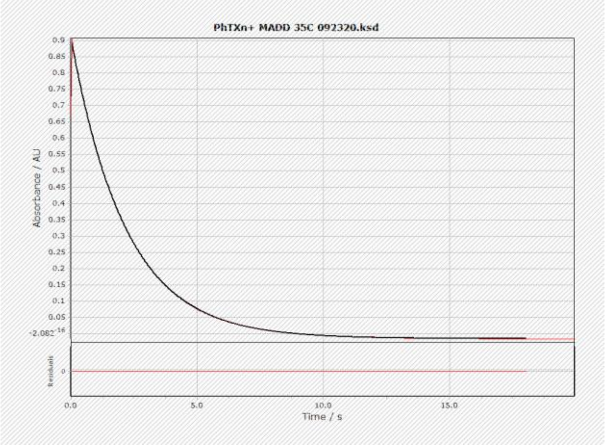

35°C D

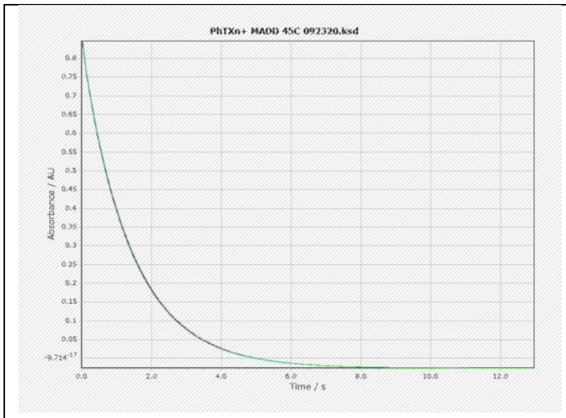

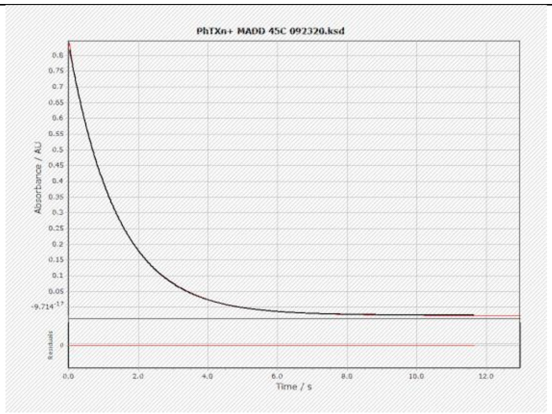

45℃ D

Day 2 data (October 7, 2020)

Temp (°C)	Trial H1	Trial H2	Trial H3	Trial H4	Trial H5	Trial H6	Average k_H^{pfo} (s^{-1})	Stdev	k_{2H} ($M^{-1}s^{-1}$)	Stdev [a]
	Pseudo-first-order rate constants k^{pfo} (s^{-1})									
45	3.127	3.153	3.158	3.158	3.178	3.157	3.155	0.017	841.338	4.408
35	2.141	2.142	2.157	2.140	2.159	2.155	2.149	0.009	573.063	2.348
25	1.360	1.359	1.369	1.357	1.387	1.372	1.367	0.011	364.656	3.015
15	0.890	0.871	0.884	0.873	0.883	0.881	0.880	0.007	234.774	1.881
5	0.519	0.517	0.521	0.528	0.525	0.537	0.525	0.007	139.868	1.968

Temp (°C)	Trial D1	Trial D2	Trial D3	Trial D4	Trial D5	Trial D6	Average k_D^{pfo} (s^{-1})	Stdev	k_{2D} ($M^{-1}s^{-1}$)	Stdev [a]
45	0.717	0.723	0.719	0.717	0.717	0.720	0.719	0.002	191.713	0.606
35	0.462	0.459	0.461	0.465	0.464	0.462	0.462	0.002	123.291	0.604
25	0.286	0.288	0.290	0.290	0.285	0.287	0.288	0.002	76.692	0.520
15	0.162	0.164	0.161	0.163	0.165	0.168	0.164	0.002	43.687	0.605
5	0.094	0.094	0.095	0.095	0.095	0.096	0.095	0.001	25.297	0.151

[a] = (Stdev(for k^{pfo})/k^{pfo})*k_{2H}

Six kinetic runs together with the averaged data

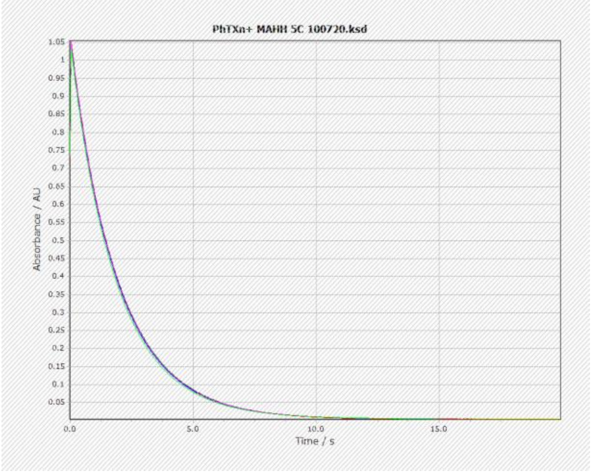

First-order kinetic fit of the averaged data as an example

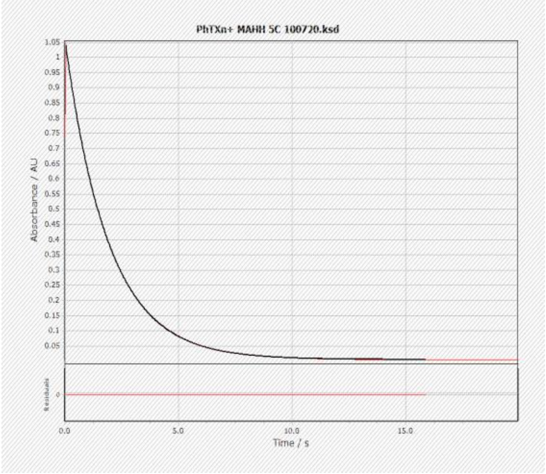

5℃ H

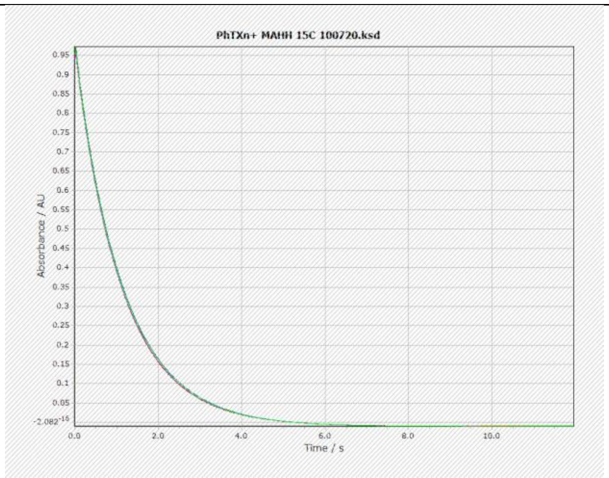

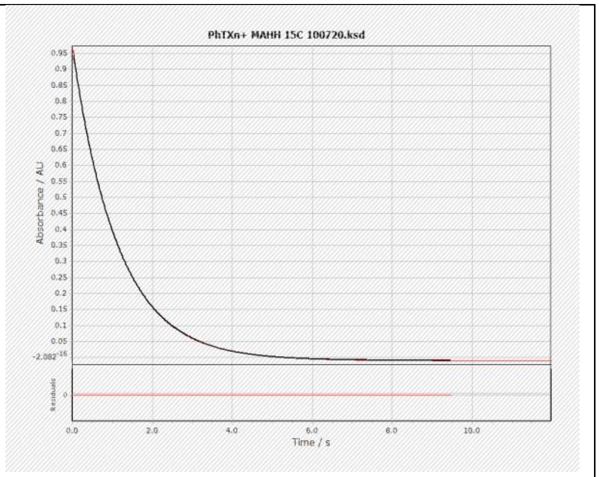

15°C H

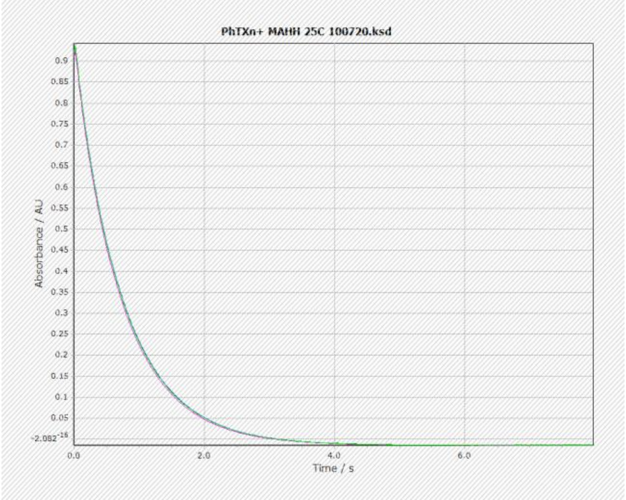

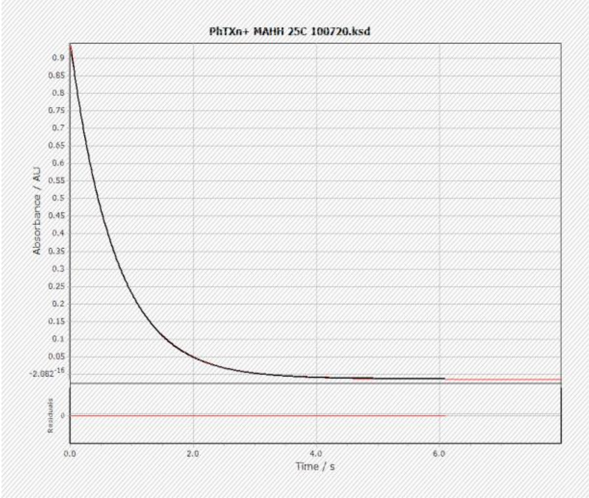

25°C H

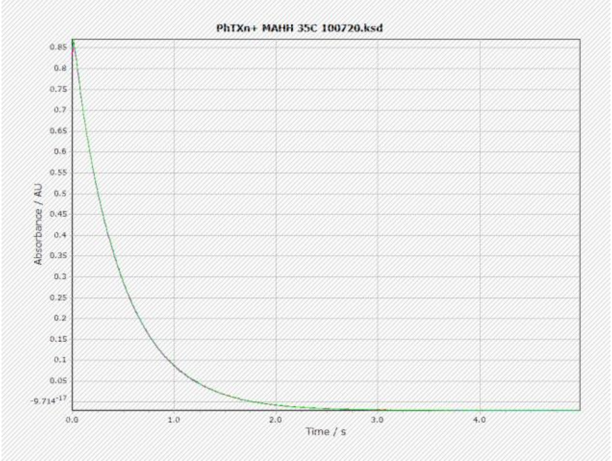

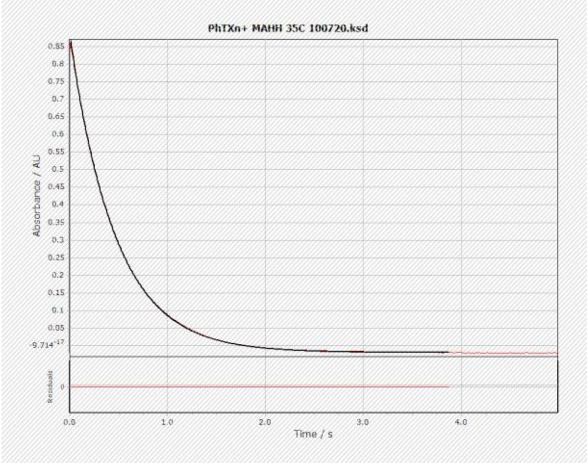

35°C H

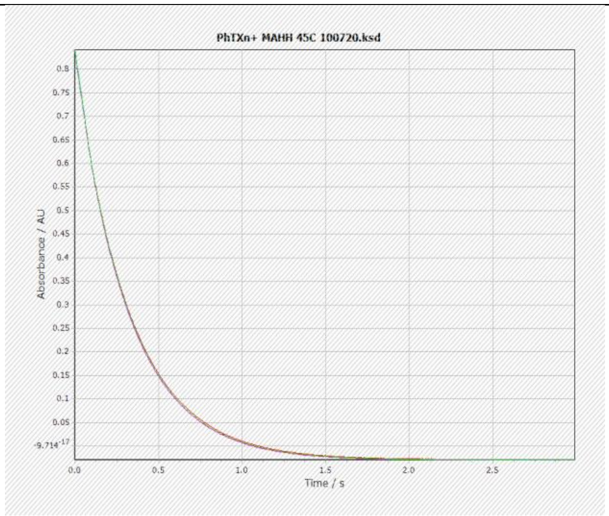

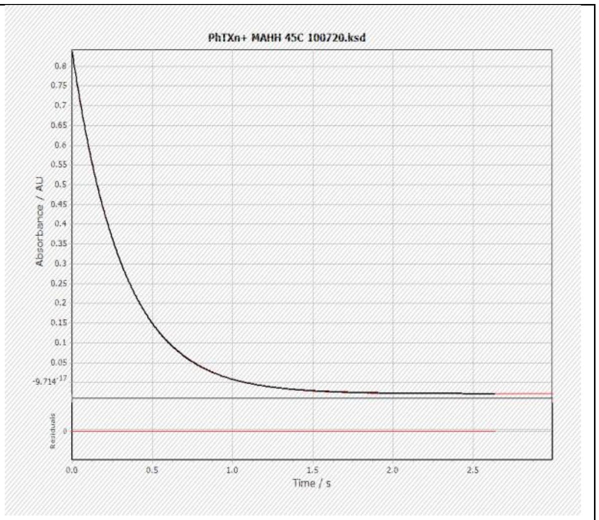

45°C H

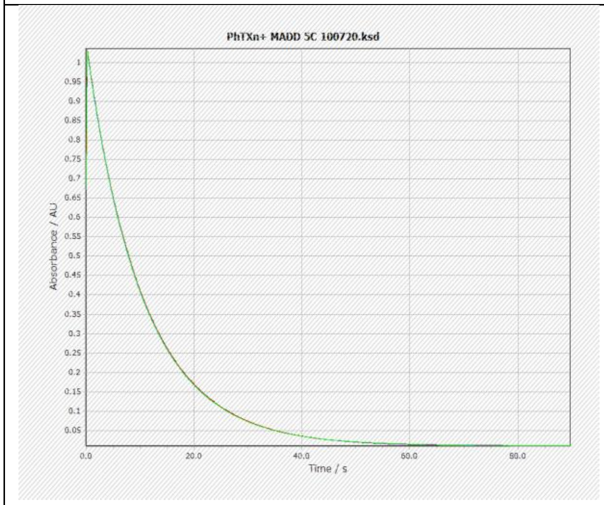

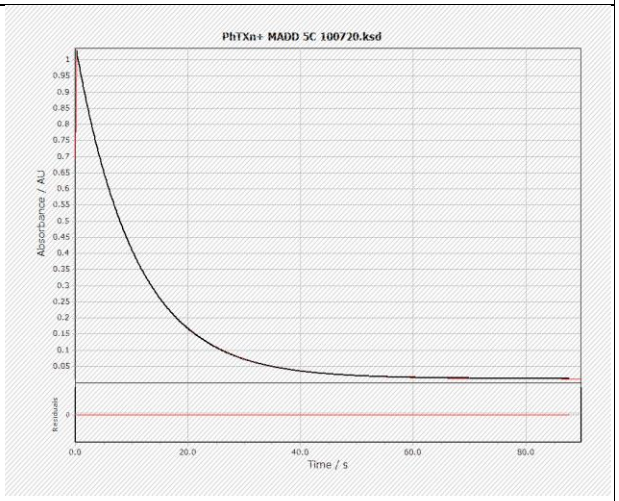

5°C D

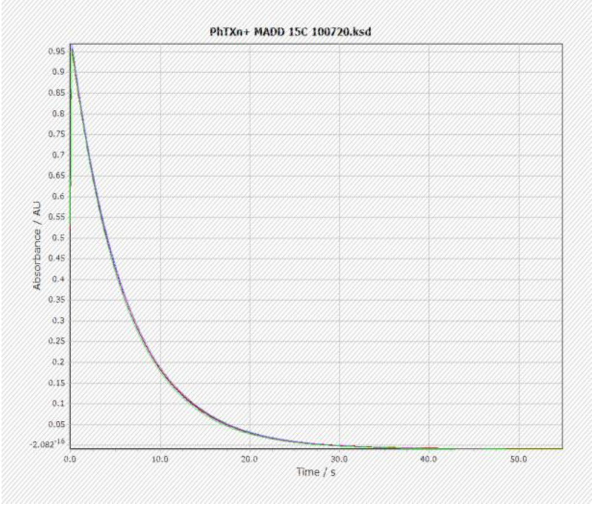

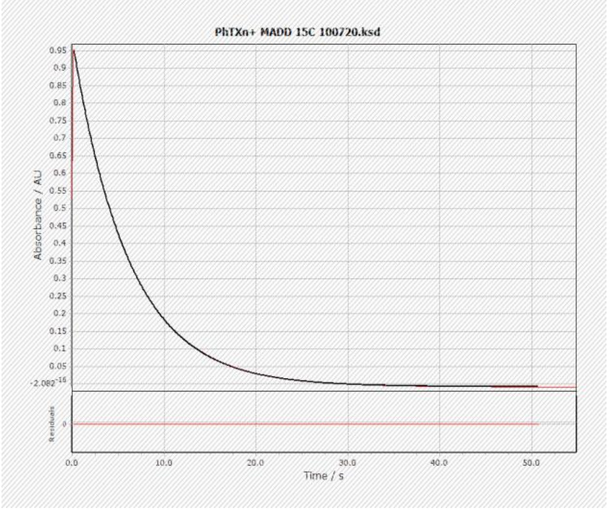

15°C D

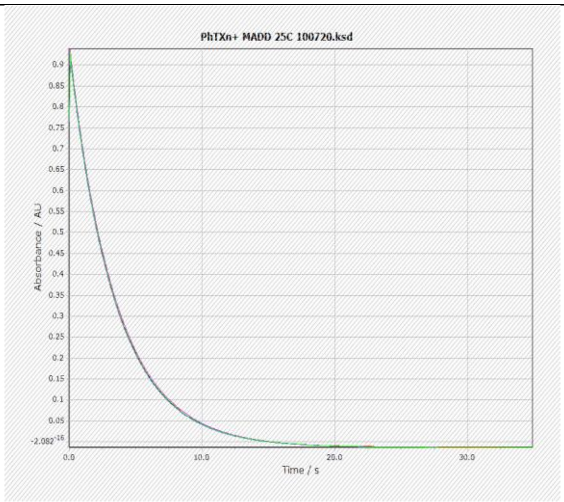

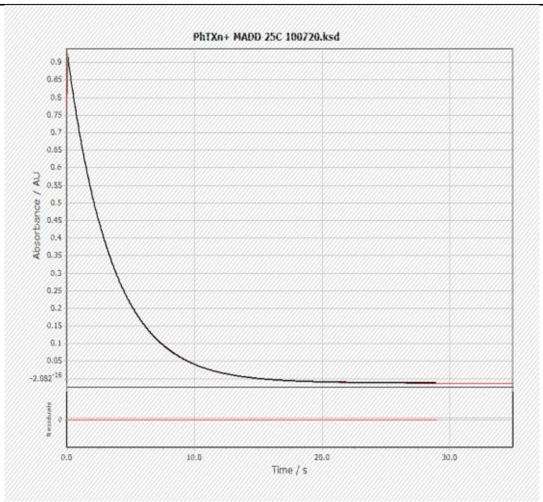

25°C D

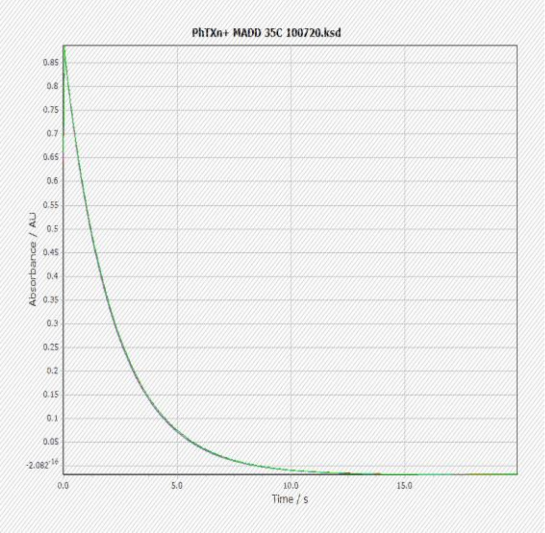

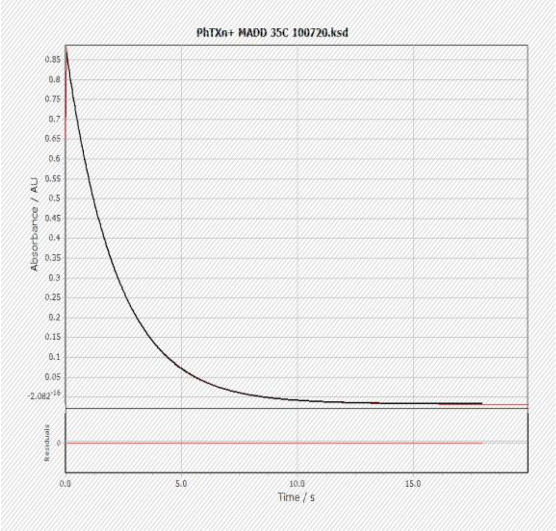

35°C D

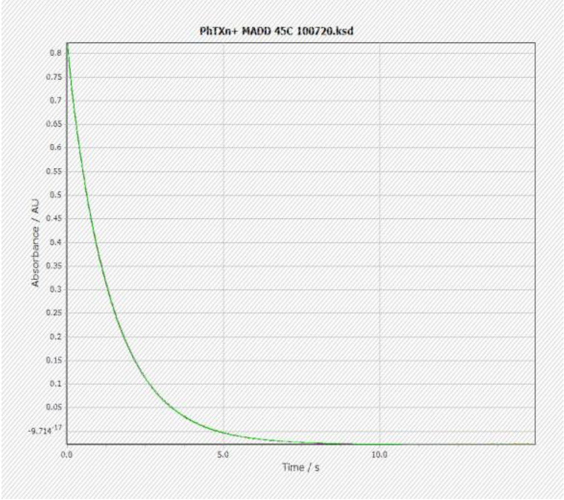

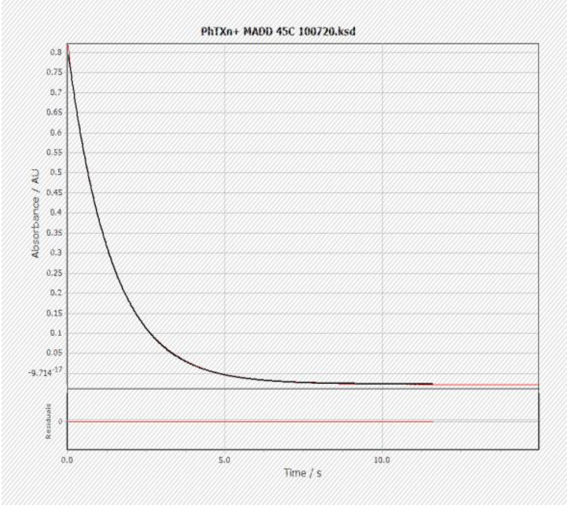

45°C D

Day 3 data (October 15, 2020)

Temp (°C)	Trial H1	Trial H2	Trial H3	Trial H4	Trial H5	Trial H6	Average k_H^{pfo} (s^{-1})	Stdev	k_{2H} ($M^{-1}s^{-1}$)	Stdev [a]
	Pseudo-first-order rate constants k^{pfo} (s^{-1})									
45	3.091	3.116	3.104	3.118	3.112	3.112	3.109	0.010	829.05	2.637
35	2.106	2.117	2.096	2.104	2.100	2.095	2.103	0.008	560.81	2.180
25	1.357	1.353	1.348	1.344	1.368	1.361	1.355	0.009	361.42	2.322
15	0.833	0.838	0.838	0.844	0.848	0.853	0.842	0.007	224.58	1.971
5	0.510	0.508	0.514	0.514	0.525	0.523	0.516	0.007	137.52	1.865

Temp (°C)	Trial D1	Trial D2	Trial D3	Trial D4	Trial D5	Trial D6	Average k_D^{pfo} (s^{-1})	Stdev	k_{2D} ($M^{-1}s^{-1}$)	Stdev [a]
45	0.719	0.720	0.723	0.715	0.713	0.713	0.717	0.004	191.30	1.097
35	0.455	0.455	0.458	0.458	0.455	0.462	0.457	0.003	121.92	0.680
25	0.283	0.288	0.287	0.287	0.291	0.289	0.288	0.003	76.76	0.715
15	0.164	0.163	0.161	0.165	0.167	0.167	0.164	0.002	43.83	0.656
5	0.095	0.093	0.093	0.096	0.096	0.097	0.095	0.001	25.34	0.385

[a] = (Stdev(for k^{pfo})/k^{pfo})*k_{2H}

Six kinetic runs together with the averaged data

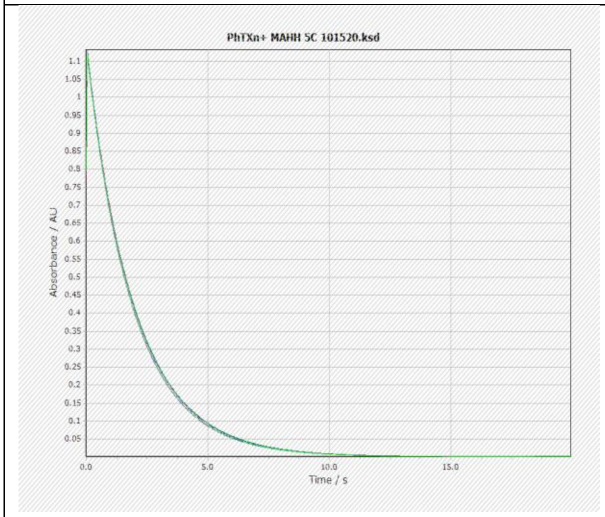

First-order kinetic fit of the averaged data as an example

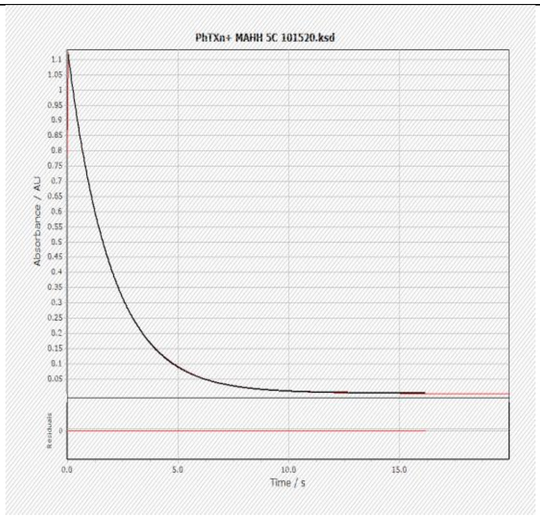

5℃ H

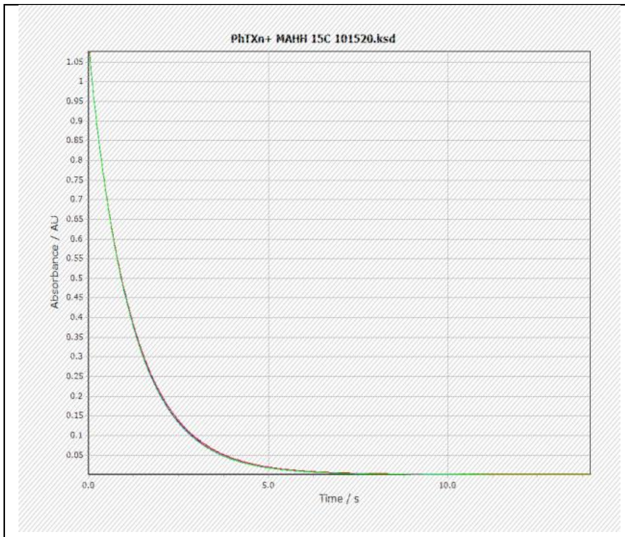

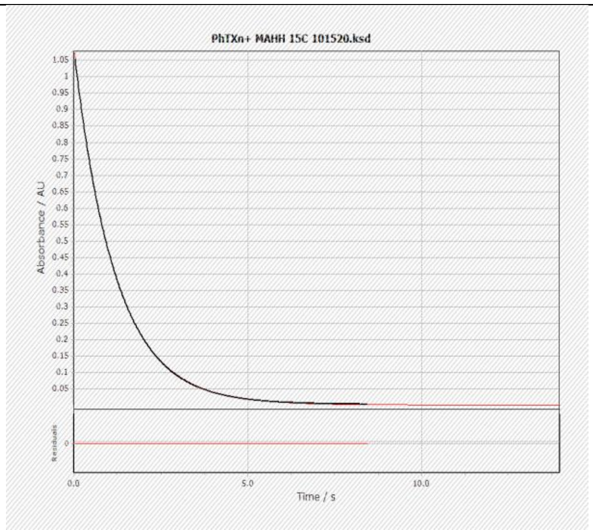

15°C H

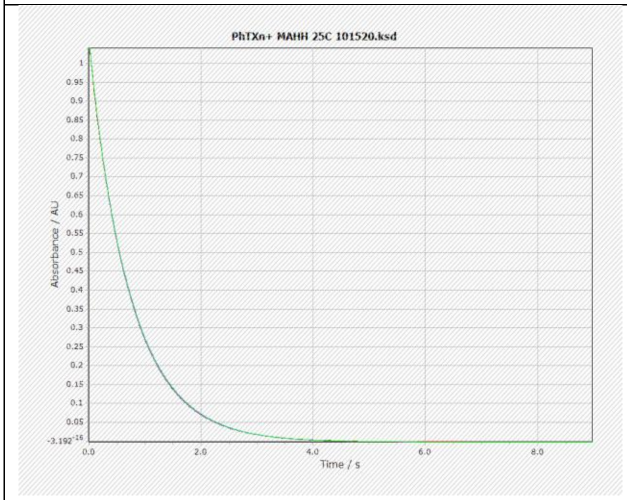

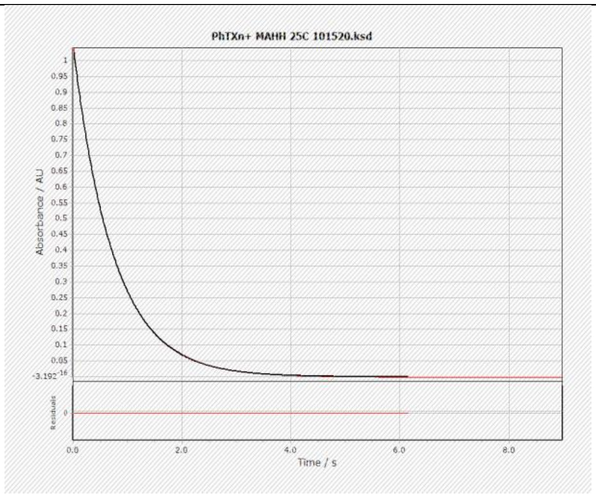

25°C H

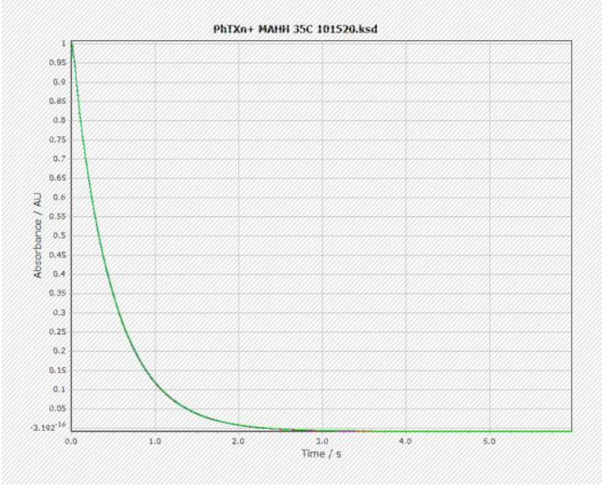

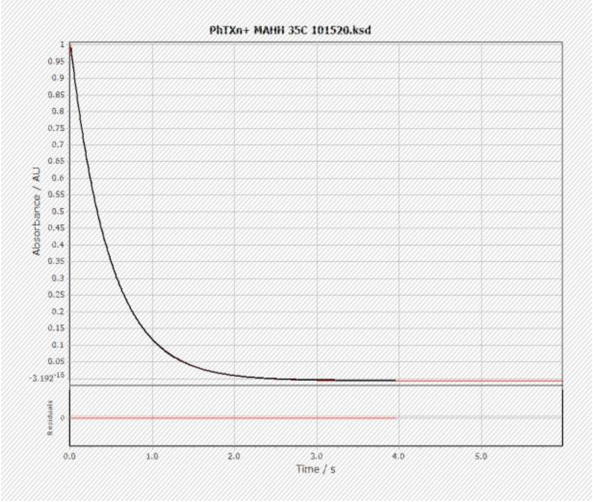

35°C H

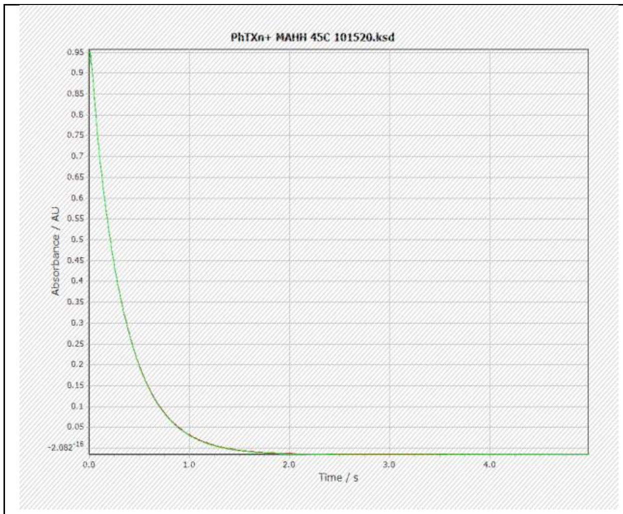
PhTXn+ MAHH 45C 101520.ksd
Absorbance / AU
Time / s

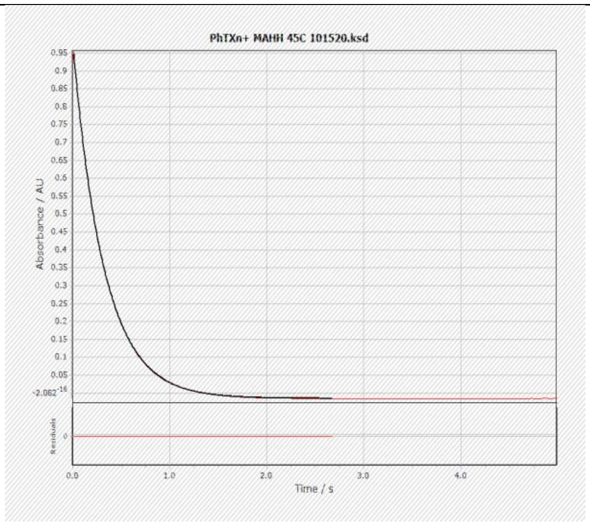
PhTXn+ MAHH 45C 101520.ksd
Absorbance / AU
Residuals
Time / s

45℃ H

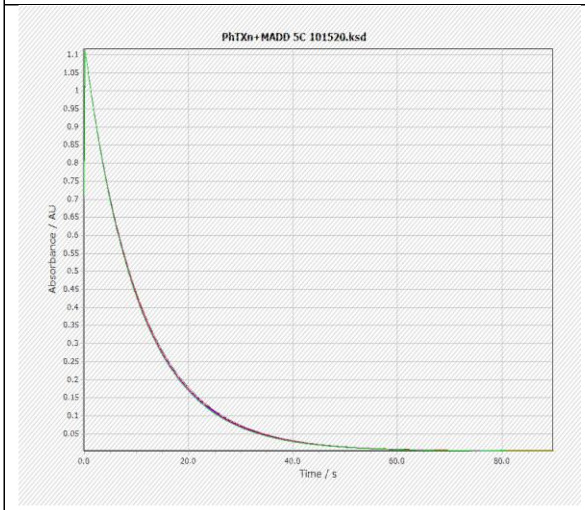
PhTXn+MADD 5C 101520.ksd
Absorbance / AU
Time / s

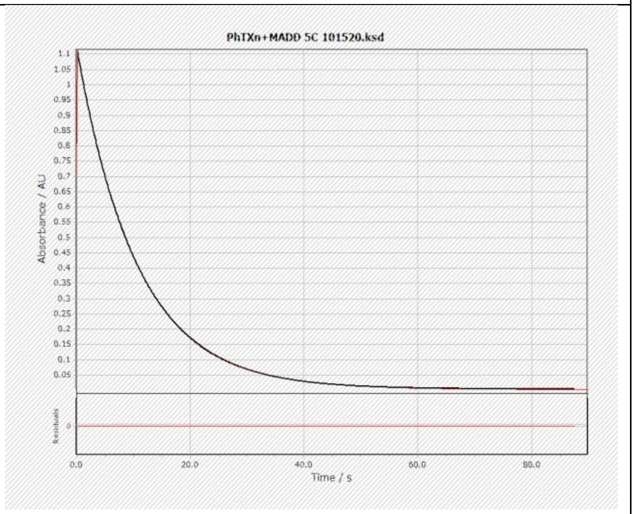
PhTXn+MADD 5C 101520.ksd
Absorbance / AU
Residuals
Time / s

5℃ D

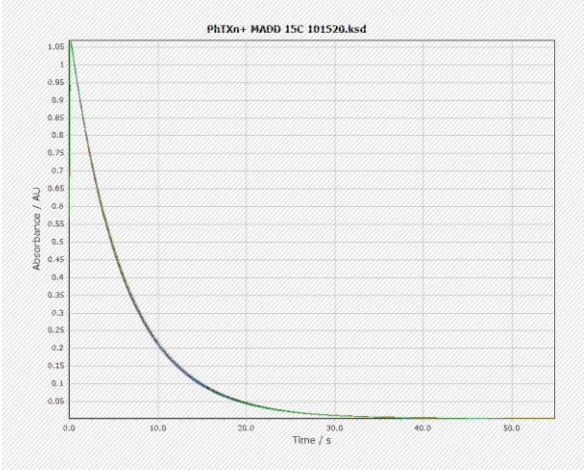
PhTXn+ MADD 15C 101520.ksd
Absorbance / AU
Time / s

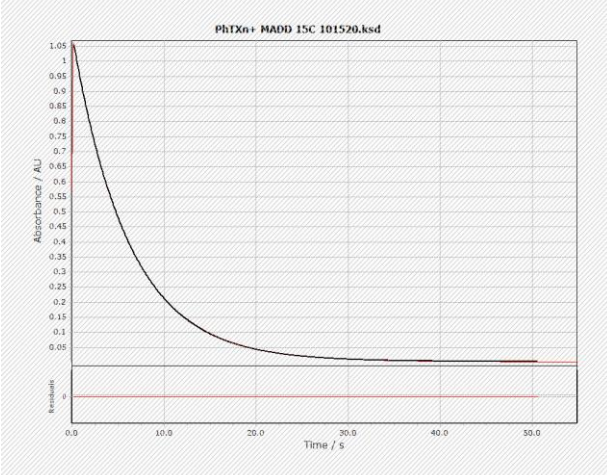
PhTXn+ MADD 15C 101520.ksd
Absorbance / AU
Residuals
Time / s

15℃ D

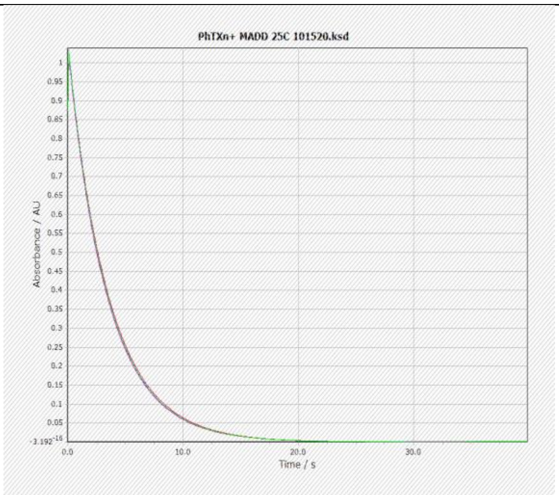

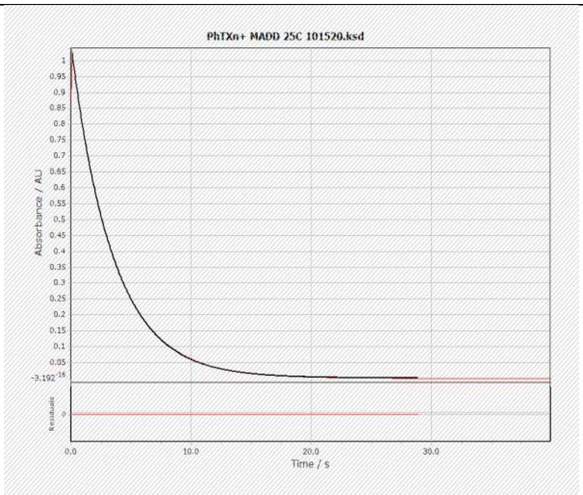

25°C D

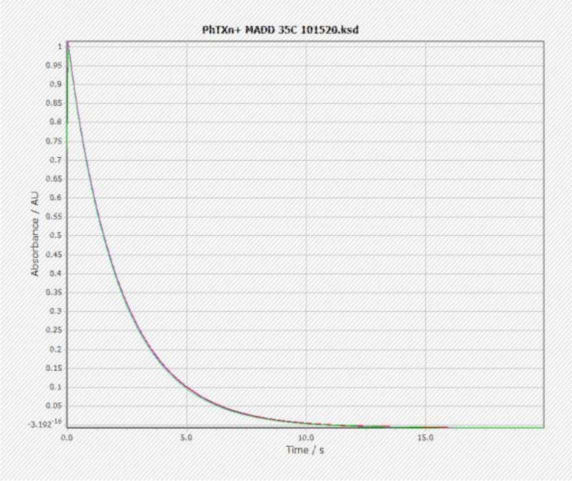

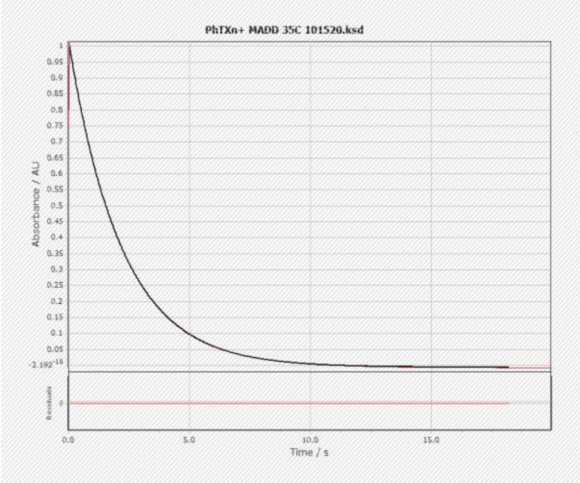

35°C D

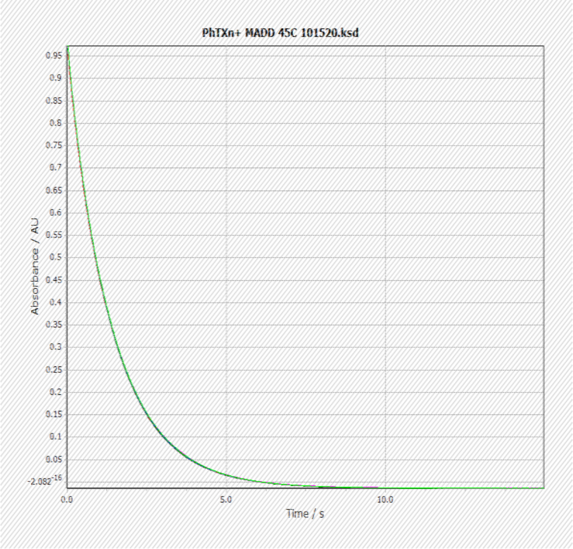

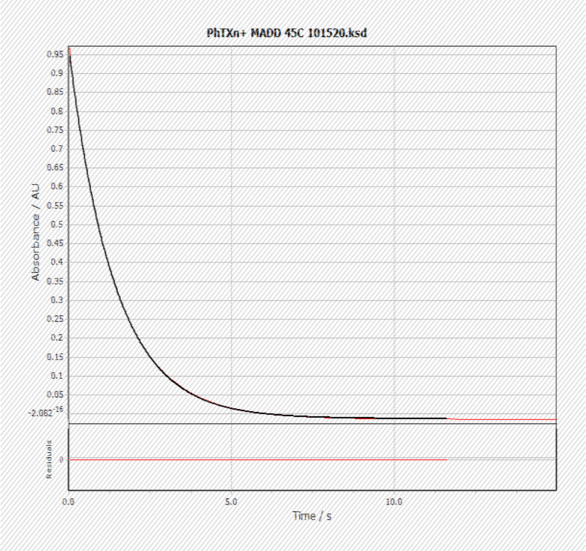

45°C D

Primary kinetic data for the rate constants in Table S3

Date of Measure-ments	***GPhT* Xn^{+}**	Pseudo-first-order rate constants k_{H}^{pfo} (s^{-1}) **Trial H1**	**Trial H2**	**Trial H3**	**Trial H4**	**Trial H5**	**Trial H6**	**Average k_{H}^{pfo} (s^{-1})**	**Stdev**	**k_{2H} (M^{-1}s^{-1})**	**Stdev [a]**
11/06/ 2020	MeO	135.34	134.52	134.58	136.78	133.77	135.50	135.08	1.040	135081.99	1039.61
11/27/ 2020	MeO	133.56	132.65	130.97	133.97	130.27	130.09	131.92	1.698	131917.95	1698.26
11/28/ 2020	MeO	132.33	131.14	131.98	133.87	130.24	131.74	131.88	1.219	131883.52	1219.04
9/30/ 2020	H	274.49	270.67	272.27	268.25	274.13	275.32	271.97	2.577	167365.75	1585.92
10/01/ 2020	H	266.34	269.34	270.29	265.40	269.43	262.24	268.16	2.151	165021.48	1323.61
10/14/ 2020	H	265.36	270.73	268.94	264.64	268.59	270.32	267.65	2.566	164706.91	1578.84
12/03/ 2020	Cl	438.12	463.46	482.23	455.44	449.42	465.17	458.97	15.09	305981.22	10059.28
12/04/ 2020	Cl	476.38	457.07	447.43	462.06	451.17	436.33	455.07	13.65	303382.33	9102.20
12/07/ 2020	Cl	442.87	465.28	464.15	464.77	464.38	459.32	460.13	8.73	306751.50	5818.18

[a] = (Stdev(for k^{pfo})/k^{pfo})*k_{2H}

Six kinetic runs together with the averaged data	First-order kinetic fit of the averaged data as an example
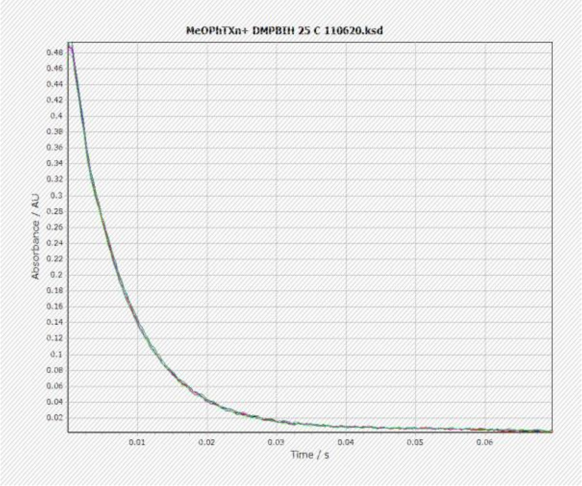	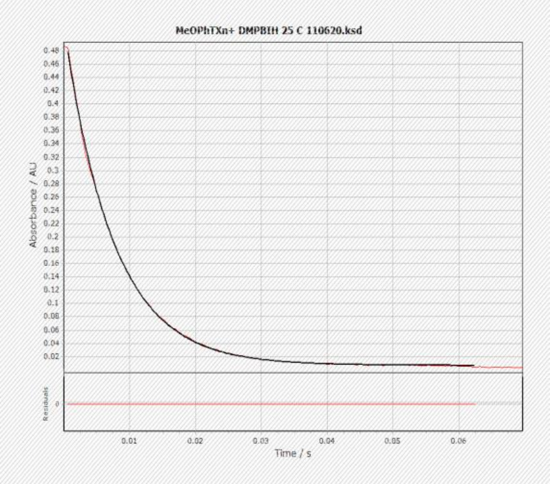

*MeO*PhTXn^{+}

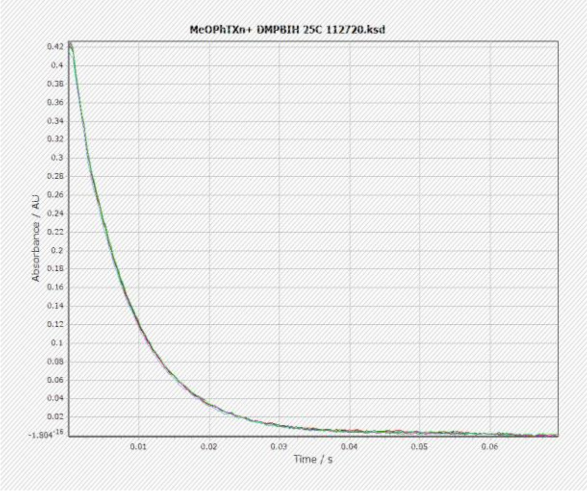	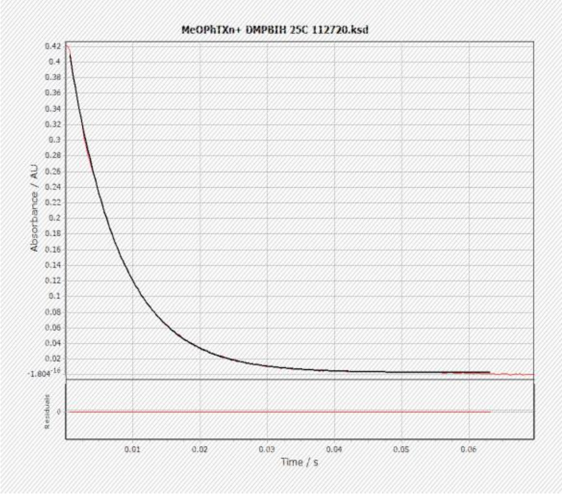

*MeO*PhTXn^{+}

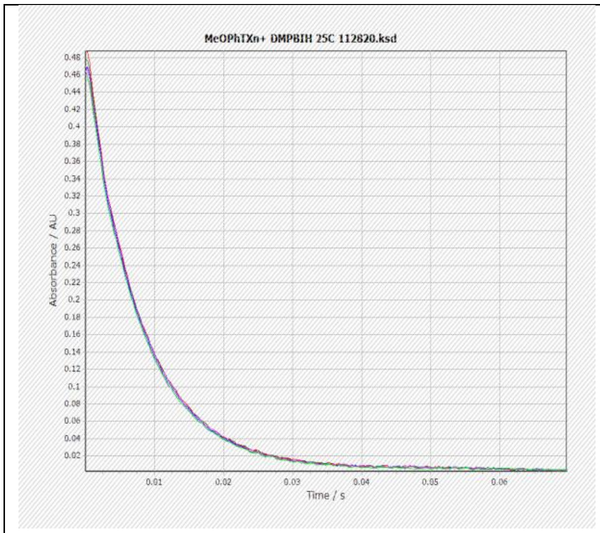

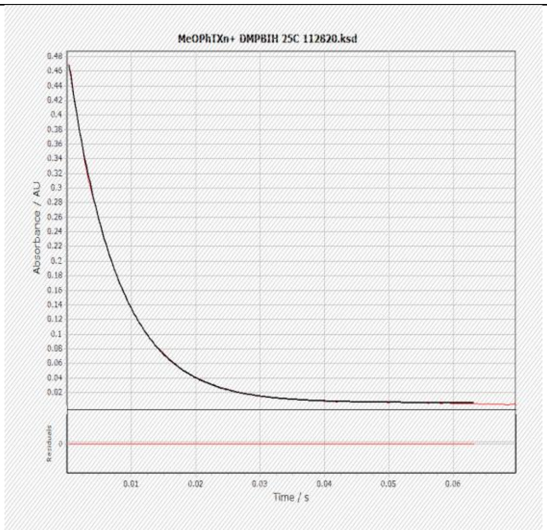

*MeO*PhTXn+

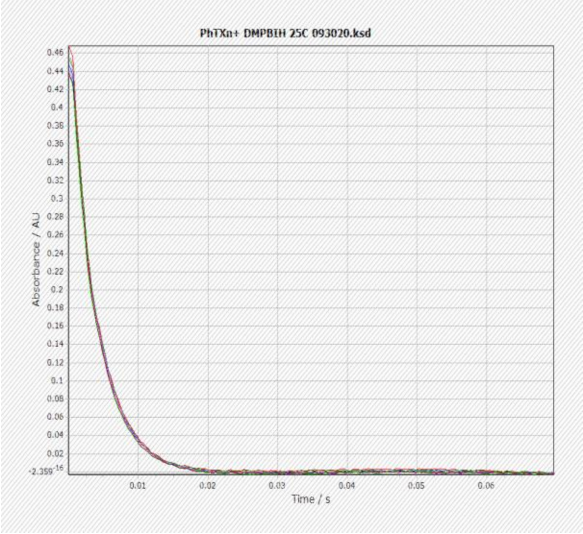

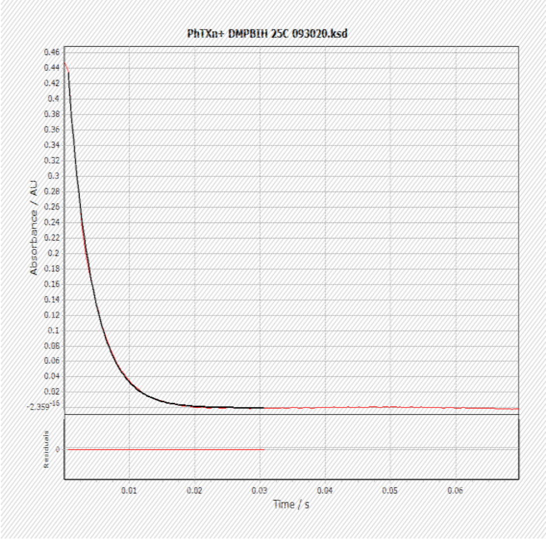

PhTXn+

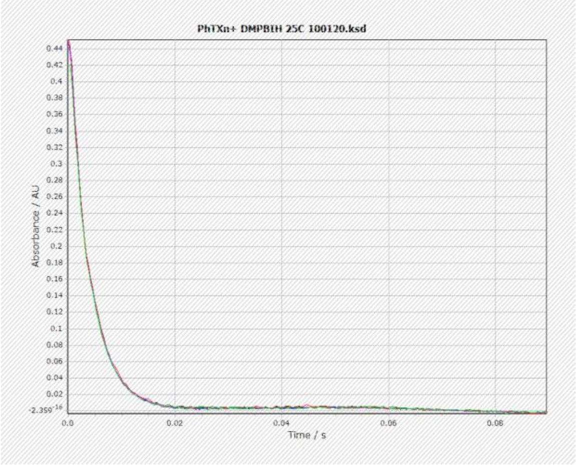

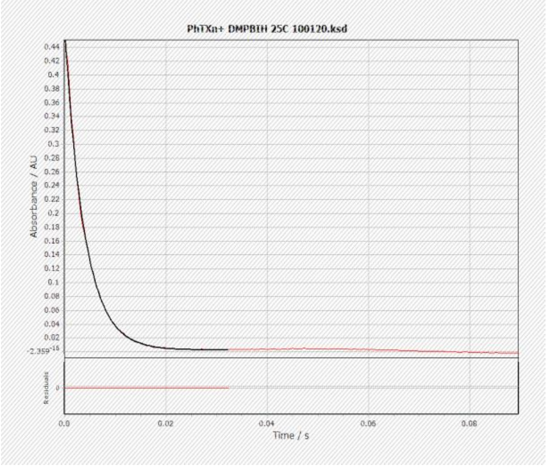

PhTXn+

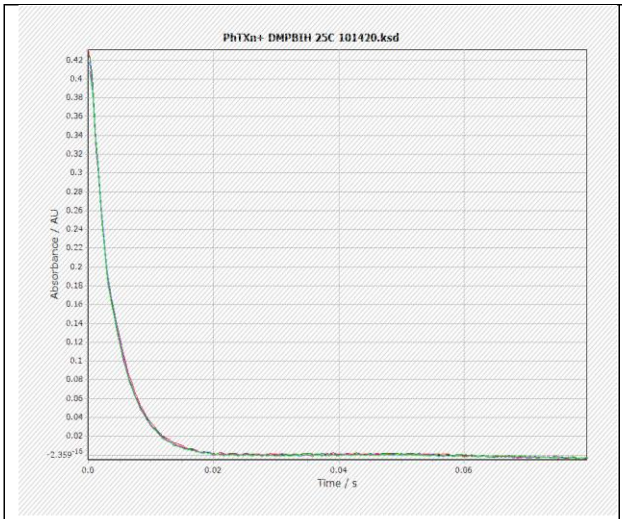

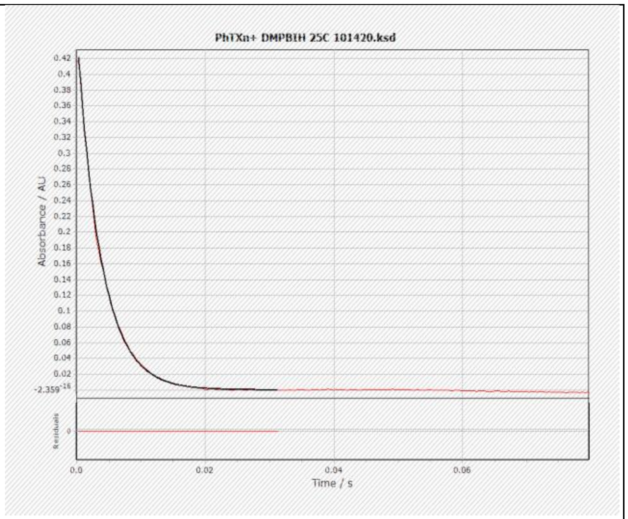

PhTXn^{+}

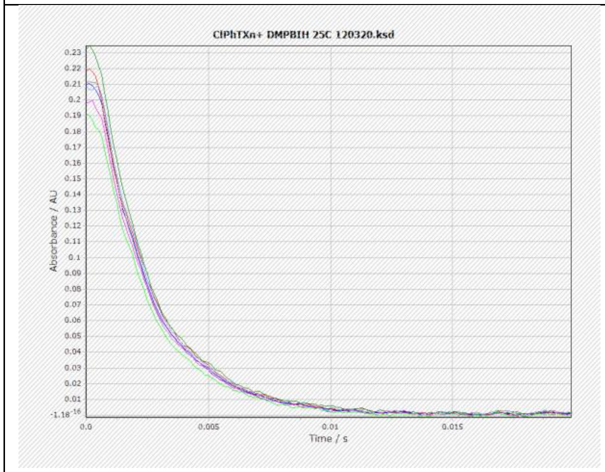

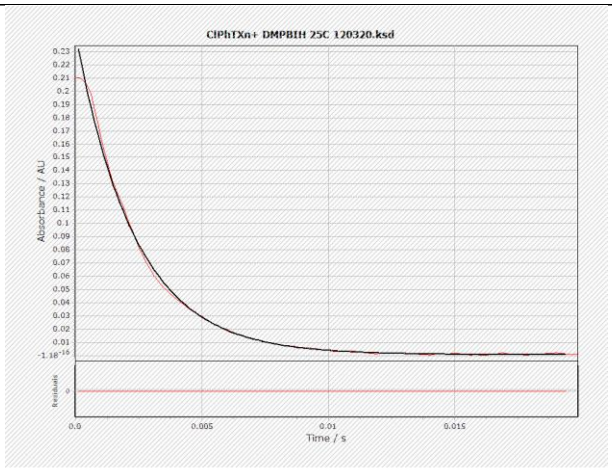

*Cl*PhTXn^{+}

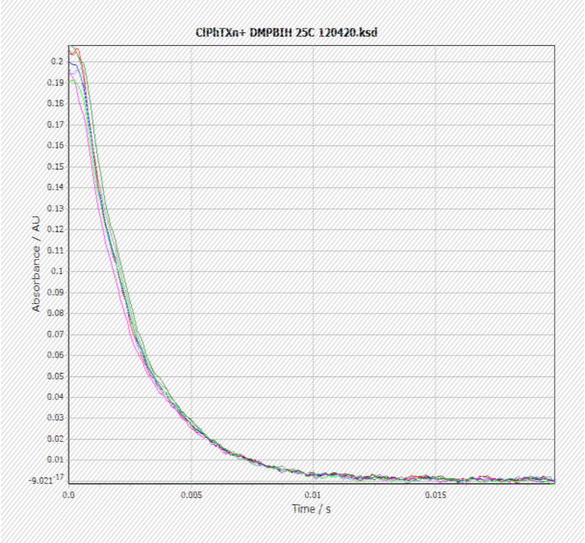

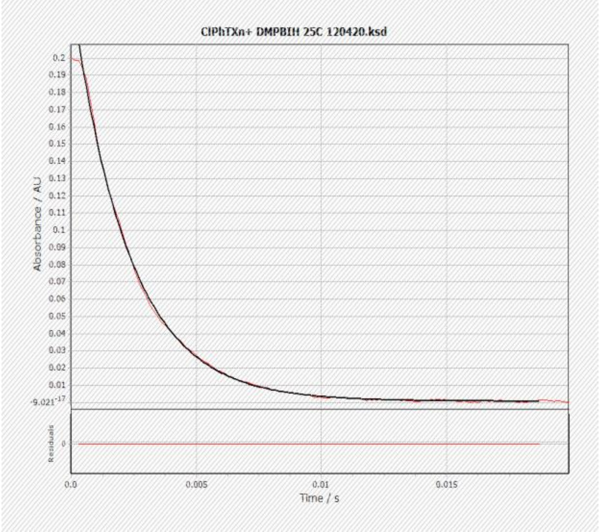

*Cl*PhTXn^{+}

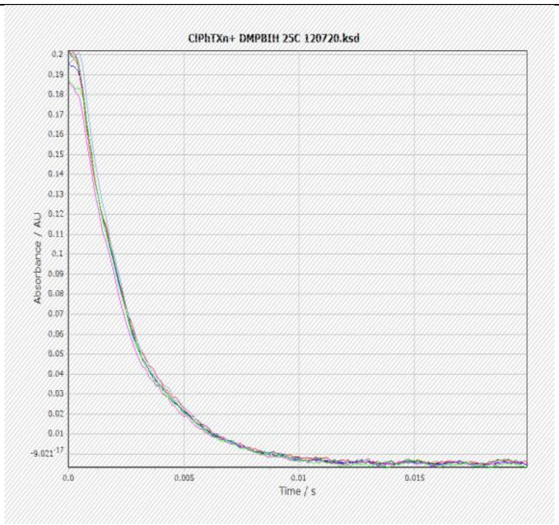

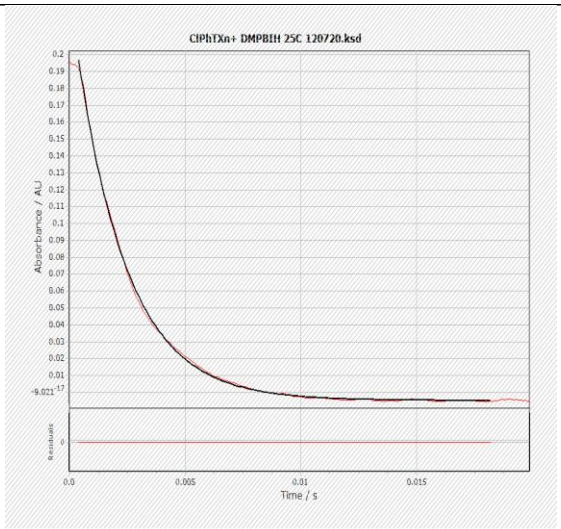

*Cl*PhTXn$^+$

Primary kinetic data for the rate constants in Table S4

For the reactions of PhXn$^+$

Day 1 data (1) (September 22, 2023)

	DMPBIH		*CF$_3$*DMPBIH		*CH$_3$O*DMPBIH [a]	
Runs	k^{pfo} (s^{-1})	k_{2H} ($M^{-1}s^{-1}$)	k^{pfo} (s^{-1})	k_{2H} ($M^{-1}s^{-1}$)	k^{pfo} (s^{-1})	k_{2H} ($M^{-1}s^{-1}$)
1	57.768		0.996		571.017	
2	58.485		1.015		550.407	
3	57.831		1.021		519.471	
4	58.410		1.009		553.207	
5	58.174		1.004		591.141	
6	58.730		1.010		587.826	
Average	58.233	47771.090	1.009	827.893	562.178	461180.007
Stdev	0.307	251.782 [b]	0.006	4.924 [b]	26.558	21786.416 [b]

[a] The rate constants are less reliable as they have reached the limit of rate measurement from our stopped-flow instrument (Use of the data for discussion has been limited, see the main paper.); [b] = (Stdev(for k^{pfo})/k^{pfo})*k_{2H}

Six kinetic runs together with the averaged data	First-order kinetic fit of the averaged data as an example

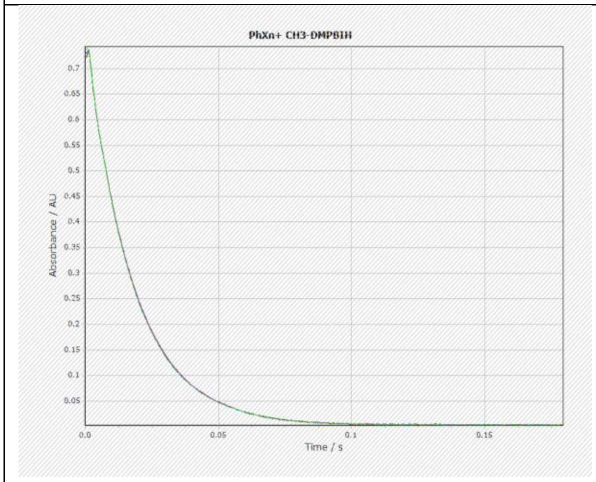

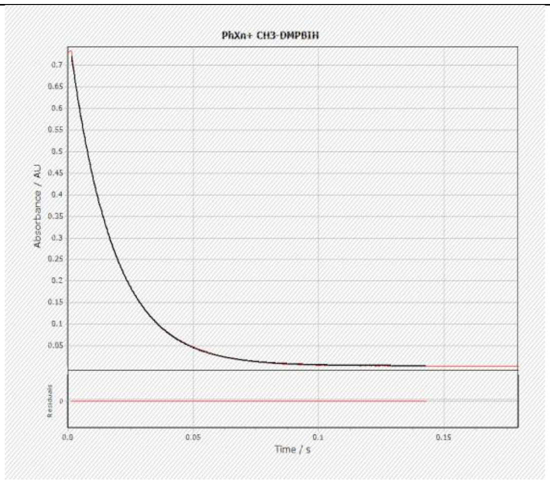

DMPBIH

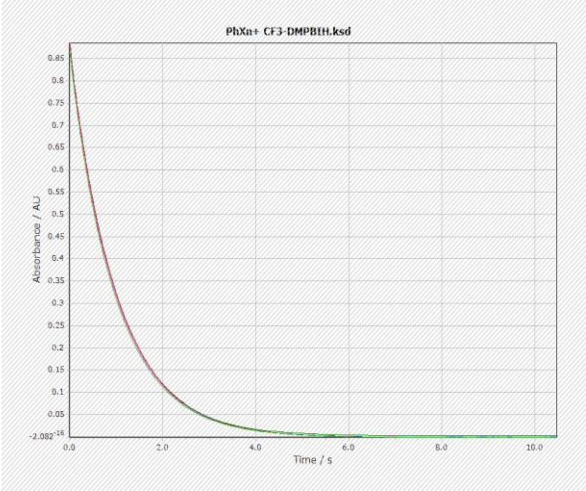

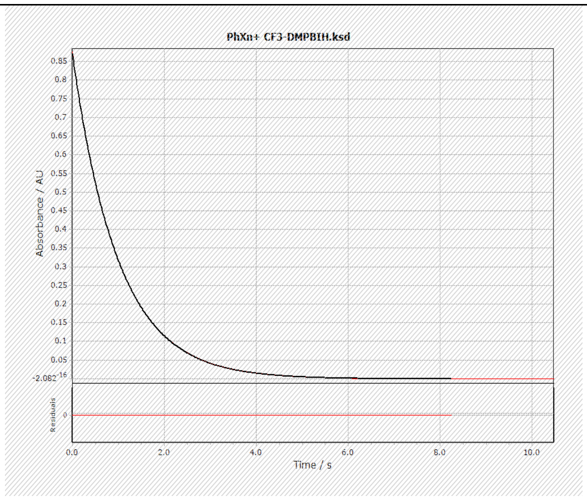

*CF3*DMPBIH

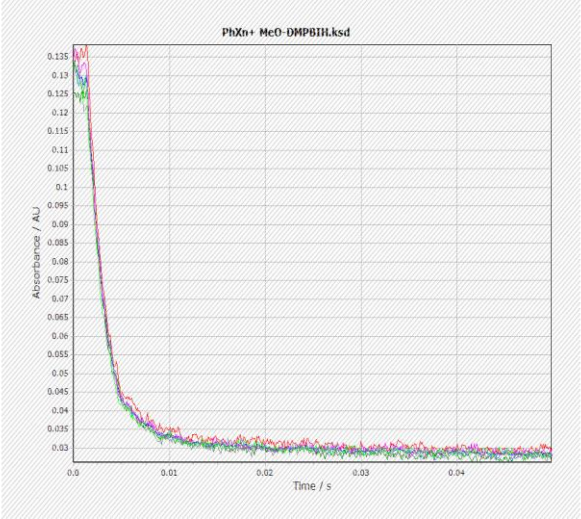

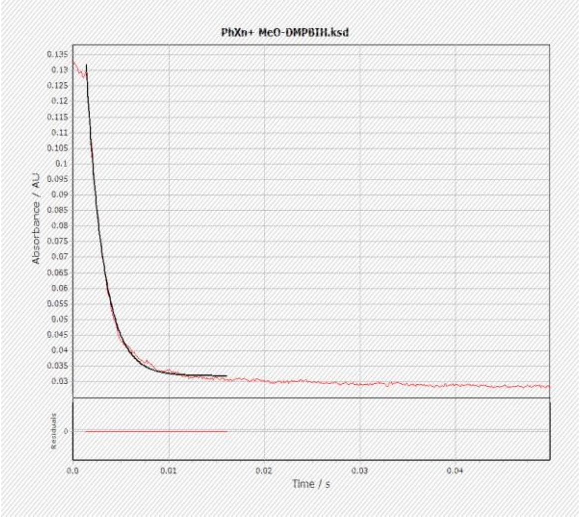

*MeO*DMPBIH

Day 1 data (2) (September 22, 2023)

	DMPBIH		*CF3*DMPBIH		*CH3O*DMPBIH [a]	
Runs	k^{pfo} (s^{-1})	k_{2H} ($M^{-1}s^{-1}$)	k^{pfo} (s^{-1})	k_{2H} ($M^{-1}s^{-1}$)	k^{pfo} (s^{-1})	k_{2H} ($M^{-1}s^{-1}$)
1	60.500		1.011		547.647	
2	59.523		1.000		568.538	
3	59.541		1.010		537.716	
4	58.953		1.009		555.419	
5	58.785		1.021		605.980	
6	59.692		1.020		579.094	
Average	59.499	48809.506	1.012	830.038	565.732	464095.481
Stdev	0.369	302.576 [b]	0.008	6.407 [b]	22.992	18861.043 [b]

[a] The rate constants are less reliable as they have reached the limit of rate measurement from our stopped-flow instrument (Use of the data for discussion has been limited, see the main paper.); [b] = (Stdev(for k^{pfo})/k^{pfo})*k_{2H}

Six kinetic runs together with the averaged data	First-order kinetic fit of the averaged data as an example
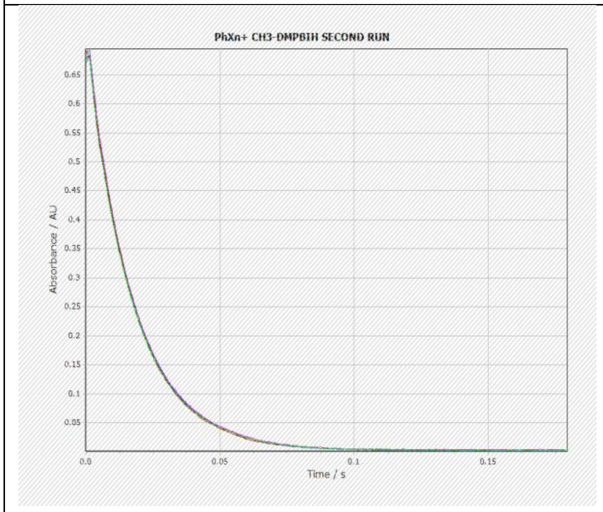	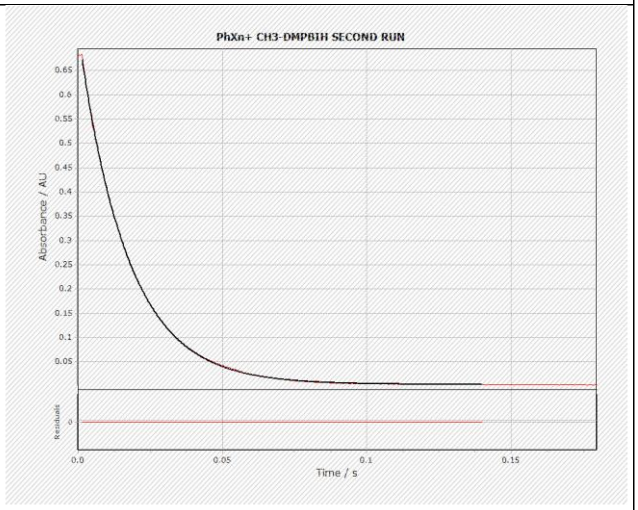
DMPBIH	
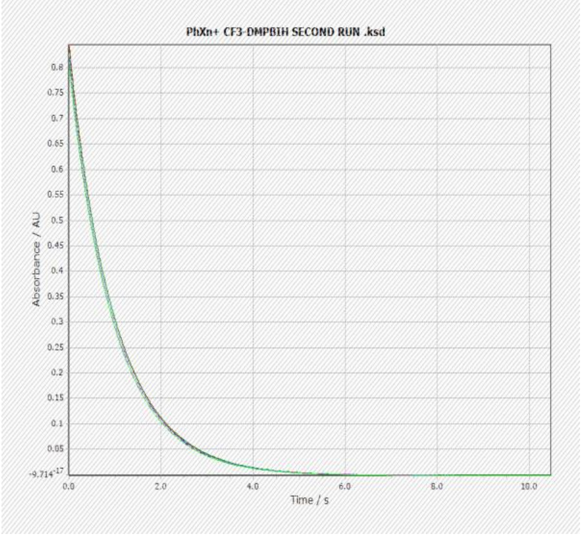	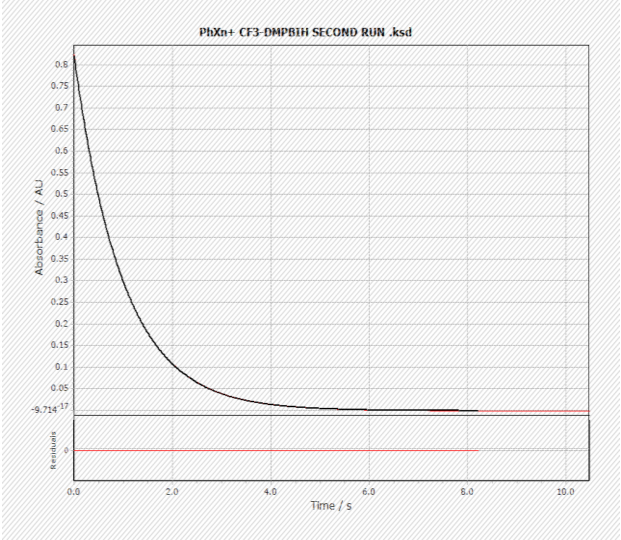
*CF3*DMPBIH	

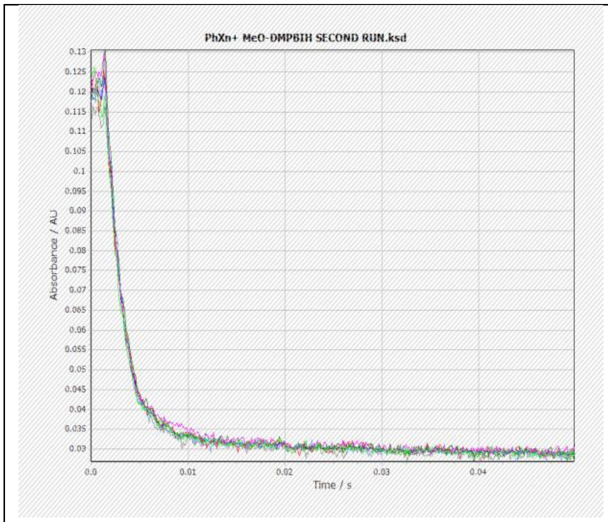	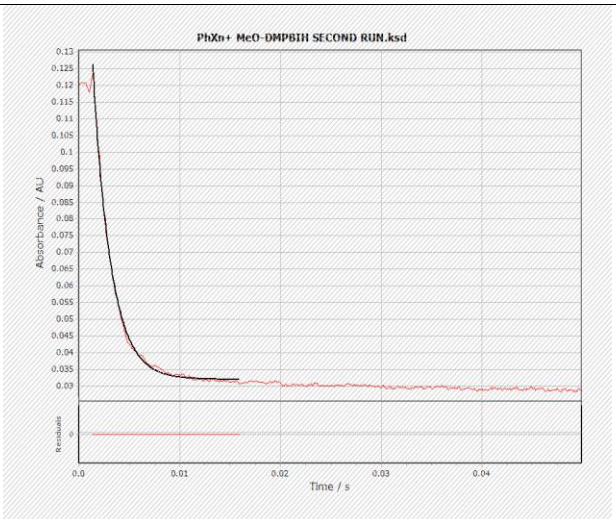
*MeO*DMPBIH	

Day 2 data (1) (September 19, 2023)

	DMPBIH		*CF₃*DMPBIH		*CH₃O*DMPBIH [a]	
Runs	k^{pfo} (s^{-1})	k_{2H} ($M^{-1}s^{-1}$)	k^{pfo} (s^{-1})	k_{2H} ($M^{-1}s^{-1}$)	k^{pfo} (s^{-1})	k_{2H} ($M^{-1}s^{-1}$)
1	56.520		1.085		598.451	
2	57.178		1.055		584.174	
3	57.634		1.053		633.228	
4	56.753		1.053		607.395	
5	57.335		1.071		610.273	
6	57.985		1.046		616.536	
Average	57.234	46951.734	1.061	870.051	608.343	499050.831
Stdev	0.420	344.764 [b]	0.009	7.103 [b]	15.867	13016.702 [b]

[a] The rate constants are less reliable as they have reached the limit of rate measurement from our stopped-flow instrument (Use of the data for discussion has been limited, see the main paper.); [b] = (Stdev(for k^{pfo})/k^{pfo})*k_{2H}

Six kinetic runs together with the averaged data	First-order kinetic fit of the averaged data as an example
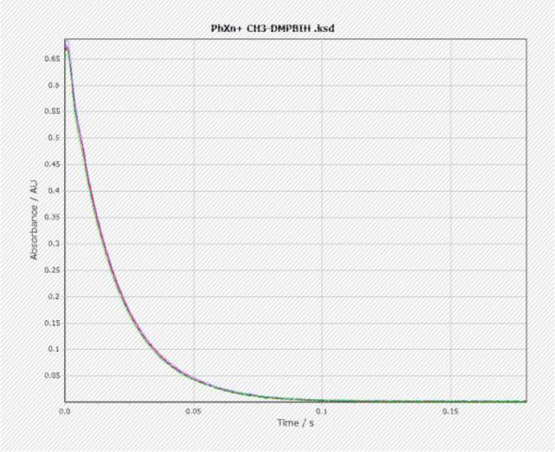	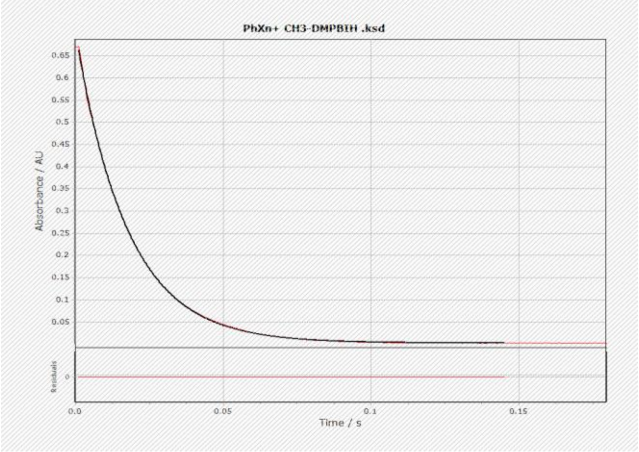
DMPBIH	

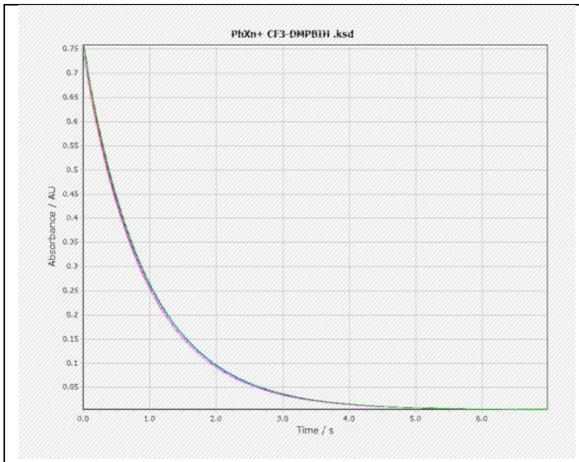

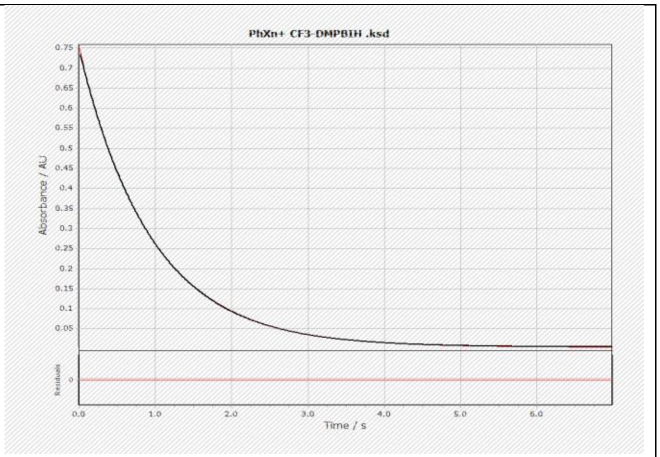

*CF3*DMPBIH

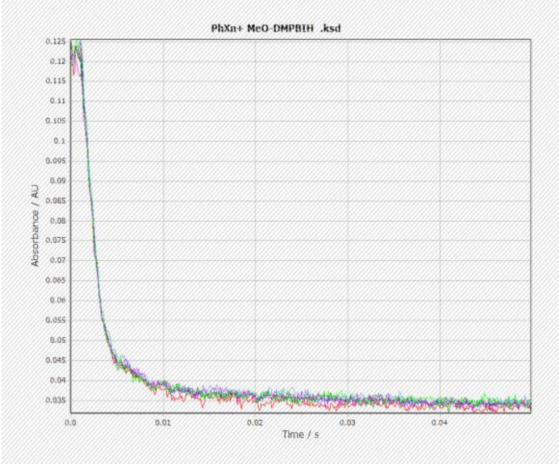

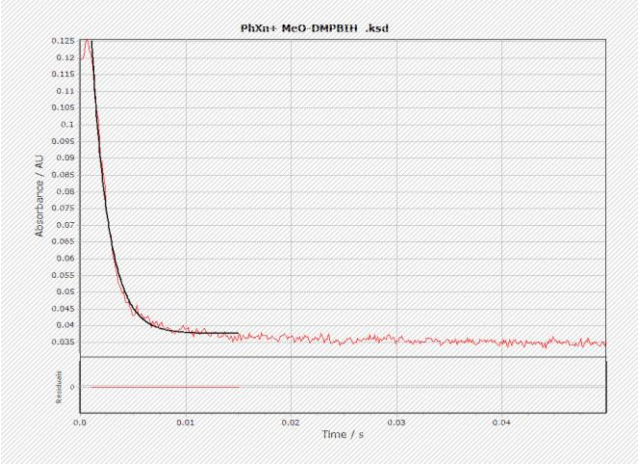

*MeO*DMPBIH

Day 2 data (2) (September 19, 2023)

	DMPBIH		CF_3DMPBIH		CH_3ODMPBIH [a]	
Runs	k^{pfo} (s^{-1})	k_{2H} ($M^{-1}s^{-1}$)	k^{pfo} (s^{-1})	k_{2H} ($M^{-1}s^{-1}$)	k^{pfo} (s^{-1})	k_{2H} ($M^{-1}s^{-1}$)
1	Not determined		1.047		662.602	
2			1.047		716.505	
3			1.049		726.358	
4			1.045		708.719	
5			1.057		728.384	
6			1.051		774.856	
Average			1.049	860.760	719.571	590295.830
Stdev			0.004	3.346 **[b]**	23.522	19296.334 **[b]**

[a] The rate constants are less reliable as they have reached the limit of rate measurement from our stopped-flow instrument (Use of the data for discussion has been limited, see the main paper.); [b] = (Stdev(for k^{pfo})/k^{pfo})*k_{2H}

<table>
<tr><td>Six kinetic runs together with the averaged data</td><td>First-order kinetic fit of the averaged data as an example</td></tr>
<tr><td>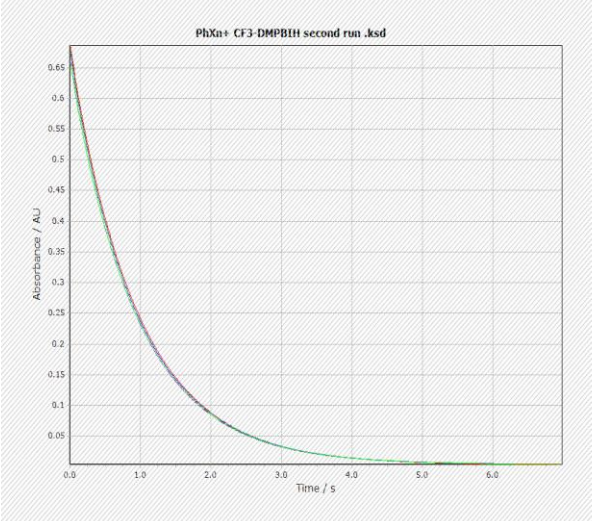
</td><td>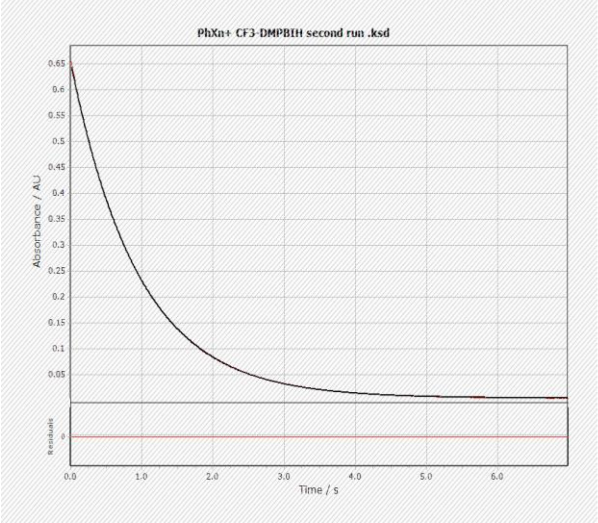
</td></tr>
<tr><td colspan="2">CF3DMPBIH</td></tr>
<tr><td>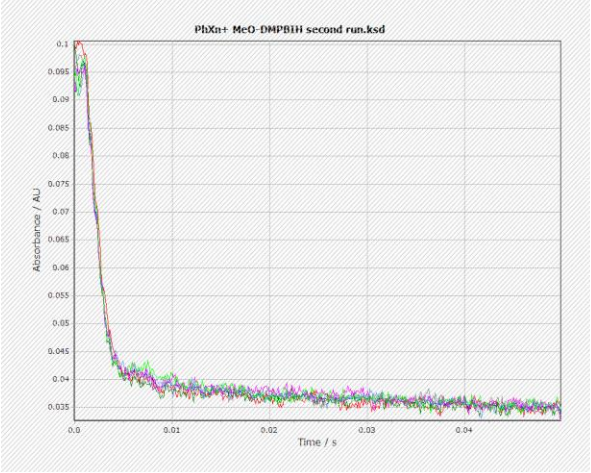
</td><td>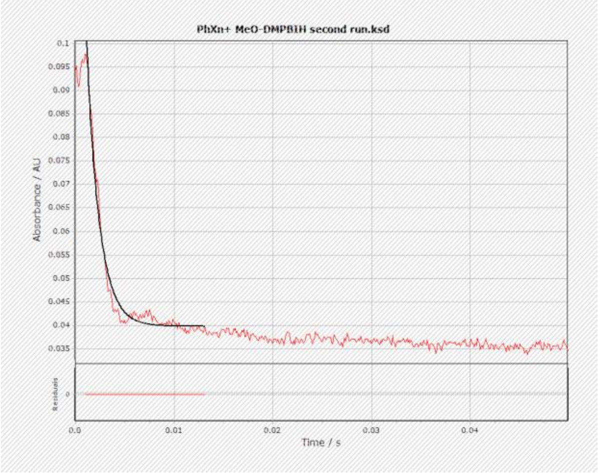
</td></tr>
<tr><td colspan="2">MeODMPBIH</td></tr>
</table>

For the reactions of PhTXn⁺

Day 1 data (1) (September 19, 2023)

	DMPBIH		*CF₃*DMPBIH		*CH₃O*DMPBIH [a]	
Runs	k^{pfo} (s^{-1})	k_{2H} ($M^{-1}s^{-1}$)	k^{pfo} (s^{-1})	k_{2H} ($M^{-1}s^{-1}$)	k^{pfo} (s^{-1})	k_{2H} ($M^{-1}s^{-1}$)
1	187.264		1.644		958.503	
2	188.161		1.625		1006.277	
3	186.773		1.638		979.089	
4	188.780		1.636		988.658	
5	189.216		1.634		937.320	
6	189.996		1.642		898.242	
Average	188.365	154524.132	1.637	1342.512	961.348	788636.766
Stdev	1.089	893.545 [b]	0.006	4.554 [b]	39.083	32061.857 [b]

[a] The rate constants are less reliable as they have reached the limit of rate measurement from our stopped-flow instrument (Use of the data for discussion has been limited, see the main paper.); [b] = (Stdev(for k^{pfo})/k^{pfo})*k_{2H}

Six kinetic runs together with the averaged data	First-order kinetic fit of the averaged data as an example

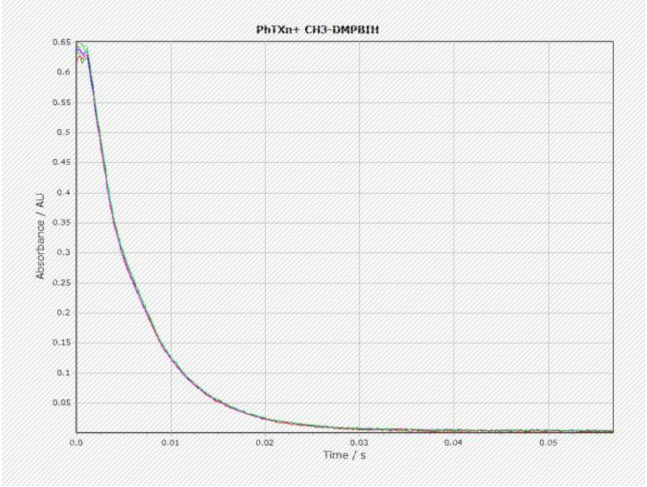

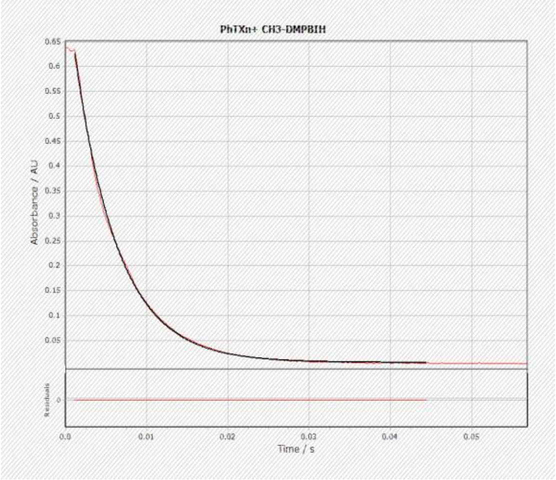

DMPBIH

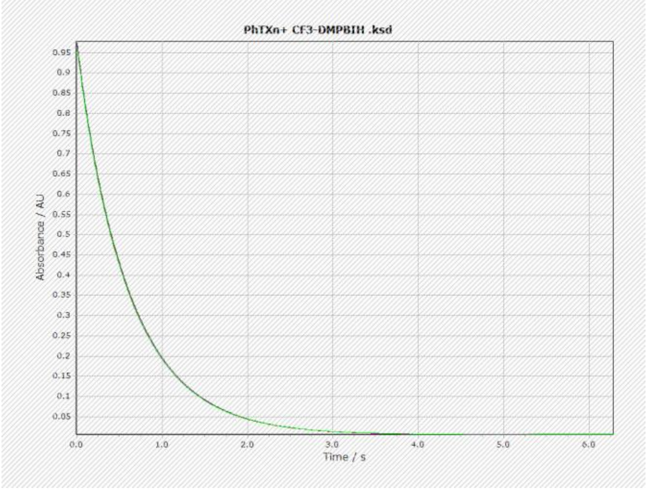

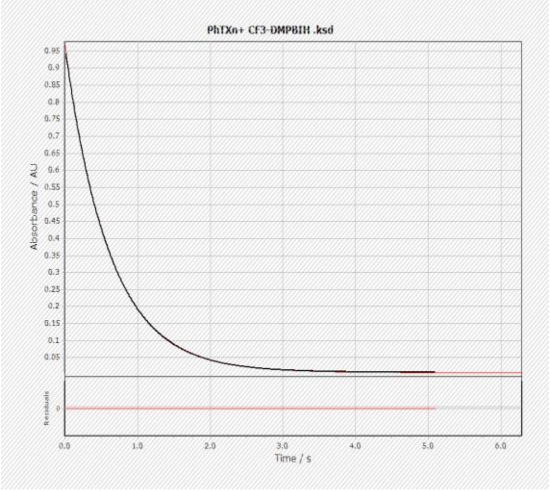

*CF3*DMPBIH

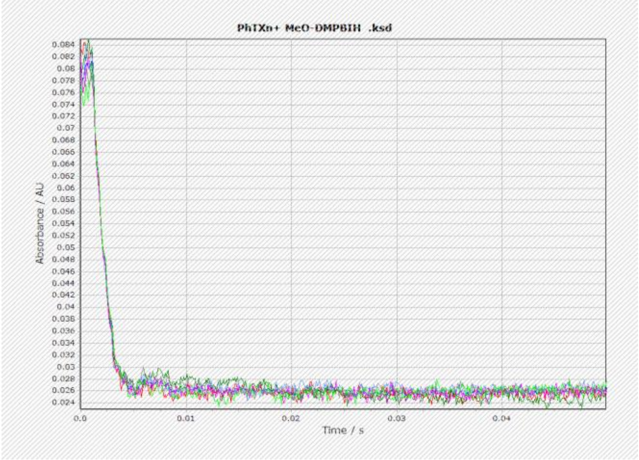

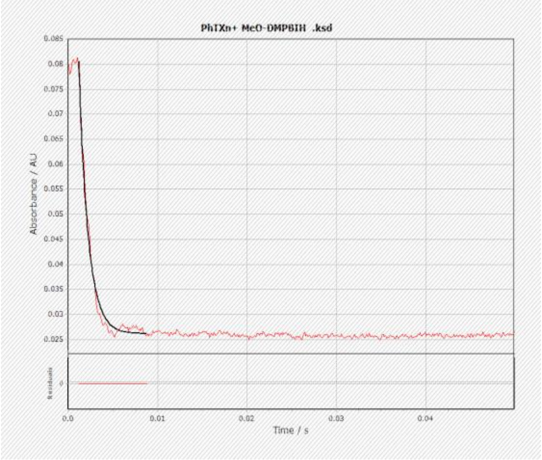

*MeO*DMPBIH

Day 1 data (2) (September 19, 2023)

	DMPBIH		*CF₃*DMPBIH		*CH₃O*DMPBIH [a]	
Runs	k^{pfo} (s^{-1})	k_{2H} ($M^{-1}s^{-1}$)	k^{pfo} (s^{-1})	k_{2H} ($M^{-1}s^{-1}$)	k^{pfo} (s^{-1})	k_{2H} ($M^{-1}s^{-1}$)
1	Not determined		1.659		816.942	
2			1.640		855.336	
3			1.643		963.220	
4			1.659		921.633	
5			1.642		961.752	
6			1.639		958.656	
Average			1.647	1351.176	912.923	748911.411
Stdev			0.007	6.104 **[b]**	42.100	34536.865 **[b]**

[a] The rate constants are less reliable as they have reached the limit of rate measurement from our stopped-flow instrument (Use of the data for discussion has been limited, see the main paper.); [b] = (Stdev(for k^{pfo})/k^{pfo})*k_{2H}

Six kinetic runs together with the averaged data	First-order kinetic fit of the averaged data as an example
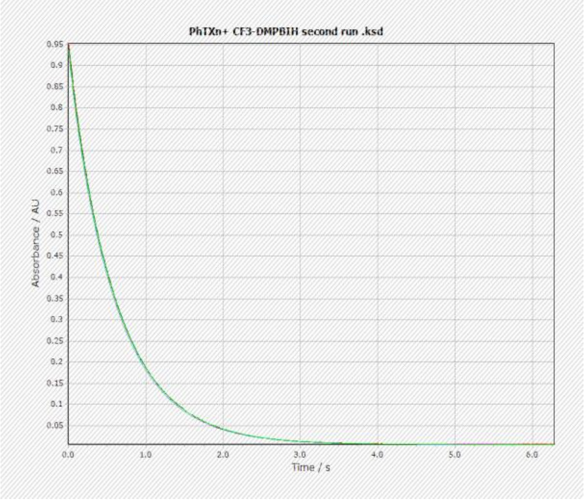	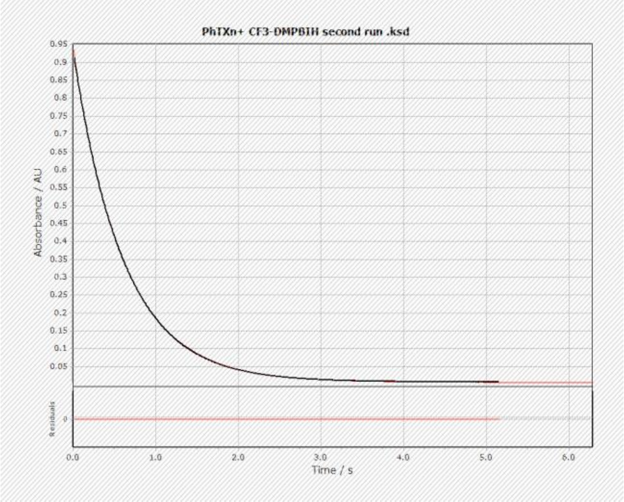
*CF3*DMPBIH	
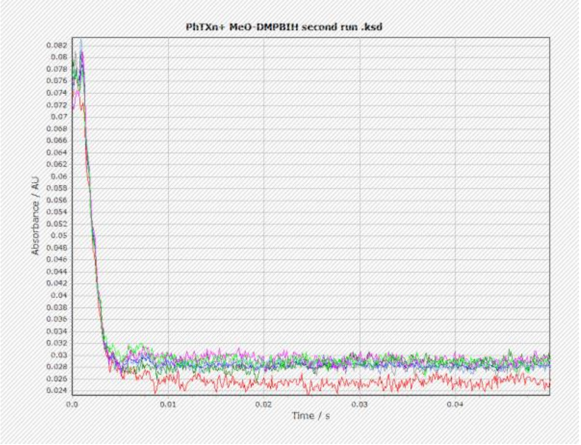	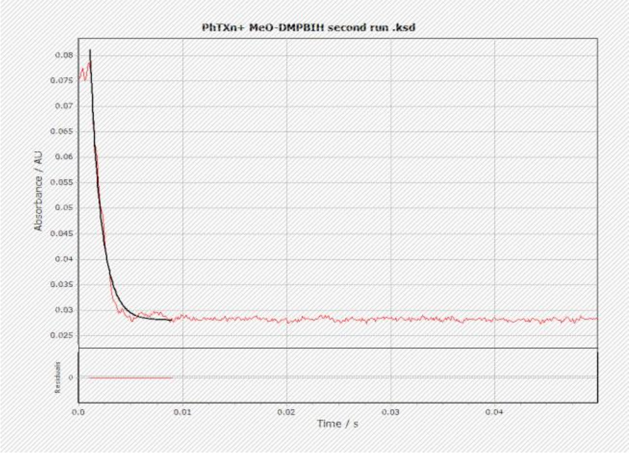
*MeO*DMPBIH	

Day 2 data (1) (September 22, 2023)

	DMPBIH		*CF₃*DMPBIH		*CH₃O*DMPBIH [a]	
Runs	k^{pfo} (s^{-1})	k_{2H} ($M^{-1}s^{-1}$)	k^{pfo} (s^{-1})	k_{2H} ($M^{-1}s^{-1}$)	k^{pfo} (s^{-1})	k_{2H} ($M^{-1}s^{-1}$)
1	192.142		1.604		833.562	
2	187.676		1.632		879.772	
3	189.130		1.591		850.393	
4	190.362		1.607		885.267	
5	186.109		1.617		958.696	
6	190.332		1.614		933.545	
Average	189.292	155284.498	1.611	1321.313	890.206	730275.468
Stdev	1.652	1355.017 **[b]**	0.013	11.068 **[b]**	39.377	32302.638 **[b]**

[a] The rate constants are less reliable as they have reached the limit of rate measurement from our stopped-flow instrument (Use of the data for discussion has been limited, see the main paper.); [b] = (Stdev(for k^{pfo})/k^{pfo})*k_{2H}

<table>
<tr><td>Six kinetic runs together with the averaged data</td><td>First-order kinetic fit of the averaged data as an example</td></tr>
<tr><td>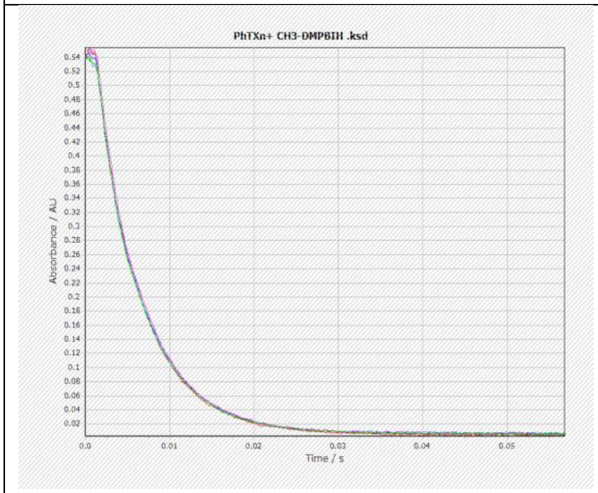
</td><td>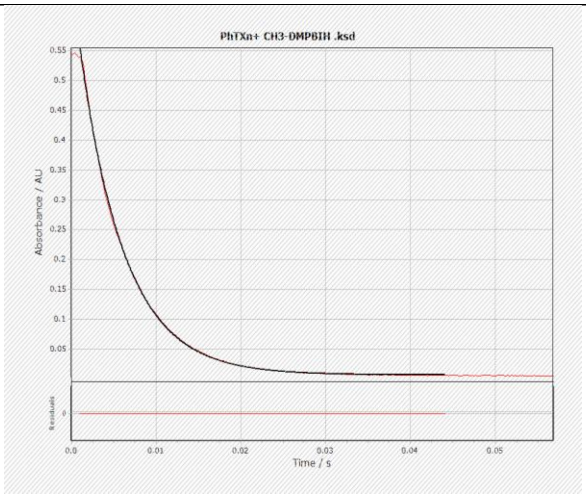
</td></tr>
<tr><td colspan="2">DMPBIH</td></tr>
<tr><td>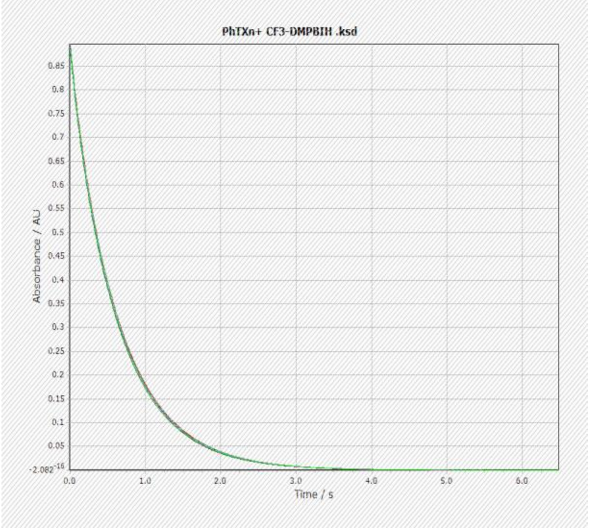
</td><td>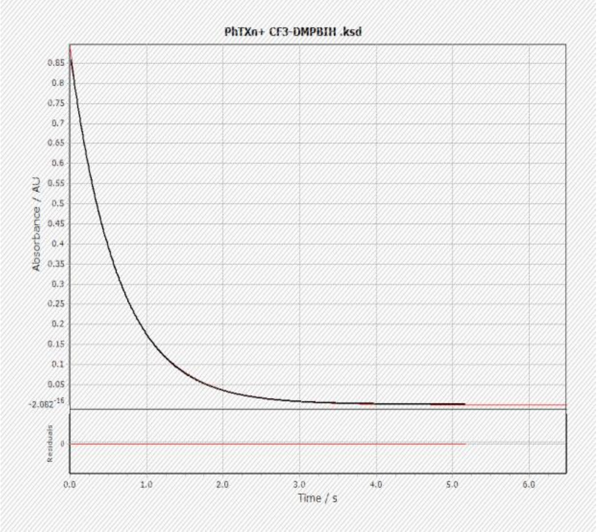
</td></tr>
<tr><td colspan="2">CF₃DMPBIH</td></tr>
</table>

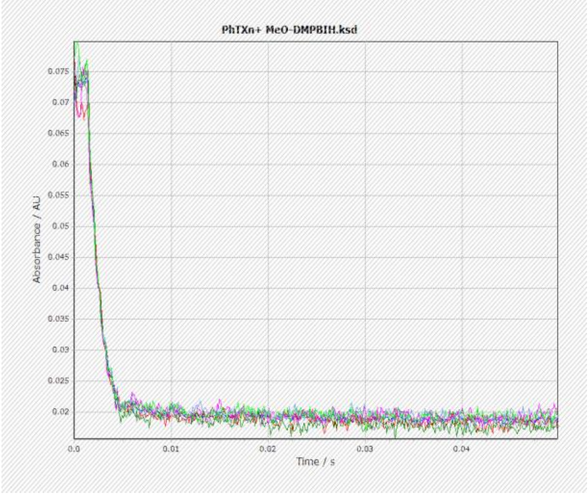

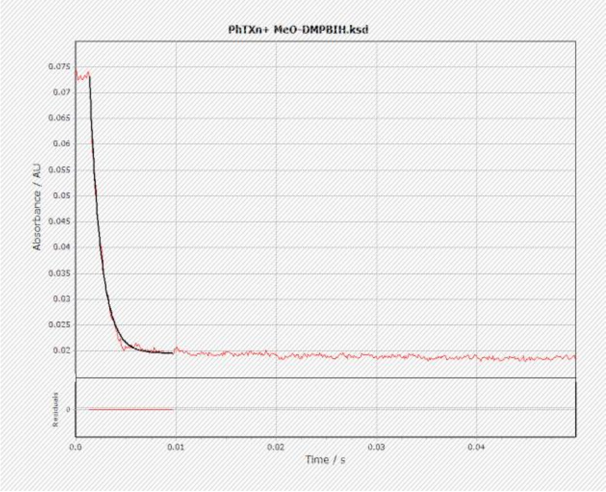

*MeO*DMPBIH

Day 2 data (2) (September 22, 2023)

	DMPBIH		*CF₃*DMPBIH		*CH₃O*DMPBIH [a]	
Runs	k^{pfo} (s^{-1})	k_{2H} ($M^{-1}s^{-1}$)	k^{pfo} (s^{-1})	k_{2H} ($M^{-1}s^{-1}$)	k^{pfo} (s^{-1})	k_{2H} ($M^{-1}s^{-1}$)
1	197.596		1.615		863.937	
2	193.782		1.615		836.371	
3	194.498		1.614		878.178	
4	193.778		1.626		903.117	
5	191.390		1.615		852.231	
6	194.450		1.617		951.323	
Average	194.249	159351.161	1.617	1326.365	880.859	722608.147
Stdev	1.170	960.167 **[b]**	0.004	3.682 **[b]**	40.543	33259.593 **[b]**

[a] The rate constants are less reliable as they have reached the limit of rate measurement from our stopped-flow instrument (Use of the data for discussion has been limited, see the main paper.); [b] = (Stdev(for k^{pfo})/k^{pfo})*k_{2H}

Six kinetic runs together with the averaged data	First-order kinetic fit of the averaged data as an example

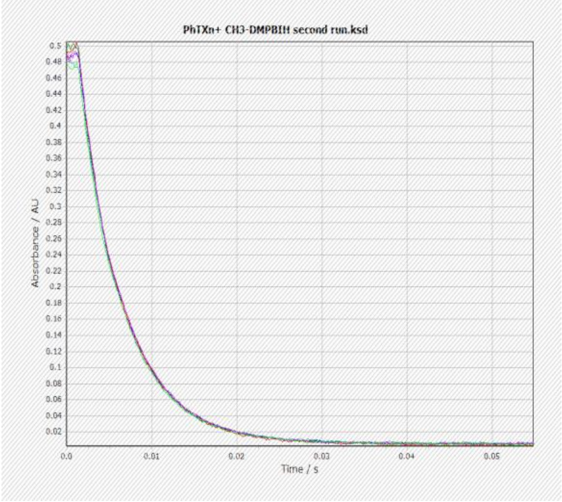

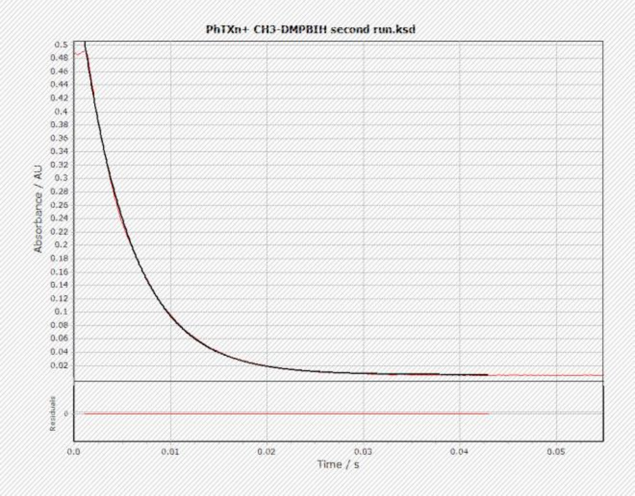

DMPBIH

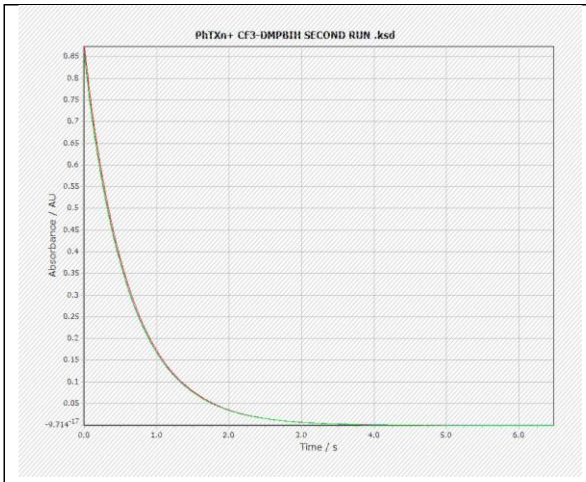

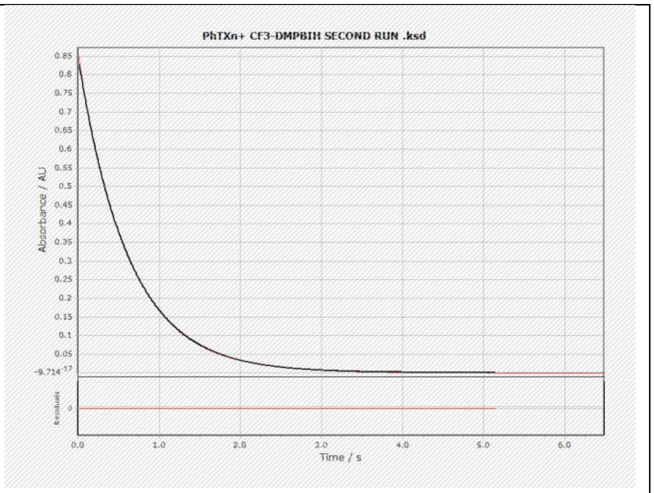

*CF3*DMPBIH

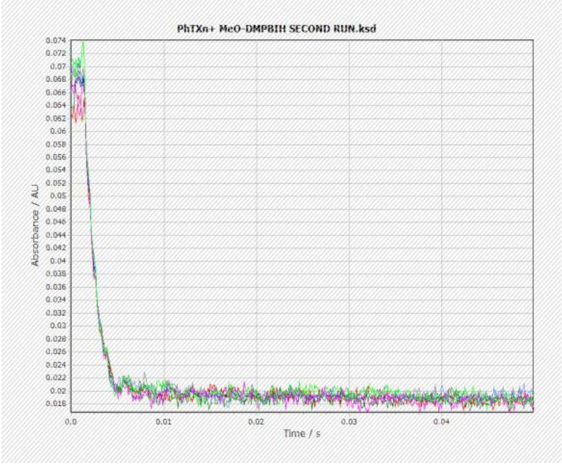

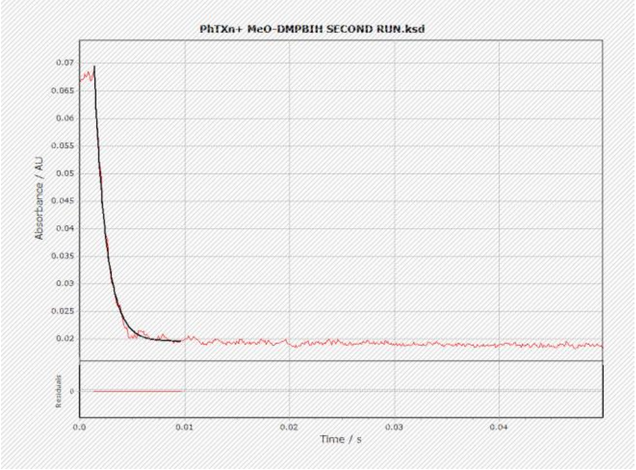

*MeO*DMPBIH

Primary kinetic data for the rate constants in Table S5

Day 1 data (July 28, 2020)

	Pseudo-first-order rate constants k^{pfo} (s^{-1})									
**Temp (°C)**	**Trial H1**	**Trial H2**	**Trial H3**	**Trial H4**	**Trial H5**	**Trial H6**	**Average k_H^{pfo} (s^{-1})**	**Stdev**	**k_{2H} (M^{-1}s^{-1})**	**Stdev [a]**
45	48.25704	49.04064	49.00741	49.01337	48.88739	49.2227	48.90476	0.335179	1.19E+05	812.55550
35	41.53487	42.51127	42.61632	42.92329	42.94155	42.97815	42.58424	0.548693	1.03E+05	1330.16552
25	36.32623	36.6378	36.38356	36.77485	36.70324	36.78161	36.60122	0.198711	8.87E+04	481.72263
15	31.09661	31.11006	31.33483	31.22849	30.80513	30.79999	31.06252	0.219223	7.53E+04	531.44854
5	26.03477	25.9342	25.58274	25.82582	25.96735	25.67555	25.83674	0.176856	6.26E+04	428.74256

**Temp (°C)**	**Trial D1**	**Trial D2**	**Trial D3**	**Trial D4**	**Trial D5**	**Trial D6**	**Average k_D^{pfo} (s^{-1})**	**Stdev**	**k_{2D} (M^{-1}s^{-1})**	**Stdev [a]**
45	14.90862	15.51975	15.56668	15.44073	15.31305	15.32097	15.34497	0.23701	3.65E+04	563.95415
35	12.79796	13.05351	13.09112	13.08142	12.94692	12.94752	12.98641	0.11232	3.09E+04	267.25949
25	10.65499	10.61251	10.61343	10.67043	10.53367	10.54304	10.60468	0.056274	2.52E+04	133.90166

15	8.59015	8.58418	8.6054	8.61483	8.61227	8.59177	8.59977	0.012762	2.05E+04	30.36653
5	6.71077	6.61944	6.64199	6.62406	6.61807	6.58252	6.63281	0.042811	1.58E+04	101.86547

[a] = (Stdev(for k^{pfo})/k^{pfo})*k_{2H}

Six kinetic runs together with the averaged data	First-order kinetic fit of the averaged data as an example

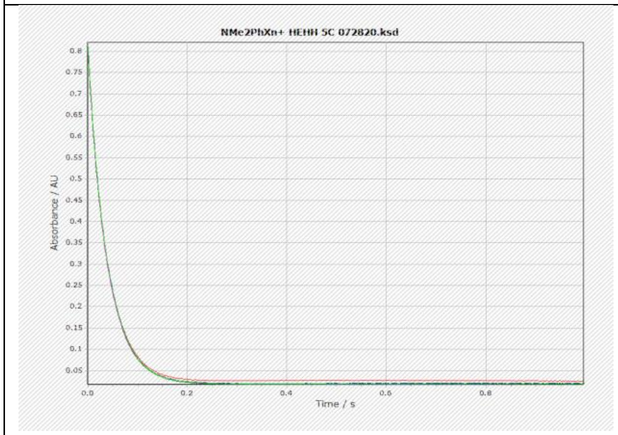

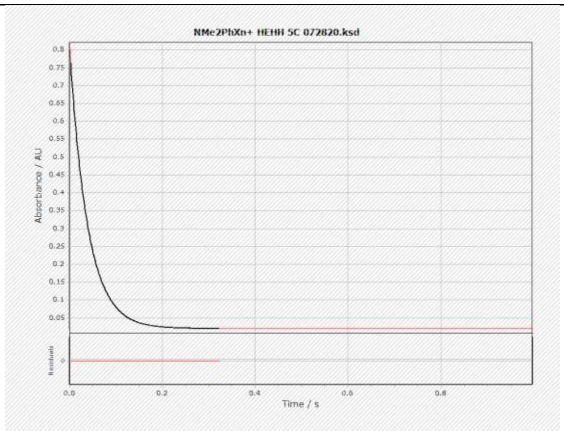

5℃ H

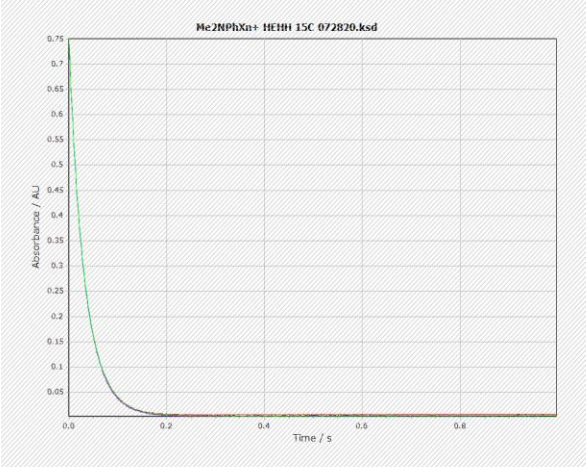

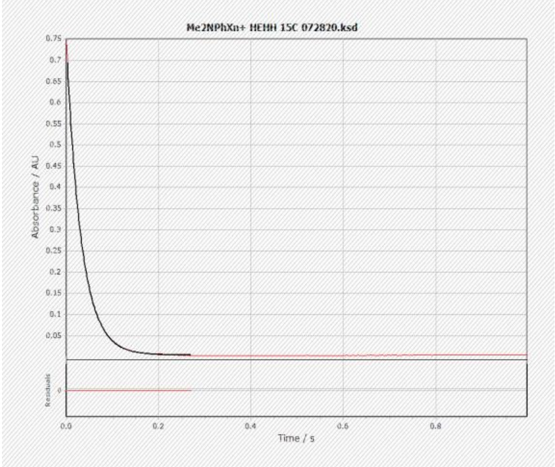

15℃ H

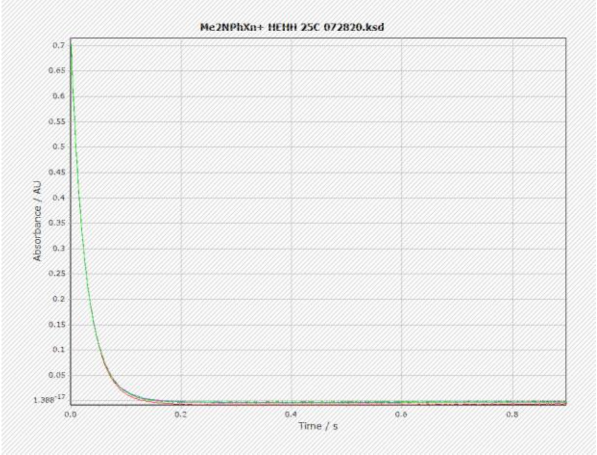

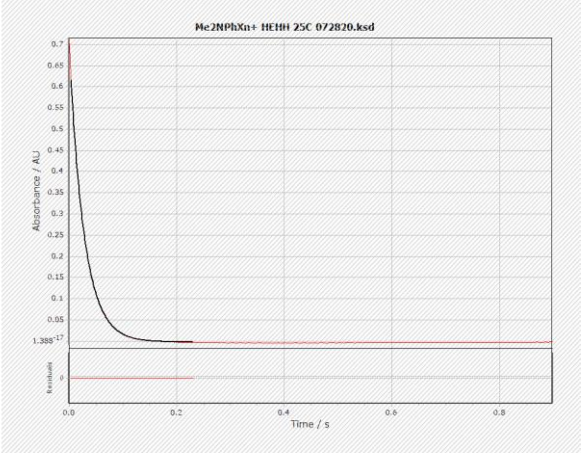

25℃ H

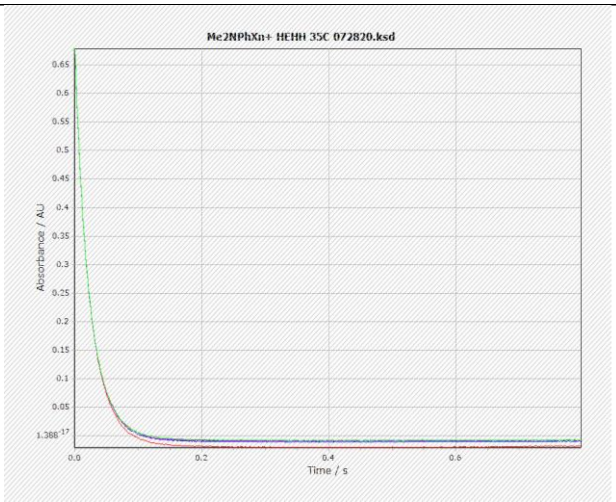

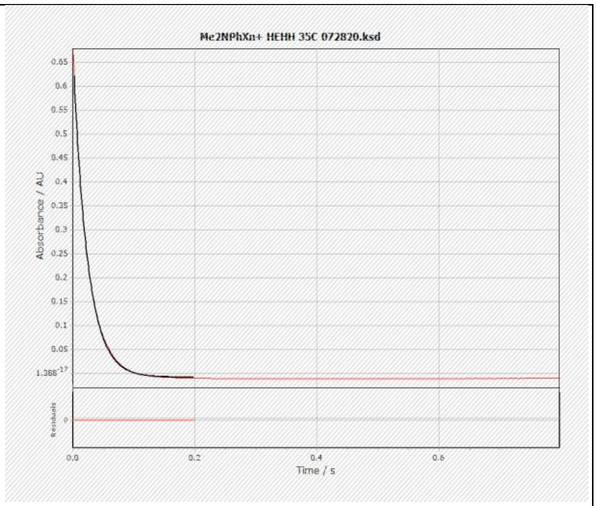

35°C H

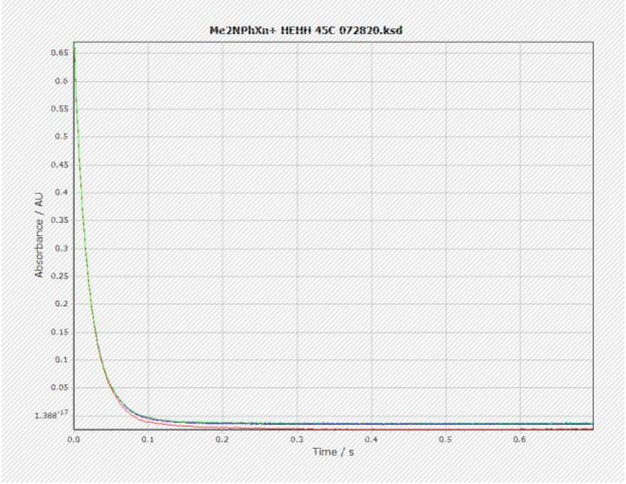

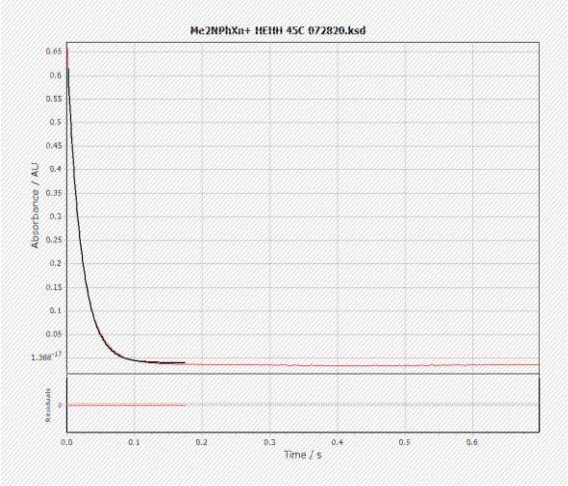

45°C H

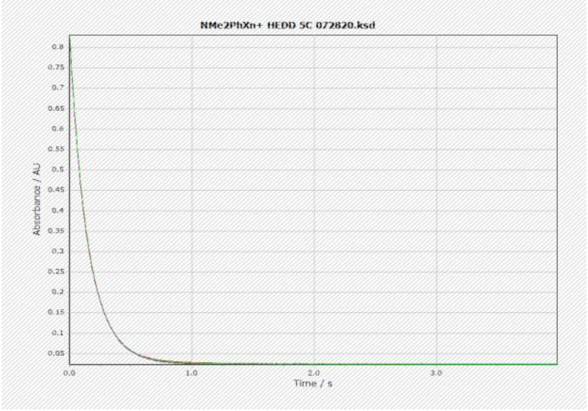

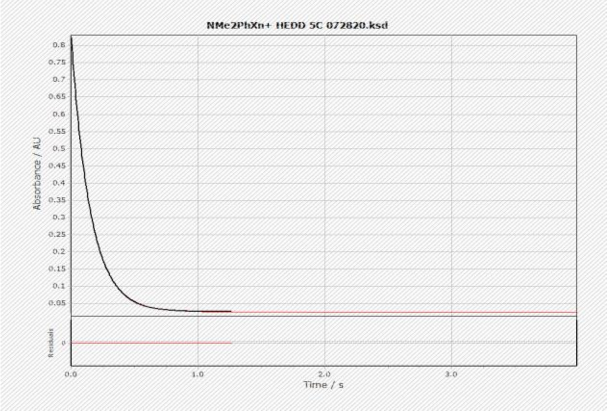

5°C D

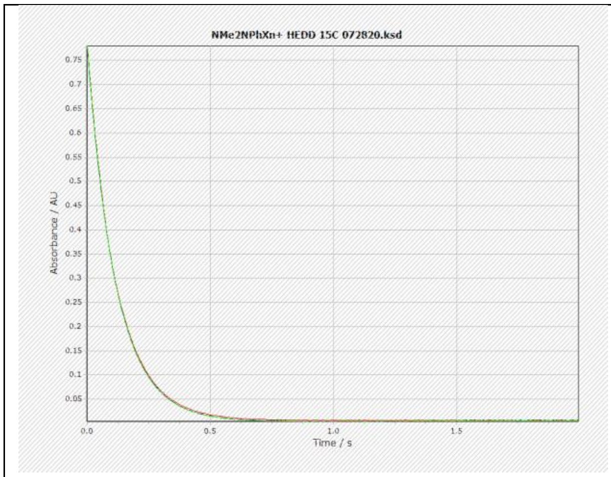
NMe2NPhXn+ HEDD 15C 072820.ksd
Absorbance / AU
Time / s

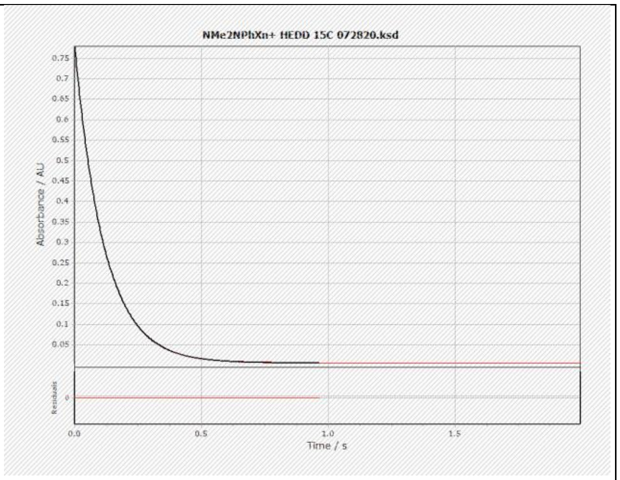
NMe2NPhXn+ HEDD 15C 072820.ksd
Absorbance / AU
Residuals
Time / s

15℃ D

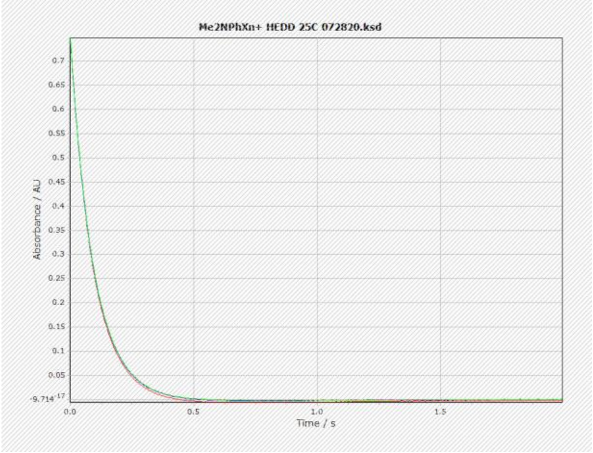
Me2NPhXn+ HEDD 25C 072820.ksd
Absorbance / AU
Time / s

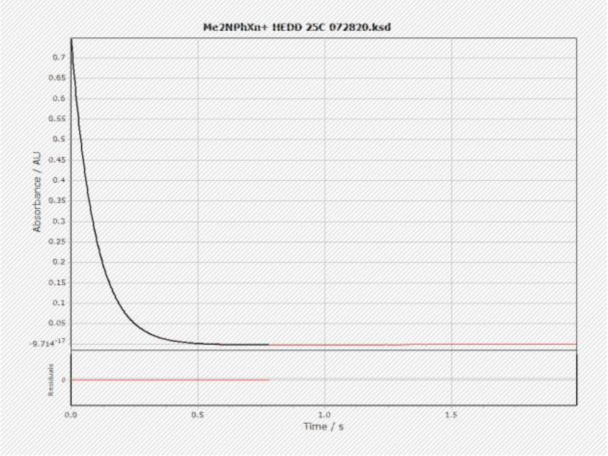
Me2NPhXn+ HEDD 25C 072820.ksd
Absorbance / AU
Residuals
Time / s

25℃ D

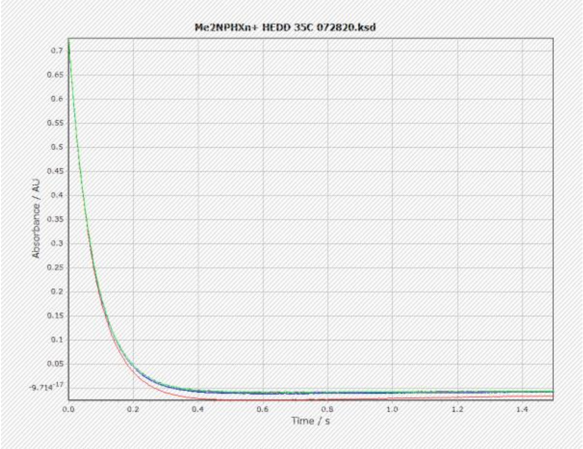
Me2NPHXn+ HEDD 35C 072820.ksd
Absorbance / AU
Time / s

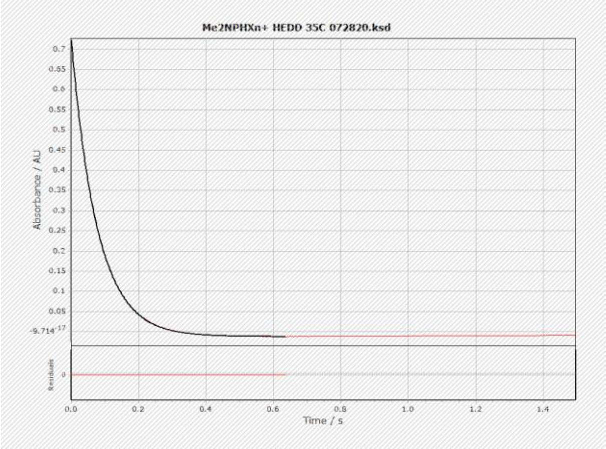
Me2NPHXn+ HEDD 35C 072820.ksd
Absorbance / AU
Residuals
Time / s

35℃ D

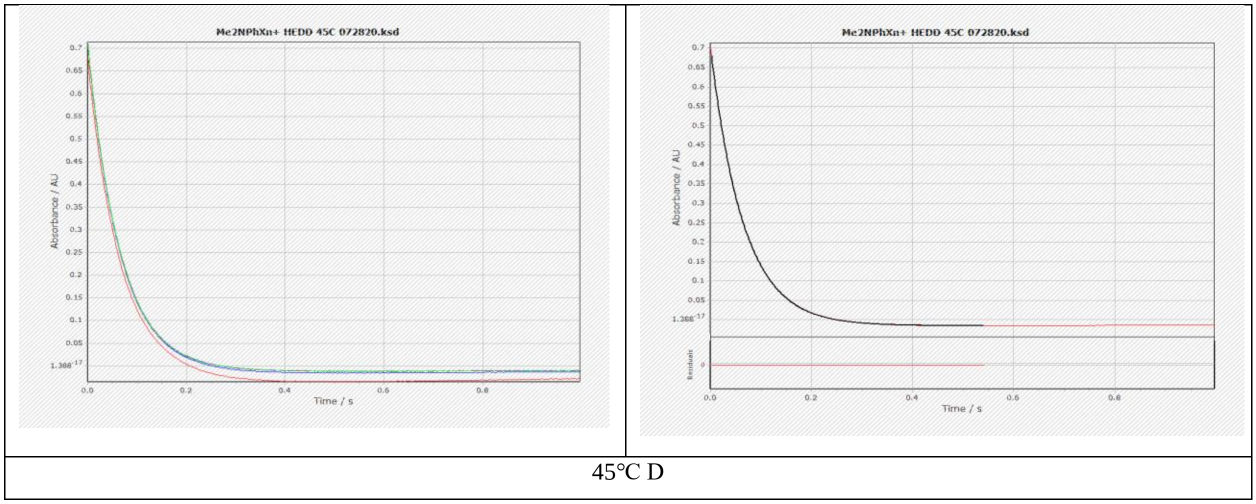

45°C D

Day 2 data (July 29, 2020)

Temp (°C)	Pseudo-first-order rate constants k^{pfo} (s^{-1}) Trial H1	Trial H2	Trial H3	Trial H4	Trial H5	Trial H6	**Average k_H^{pfo} (s^{-1})**	**Stdev**	**k_{2H} (M^{-1}s^{-1})**	**Stdev [a]**
45	45.83496	46.48853	46.40828	46.76407	47.32197	46.99912	46.63616	0.516543	1.13E+05	1252.22465
35	39.40389	40.2402	40.84949	40.82641	40.71829	40.44155	40.41331	0.548381	9.80E+04	1329.40941
25	34.47456	35.03873	34.86193	34.73297	35.00059	35.00516	34.85232	0.217776	8.45E+04	527.94205
15	29.3686	29.49025	29.11088	29.48054	29.51612	29.66313	29.43825	0.186091	7.14E+04	451.12987
5	24.8025	24.64166	24.57538	24.6848	24.34777	24.39445	24.57443	0.174634	5.96E+04	423.35602
**Temp (°C)**	**Trial D1**	**Trial D2**	**Trial D3**	**Trial D4**	**Trial D5**	**Trial D6**	**Average k_D^{pfo} (s^{-1})**	**Stdev**	**k_{2D} (M^{-1}s^{-1})**	**Stdev [a]**
45	14.12968	14.71581	14.76461	14.76461	14.81433	14.74801	14.65618	0.259891	3.42E+04	606.88269
35	12.36559	12.43345	12.45715	12.43589	12.37544	12.36605	12.40560	0.041049	2.90E+04	95.85554
25	10.14306	10.12765	10.1322	10.14847	10.1274	10.07367	10.12541	0.026741	2.36E+04	62.44401
15	8.1875	8.15538	8.12462	8.20977	8.09194	8.10549	8.14578	0.046685	1.90E+04	109.01506
5	6.38264	6.32863	6.35578	6.38406	6.35645	6.29454	6.35035	0.034153	1.48E+04	79.75274

[a] = (Stdev(for k^{pfo})/k^{pfo})*k_{2H}

<table>
<tr><td>Six kinetic runs together with the averaged data</td><td>First-order kinetic fit of the averaged data as an example</td></tr>
<tr><td>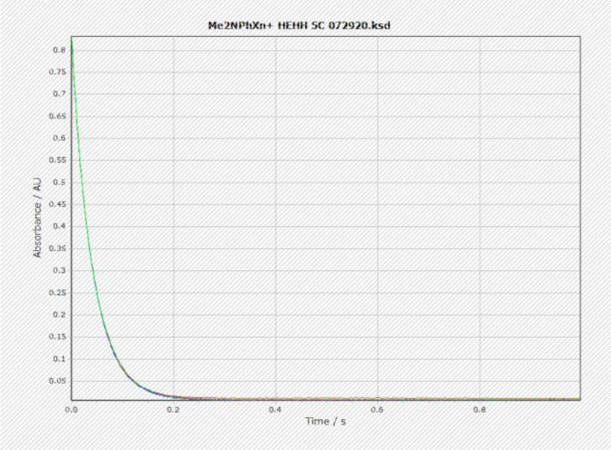
</td><td>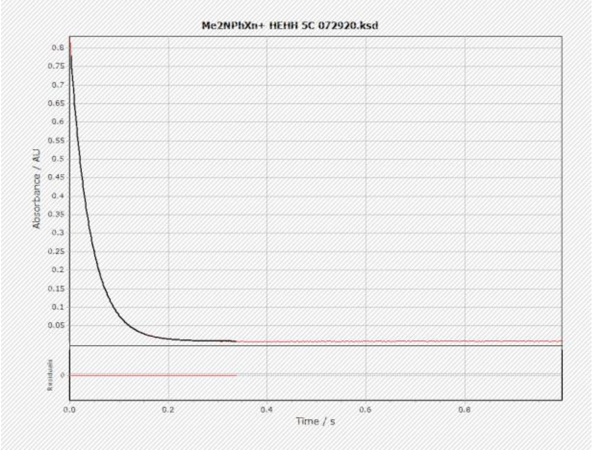
</td></tr>
<tr><td colspan="2">5℃ H</td></tr>
<tr><td>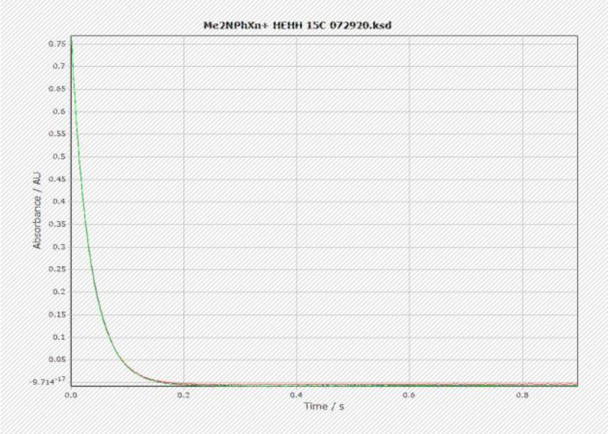
</td><td>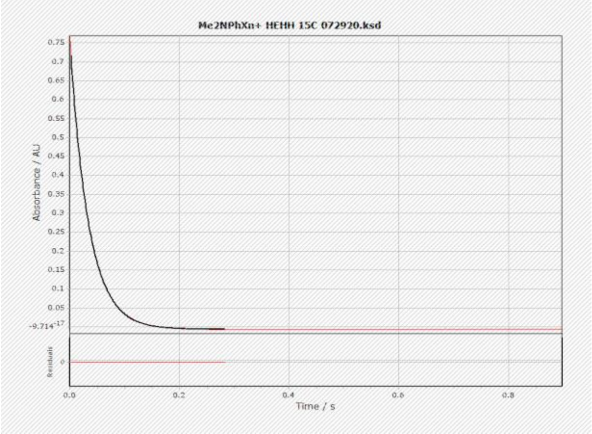
</td></tr>
<tr><td colspan="2">15℃ H</td></tr>
<tr><td>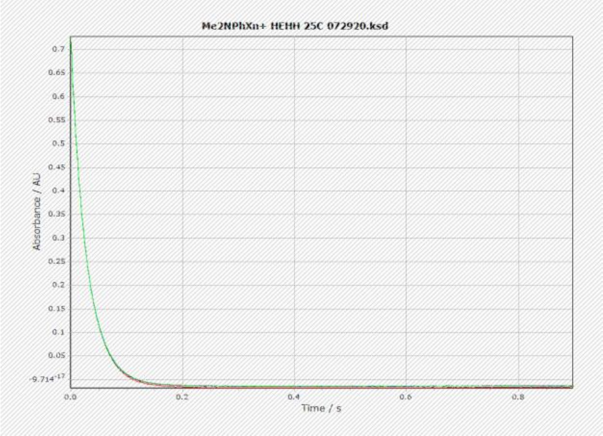
</td><td>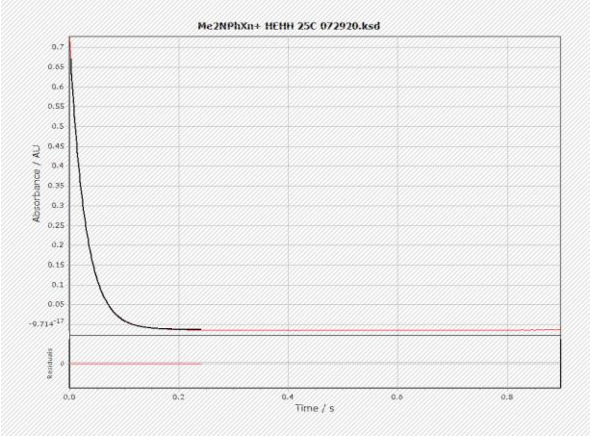
</td></tr>
<tr><td colspan="2">25℃ H</td></tr>
</table>

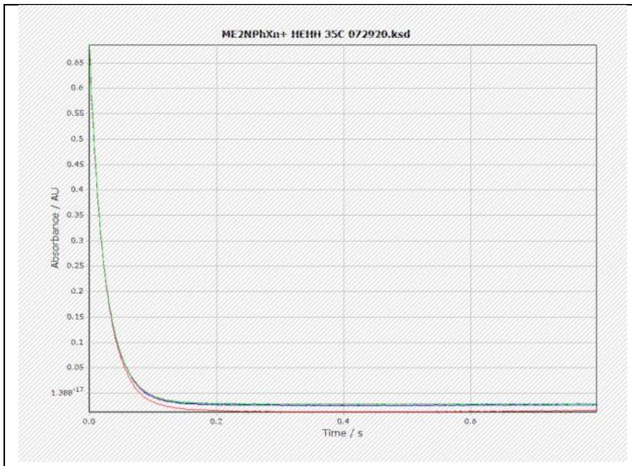

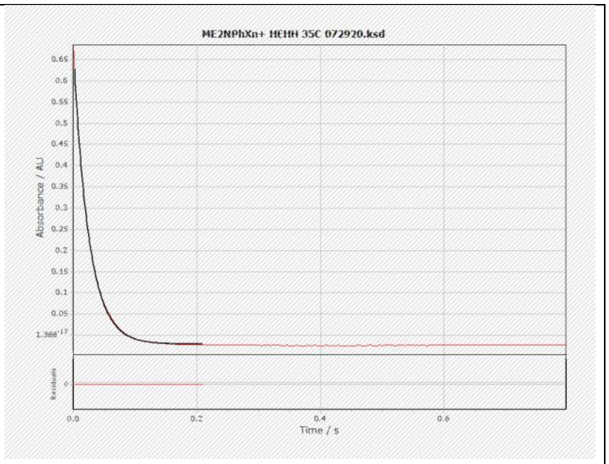

35°C H

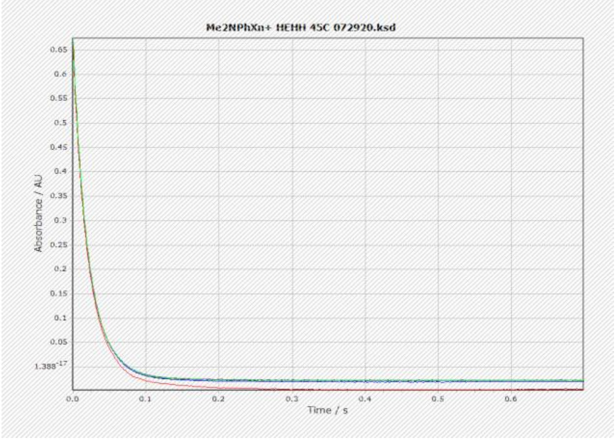

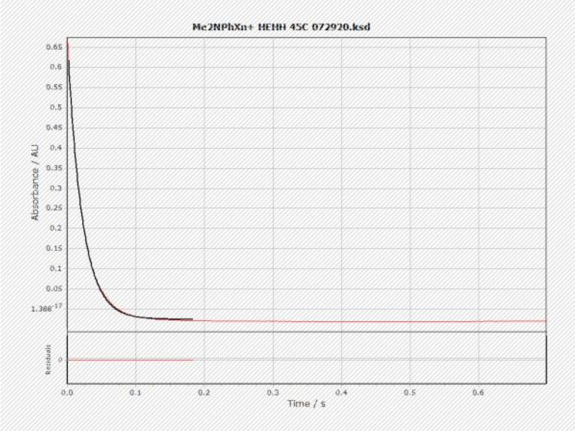

45°C H

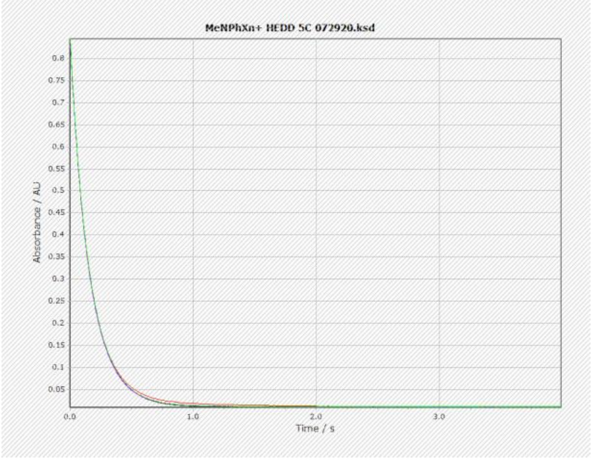

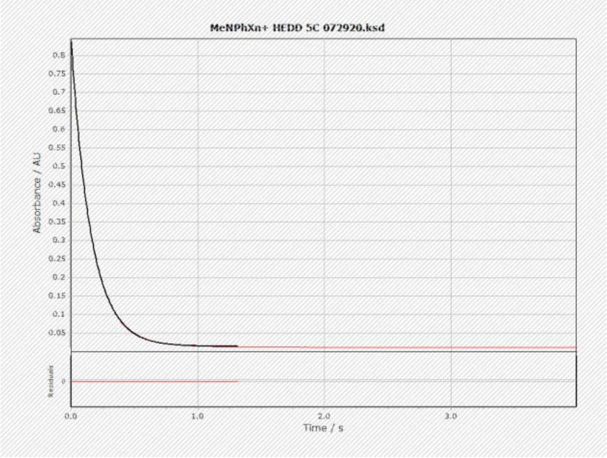

5°C D

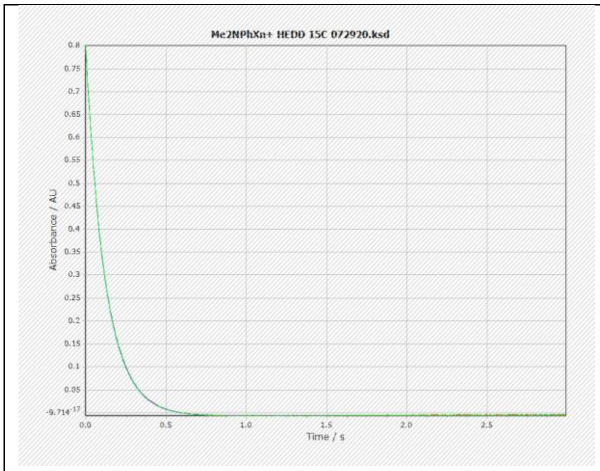
Me2NPhXn+ HEDD 15C 072920.ksd
Absorbance / AU
Time / s

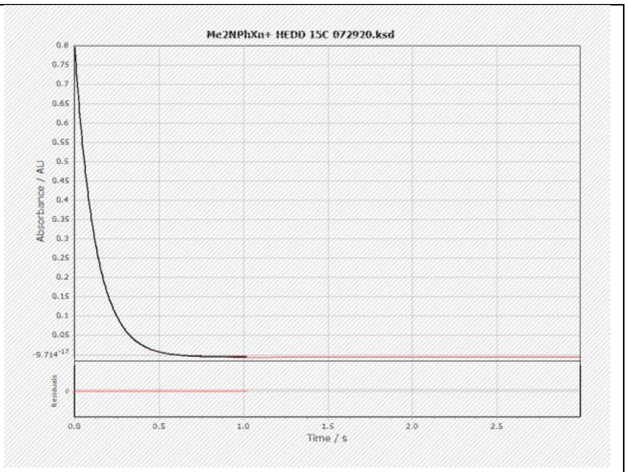
Me2NPhXn+ HEDD 15C 072920.ksd
Absorbance / AU
Residuals
Time / s

15℃ D

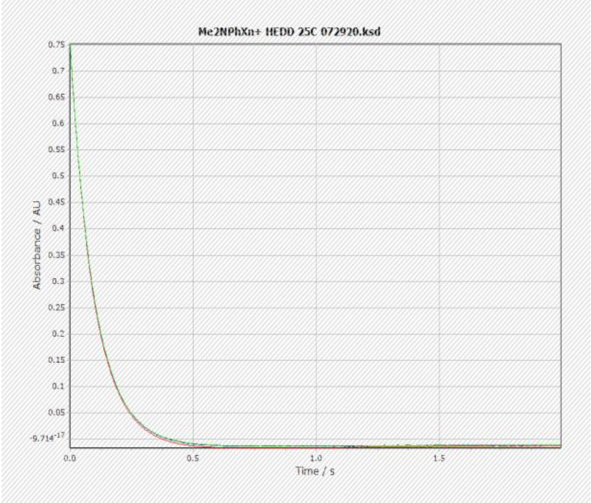
Me2NPhXn+ HEDD 25C 072920.ksd
Absorbance / AU
Time / s

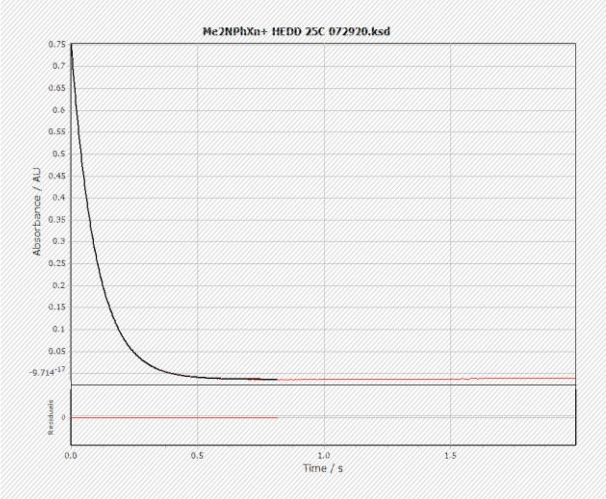
Me2NPhXn+ HEDD 25C 072920.ksd
Absorbance / AU
Residuals
Time / s

25℃ D

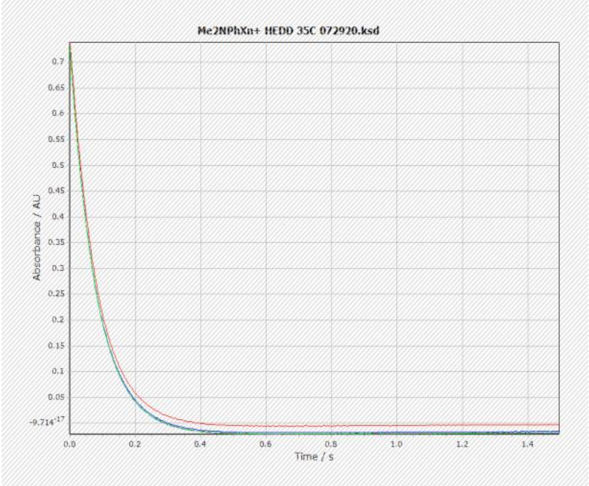
Me2NPhXn+ HEDD 35C 072920.ksd
Absorbance / AU
Time / s

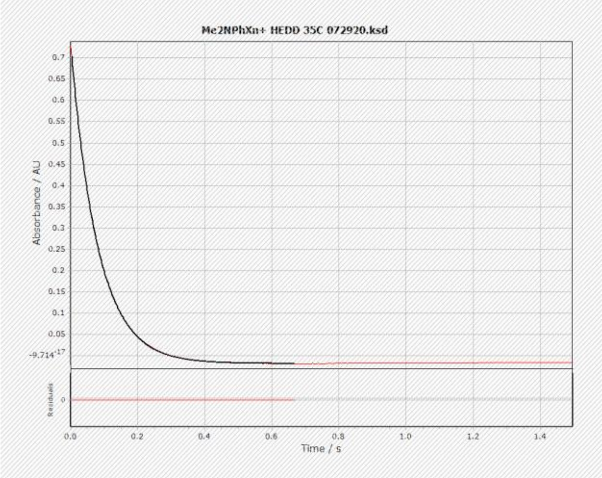
Me2NPhXn+ HEDD 35C 072920.ksd
Absorbance / AU
Residuals
Time / s

35℃ D

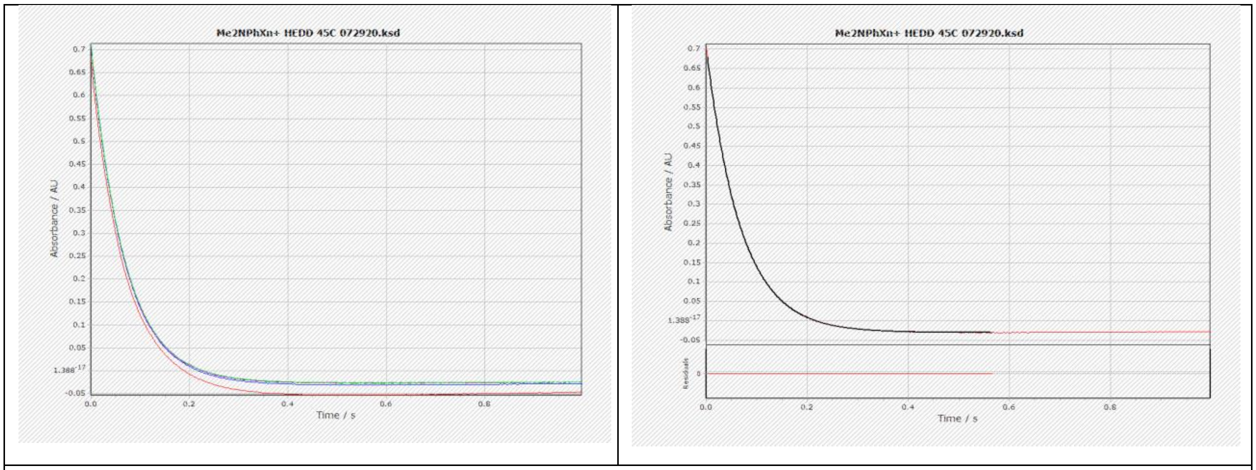

45°C D

Day 3 data (July 31, 2020)

**Temp (°C)**	Pseudo-first-order rate constants k^{pfo} (s^{-1}) **Trial H1**	**Trial H2**	**Trial H3**	**Trial H4**	**Trial H5**	**Trial H6**	**Average k_H^{pfo} (s^{-1})**	**Stdev**	**k_{2H} ($M^{-1}s^{-1}$)**	**Stdev [a]**
45	50.31484	51.37976	51.49387	52.00785	51.2815	51.83946	51.38621	0.593684	1.25E+05	1439.23334
35	44.88547	44.78339	45.65123	45.40857	45.02419	44.81617	45.09484	0.355382	1.09E+05	861.53199
25	37.92999	38.25722	38.36439	38.37835	38.46373	38.62355	38.33621	0.233516	9.29E+04	566.10045
15	32.27856	32.17676	32.20495	32.48295	32.27038	32.2487	32.27705	0.108168	7.82E+04	262.22594
5	27.72149	26.66238	26.97146	27.03196	26.74188	26.86614	26.99922	0.379714	6.55E+04	920.51765
**Temp (°C)**	**Trial D1**	**Trial D2**	**Trial D3**	**Trial D4**	**Trial D5**	**Trial D6**	**Average k_D^{pfo} (s^{-1})**	**Stdev**	**k_{2D} ($M^{-1}s^{-1}$)**	**Stdev [a]**
45	15.11299	15.79634	15.65128	15.73021	15.61332	15.77592	15.61334	0.255096	3.77E+04	615.30045
35	13.19684	13.18218	13.13371	13.22759	13.24699	13.21735	13.20078	0.039971	3.18E+04	96.41253
25	10.7801	10.77896	10.76105	10.74225	10.71565	10.74782	10.75431	0.024496	2.59E+04	59.08587
15	8.61562	8.56174	8.61576	8.58399	8.54518	8.61207	8.58906	0.030481	2.07E+04	73.52024
5	6.85271	6.74832	6.72994	6.74756	6.82806	6.82806	6.78911	0.052859	1.64E+04	127.49834

[a] = (Stdev(for k^{pfo})/k^{pfo})*k_{2H}

<table>
<tr><td>Six kinetic runs together with the averaged data</td><td>First-order kinetic fit of the averaged data as an example</td></tr>
<tr><td>
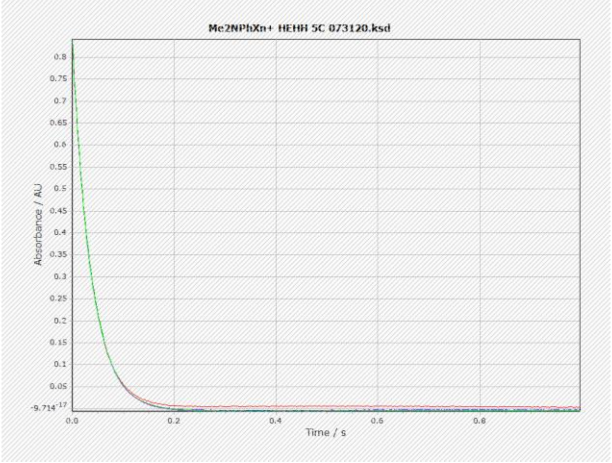

</td><td>
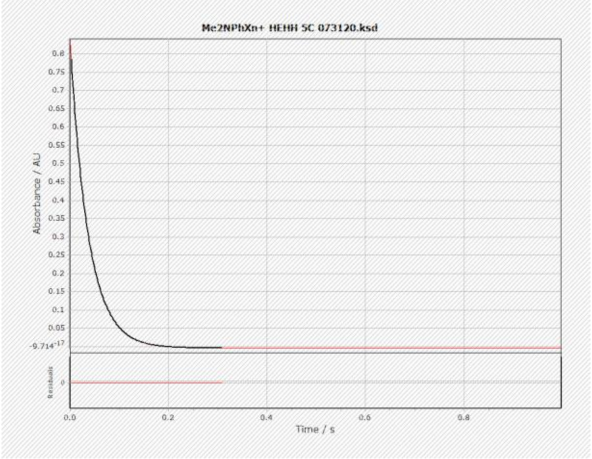

</td></tr>
<tr><td colspan="2">5℃ H</td></tr>
<tr><td>
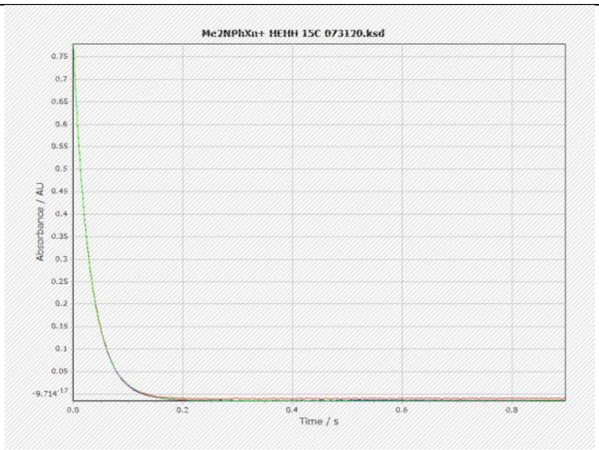

</td><td>
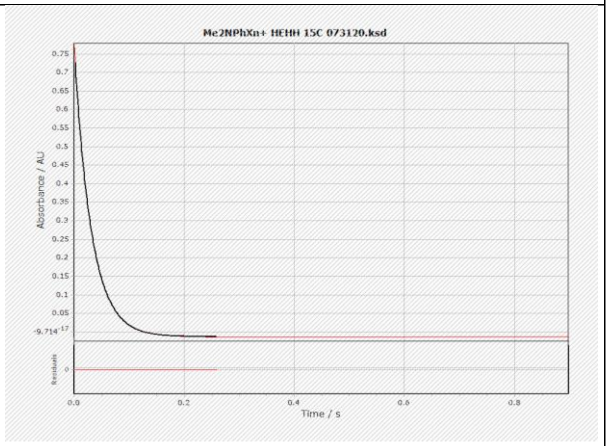

</td></tr>
<tr><td colspan="2">15℃ H</td></tr>
<tr><td>
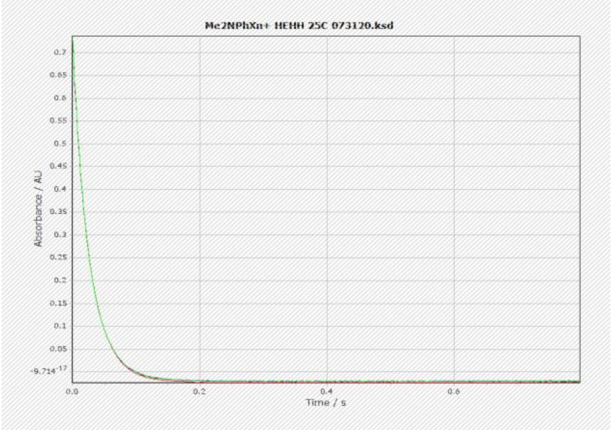

</td><td>
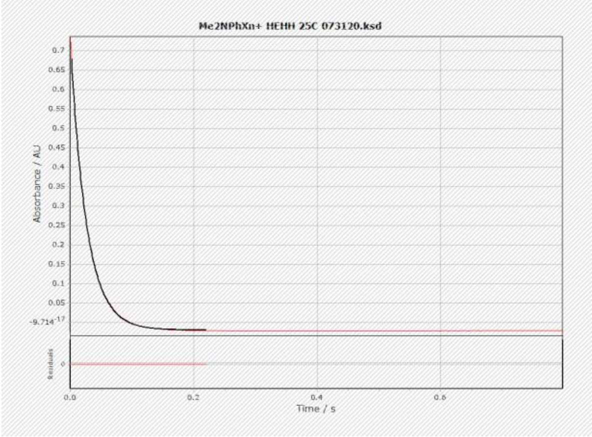

</td></tr>
<tr><td colspan="2">25℃ H</td></tr>
</table>

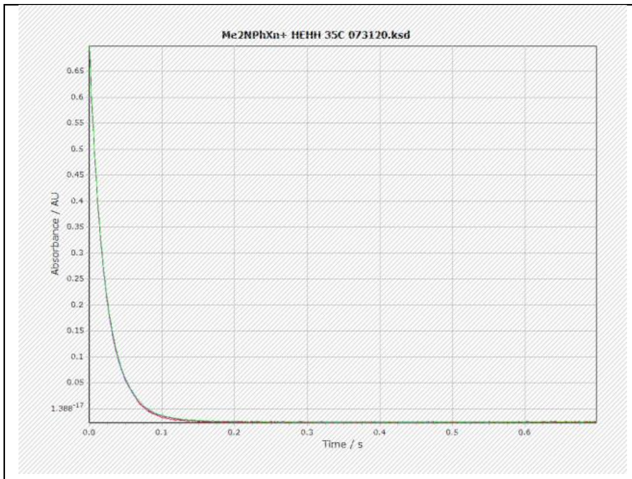

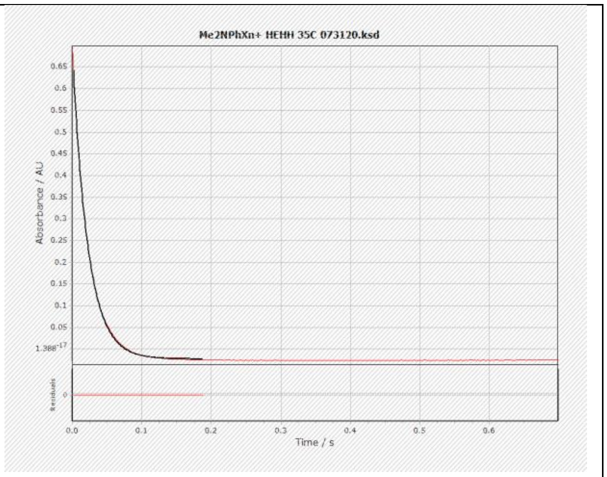

35°C H

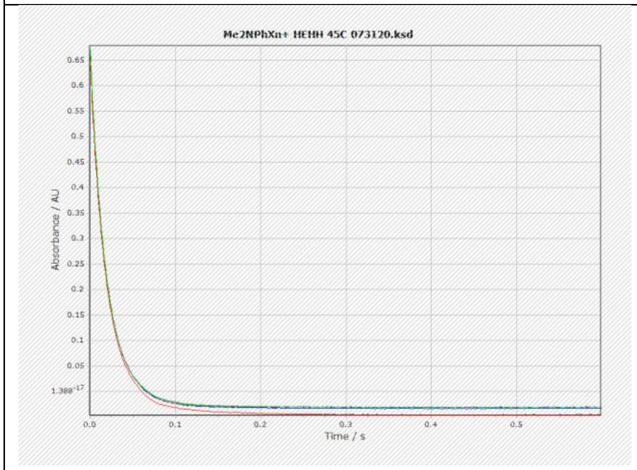

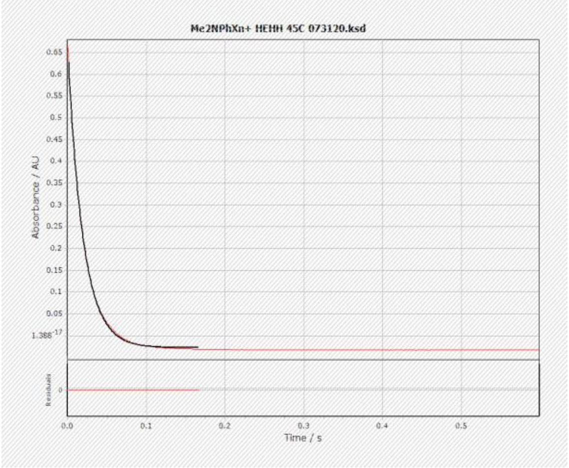

45°C H

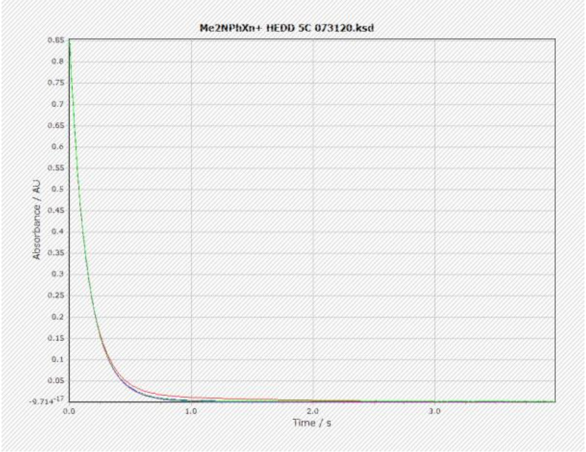

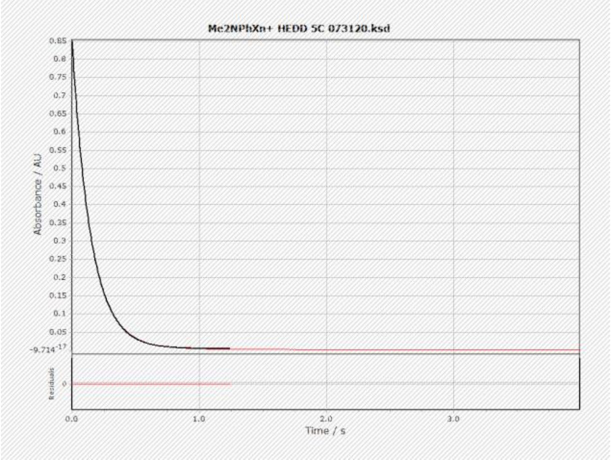

5°C D

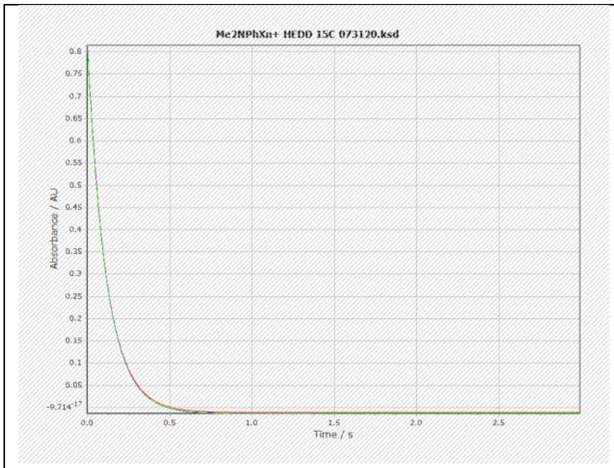
Me2NPhXn+ HEDD 15C 073120.ksd
Absorbance / AU
Time / s

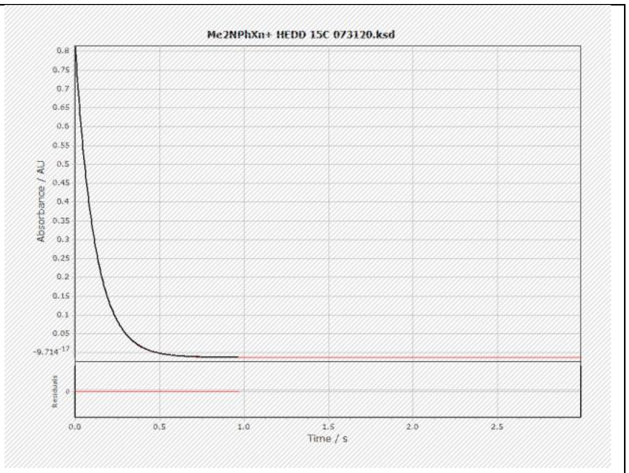
Me2NPhXn+ HEDD 15C 073120.ksd
Absorbance / AU
Residuals
Time / s

15℃ D

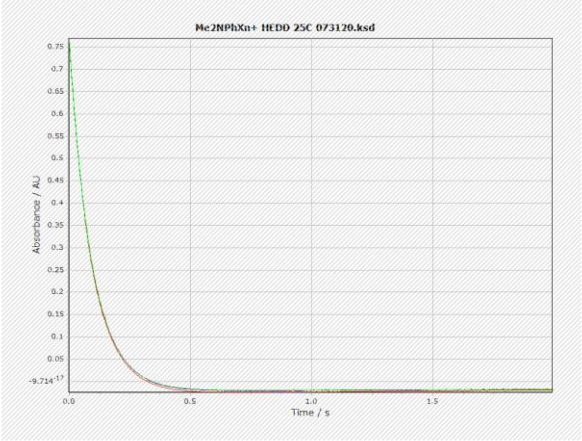
Me2NPhXn+ HEDD 25C 073120.ksd
Absorbance / AU
Time / s

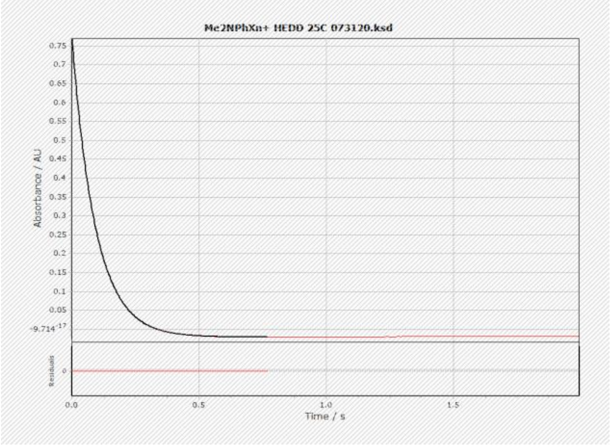
Me2NPhXn+ HEDD 25C 073120.ksd
Absorbance / AU
Residuals
Time / s

25℃ D

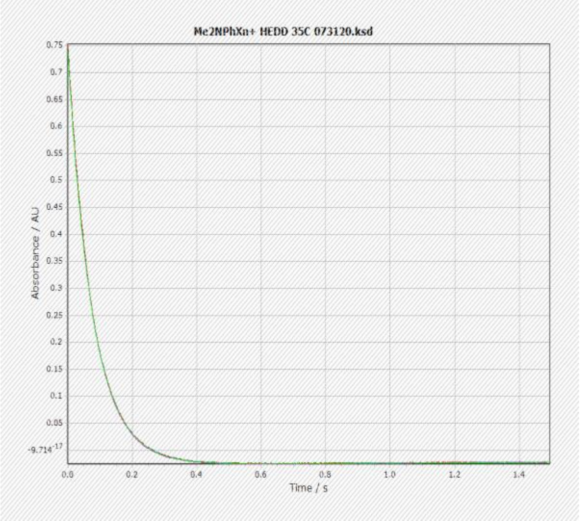
Me2NPhXn+ HEDD 35C 073120.ksd
Absorbance / AU
Time / s

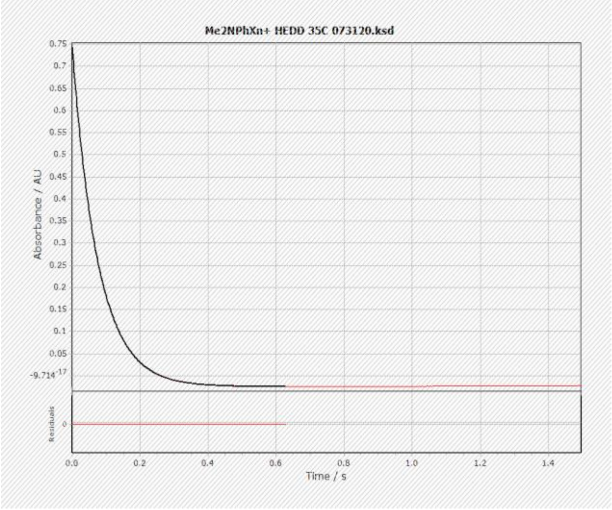
Me2NPhXn+ HEDD 35C 073120.ksd
Absorbance / AU
Residuals
Time / s

35℃ D

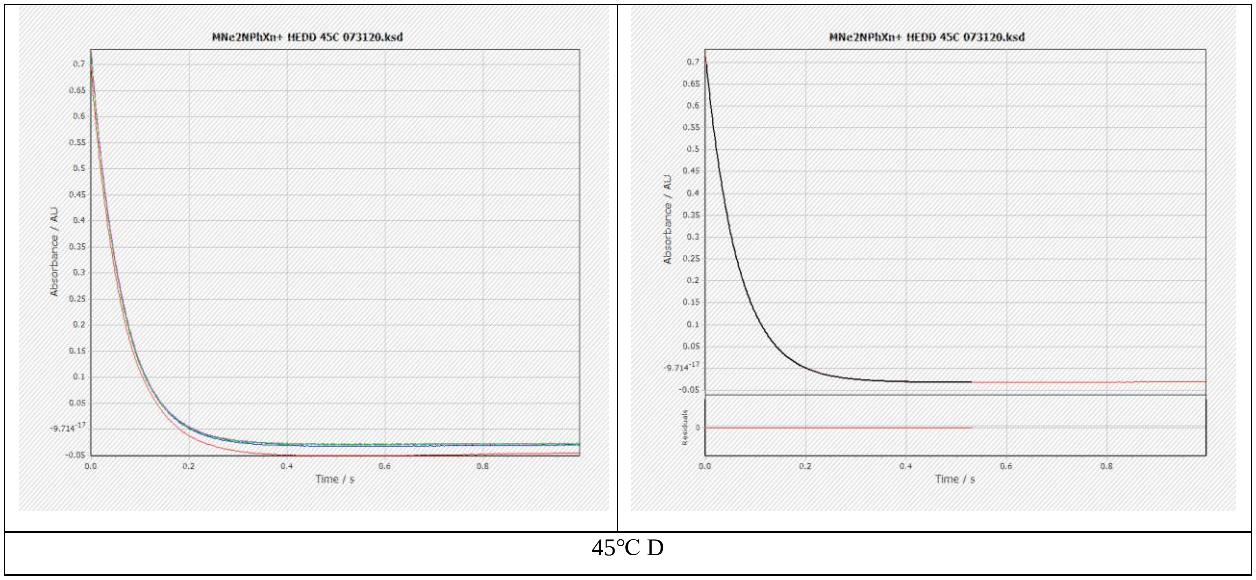

45°C D

Primary kinetic data for the rate constants in Table S6

Day 1 data (September 10, 2021)

Temp (°C)	Pseudo-first-order rate constants k^{pfo} (s^{-1}) Trial H1	Trial H2	Trial H3	Trial H4	Trial H5	Trial H6	Average k_H^{pfo} (s^{-1})	Stdev	k_{2H} ($M^{-1}s^{-1}$)	Stdev [a]
55	0.07865	0.07846	0.07921	0.07890	0.07885	0.07898	0.07884	0.00026	13.14028	0.04351
45	0.05532	0.05517	0.05544	0.05561	0.05544	0.05634	0.05555	0.00041	9.25889	0.06868
35	0.03841	0.03839	0.03852	0.03855	0.03841	0.03853	0.03847	0.00007	6.41139	0.01204
25	0.02522	0.02517	0.02502	0.02467	0.02513	0.02507	0.02505	0.00020	4.17444	0.03294
15	0.01592	0.01593	0.01561	0.01571	0.01584	0.01593	0.01582	0.00013	2.63722	0.02245
**Temp (°C)**	**Trial D1**	**Trial D2**	**Trial D3**	**Trial D4**	**Trial D5**	**Trial D6**	**Average k_D^{pfo} (s^{-1})**	**Stdev**	**k_{2D} ($M^{-1}s^{-1}$)**	**Stdev [a]**
55	0.01851	0.01858	0.01849	0.01837	0.01854	0.01860	0.01852	0.00008	3.08583	0.01369
45	0.01220	0.01240	0.01235	0.01247	0.01245	0.01245	0.01239	0.00010	2.06444	0.01689
35	0.00798	0.00790	0.00789	0.00791	0.00794	0.00796	0.00793	0.00004	1.32167	0.00596
25	0.00493	0.00495	0.00498	0.00491	0.00506	0.00491	0.00496	0.00006	0.82611	0.00953
15	0.00282	0.00283	0.00290	0.00287	0.00281	0.00283	0.00284	0.00003	0.47389	0.00574

[a] = (Stdev(for k^{pfo})/k^{pfo})*k_{2H}

<table>
<tr><td>Six kinetic runs together with the averaged data</td><td>First-order kinetic fit of the averaged data as an example</td></tr>
<tr><td>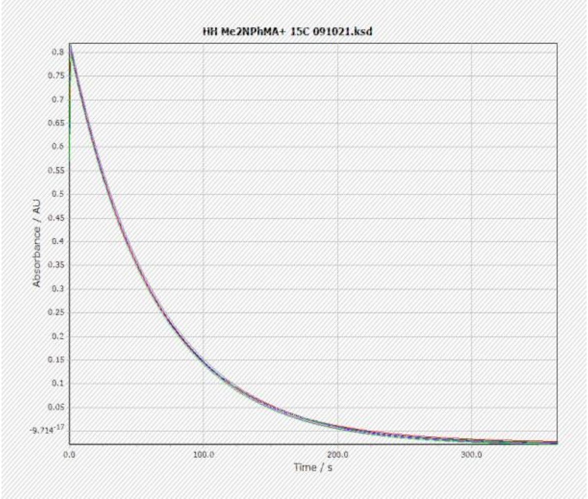
</td><td>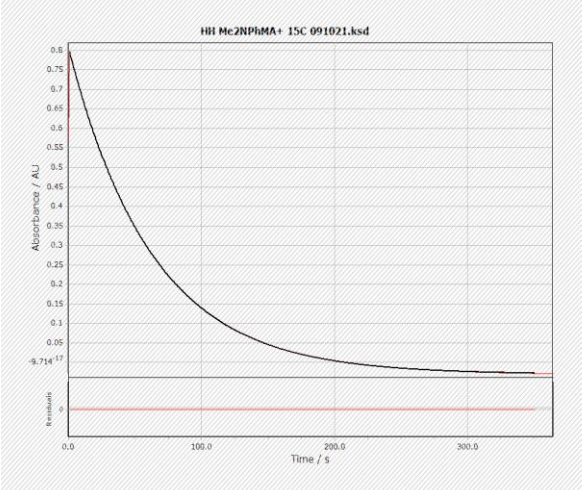
</td></tr>
<tr><td colspan="2">15℃ H</td></tr>
<tr><td>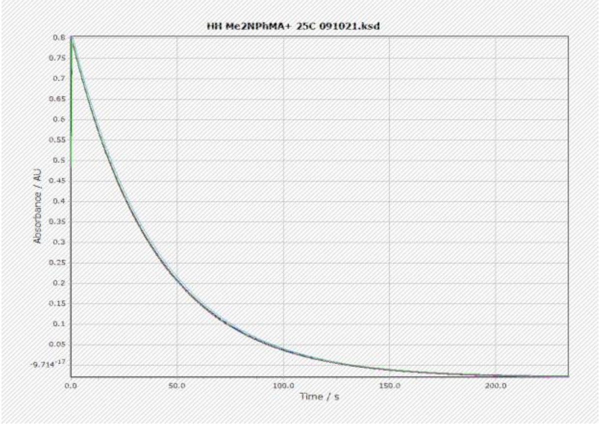
</td><td>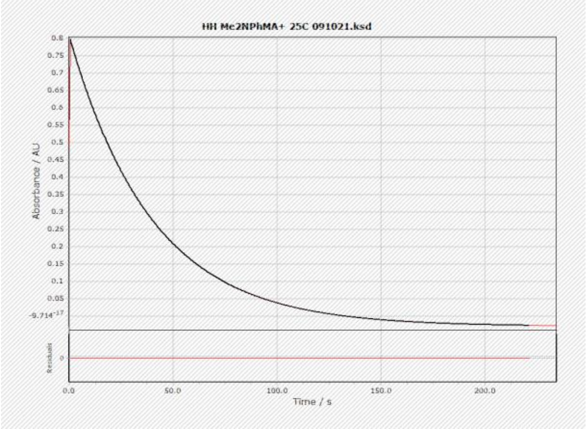
</td></tr>
<tr><td colspan="2">25℃ H</td></tr>
<tr><td>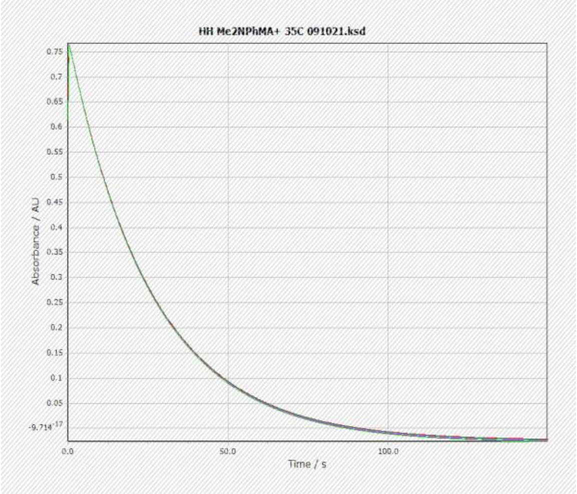
</td><td>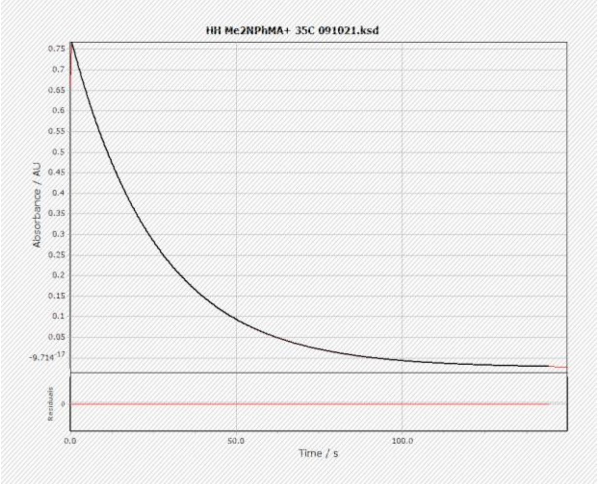
</td></tr>
<tr><td colspan="2">35℃ H</td></tr>
</table>

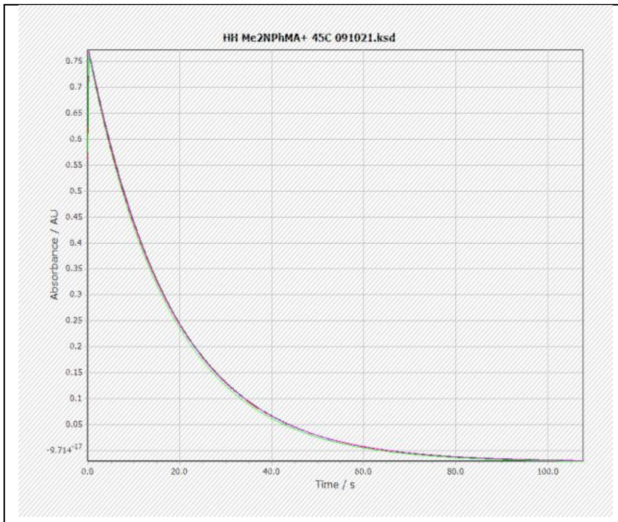
HH Me2NPhMA+ 45C 091021.ksd
Absorbance / AU
Time / s

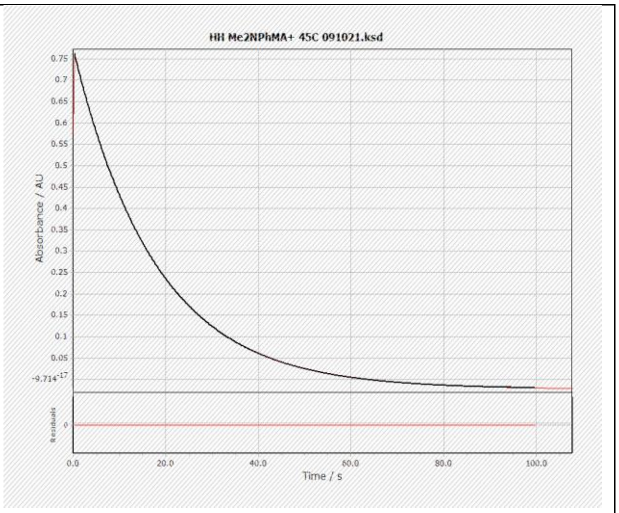
HH Me2NPhMA+ 45C 091021.ksd
Absorbance / AU
Residuals
Time / s

45℃ H

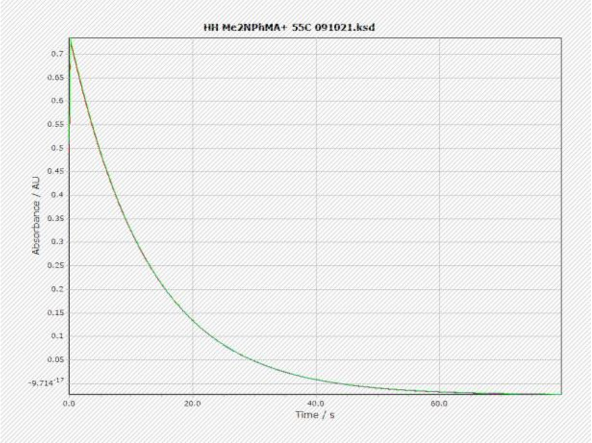
HH Me2NPhMA+ 55C 091021.ksd
Absorbance / AU
Time / s

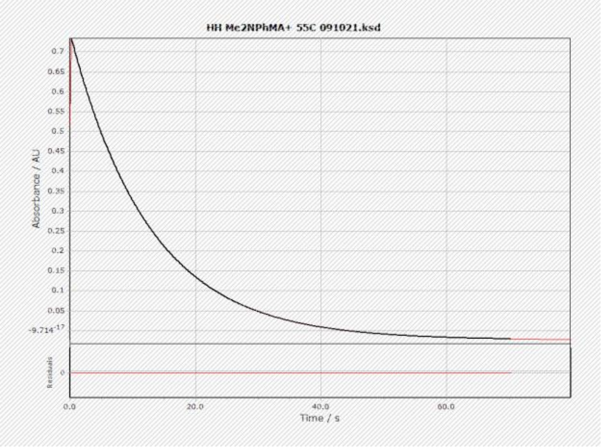
HH Me2NPhMA+ 55C 091021.ksd
Absorbance / AU
Residuals
Time / s

55℃ H

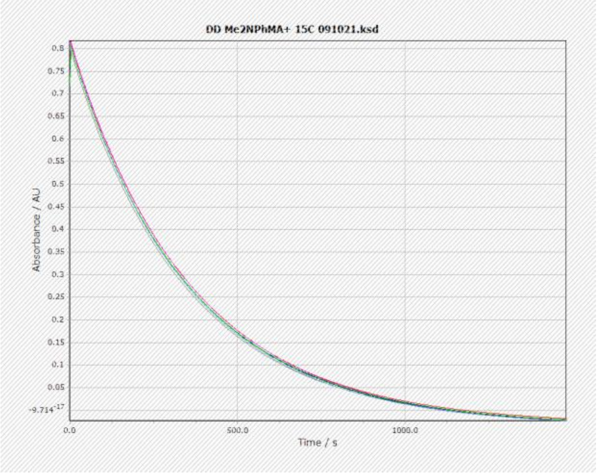
DD Me2NPhMA+ 15C 091021.ksd
Absorbance / AU
Time / s

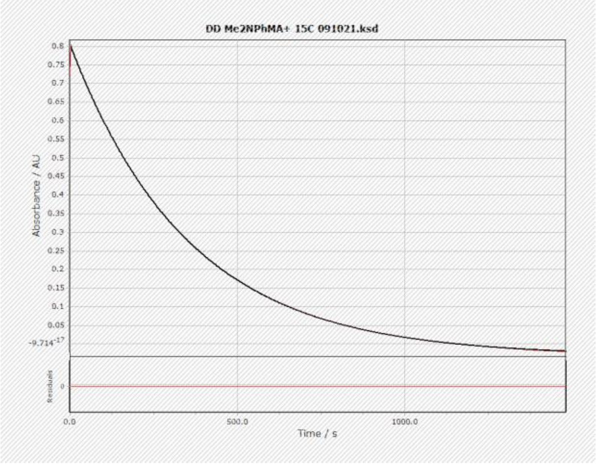
DD Me2NPhMA+ 15C 091021.ksd
Absorbance / AU
Residuals
Time / s

15℃ D

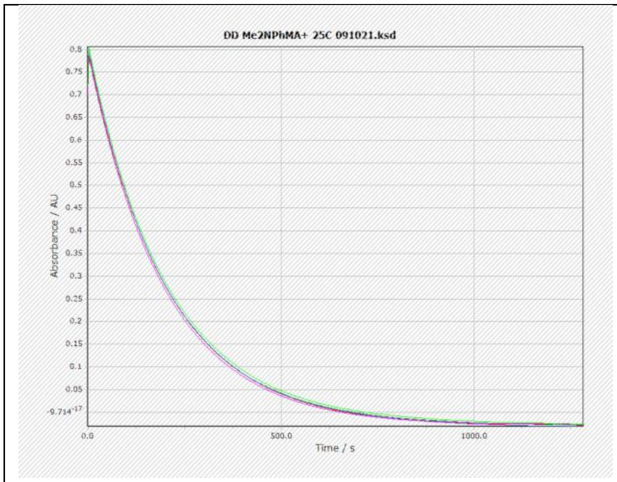

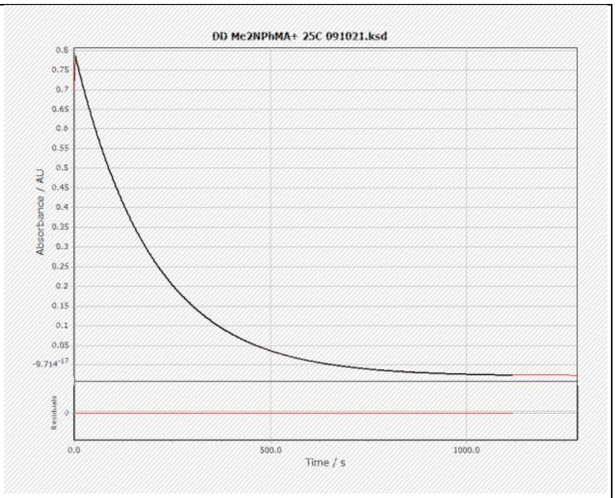

25°C D

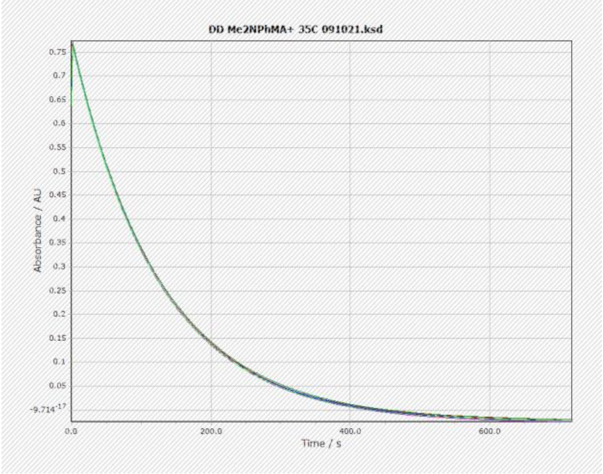

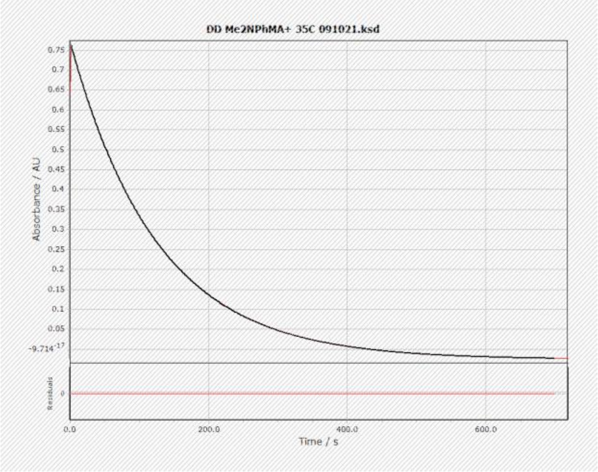

35°C D

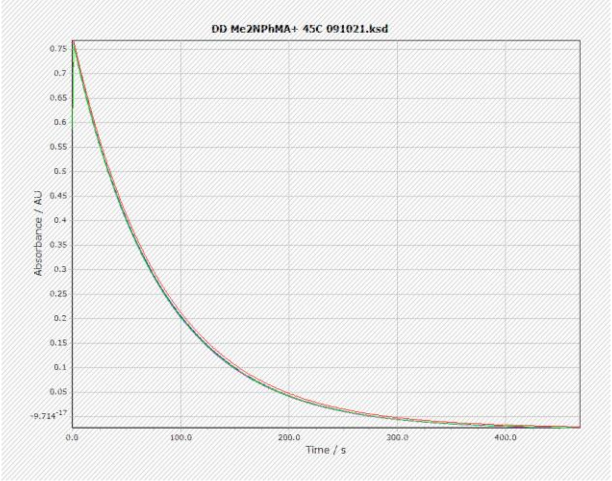

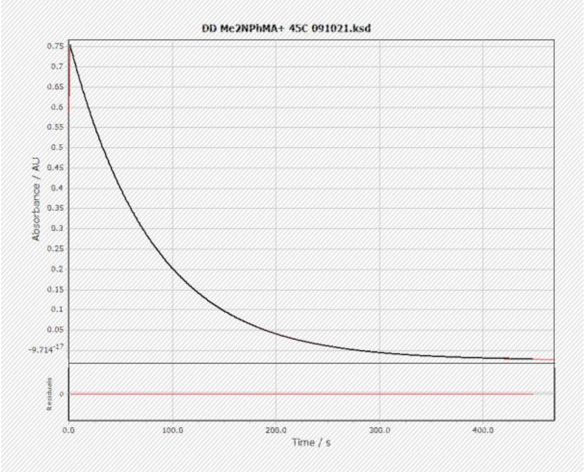

45°C D

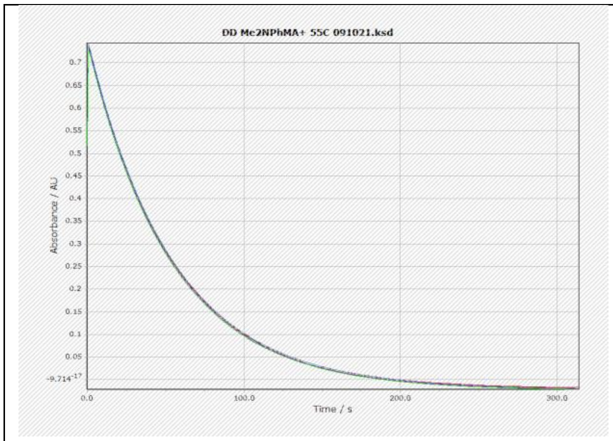

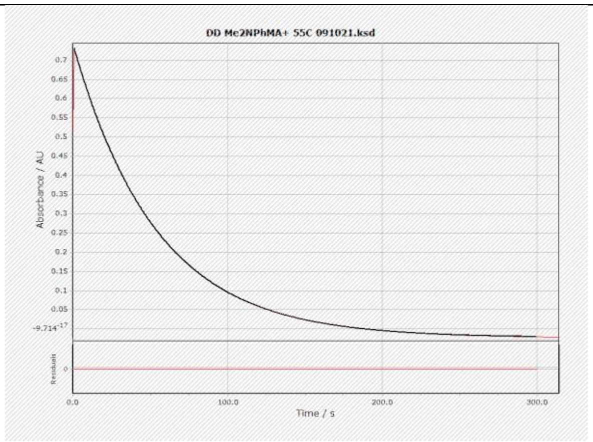

55℃ D

Day 2 data (September 16, 2021)

Temp (°C)	Pseudo-first-order rate constants k^{pfo} (s^{-1}) Trial H1	Trial H2	Trial H3	Trial H4	Trial H5	Trial H6	Average k_H^{pfo} (s^{-1})	Stdev	k_{2H} ($M^{-1}s^{-1}$)	Stdev [a]
55	0.07903	0.07991	0.07900	0.07912	0.07929	0.07942	0.07930	0.00034	13.21583	0.05685
45	0.05556	0.05552	0.05612	0.05596	0.05584	0.05560	0.05577	0.00024	9.29444	0.04075
35	0.03778	0.03798	0.03782	0.03822	0.03814	0.03819	0.03802	0.00019	6.33694	0.03184
25	0.02522	0.02518	0.02528	0.02532	0.02509	0.02496	0.02518	0.00013	4.19583	0.02205
15	0.01584	0.01584	0.01587	0.01585	0.01561	0.01577	0.01580	0.00010	2.63278	0.01625

Temp (°C)	Trial D1	Trial D2	Trial D3	Trial D4	Trial D5	Trial D6	Average k_D^{pfo} (s^{-1})	Stdev	k_{2D} ($M^{-1}s^{-1}$)	Stdev [a]
55	0.01853	0.01881	0.01879	0.01851	0.01878	0.01870	0.01869	0.00013	3.11444	0.02243
45	0.01224	0.01252	0.01252	0.01244	0.01255	0.01264	0.01249	0.00014	2.08083	0.02270
35	0.00793	0.00792	0.00796	0.00800	0.00796	0.00801	0.00796	0.00004	1.32722	0.00602
25	0.00489	0.00493	0.00493	0.00499	0.00489	0.00485	0.00491	0.00005	0.81889	0.00800
15	0.00280	0.00286	0.00284	0.00283	0.00283	0.00278	0.00282	0.00003	0.47056	0.00479

[a] = (Stdev(for k^{pfo})/k^{pfo})*k_{2H}

Six kinetic runs together with the averaged data	First-order kinetic fit of the averaged data as an example

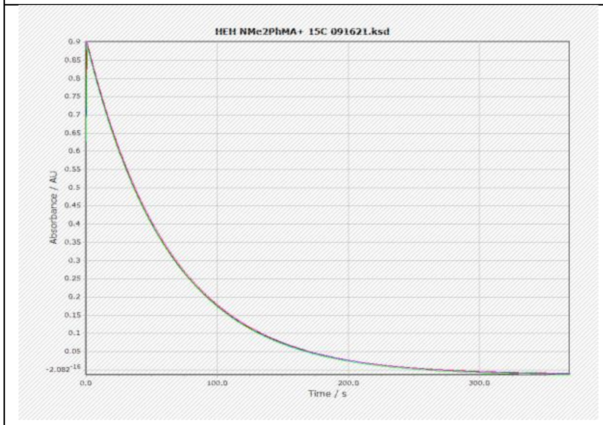

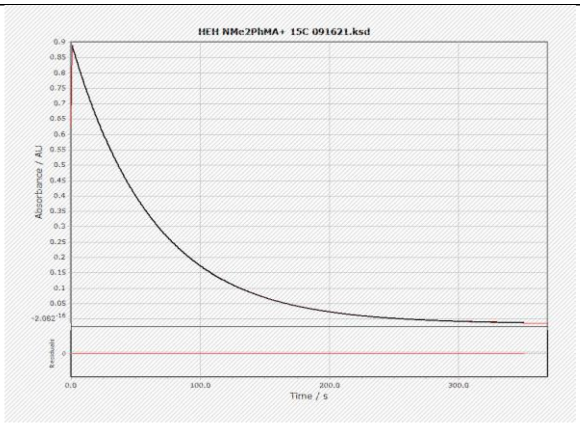

15℃ H

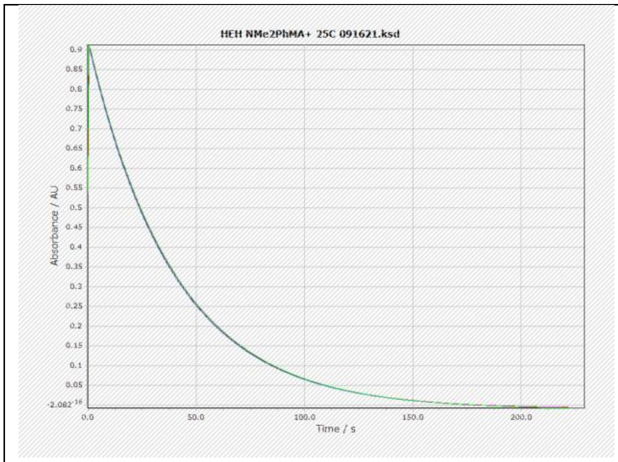
HEH NMe2PhMA+ 25C 091621.ksd
Absorbance / AU
Time / s

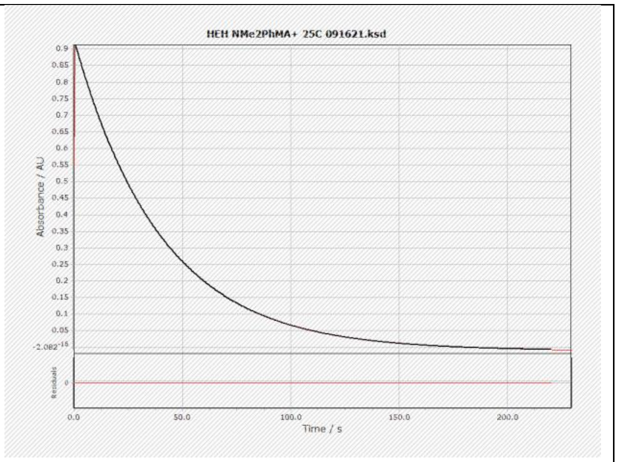
HEH NMe2PhMA+ 25C 091621.ksd
Absorbance / AU
Residuals
Time / s

25℃ H

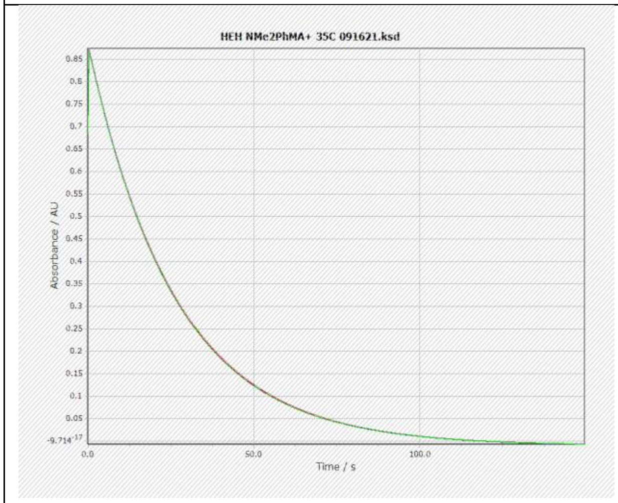
HEH NMe2PhMA+ 35C 091621.ksd
Absorbance / AU
Time / s

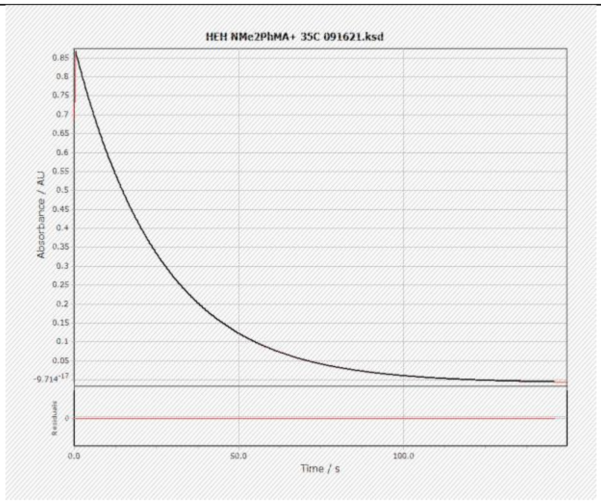
HEH NMe2PhMA+ 35C 091621.ksd
Absorbance / AU
Residuals
Time / s

35℃ H

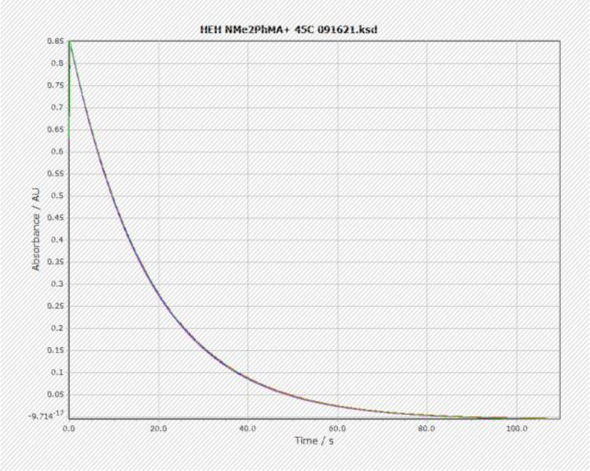
HEH NMe2PhMA+ 45C 091621.ksd
Absorbance / AU
Time / s

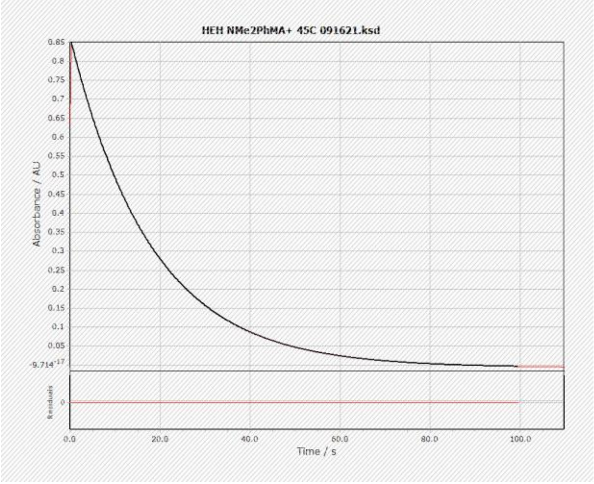
HEH NMe2PhMA+ 45C 091621.ksd
Absorbance / AU
Residuals
Time / s

45℃ H

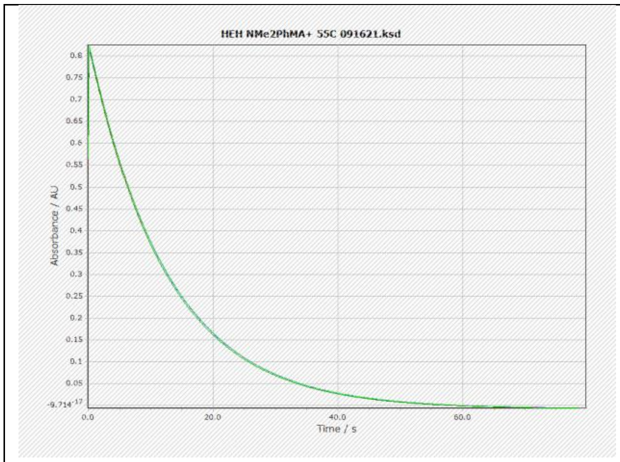
HEH NMe2PhMA+ 55C 091621.ksd
Absorbance / AU
Time / s

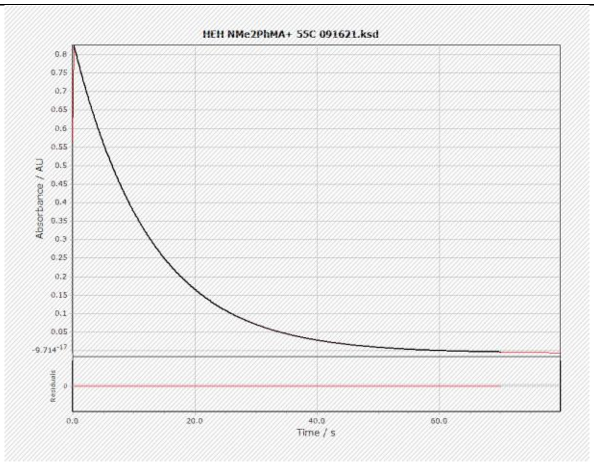
HEH NMe2PhMA+ 55C 091621.ksd
Absorbance / AU
Residuals
Time / s

55℃ H

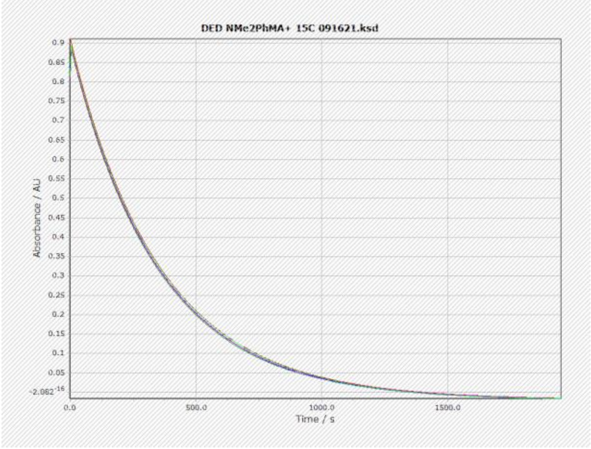
DED NMe2PhMA+ 15C 091621.ksd
Absorbance / AU
Time / s

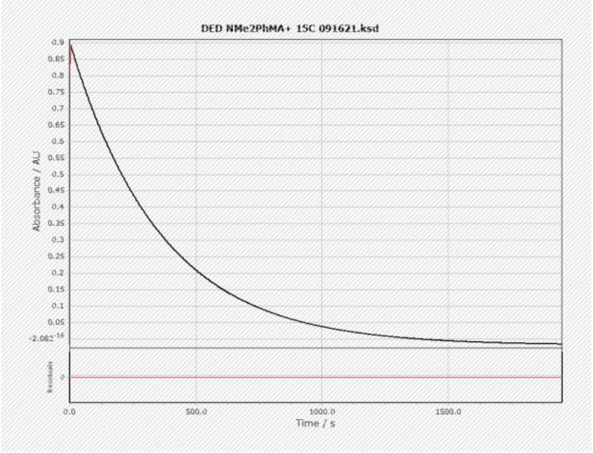
DED NMe2PhMA+ 15C 091621.ksd
Absorbance / AU
Residuals
Time / s

15℃ D

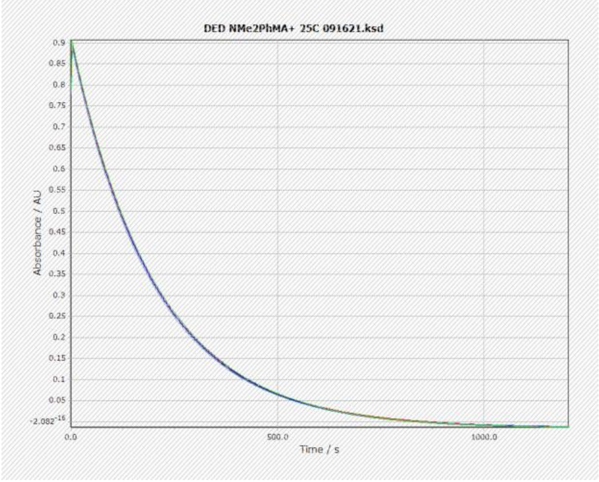
DED NMe2PhMA+ 25C 091621.ksd
Absorbance / AU
Time / s

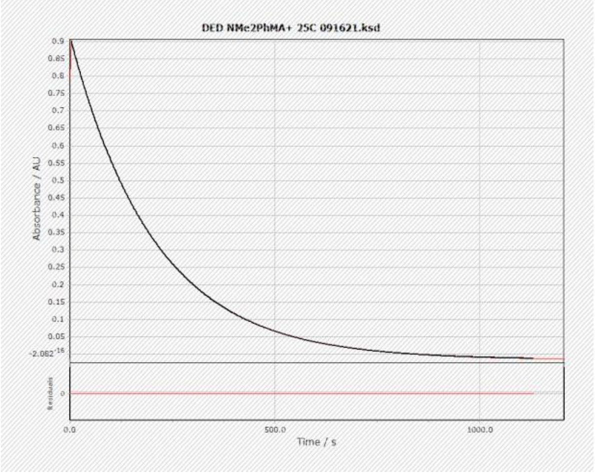
DED NMe2PhMA+ 25C 091621.ksd
Absorbance / AU
Residuals
Time / s

25℃ D

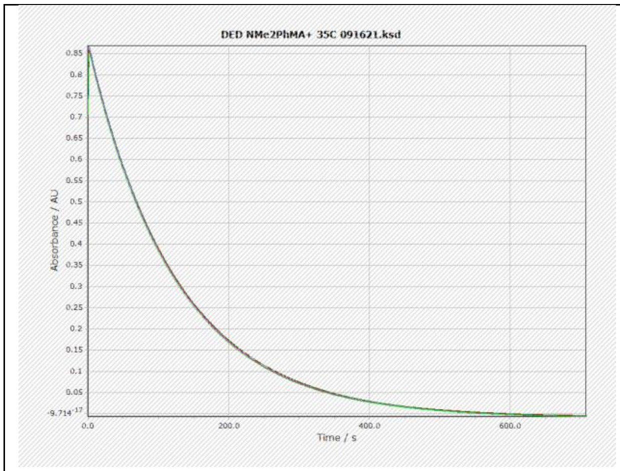
DED NMe2PhMA+ 35C 091621.ksd
Absorbance / AU
Time / s

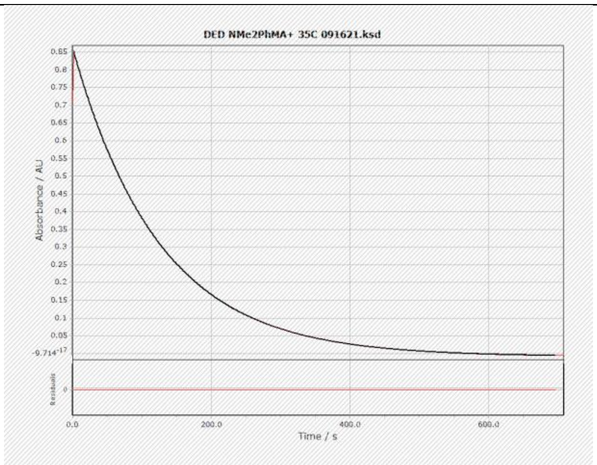
DED NMe2PhMA+ 35C 091621.ksd
Absorbance / AU
Residuals
Time / s

35℃ D

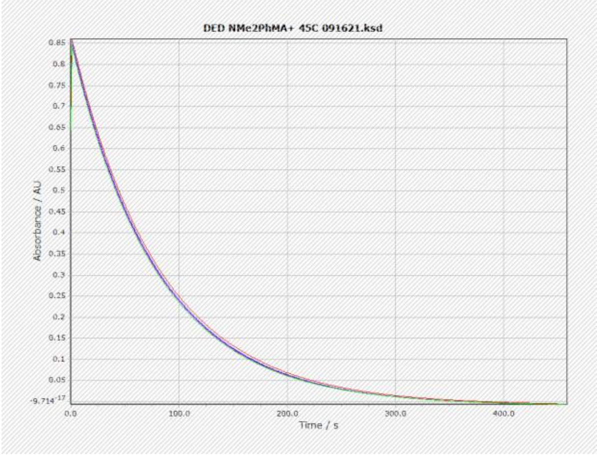
DED NMe2PhMA+ 45C 091621.ksd
Absorbance / AU
Time / s

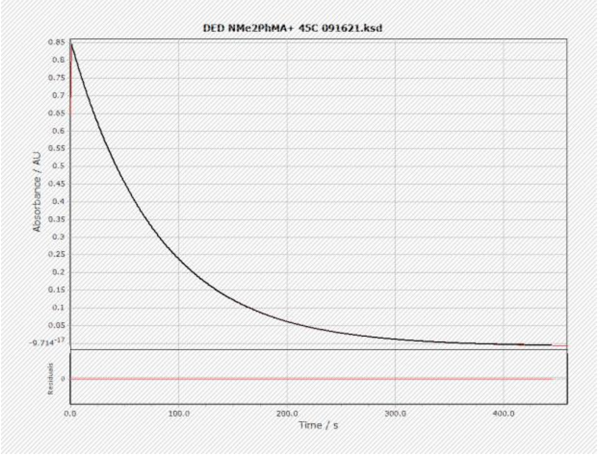
DED NMe2PhMA+ 45C 091621.ksd
Absorbance / AU
Residuals
Time / s

45℃ D

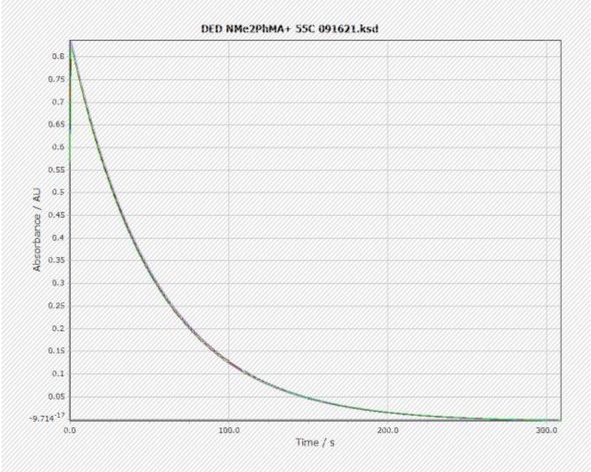
DED NMe2PhMA+ 55C 091621.ksd
Absorbance / AU
Time / s

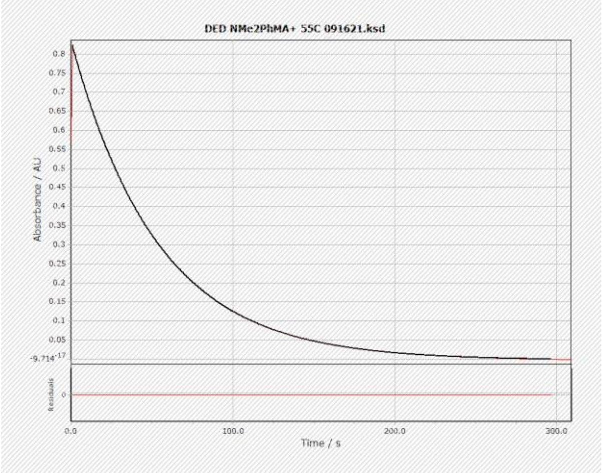
DED NMe2PhMA+ 55C 091621.ksd
Absorbance / AU
Residuals
Time / s

55℃ D

Primary kinetic data for the rate constants in Table S7

Day 1 data (June 15, 2021)

Temp (°C)	Pseudo-first-order rate constants k^{pfo} (s^{-1}) Trial H1	Trial H2	Trial H3	Trial H4	Trial H5	Trial H6	Average k_H^{pfo} (s^{-1})	Stdev	k_{2H} ($M^{-1}s^{-1}$)	Stdev [a]
55	0.1693	0.1718	0.1692	0.1730	0.1711	0.1698	0.1707	0.0015	28.4483	0.2539
45	0.1224	0.1208	0.1207	0.1198	0.1218	0.1212	0.1211	0.0009	20.1869	0.1537
35	0.0878	0.0882	0.0895	0.0894	0.0888	0.0906	0.0890	0.0010	14.8383	0.1728
25	0.0597	0.0606	0.0604	0.0609	0.0614	0.0611	0.0607	0.0006	10.1150	0.0999
15	0.0400	0.0399	0.0410	0.0410	0.0411	0.0404	0.0406	0.0005	6.7622	0.0882

Temp (°C)	Trial D1	Trial D2	Trial D3	Trial D4	Trial D5	Trial D6	Average k_D^{pfo} (s^{-1})	Stdev	k_{2D} ($M^{-1}s^{-1}$)	Stdev [a]
55	0.04035	0.04146	0.04129	0.04124	0.04147	0.04081	0.04110	0.00044	6.71415	0.07190
45	0.02806	0.02807	0.02822	0.02829	0.02812	0.02841	0.02820	0.00014	4.60560	0.02256
35	0.01870	0.01886	0.01874	0.01880	0.01895	0.01868	0.01879	0.00010	3.06904	0.01686
25	0.01191	0.01202	0.01198	0.01200	0.01211	0.01223	0.01204	0.00011	1.96698	0.01841
15	0.00735	0.00748	0.00747	0.00745	0.00746	0.00740	0.00744	0.00005	1.21449	0.00818

[a] = (Stdev(for k^{pfo})/k^{pfo})*k_{2H}

Six kinetic runs together with the averaged data

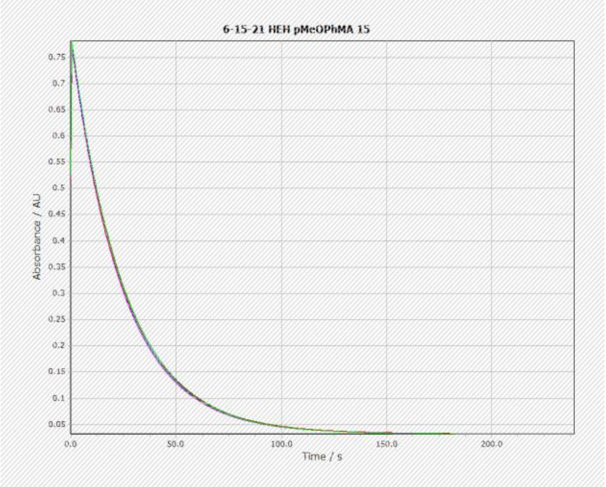

First-order kinetic fit of the averaged data as an example

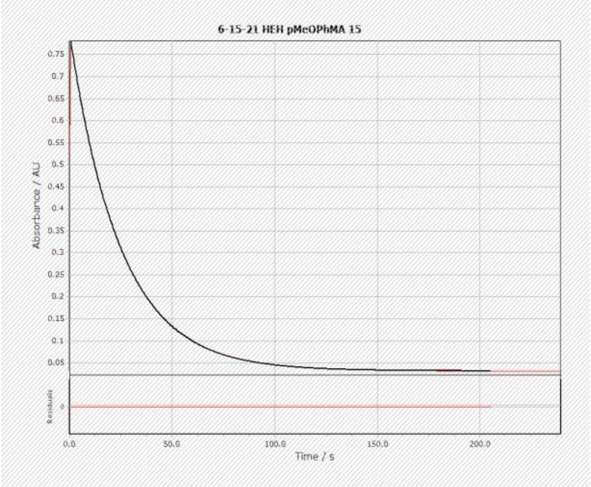

15℃ H

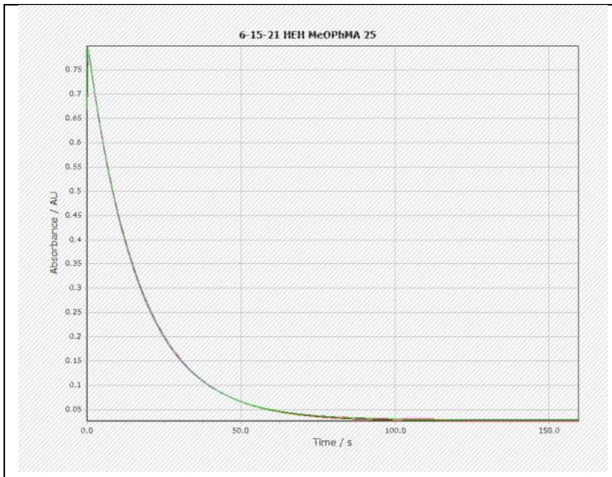
6-15-21 HEH MeOPhMA 25
Absorbance / AU
Time / s

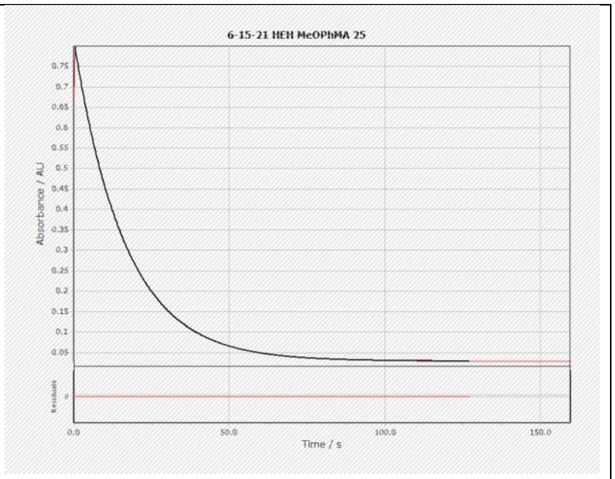
6-15-21 HEH MeOPhMA 25
Absorbance / AU
Residuals
Time / s

25℃ H

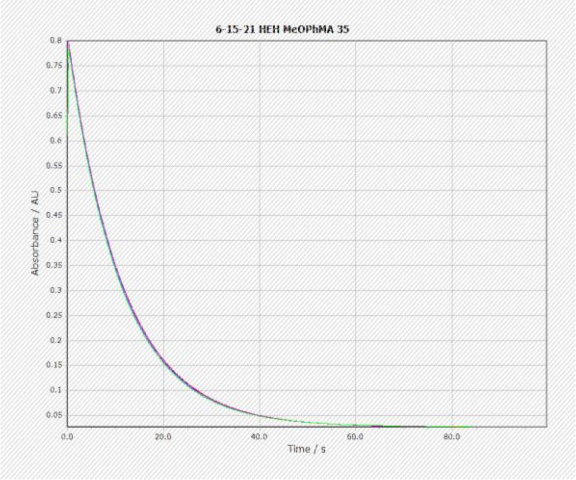
6-15-21 HEH MeOPhMA 35
Absorbance / AU
Time / s

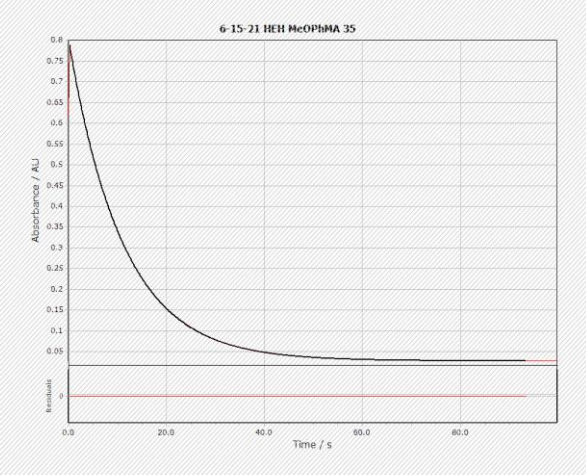
6-15-21 HEH MeOPhMA 35
Absorbance / AU
Residuals
Time / s

35℃ H

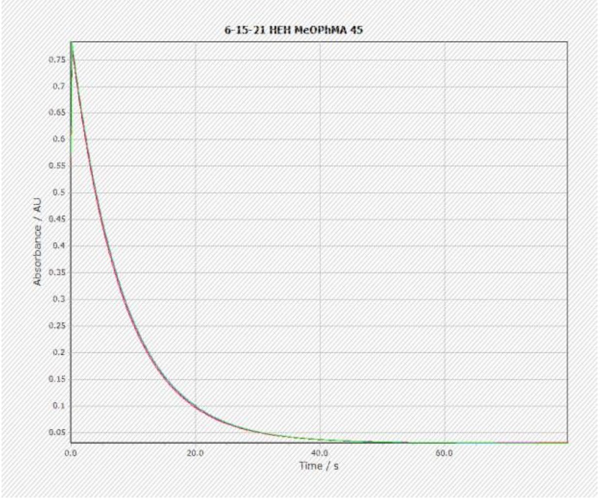
6-15-21 HEH MeOPhMA 45
Absorbance / AU
Time / s

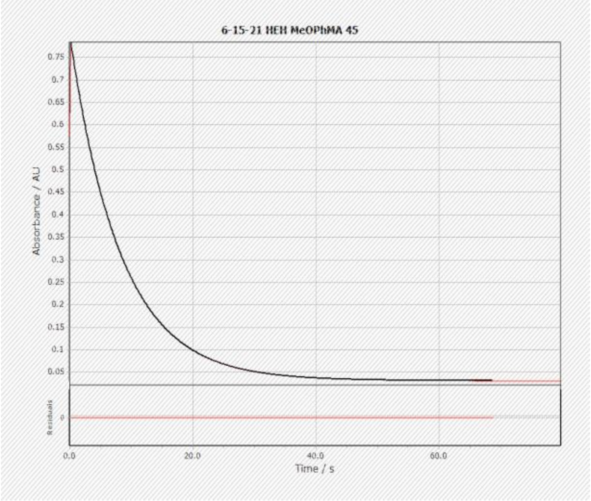
6-15-21 HEH MeOPhMA 45
Absorbance / AU
Residuals
Time / s

45℃ H

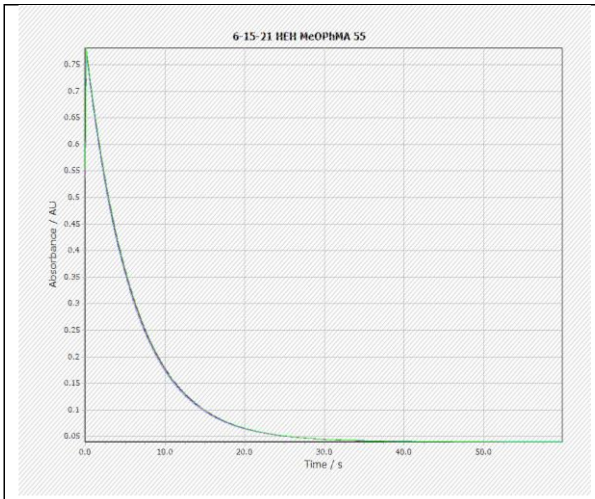
6-15-21 HEH MeOPhMA 55
Absorbance / AU
Time / s

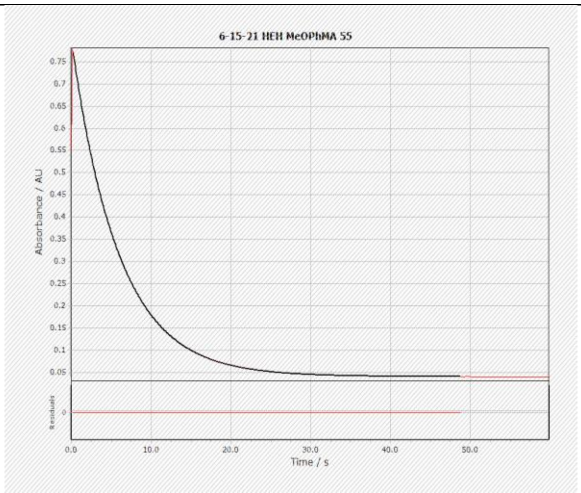
6-15-21 HEH MeOPhMA 55
Absorbance / AU
Residuals
Time / s

55℃ H

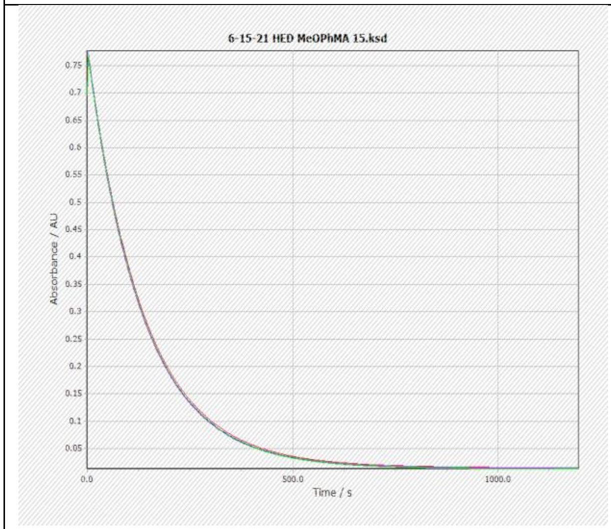
6-15-21 HED MeOPhMA 15.ksd
Absorbance / AU
Time / s

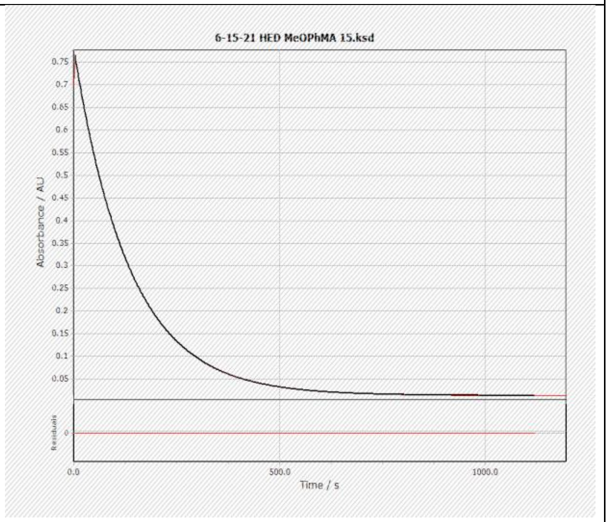
6-15-21 HED MeOPhMA 15.ksd
Absorbance / AU
Residuals
Time / s

15℃ D

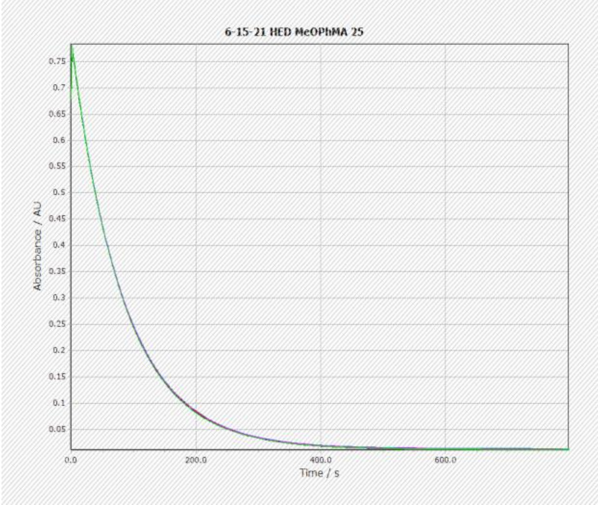
6-15-21 HED MeOPhMA 25
Absorbance / AU
Time / s

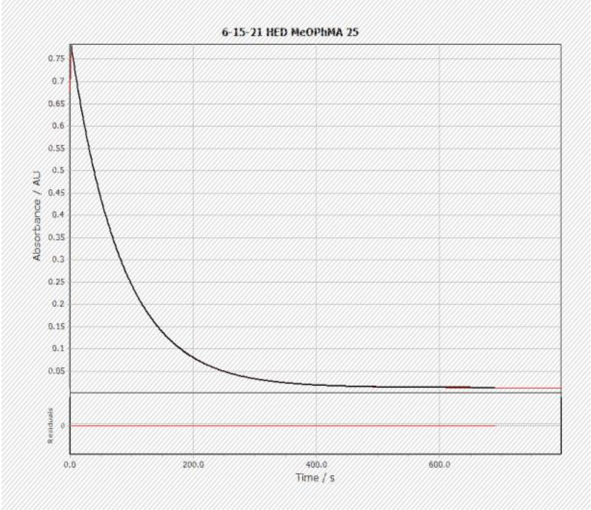
6-15-21 HED MeOPhMA 25
Absorbance / AU
Residuals
Time / s

25℃ D

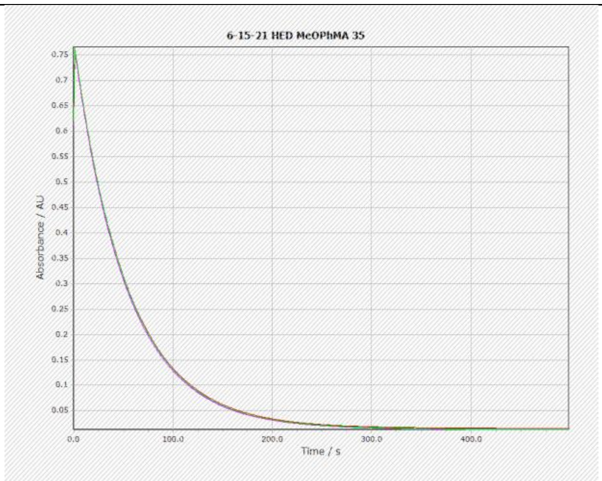
6-15-21 HED MeOPhMA 35
Absorbance / AU
Time / s
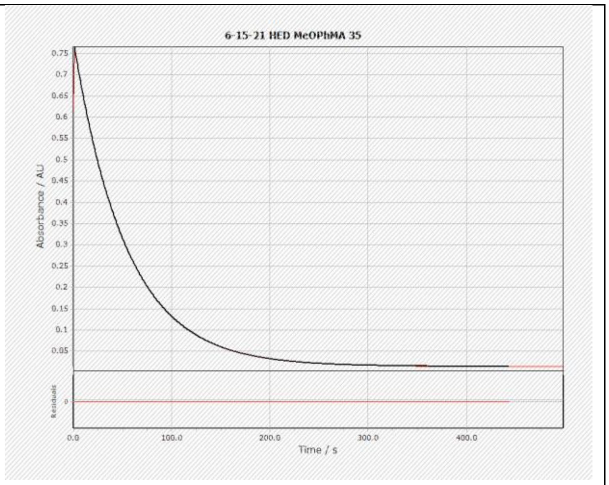
6-15-21 HED MeOPhMA 35
Absorbance / AU
Residuals
Time / s

35℃ D

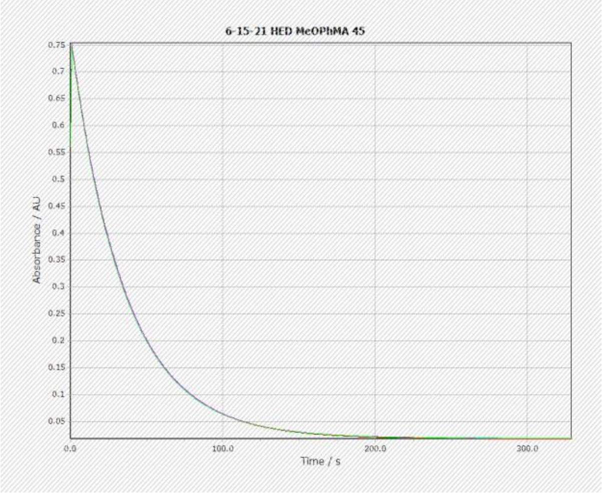
6-15-21 HED MeOPhMA 45
Absorbance / AU
Time / s
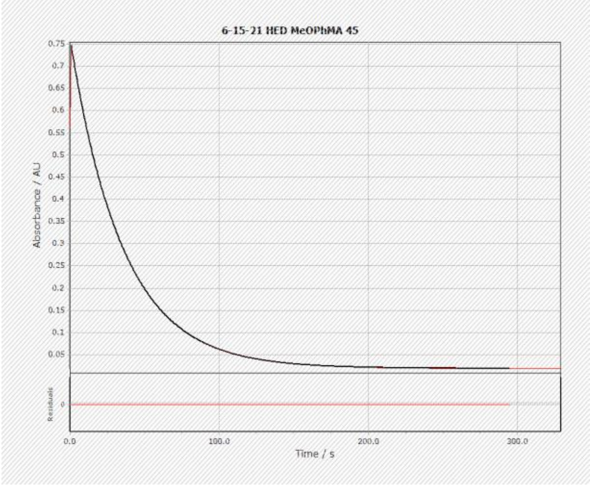
6-15-21 HED MeOPhMA 45
Absorbance / AU
Residuals
Time / s

45℃ D

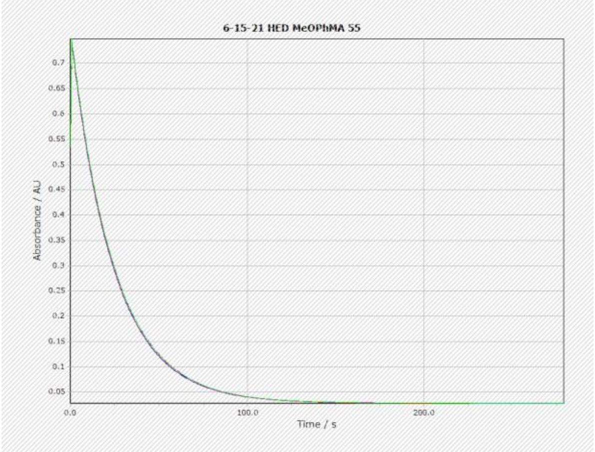
6-15-21 HED MeOPhMA 55
Absorbance / AU
Time / s
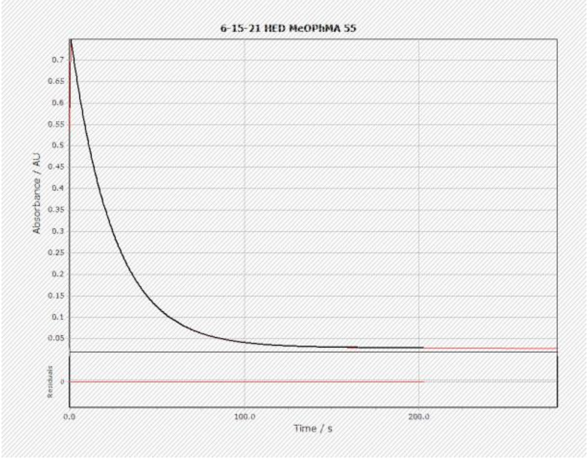
6-15-21 HED MeOPhMA 55
Absorbance / AU
Residuals
Time / s

55℃ D

Day 2 data (June 17, 2021)

Temp (°C)	Pseudo-first-order rate constants k^{pfo} (s^{-1}) Trial H1	Trial H2	Trial H3	Trial H4	Trial H5	Trial H6	Average k_H^{pfo} (s^{-1})	Stdev	k_{2H} ($M^{-1}s^{-1}$)	Stdev [a]
55	0.1702	0.1697	0.1686	0.1678	0.1714	0.1686	0.1694	0.0013	28.2281	0.2180
45	0.1219	0.1218	0.1217	0.1229	0.1223	0.1217	0.1220	0.0005	20.3381	0.0796
35	0.0868	0.0888	0.0884	0.0893	0.0887	0.0889	0.0885	0.0009	14.7453	0.1473
25	0.0584	0.0592	0.0596	0.0601	0.0610	0.0606	0.0598	0.0009	9.9658	0.1575
15	0.0401	0.0399	0.0405	0.0403	0.0402	0.0408	0.0403	0.0003	6.7150	0.0538
**Temp (°C)**	**Trial D1**	**Trial D2**	**Trial D3**	**Trial D4**	**Trial D5**	**Trial D6**	**Average k_D^{pfo} (s^{-1})**	**Stdev**	**k_{2D} ($M^{-1}s^{-1}$)**	**Stdev [a]**
55	0.04129	0.04142	0.04134	0.04135	0.04156	0.04137	0.04139	0.00009	6.85063	0.01558
45	0.02758	0.02791	0.02825	0.02854	0.02847	0.02770	0.02808	0.00040	4.64700	0.06686
35	0.01871	0.01883	0.01889	0.01882	0.01851	0.01886	0.01877	0.00014	3.10683	0.02338
25	0.01181	0.01183	0.01178	0.01190	0.01180	0.01194	0.01184	0.00006	1.96032	0.01040
15	0.00742	0.00763	0.00753	0.00755	0.00758	0.00742	0.00752	0.00009	1.24499	0.01418

[a] = (Stdev(for k^{pfo})/k^{pfo})*k_{2H}

Six kinetic runs together with the averaged data	First-order kinetic fit of the averaged data as an example
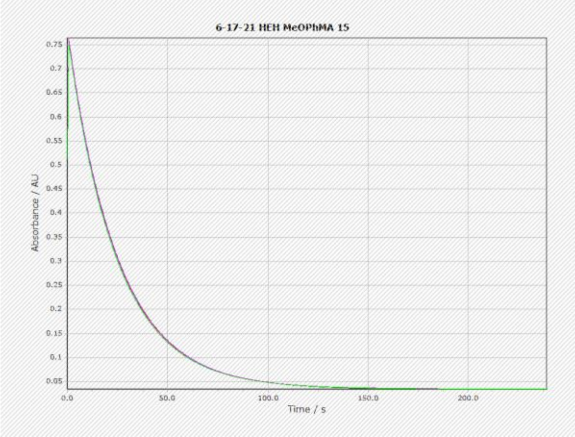	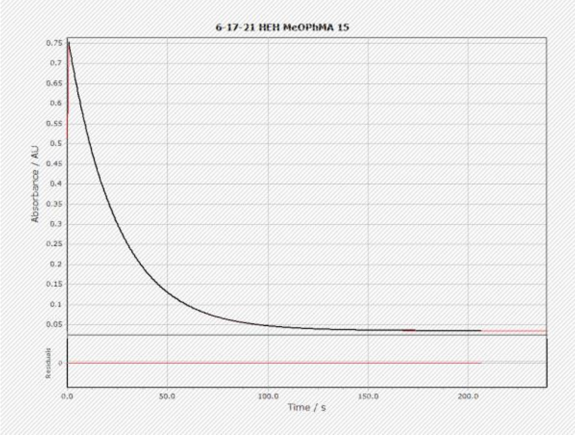

15 °C H

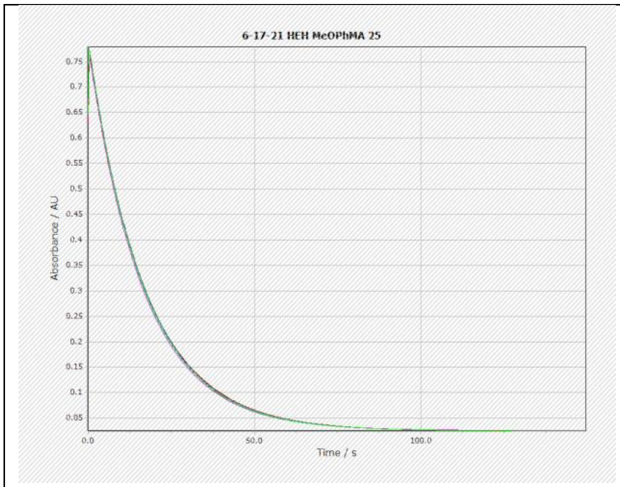

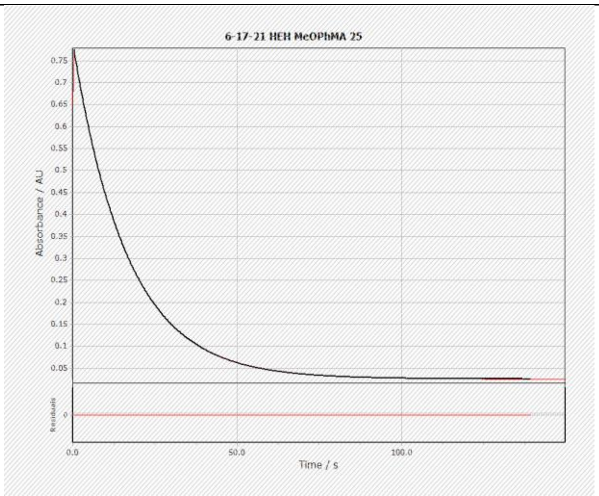

25°C H

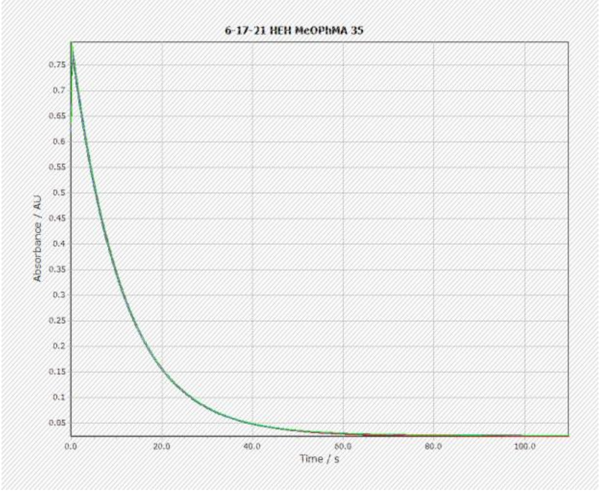

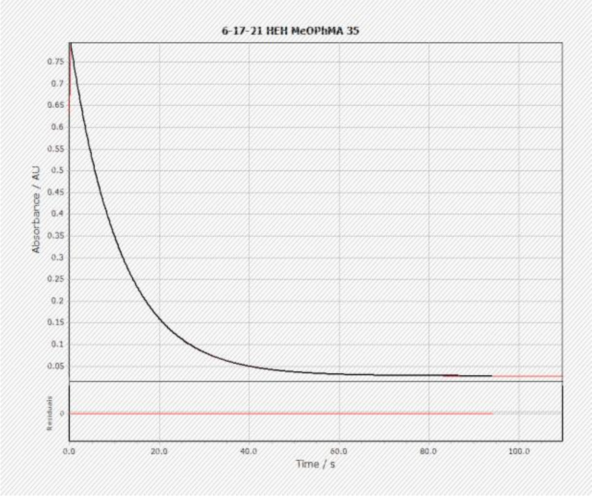

35°C H

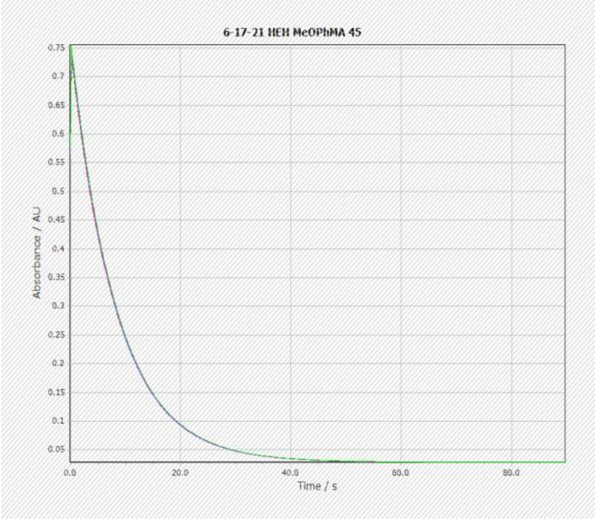

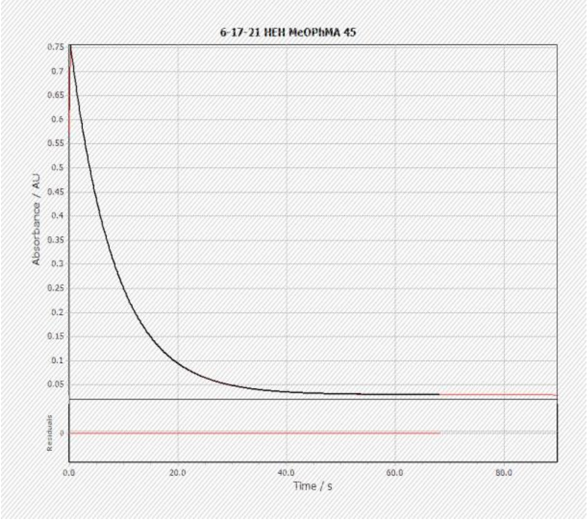

45°C H

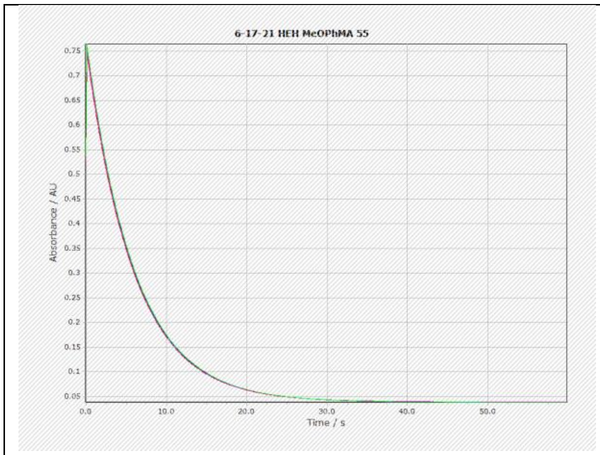
6-17-21 HEH MeOPhMA 55
Absorbance / AU
Time / s

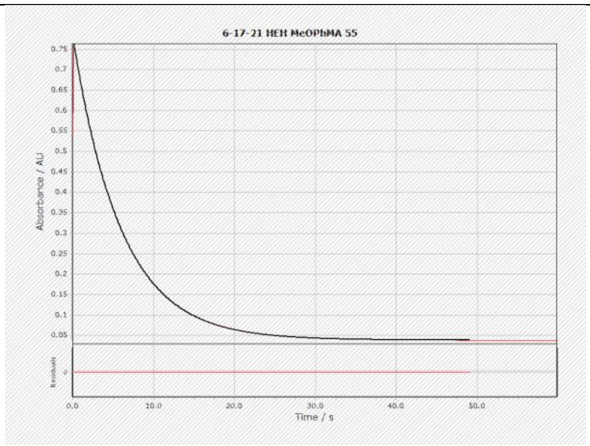
6-17-21 HEH MeOPhMA 55
Absorbance / AU
Residuals
Time / s

55℃ H

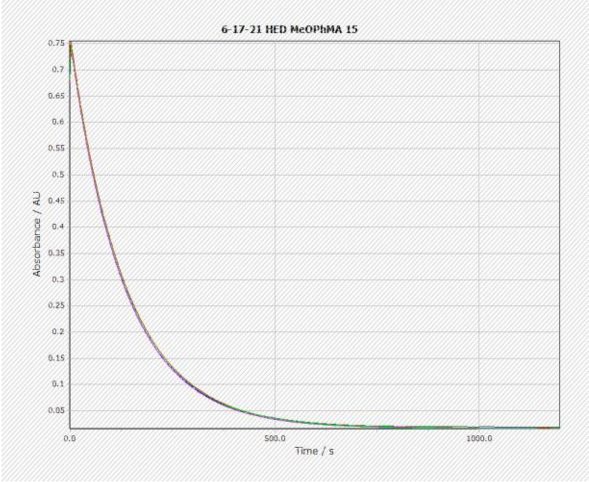
6-17-21 HED MeOPhMA 15
Absorbance / AU
Time / s

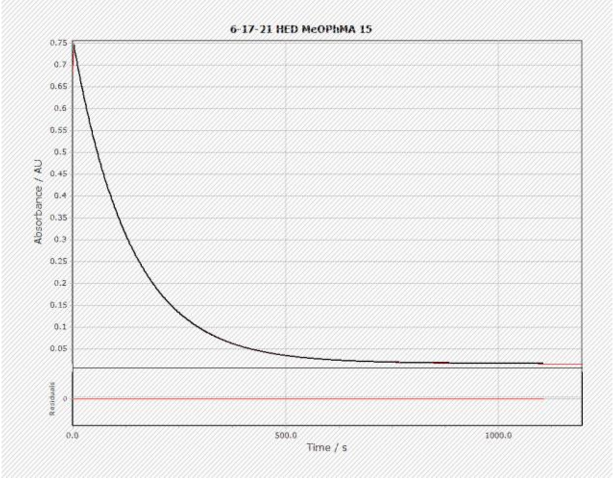
6-17-21 HED MeOPhMA 15
Absorbance / AU
Residuals
Time / s

15℃ D

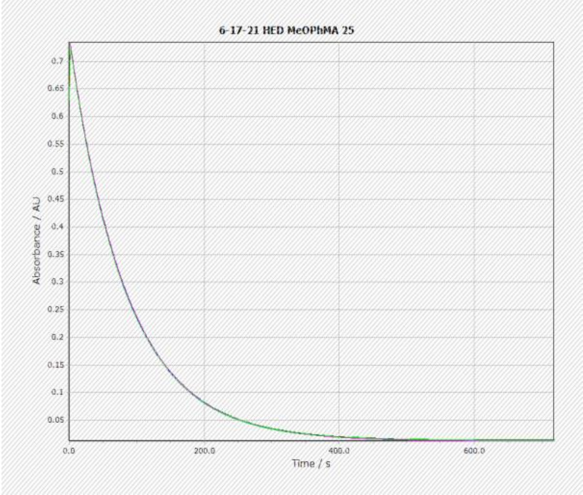
6-17-21 HED MeOPhMA 25
Absorbance / AU
Time / s

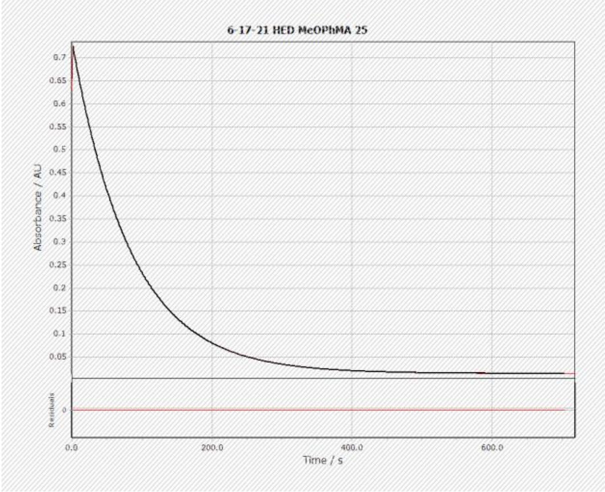
6-17-21 HED MeOPhMA 25
Absorbance / AU
Residuals
Time / s

25℃ D

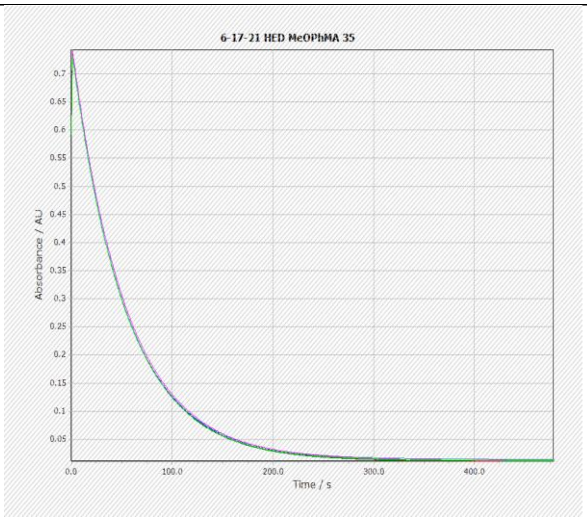
6-17-21 HED MeOPhMA 35
Absorbance / AU
Time / s

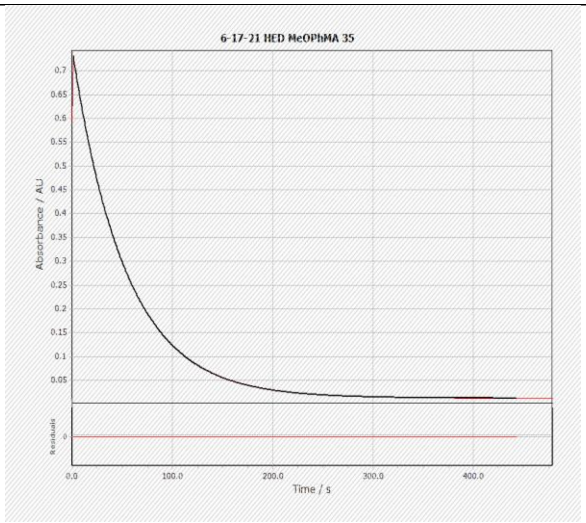
6-17-21 HED MeOPhMA 35
Absorbance / AU
Time / s

35℃ D

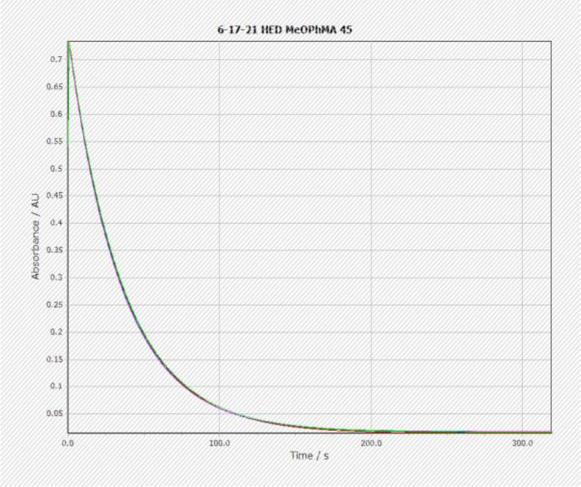
6-17-21 HED MeOPhMA 45
Absorbance / AU
Time / s

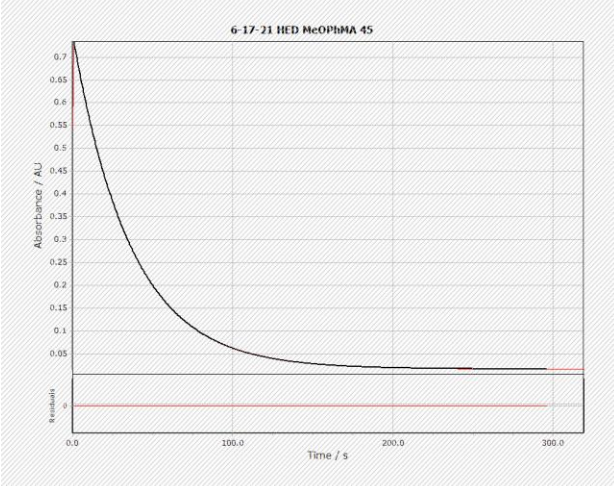
6-17-21 HED MeOPhMA 45
Absorbance / AU
Time / s

45℃ D

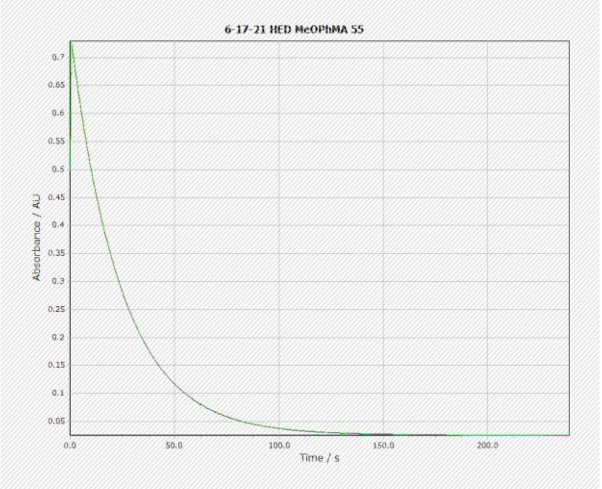
6-17-21 HED MeOPhMA 55
Absorbance / AU
Time / s

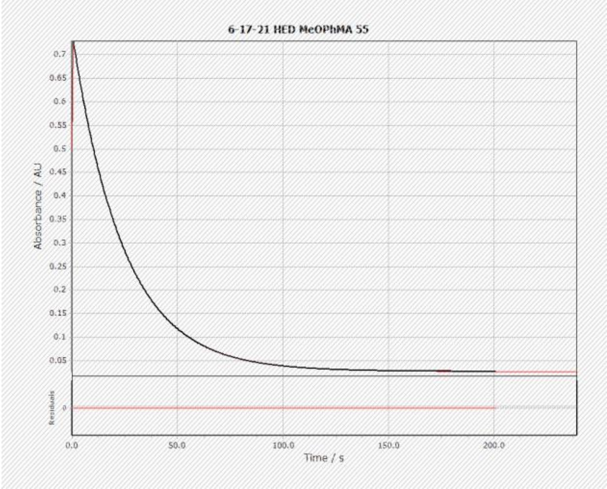
6-17-21 HED MeOPhMA 55
Absorbance / AU
Time / s

55℃ D

Primary kinetic data for the rate constants in Table S8

Day 1 data (June 25, 2021)

	Pseudo-first-order rate constants k^{pfo} (s^{-1})									
**Temp (°C)**	**Trial H1**	**Trial H2**	**Trial H3**	**Trial H4**	**Trial H5**	**Trial H6**	**Average k_H^{pfo} (s^{-1})**	**Stdev**	**k_{2H} (M^{-1}s^{-1})**	**Stdev [a]**
55	0.1875	0.1886	0.1886	0.1883	0.1914	0.19169	0.1893	0.001738	3.16E+01	0.28964
45	0.1378	0.1373	0.1371	0.1381	0.1378	0.1405	0.1381	0.001233	2.30E+01	0.20547
35	0.0984	0.0994	0.0973	0.09921	0.0991	0.09931	0.0988	0.000834	1.646E+01	0.13896
25	0.0674	0.0670	0.0681	0.0676	0.0683	0.0687	0.0678	0.000648	1.131E+01	0.10801
15	0.0451	0.0451	0.0449	0.0451	0.0452	0.0452	0.0451	0.000127	7.51E+00	0.02111

**Temp (°C)**	**Trial D1**	**Trial D2**	**Trial D3**	**Trial D4**	**Trial D5**	**Trial D6**	**Average k_D^{pfo} (s^{-1})**	**Stdev**	**k_{2D} (M^{-1}s^{-1})**	**Stdev [a]**
55	0.04584	0.04598	0.04563	0.04572	0.04600	0.04524	0.0457	0.000282	7.47E+00	0.04606
45	0.0310	0.0312	0.0313	0.0310	0.0313	0.0315	0.0312	0.000199	5.10E+00	0.03258
35	0.0205	0.0206	0.0208	0.0210	0.0204	0.0209	0.0207	0.000245	3.38E+00	0.04002
25	0.0126	0.0130	0.0130	0.0131	0.0132	0.0130	0.0130	0.000179	2.12E+00	0.02925
15	0.00810	0.00830	0.00840	0.00830	0.00820	0.00830	0.00830	0.000104	1.35E+00	0.01704

[a] = (Stdev(for k^{pfo})/k^{pfo})*k_{2H}

Six kinetic runs together with the averaged data

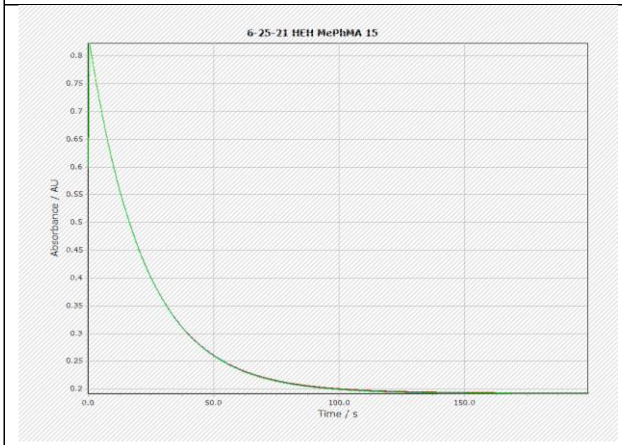

First-order kinetic fit of the averaged data as an example

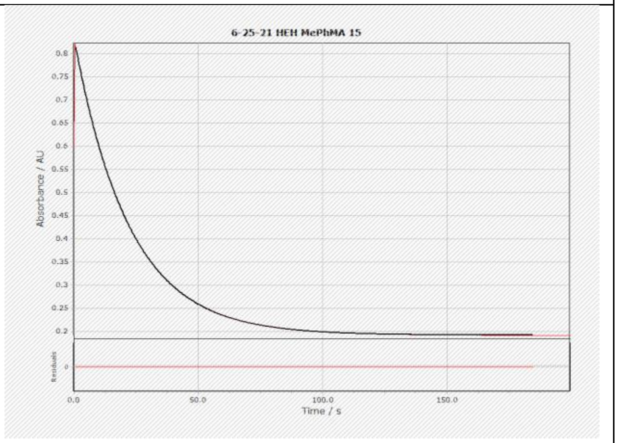

15℃ H

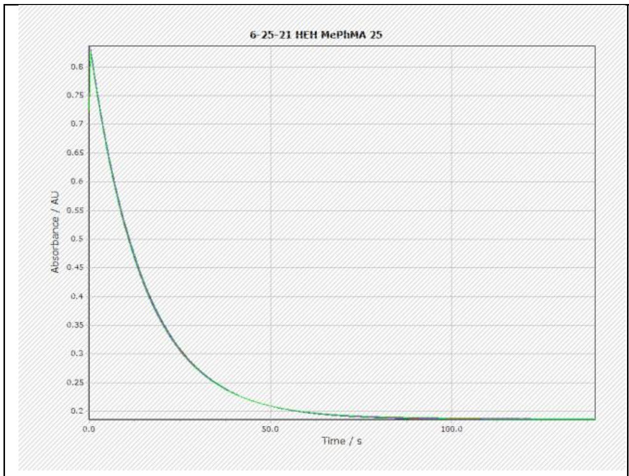

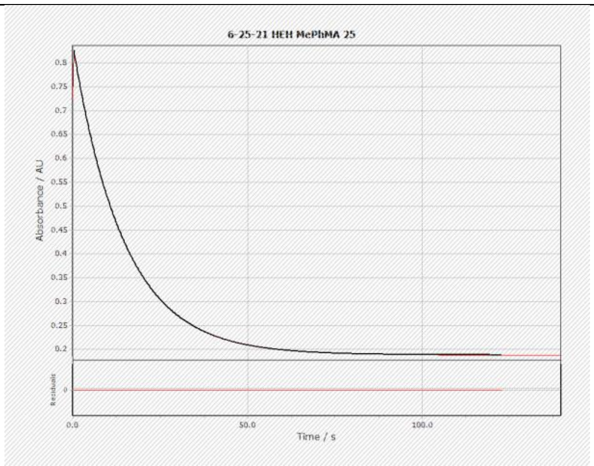

25°C H

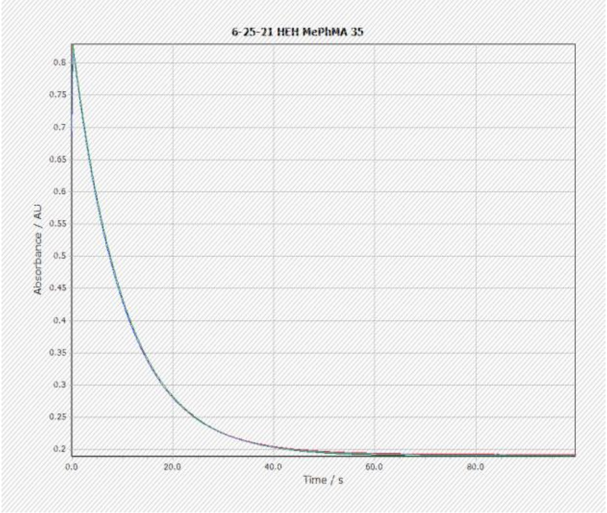

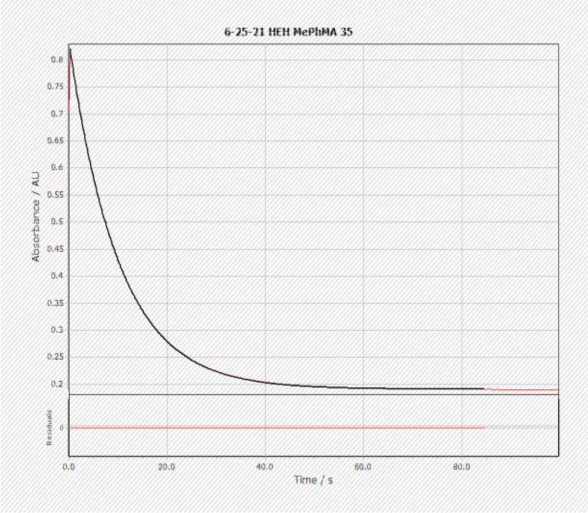

35°C H

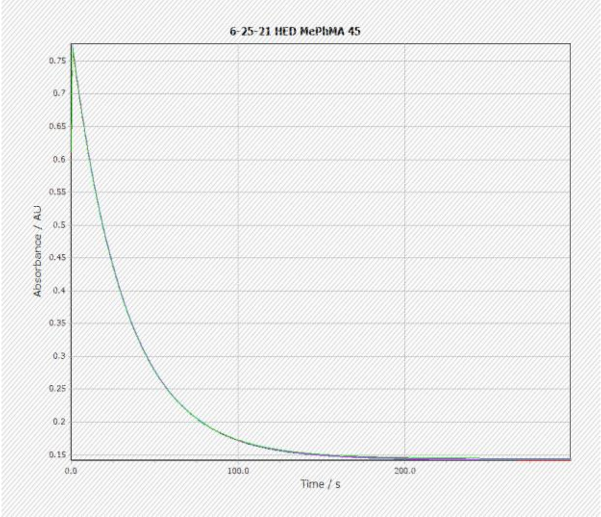

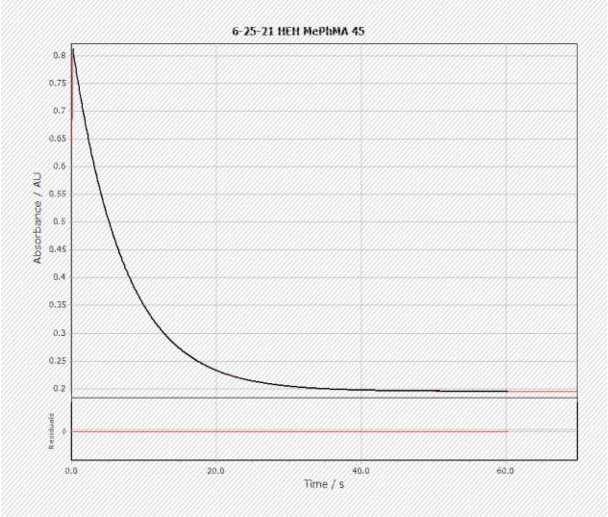

45°C H

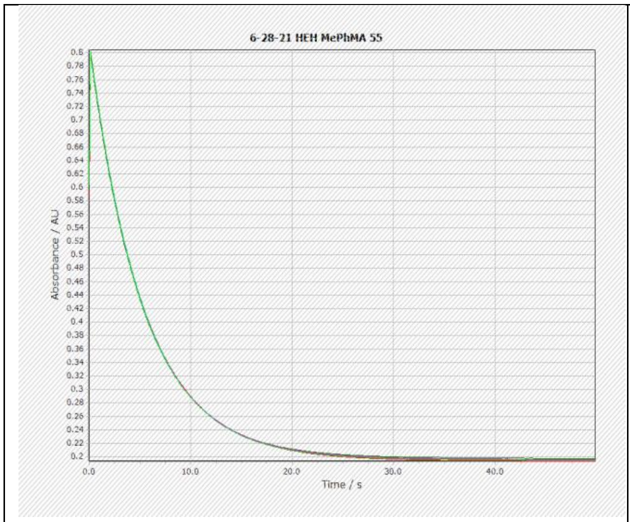
6-28-21 HEH MePhMA 55
Absorbance / AU
Time / s
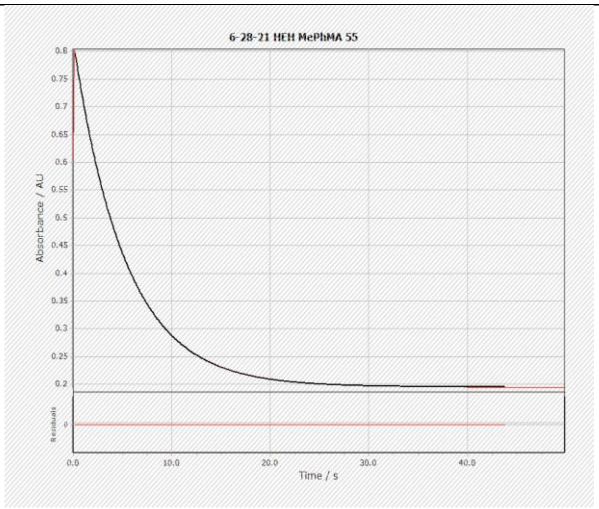
6-28-21 HEH MePhMA 55
Absorbance / AU
Residuals
Time / s

55℃ H

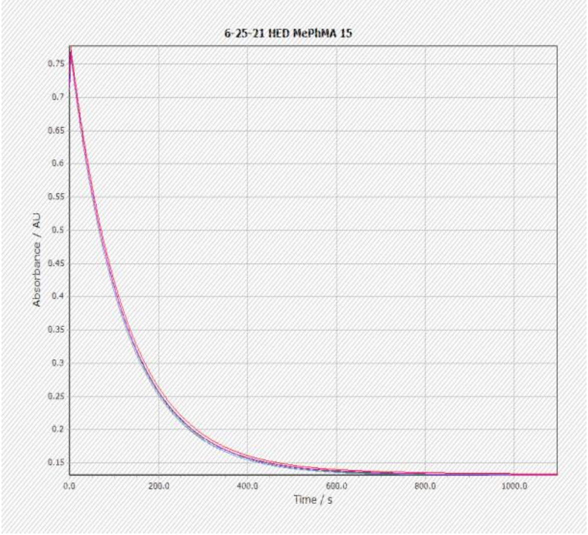
6-25-21 HED MePhMA 15
Absorbance / AU
Time / s
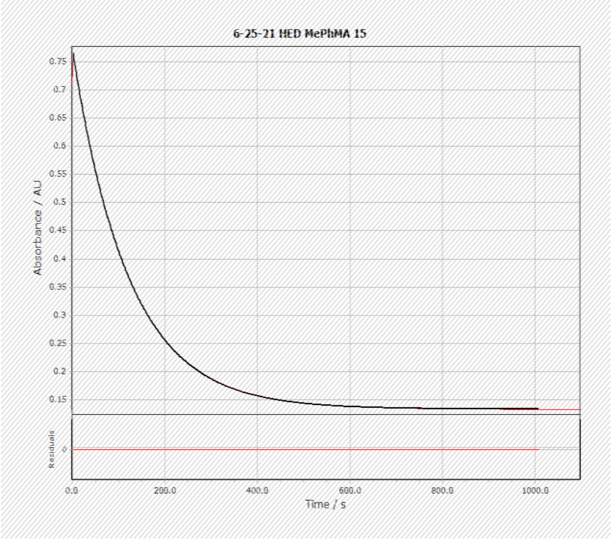
6-25-21 HED MePhMA 15
Absorbance / AU
Residuals
Time / s

15℃ D

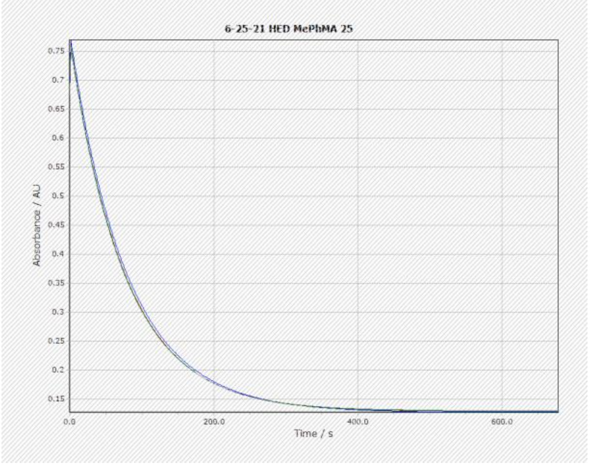
6-25-21 HED MePhMA 25
Absorbance / AU
Time / s
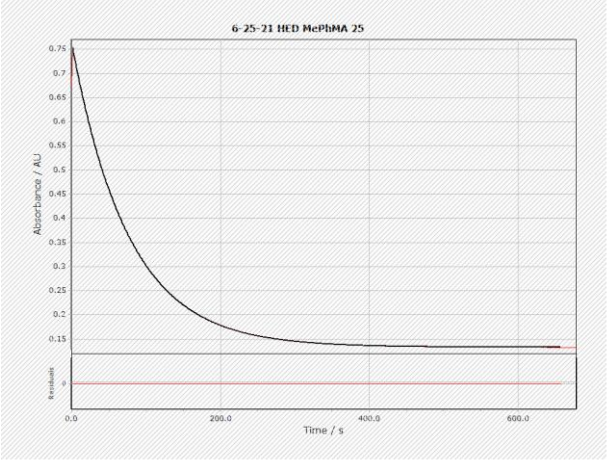
6-25-21 HED MePhMA 25
Absorbance / AU
Residuals
Time / s

25℃ D

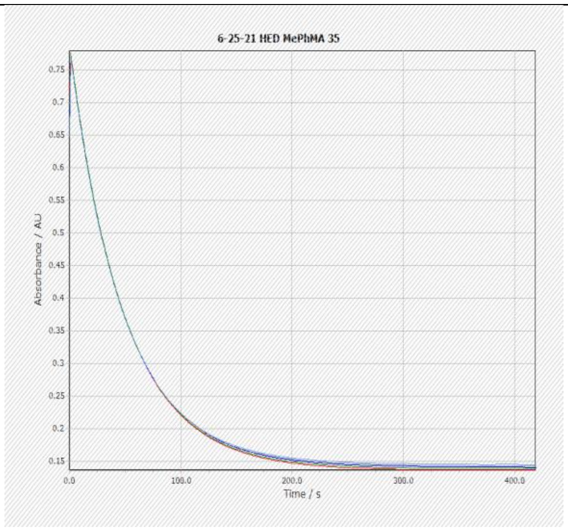
6-25-21 HED MePhMA 35
Absorbance / AU
Time / s

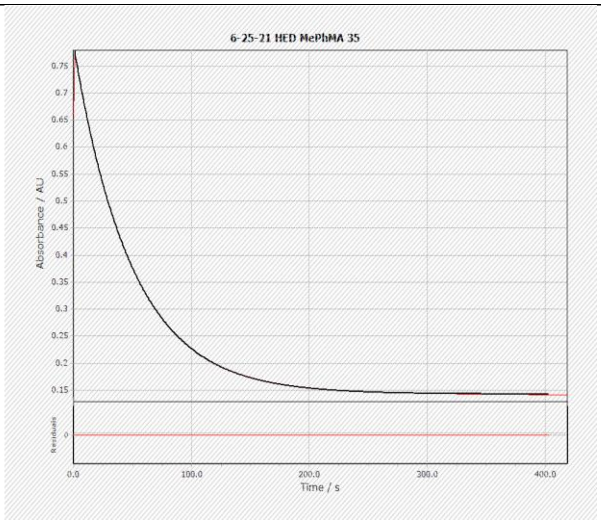
6-25-21 HED MePhMA 35
Absorbance / AU
Residuals
Time / s

35℃ D

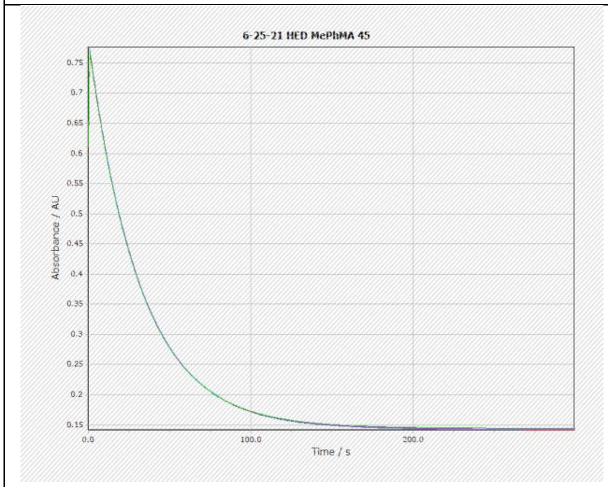
6-25-21 HED MePhMA 45
Absorbance / AU
Time / s

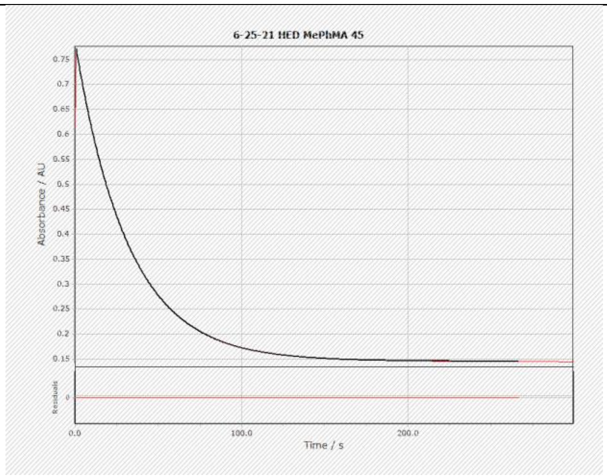
6-25-21 HED MePhMA 45
Absorbance / AU
Residuals
Time / s

45℃ D

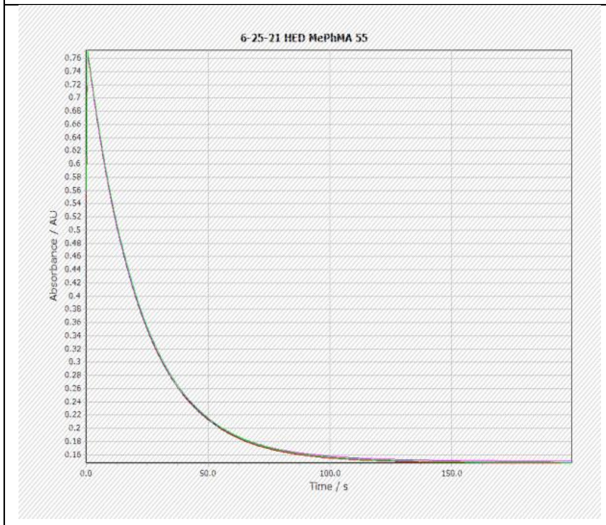
6-25-21 HED MePhMA 55
Absorbance / AU
Time / s

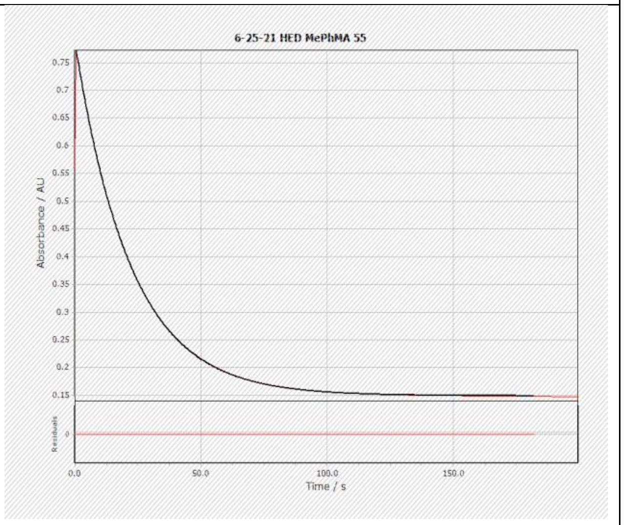
6-25-21 HED MePhMA 55
Absorbance / AU
Residuals
Time / s

55℃ D

Day 2 data (June 28, 2021)

**Temp (°C)**	Pseudo-first-order rate constants k^{pfo} (s^{-1}) **Trial H1**	**Trial H2**	**Trial H3**	**Trial H4**	**Trial H5**	**Trial H6**	**Average k_H^{pfo} (s^{-1})**	**Stdev**	**k_{2H} (M^{-1}s^{-1})**	**Stdev [a]**
55	0.1864	0.1919	0.1901	0.1892	0.1904	0.1903	0.1897	0.001836	3.16E+01	0.30606
45	0.1372	0.1371	0.1385	0.1389	0.1399	0.1374	0.1382	0.001105	2.30E+01	0.18415
35	0.0990	0.0991	0.1001	0.10037	0.10073	0.10214	0.1002	0.00116	1.67E+01	0.19339
25	0.0650	0.0671	0.0681	0.0688	0.0683	0.0687	0.0677	0.001446	1.13E+01	0.24098
15	0.0447	0.0444	0.0453	0.0451	0.0455	0.0451	0.0450	0.000399	7.50E+00	0.06656

**Temp (°C)**	**Trial D1**	**Trial D2**	**Trial D3**	**Trial D4**	**Trial D5**	**Trial D6**	**Average k_D^{pfo} (s^{-1})**	**Stdev**	**k_{2D} (M^{-1}s^{-1})**	**Stdev [a]**
55	0.04550	0.04514	0.04631	0.04549	0.04533	0.04510	0.0455	0.000441	7.53E+00	0.07298
45	0.0308	0.0308	0.0304	0.0305	0.0307	0.0312	0.0307	0.000283	5.09E+00	0.04685
35	0.0199	0.0205	0.0207	0.0206	0.0205	0.0208	0.0205	0.000316	3.39E+00	0.05237
25	0.0130	0.0130	0.0128	0.0130	0.0129	0.0130	0.0129	8.47E-05	2.14E+00	0.01402
15	0.00810	0.00800	0.00820	0.00840	0.00820	0.00830	0.00820	0.000109	1.36E+00	0.01808

[a] = (Stdev(for k^{pfo})/k^{pfo})*k_{2H}

Six kinetic runs together with the averaged data	First-order kinetic fit of the averaged data as an example
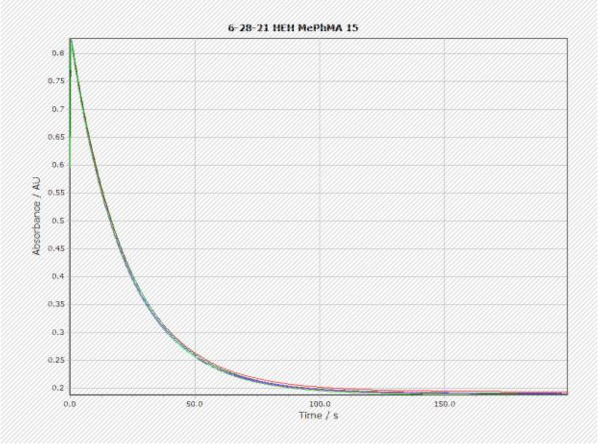	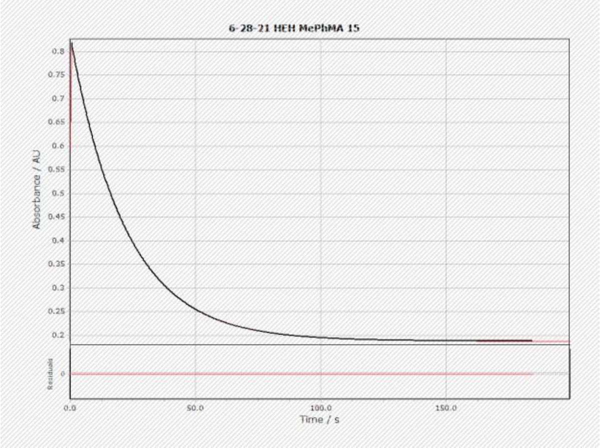
15°C H	

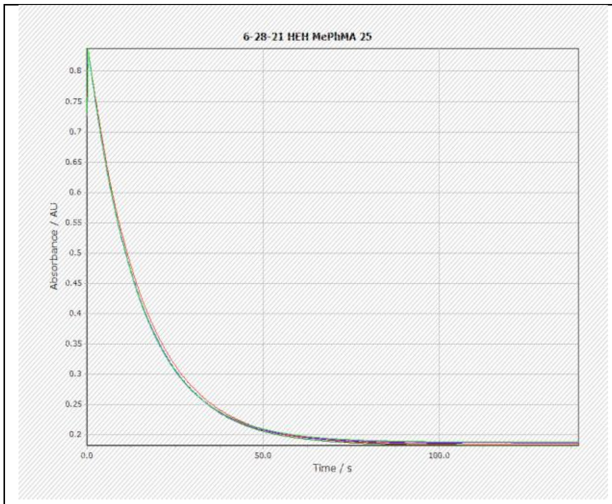

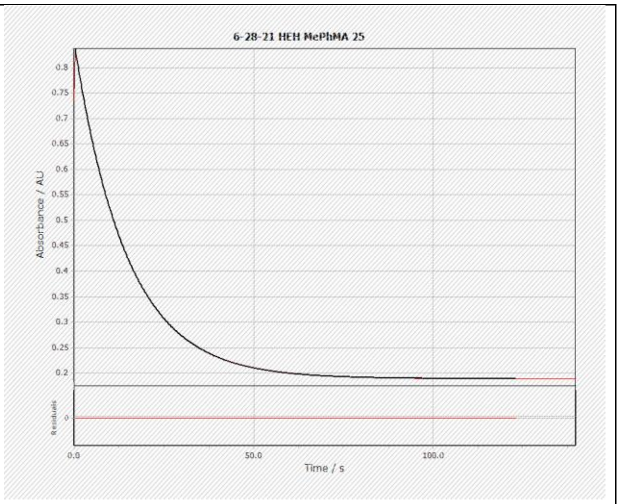

25°C H

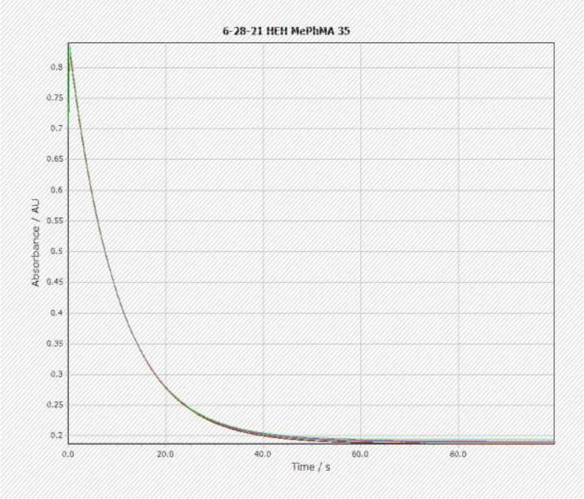

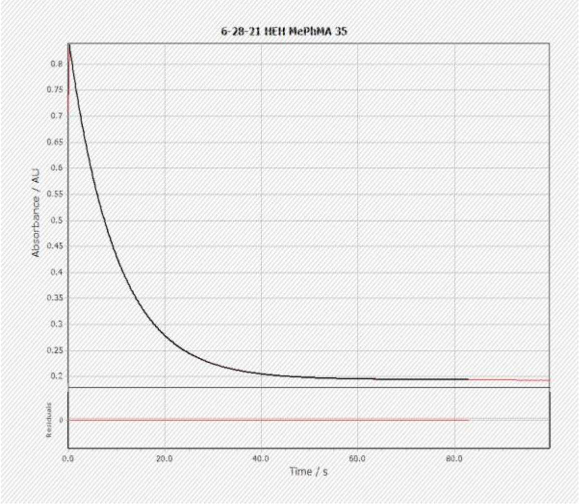

35°C H

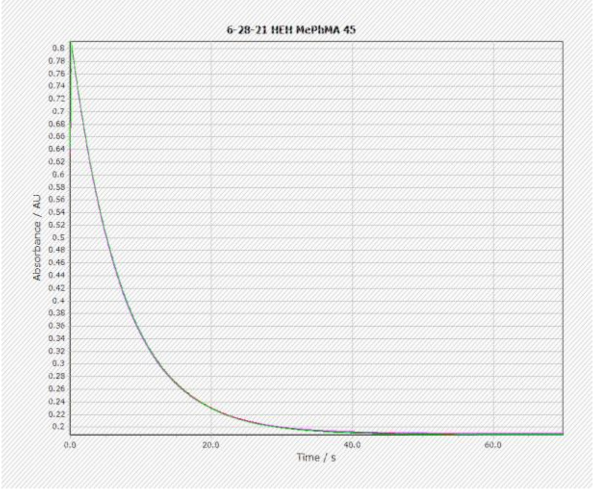

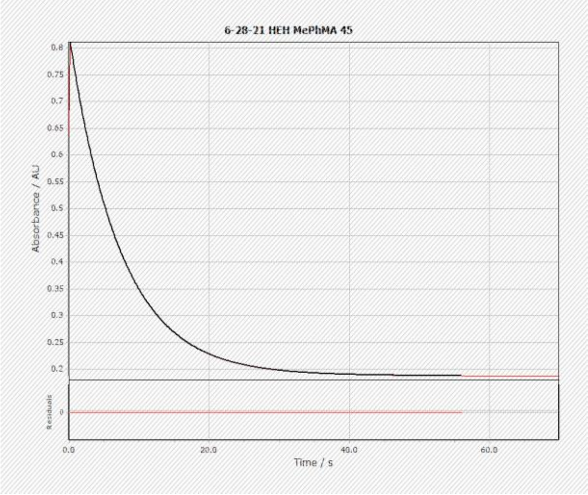

45°C H

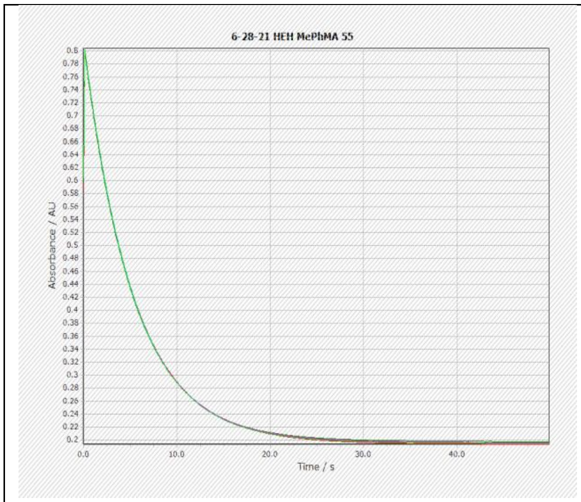

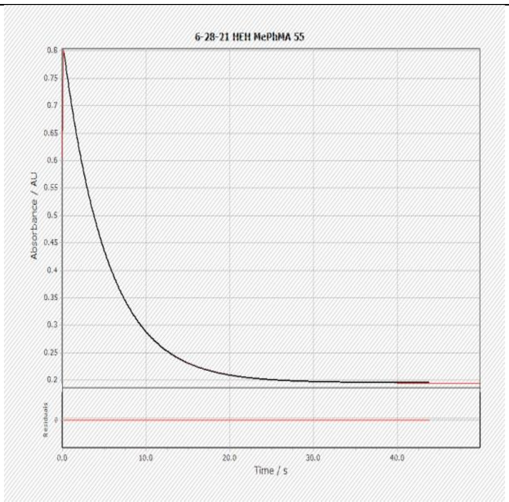

55°C H

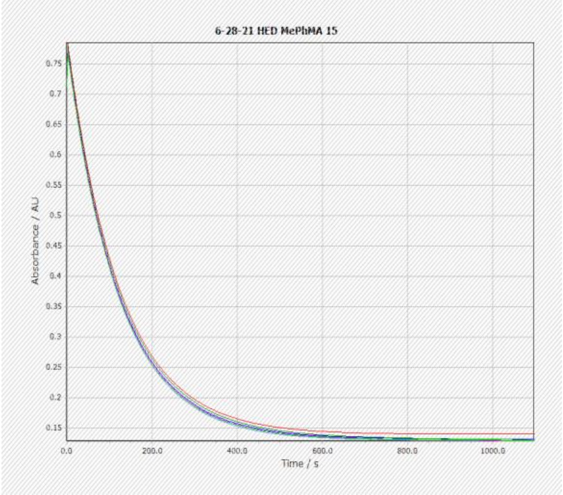

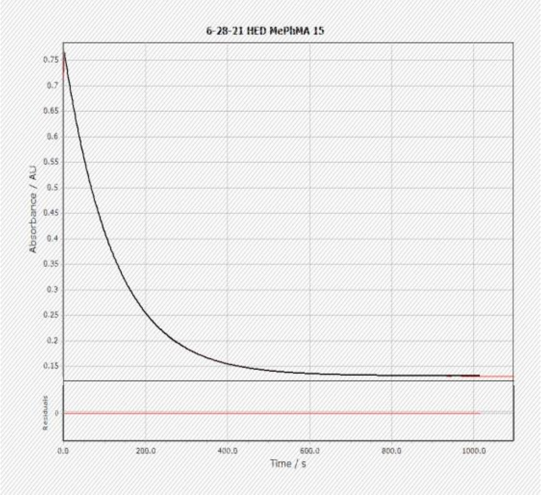

15°C D

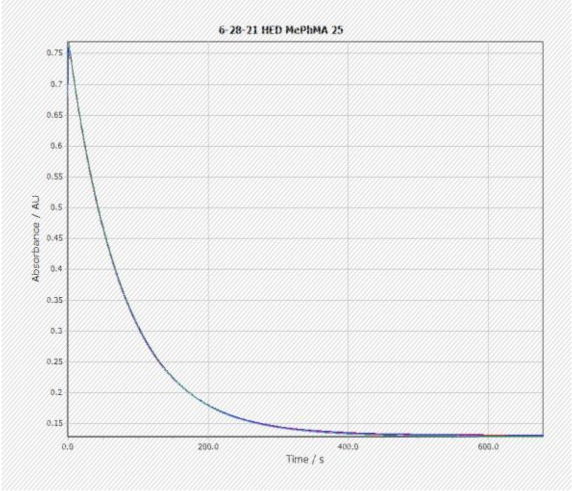

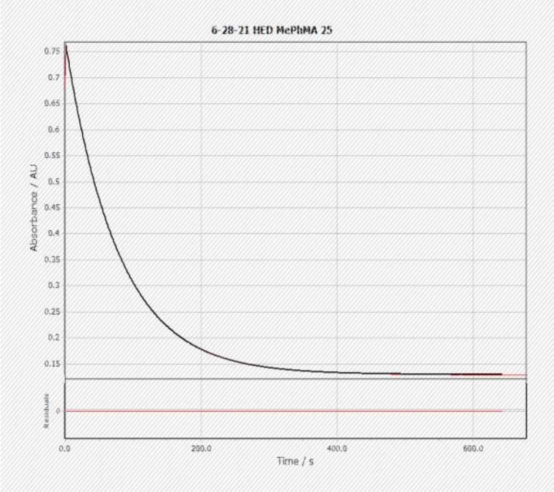

25°C D

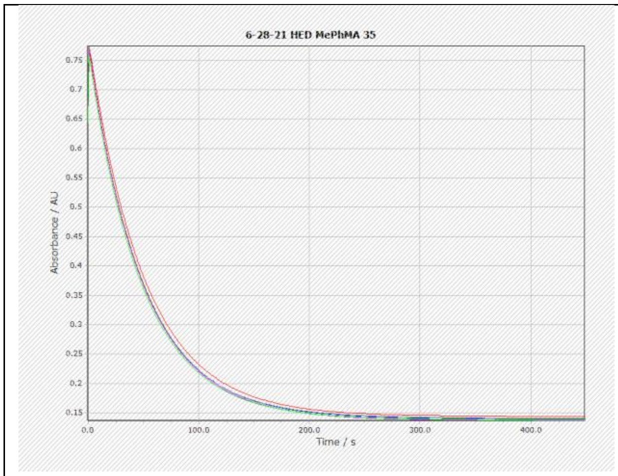
6-28-21 HED MePhMA 35
Absorbance / AU
Time / s

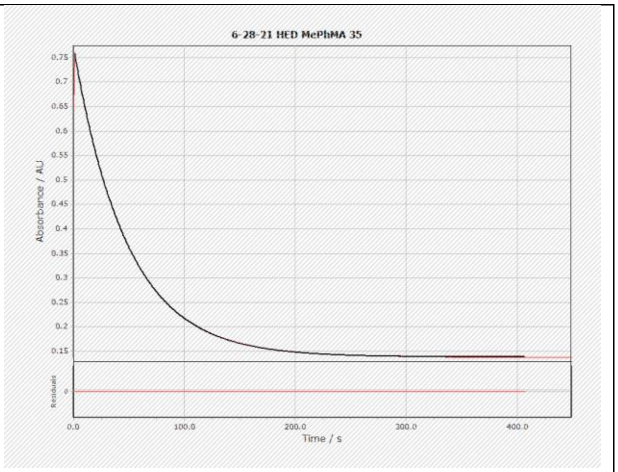
6-28-21 HED MePhMA 35
Absorbance / AU
Residuals
Time / s

35℃ D

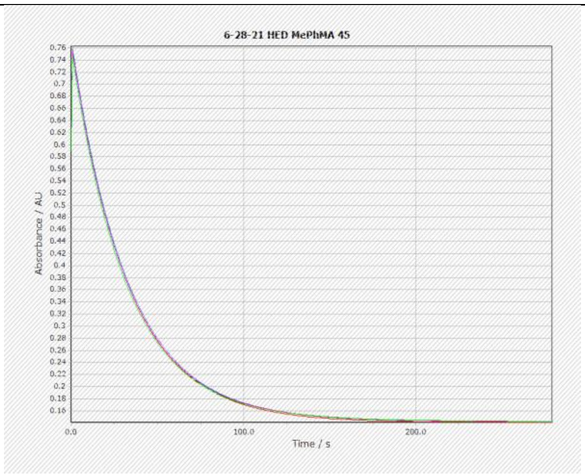
6-28-21 HED MePhMA 45
Absorbance / AU
Time / s

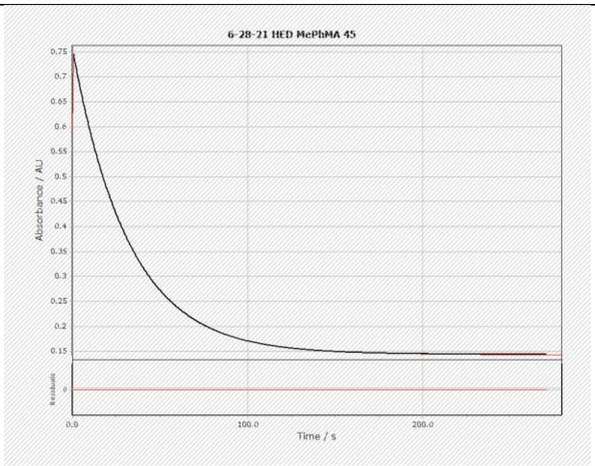
6-28-21 HED MePhMA 45
Absorbance / AU
Residuals
Time / s

45℃ D

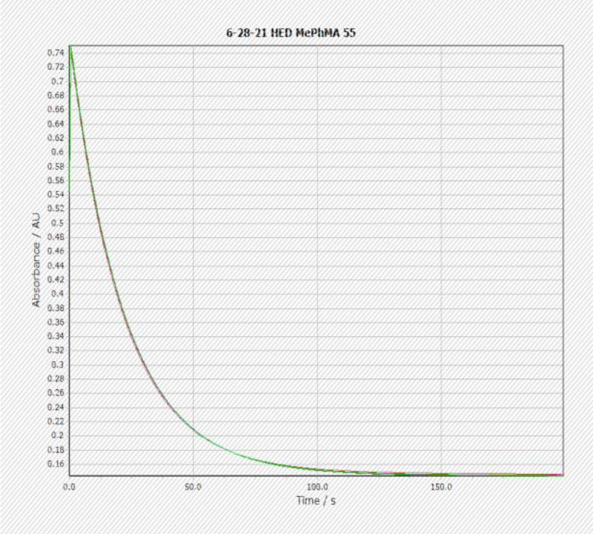
6-28-21 HED MePhMA 55
Absorbance / AU
Time / s

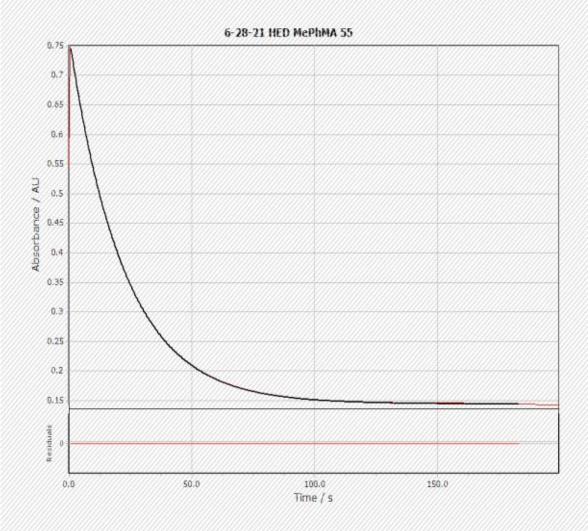
6-28-21 HED MePhMA 55
Absorbance / AU
Residuals
Time / s

55℃ D

Primary kinetic data for the rate constants in Table S9

Day 1 data (October 7, 2019)

	Pseudo-first-order rate constants k^{pfo} (s^{-1})									
**Temp (°C)**	**Trial H1**	**Trial H2**	**Trial H3**	**Trial H4**	**Trial H5**	**Trial H6**	**Average k_H^{pfo} (s^{-1})**	**Stdev**	**k_{2H} ($M^{-1}s^{-1}$)**	**Stdev [a]**
55	0.2328	0.2344	0.2335	0.2347	0.2376	0.2350	0.2347	0.00166	39.10942	0.27730
45	0.1714	0.1700	0.1698	0.1707	0.1682	0.1692	0.1699	0.001118	2.83E+01	0.18641
35	0.1199	0.1205	0.1190	0.1199	0.1195	0.1203	0.1198	0.00053	2.00E+01	0.08831
25	0.0821	0.0828	0.0821	0.0826	0.0829	0.0826	0.0825	0.000346	1.38E+01	0.05772
15	0.0539	0.0547	0.0545	0.0543	0.0548	0.0545	0.0544	0.000301	9.07E+00	0.05016
**Temp (°C)**	**Trial D1**	**Trial D2**	**Trial D3**	**Trial D4**	**Trial D5**	**Trial D6**	**Average k_D^{pfo} (s^{-1})**	**Stdev**	**k_{2D} ($M^{-1}s^{-1}$)**	**Stdev [a]**
55	0.05346	0.05277	0.05281	0.05284	0.05298	0.05292	0.0530	0.000254	8.65E+00	0.04144
45	0.0363	0.0352	0.0364	0.0361	0.0353	0.0365	0.0360	0.000588	5.88E+00	0.09602
35	0.0244	0.0241	0.0240	0.0240	0.0239	0.0237	0.0240	0.000198	3.92E+00	0.03240
25	0.0157	0.0156	0.0154	0.0155	0.0155	0.0155	0.0155	1E-04	2.54E+00	0.01633
15	0.00940	0.00950	0.00940	0.00950	0.00950	0.00940	0.00950	3.51E-05	1.55E+00	0.00573

[a] = (Stdev(for k^{pfo})/k^{pfo})*k_{2H}

Long time kinetic run (> 12 half-lives)	Six kinetic runs (3 half-lives) together with the averaged data	First-order kinetic fit of the average data
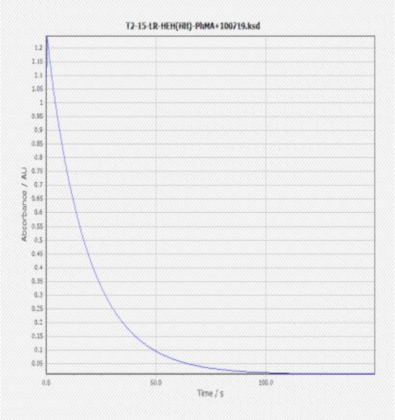	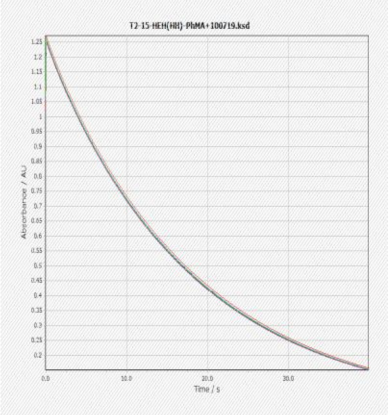	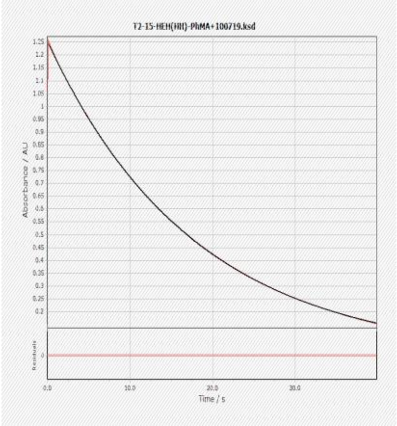
15°C H		

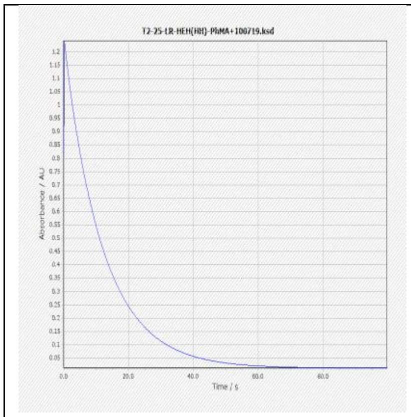 T2-25-LR-HEH(HH)-PhMA+100719.ksd<br>Absorbance / AU<br>Time / s	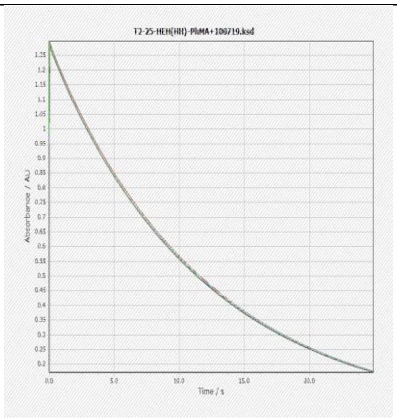 T2-25-HEH(HH)-PhMA+100719.ksd<br>Absorbance / AU<br>Time / s	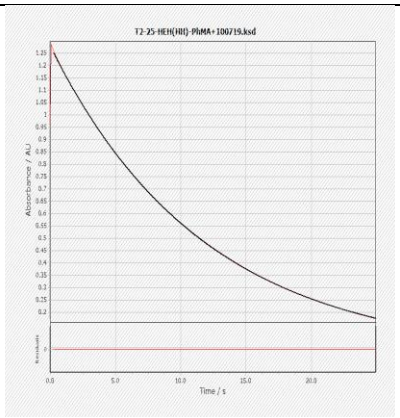 T2-25-HEH(HH)-PhMA+100719.ksd<br>Absorbance / AU<br>Time / s
25℃ H		
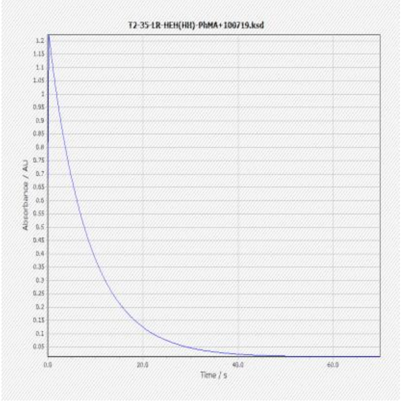 T2-35-LR-HEH(HH)-PhMA+100719.ksd<br>Absorbance / AU<br>Time / s	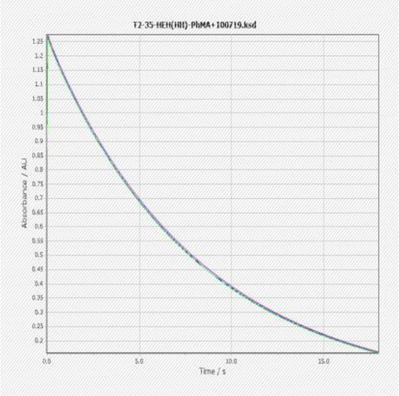 T2-35-HEH(HH)-PhMA+100719.ksd<br>Absorbance / AU<br>Time / s	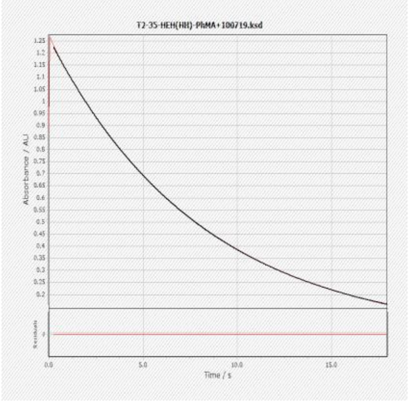 T2-35-HEH(HH)-PhMA+100719.ksd<br>Absorbance / AU<br>Time / s
35℃ H		
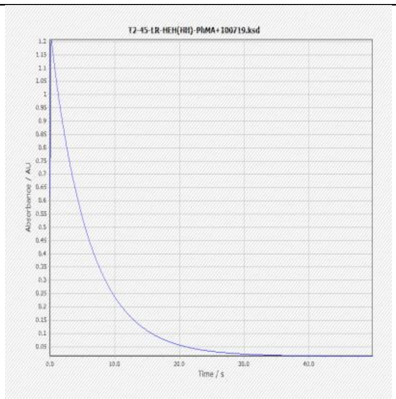 T2-45-LR-HEH(HH)-PhMA+100719.ksd<br>Absorbance / AU<br>Time / s	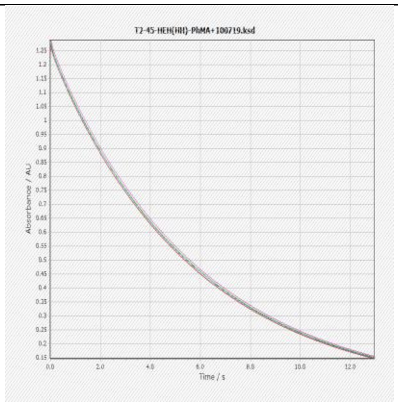 T2-45-HEH(HH)-PhMA+100719.ksd<br>Absorbance / AU<br>Time / s	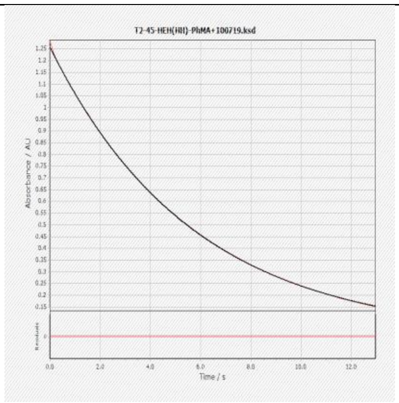 T2-45-HEH(HH)-PhMA+100719.ksd<br>Absorbance / AU<br>Time / s
45℃ H		

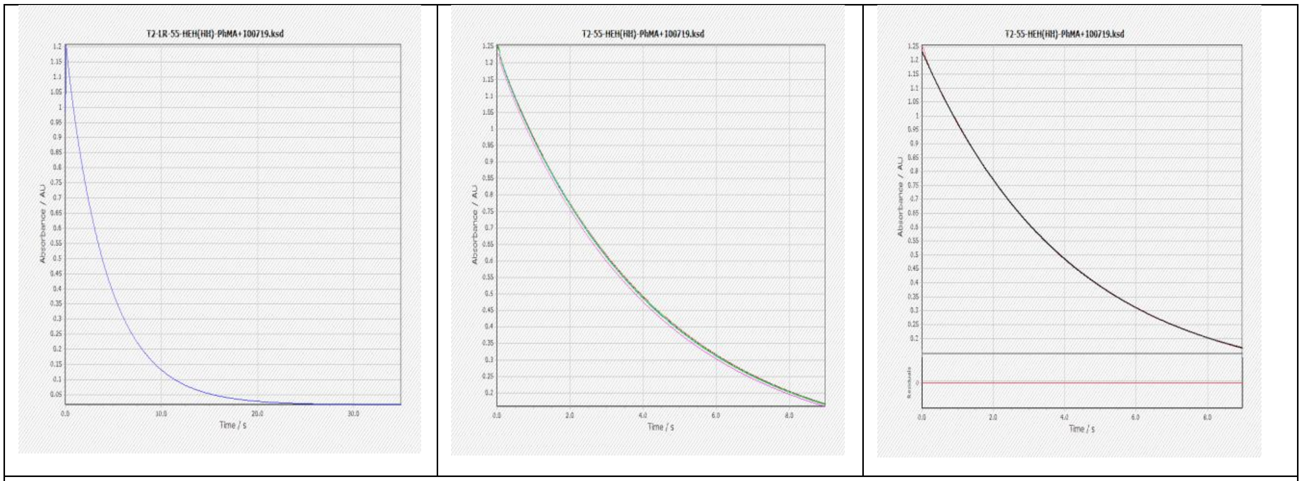
T2-LR-55-HEH(HH)-PhMA+100719.ksd
T2-55-HEH(HH)-PhMA+100719.ksd
T2-55-HEH(HH)-PhMA+100719.ksd
Absorbance / AU
Time / s

55℃ H

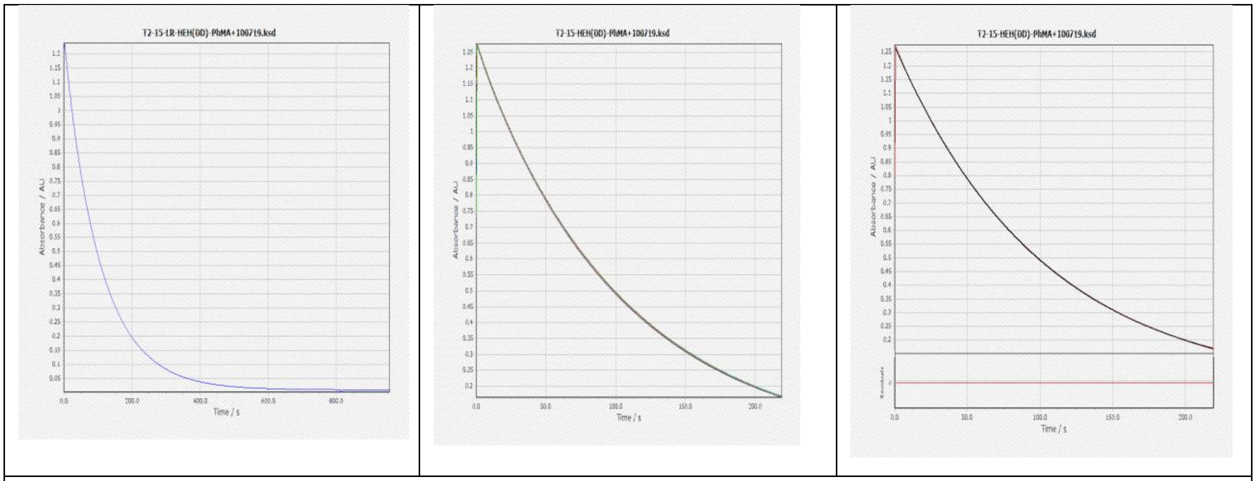
T2-15-LR-HEH(DD)-PhMA+100719.ksd
T2-15-HEH(DD)-PhMA+100719.ksd
T2-15-HEH(DD)-PhMA+100719.ksd
Absorbance / AU
Time / s

15℃ D

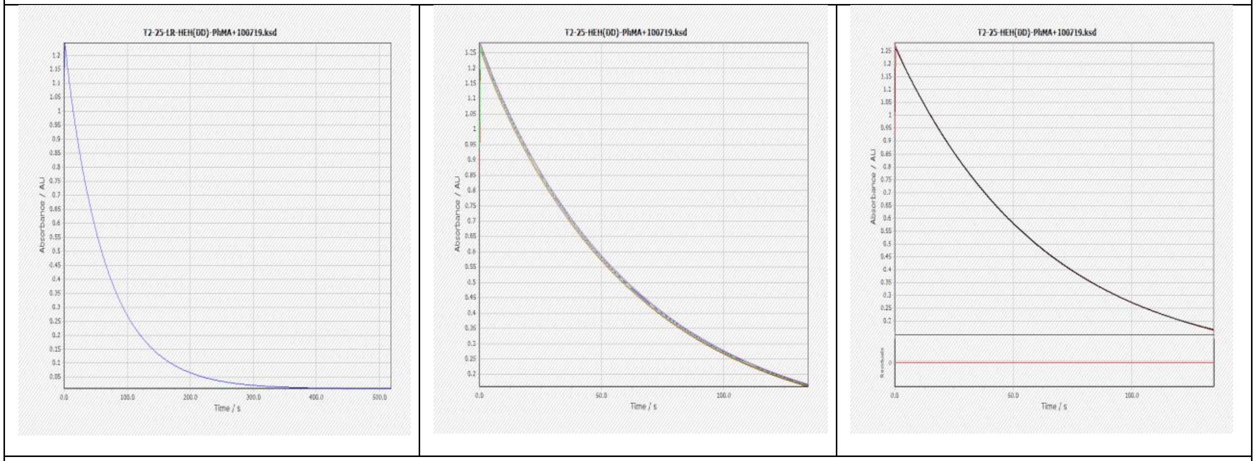
T2-25-LR-HEH(DD)-PhMA+100719.ksd
T2-25-HEH(DD)-PhMA+100719.ksd
T2-25-HEH(DD)-PhMA+100719.ksd
Absorbance / AU
Time / s

25℃ D

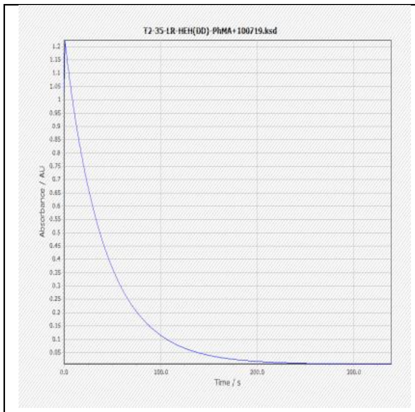
T2-35-LR-HEH(DD)-PhMA+100719.ksd
Absorbance / AU
Time / s

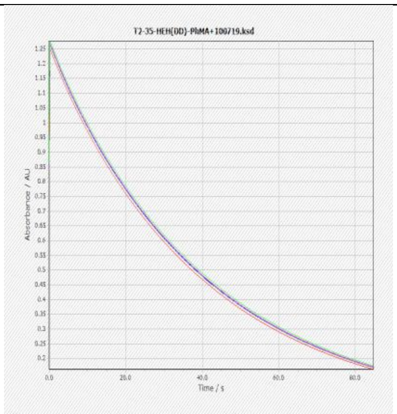
T2-35-HEH(DD)-PhMA+100719.ksd
Absorbance / AU
Time / s

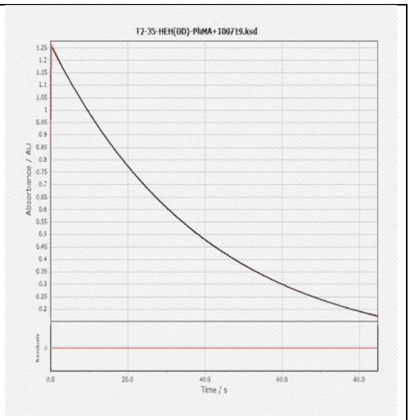
T2-35-HEH(DD)-PhMA+100719.ksd
Absorbance / AU
Time / s

35℃ D

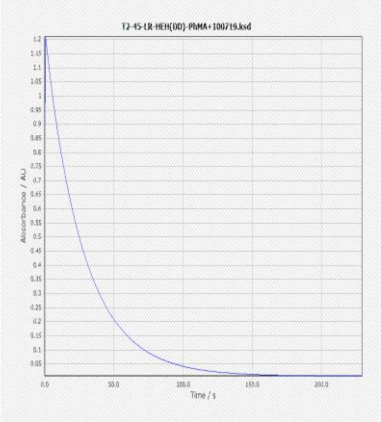
T2-45-LR-HEH(DD)-PhMA+100719.ksd
Absorbance / AU
Time / s

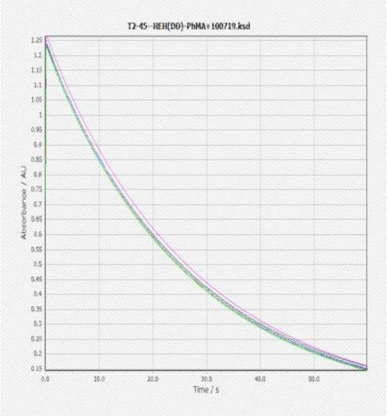
T2-45--HEH(DD)-PhMA+100719.ksd
Absorbance / AU
Time / s

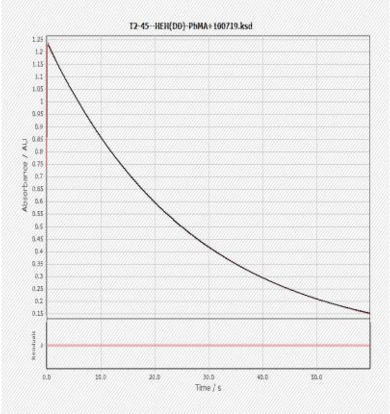
T2-45--HEH(DD)-PhMA+100719.ksd
Absorbance / AU
Time / s

45℃ D

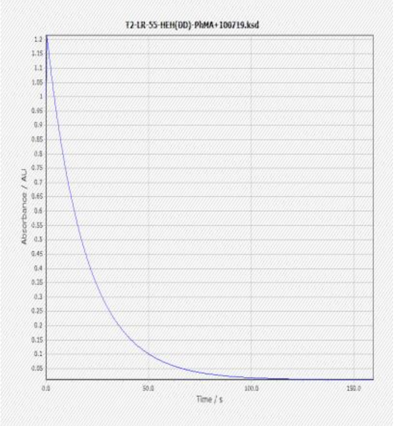
T2-LR-55-HEH(DD)-PhMA+100719.ksd
Absorbance / AU
Time / s

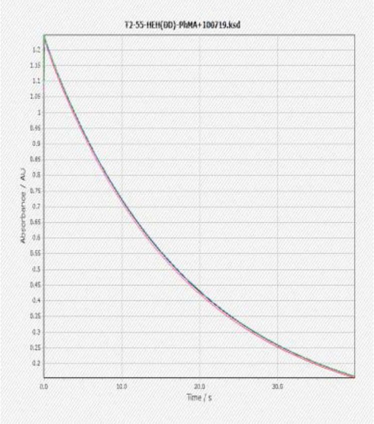
T2-55-HEH(DD)-PhMA+100719.ksd
Absorbance / AU
Time / s

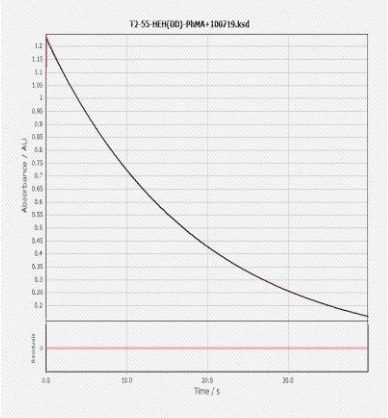
T2-55-HEH(DD)-PhMA+100719.ksd
Absorbance / AU
Time / s

55℃ D

Day 2 data (October 8, 2019)

	Pseudo-first-order rate constants k^{pfo} (s^{-1})									
**Temp (°C)**	**Trial H1**	**Trial H2**	**Trial H3**	**Trial H4**	**Trial H5**	**Trial H6**	**Average k_H^{pfo} (s^{-1})**	**Stdev**	**k_{2H} ($M^{-1}s^{-1}$)**	**Stdev [a]**
55	0.2370	0.2318	0.2348	0.2347	0.2378	0.2349	0.2352	0.002099	3.92E+01	0.34984
45	0.1722	0.1724	0.1707	0.1740	0.1707	0.1721	0.1720	0.001245	2.87E+01	0.20750
35	0.1221	0.1203	0.1185	0.1194	0.1191	0.1199	0.1199	0.001234	2.00E+01	0.20569
25	0.0821	0.0820	0.0817	0.0821	0.0828	0.0828	0.0822	0.000457	1.37E+01	0.07621
15	0.0542	0.0544	0.0548	0.0544	0.0542	0.0544	0.0544	0.000234	9.07E+00	0.03904
**Temp (°C)**	**Trial D1**	**Trial D2**	**Trial D3**	**Trial D4**	**Trial D5**	**Trial D6**	**Average k_D^{pfo} (s^{-1})**	**Stdev**	**k_{2D} ($M^{-1}s^{-1}$)**	**Stdev [a]**
55	0.0545	0.0535	0.0543	0.0541	0.0545	0.0542	0.0542	0.000348	8.97E+00	0.05754
45	0.0372	0.0369	0.0368	0.0367	0.0368	0.0366	0.0368	0.000209	6.10E+00	0.03456
35	0.0245	0.0243	0.0245	0.0241	0.0240	0.0241	0.0243	0.000205	4.01E+00	0.03389
25	0.0158	0.0156	0.0159	0.0158	0.0158	0.0157	0.0158	9.85E-05	2.61E+00	0.01630
15	0.00960	0.00970	0.00950	0.00950	0.00950	0.00960	0.00960	6.94E-05	1.58E+00	0.01148

[a] = (Stdev(for k^{pfo})/k^{pfo})*k_{2H}

Long time kinetic run (> 12 half-lives)	Six kinetic runs (3 half-lives) together with the averaged data	First-order kinetic fit of the average data
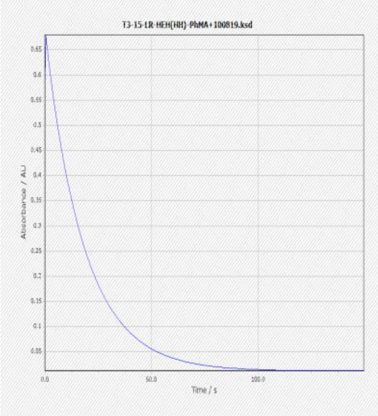	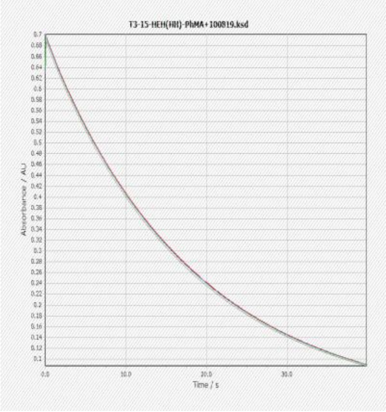	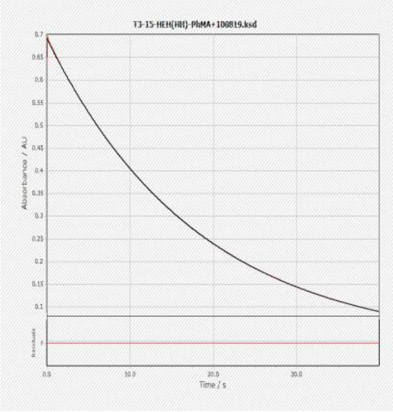
15°C H		
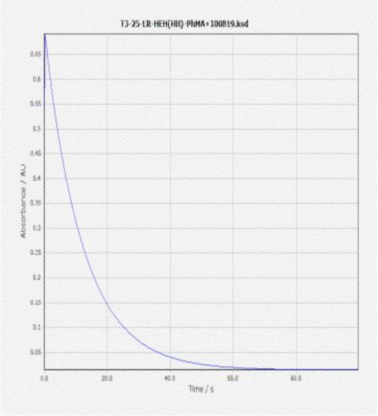	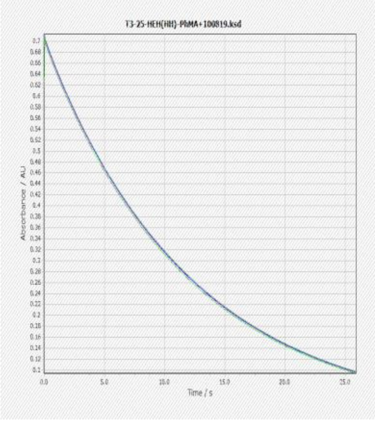	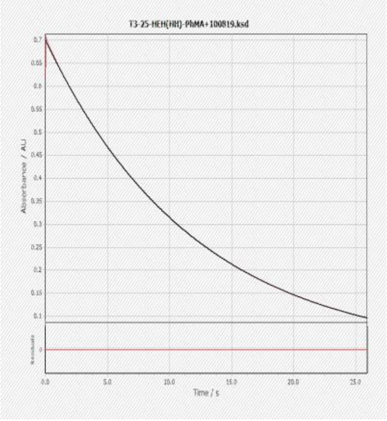
25°C H		

<table>
<tr><td>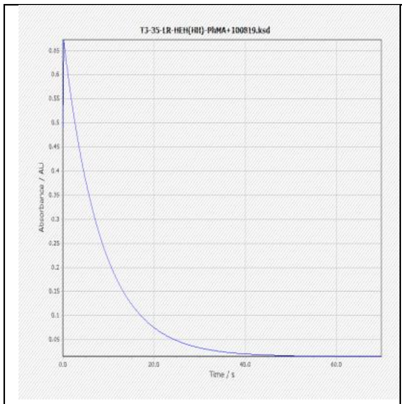
</td><td>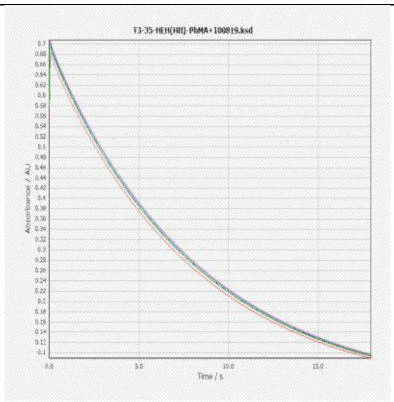
</td><td>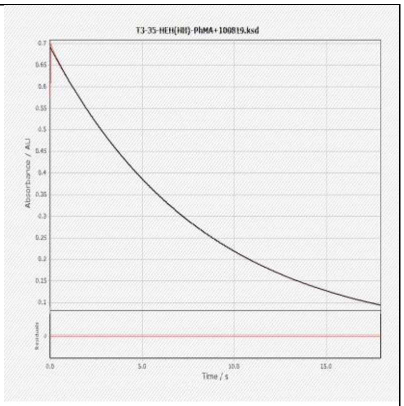
</td></tr>
<tr><td colspan="3">35℃ H</td></tr>
<tr><td>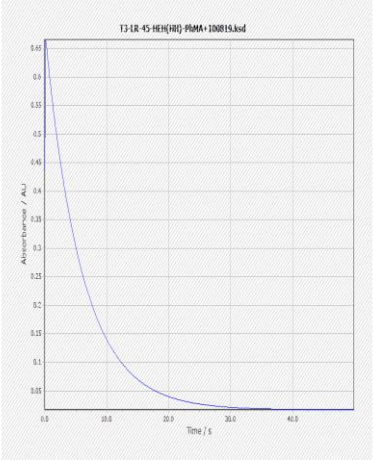
</td><td>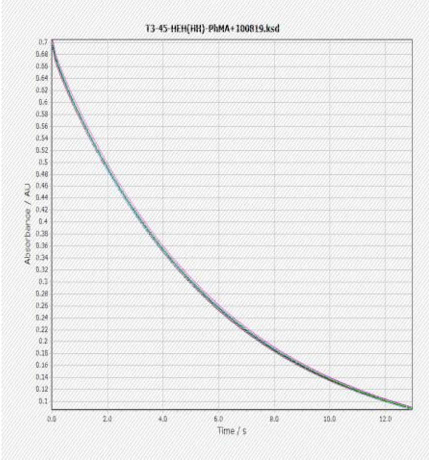
</td><td>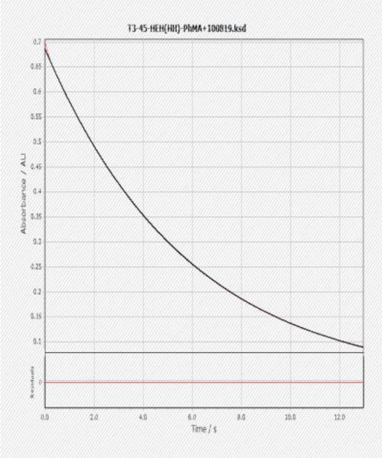
</td></tr>
<tr><td colspan="3">45℃ H</td></tr>
<tr><td>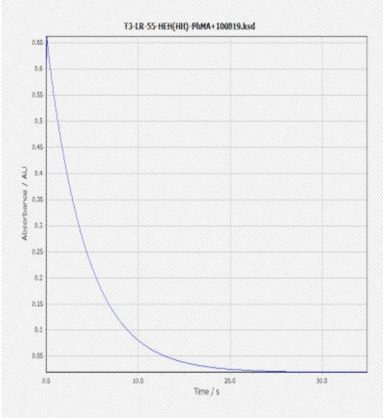
</td><td>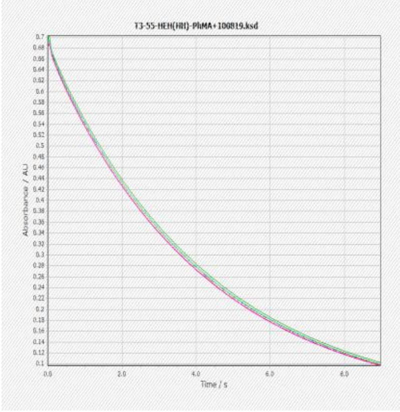
</td><td>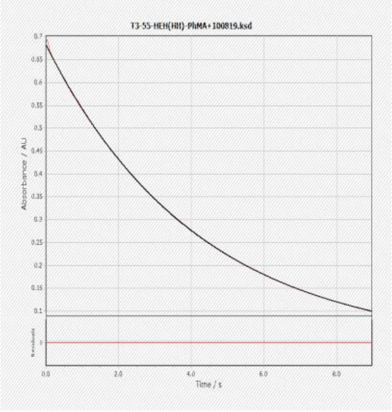
</td></tr>
<tr><td colspan="3">55℃ H</td></tr>
</table>

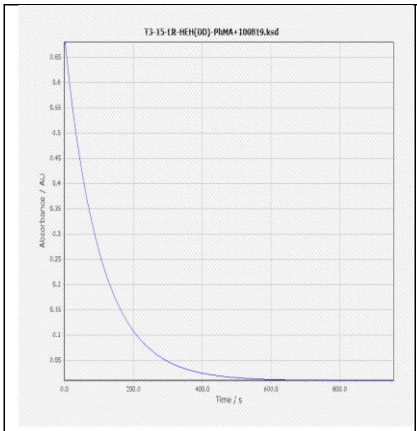
T3-15-LR-HEH(DD)-PhMA+100819.ksd
Absorbance / AU
Time / s
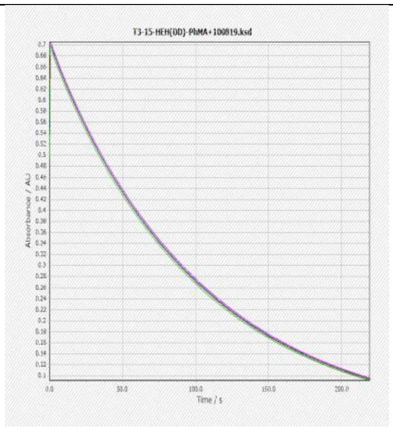
T3-15-HEH(DD)-PhMA+100819.ksd
Absorbance / AU
Time / s
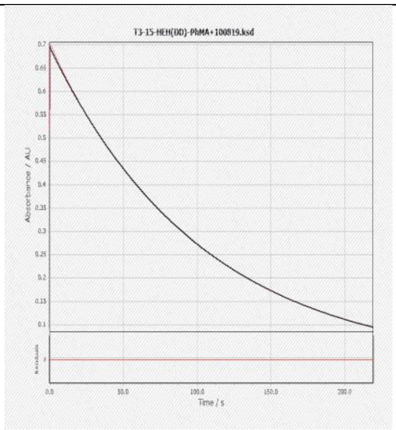
T3-15-HEH(DD)-PhMA+100819.ksd
Absorbance / AU
Time / s

15℃ D

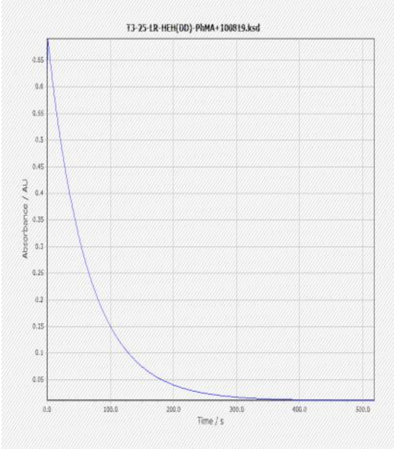
T3-25-LR-HEH(DD)-PhMA+100819.ksd
Absorbance / AU
Time / s
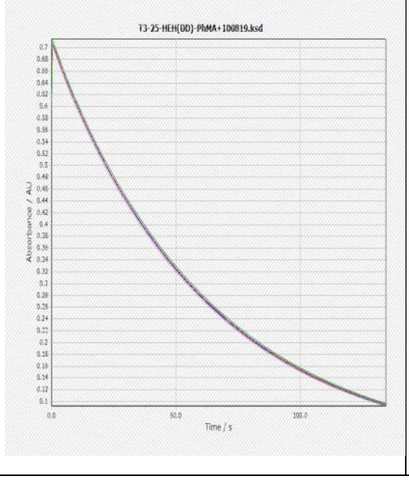
T3-25-HEH(DD)-PhMA+100819.ksd
Absorbance / AU
Time / s
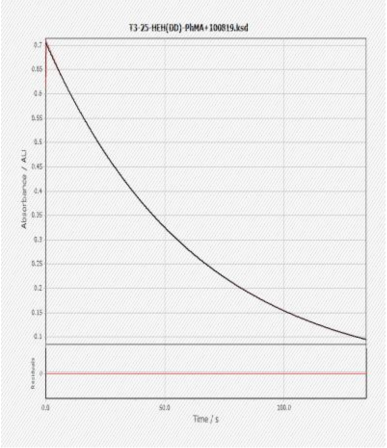
T3-25-HEH(DD)-PhMA+100819.ksd
Absorbance / AU
Time / s

25℃ D

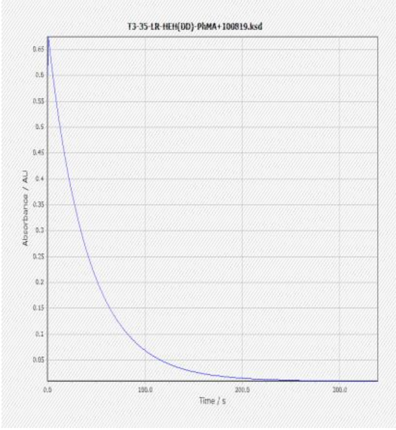
T3-35-LR-HEH(DD)-PhMA+100819.ksd
Absorbance / AU
Time / s
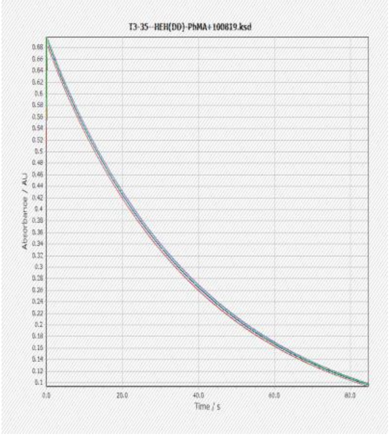
T3-35--HEH(DD)-PhMA+100819.ksd
Absorbance / AU
Time / s
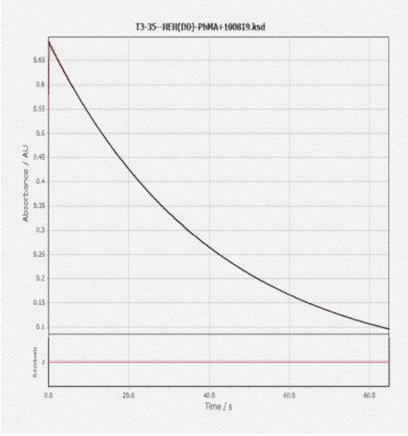
T3-35--HEH(DD)-PhMA+100819.ksd
Absorbance / AU
Time / s

35℃ D

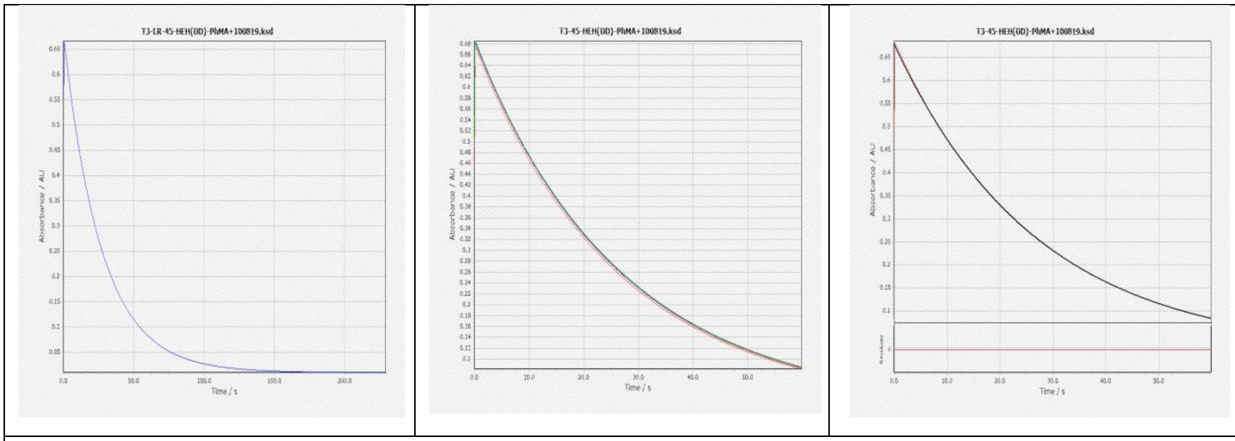

45°C D

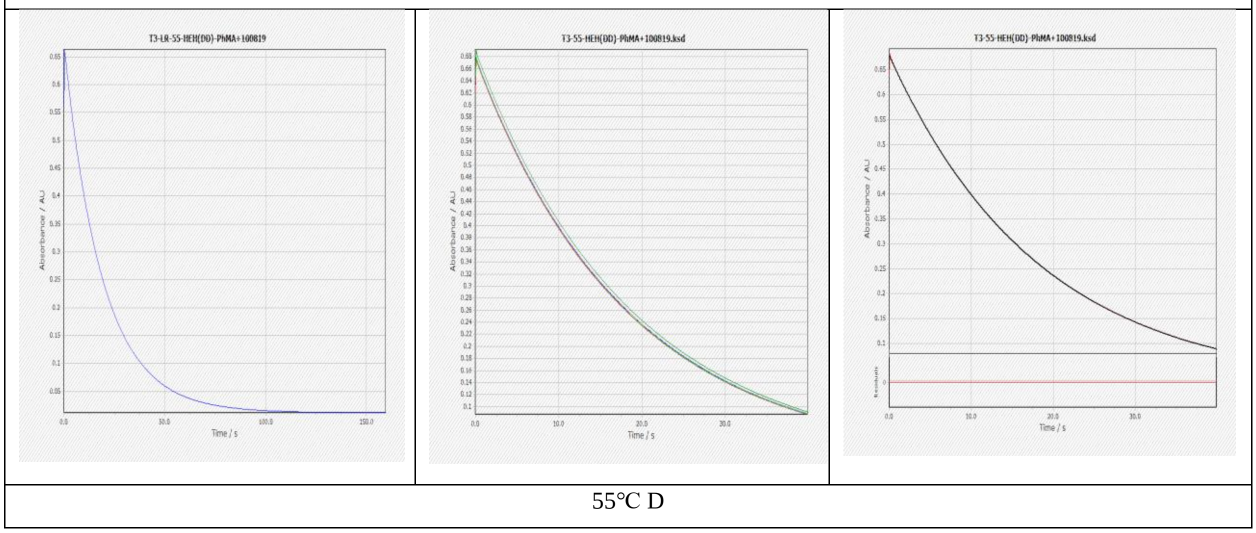

55°C D

Day 3 data (October 9, 2019)

	Pseudo-first-order rate constants k^{pfo} (s^{-1})									
**Temp (°C)**	**Trial H1**	**Trial H2**	**Trial H3**	**Trial H4**	**Trial H5**	**Trial H6**	**Average k_H^{pfo} (s^{-1})**	**Stdev**	**k_{2H} ($M^{-1}s^{-1}$)**	**Stdev [a]**
55	0.2328	0.2333	0.2335	0.2332	0.2345	0.2340	0.2335	0.0006	38.9222	0.1022
45	0.1661	0.1681	0.1686	0.1666	0.1680	0.1679	0.1676	0.0010	27.9277	0.1638
35	0.1181	0.1186	0.1188	0.1181	0.1181	0.1186	0.1184	0.0003	19.7294	0.0569
25	0.0812	0.0812	0.0816	0.0821	0.0826	0.0821	0.0818	0.0005	13.6318	0.0915
15	0.0536	0.0533	0.0540	0.0532	0.0539	0.0536	0.0536	0.0003	8.9324	0.0524
**Temp (°C)**	**Trial D1**	**Trial D2**	**Trial D3**	**Trial D4**	**Trial D5**	**Trial D6**	**Average k_D^{pfo} (s^{-1})**	**Stdev**	**k_{2D} ($M^{-1}s^{-1}$)**	**Stdev [a]**
55	0.0535	0.0539	0.0533	0.0537	0.0534	0.0539	0.0536	0.000245	8.91E+00	0.04072
45	0.0364	0.0364	0.0364	0.0359	0.0364	0.0363	0.0363	0.00019	6.03E+00	0.03157
35	0.0244	0.0243	0.0245	0.0241	0.0242	0.0244	0.0243	0.00016	4.04E+00	0.02665
25	0.0156	0.0156	0.0156	0.0156	0.0155	0.0157	0.0156	5.3E-05	2.59E+00	0.00881
15	0.00970	0.00970	0.00970	0.00960	0.00980	0.00970	0.00970	4.25E-05	1.61E+00	0.00707

[a] = (Stdev(for k^{pfo})/k^{pfo})*k_{2H}

Long time kinetic run (> 12 half-lives)	Six kinetic runs (3 half-lives) together with the averaged data	First-order kinetic fit of the average data
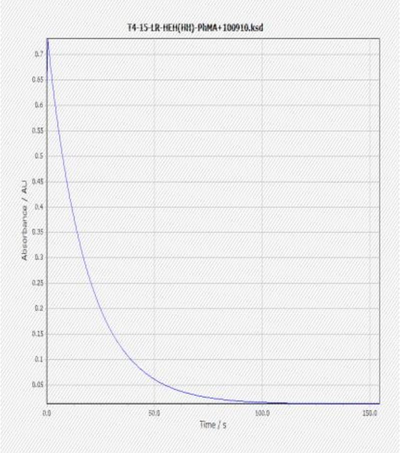	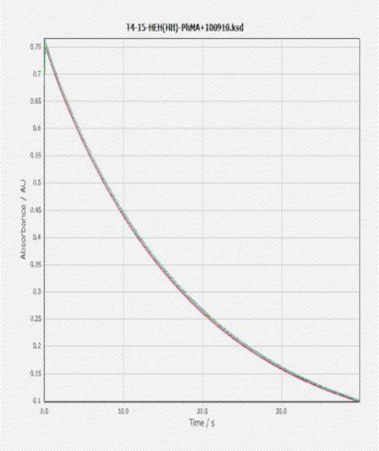	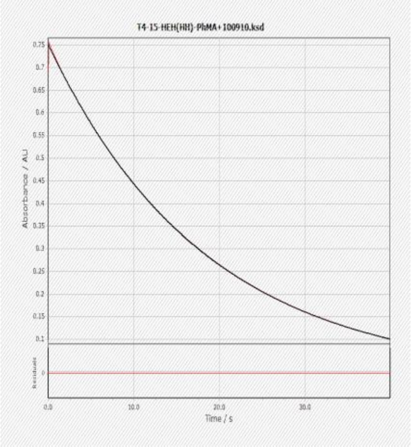
15℃ H		
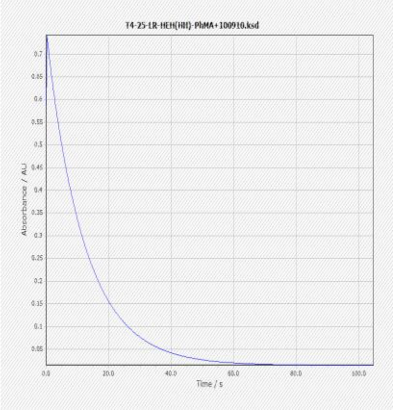	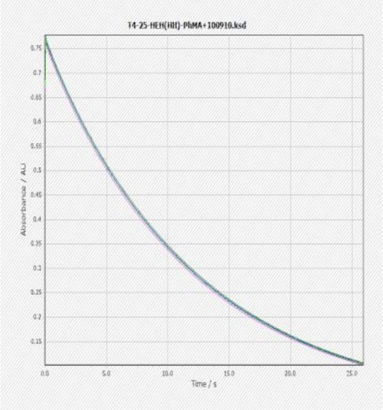	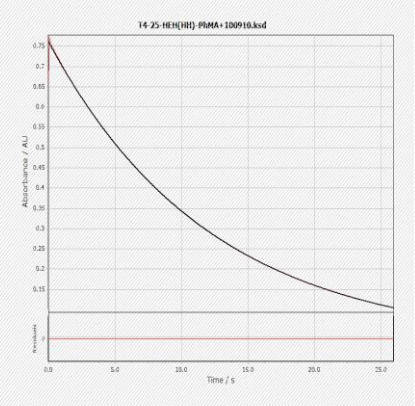
25℃ H		
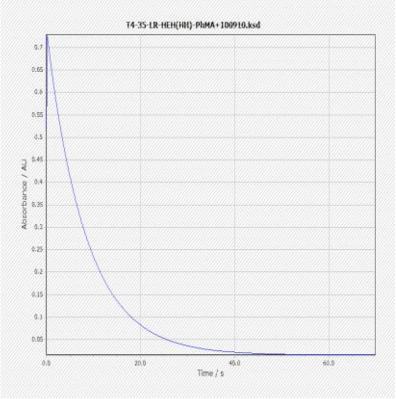	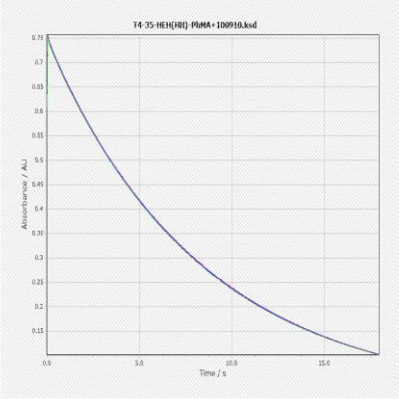	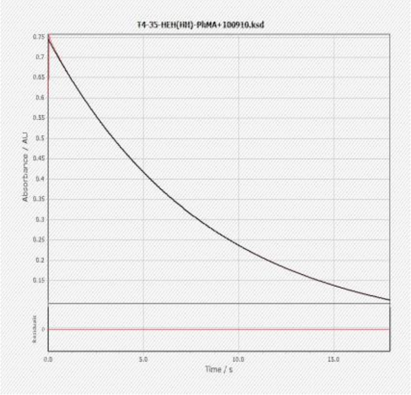
35℃ H		

<table>
<tr>
<td>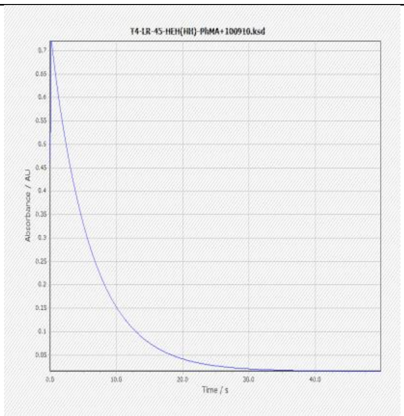
</td>
<td>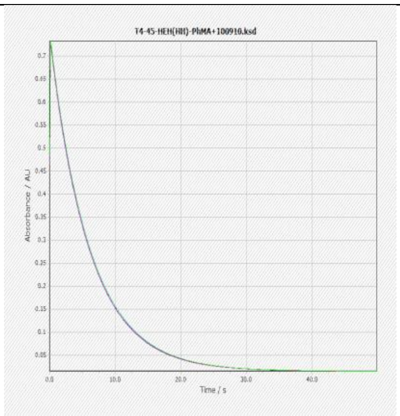
</td>
<td>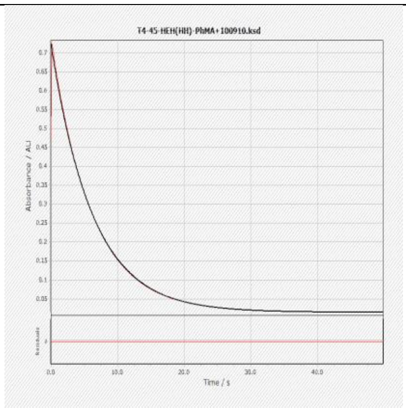
</td>
</tr>
<tr><td colspan="3">45℃ H</td></tr>
<tr>
<td>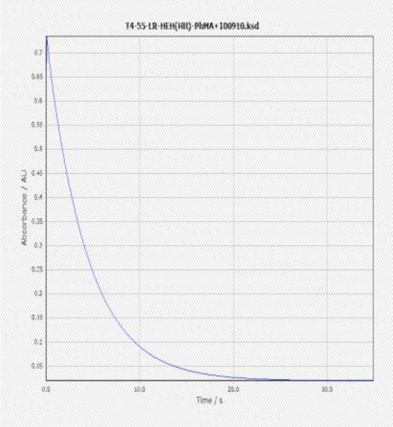
</td>
<td>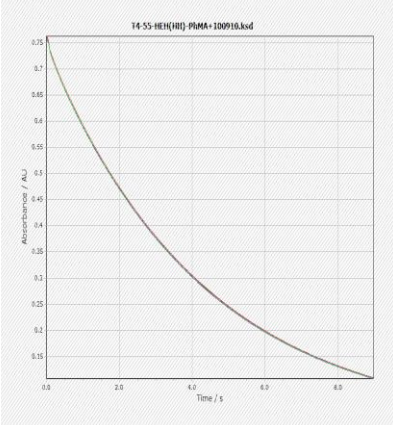
</td>
<td>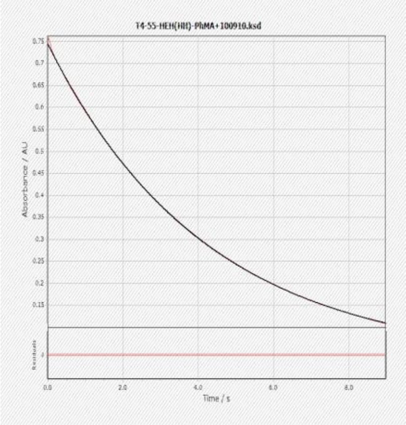
</td>
</tr>
<tr><td colspan="3">55℃ H</td></tr>
</table>

<table>
<tr>
<td>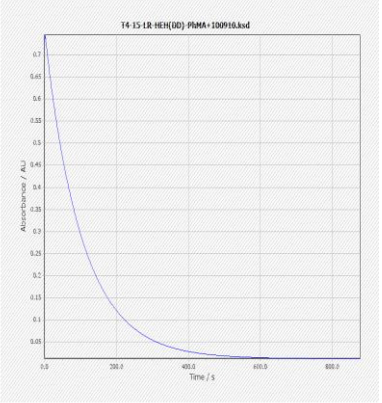
</td>
<td>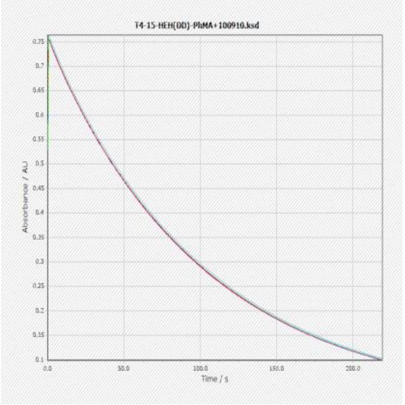
</td>
<td>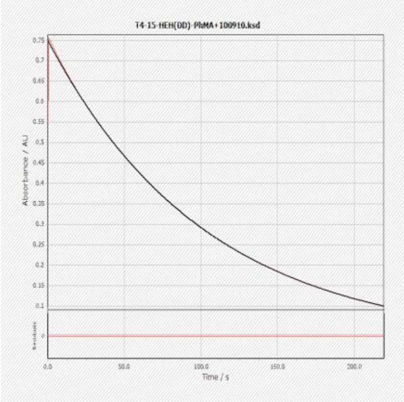
</td>
</tr>
<tr><td colspan="3">15℃ D</td></tr>
</table>

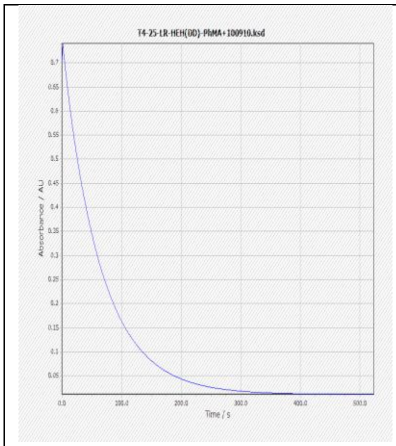
T4-25-LR-HEH(DD)-PhMA+100910.ksd
Absorbance / AU
Time / s

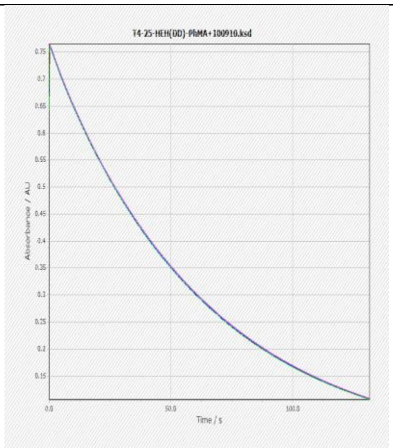
T4-25-HEH(DD)-PhMA+100910.ksd
Absorbance / AU
Time / s

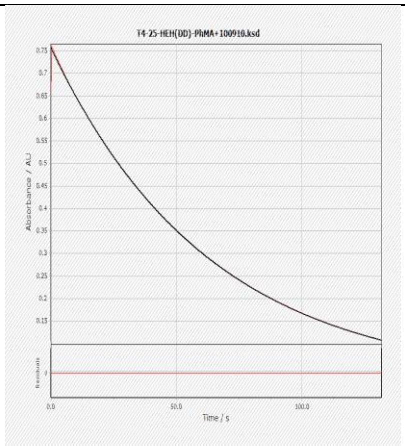
T4-25-HEH(DD)-PhMA+100910.ksd
Absorbance / AU
Time / s

25℃ D

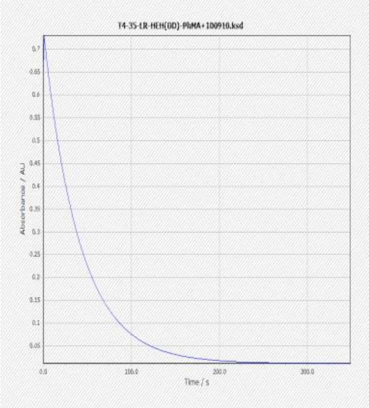
T4-35-LR-HEH(DD)-PhMA+100910.ksd
Absorbance / AU
Time / s

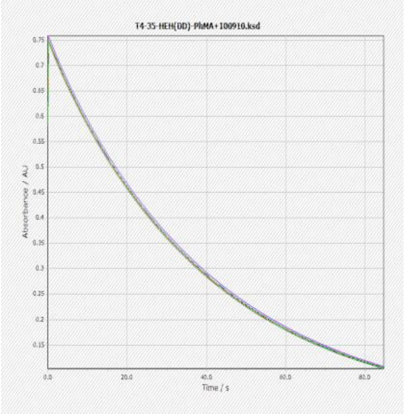
T4-35-HEH(DD)-PhMA+100910.ksd
Absorbance / AU
Time / s

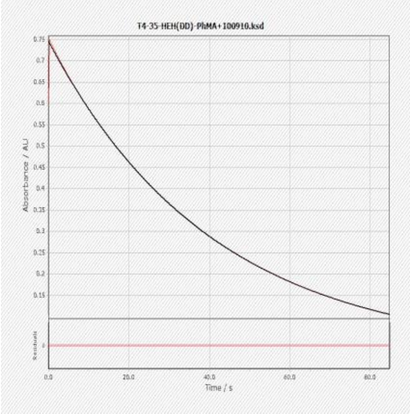
T4-35-HEH(DD)-PhMA+100910.ksd
Absorbance / AU
Time / s

35℃ D

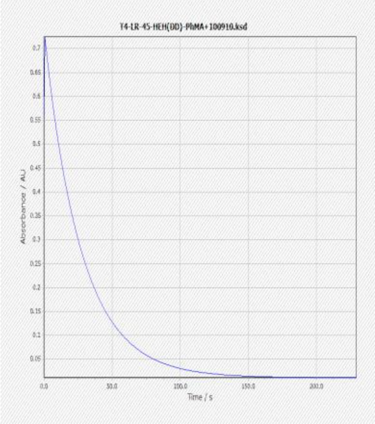
T4-LR-45-HEH(DD)-PhMA+100910.ksd
Absorbance / AU
Time / s

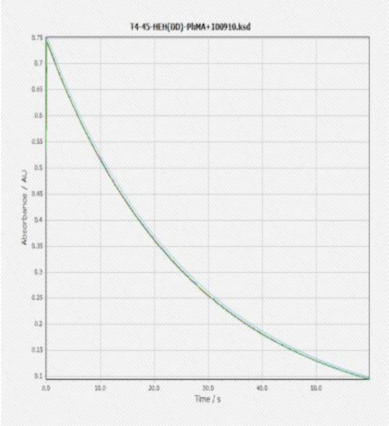
T4-45-HEH(DD)-PhMA+100910.ksd
Absorbance / AU
Time / s

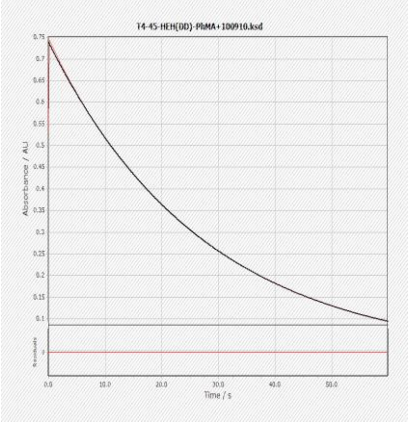
T4-45-HEH(DD)-PhMA+100910.ksd
Absorbance / AU
Time / s

45℃ D

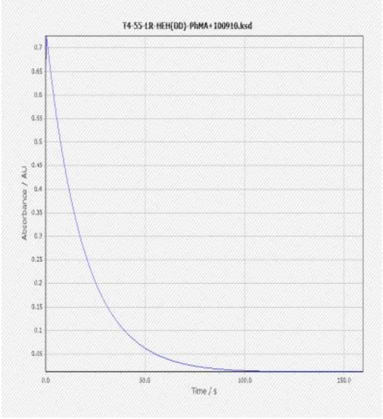 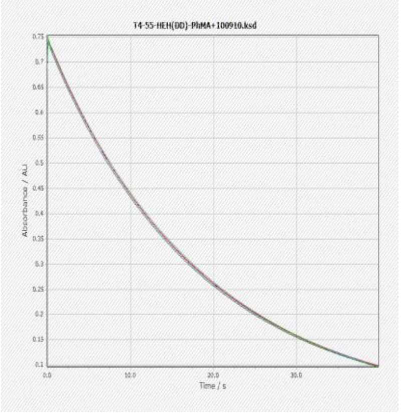 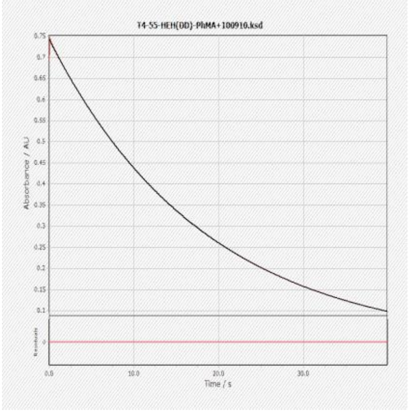

55℃ D

Primary kinetic data for the rate constants in Table S10

Day 1 data (June 21, 2021)

	Pseudo-first-order rate constants k^{pfo} (s^{-1})									
**Temp (°C)**	**Trial H1**	**Trial H2**	**Trial H3**	**Trial H4**	**Trial H5**	**Trial H6**	**Average k_H^{pfo} (s^{-1})**	**Stdev**	**k_{2H} (M^{-1}s^{-1})**	**Stdev [a]**
55	0.3312	0.3320	0.3369	0.3353	0.3390	0.3331	0.0802	0.0816	55.7603	0.5017
45	0.2425	0.2446	0.2450	0.2442	0.2427	0.2445	0.0560	0.0554	40.6489	0.1766
35	0.1798	0.1798	0.1797	0.1835	0.1820	0.1823	0.0375	0.0386	30.1933	0.2751
25	0.1247	0.1246	0.1246	0.1261	0.1271	0.1270	0.0244	0.0245	20.9469	0.2010
15	0.0842	0.0849	0.0854	0.0856	0.0858	0.0859	0.0152	0.0159	14.2161	0.1066

**Temp (°C)**	**Trial D1**	**Trial D2**	**Trial D3**	**Trial D4**	**Trial D5**	**Trial D6**	**Average k_D^{pfo} (s^{-1})**	**Stdev**	**k_{2D} (M^{-1}s^{-1})**	**Stdev [a]**
55	0.08024	0.08163	0.08200	0.08159	0.08087	0.08123	0.0813	0.00063	1.33E+01	0.10297
45	0.0560	0.0554	0.0561	0.0556	0.0563	0.0558	0.0559	0.000318	9.12E+00	0.05199
35	0.0375	0.0386	0.0385	0.0383	0.0380	0.0383	0.0382	0.000429	6.24E+00	0.07012
25	0.0244	0.0245	0.0245	0.0245	0.0247	0.0247	0.0245	0.000143	4.01E+00	0.02337
15	0.0152	0.0159	0.0159	0.0160	0.0162	0.0158	0.0158	0.00033	2.59E+00	0.05385

[a] = (Stdev(for k^{pfo})/k^{pfo})*k_{2H}

<table>
<tr><td>Six kinetic runs together with the averaged data</td><td>First-order kinetic fit of the averaged data as an example</td></tr>
<tr><td>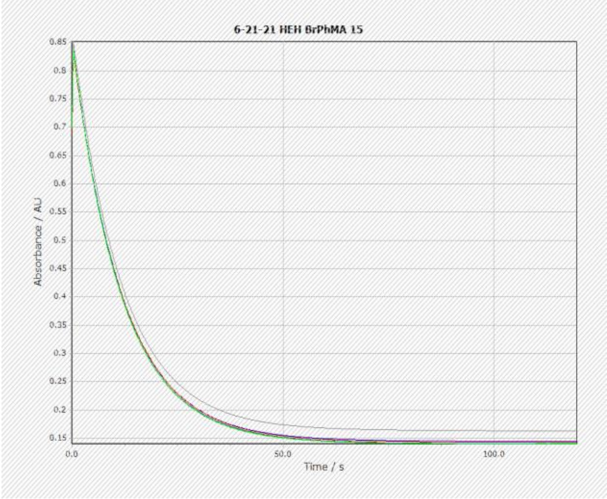
</td><td>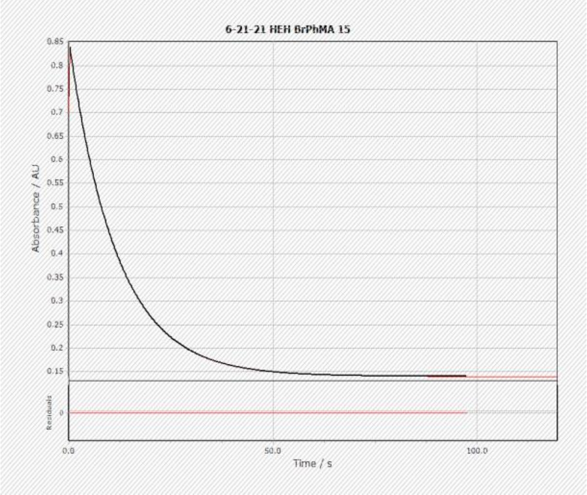
</td></tr>
<tr><td colspan="2">15°C H</td></tr>
<tr><td>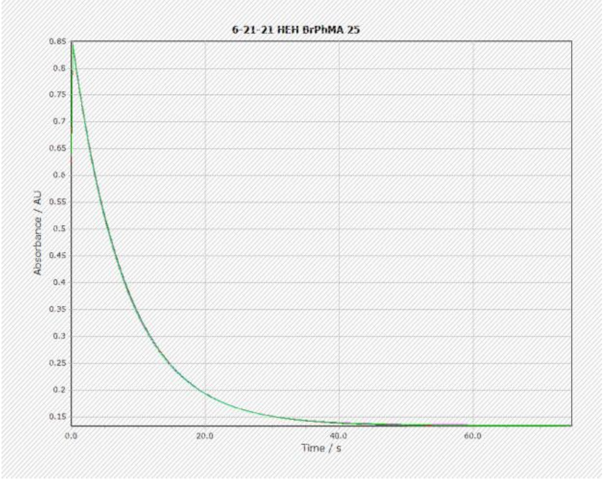
</td><td>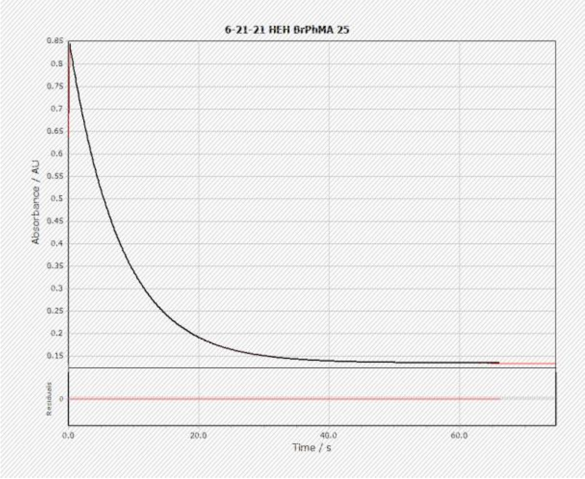
</td></tr>
<tr><td colspan="2">25°C H</td></tr>
<tr><td>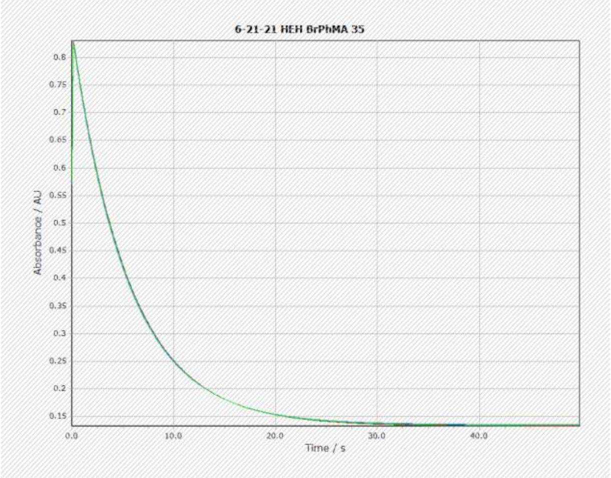
</td><td>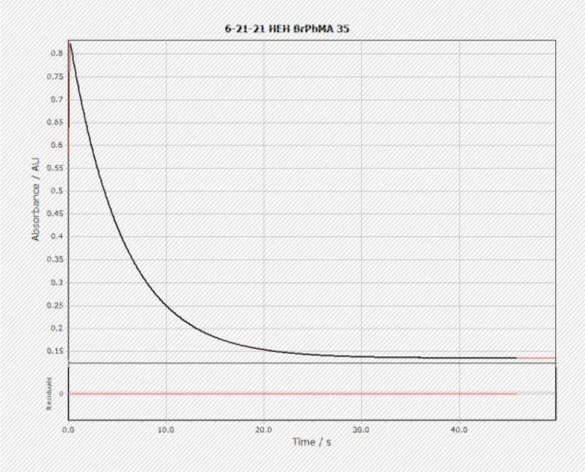
</td></tr>
<tr><td colspan="2">35°C H</td></tr>
</table>

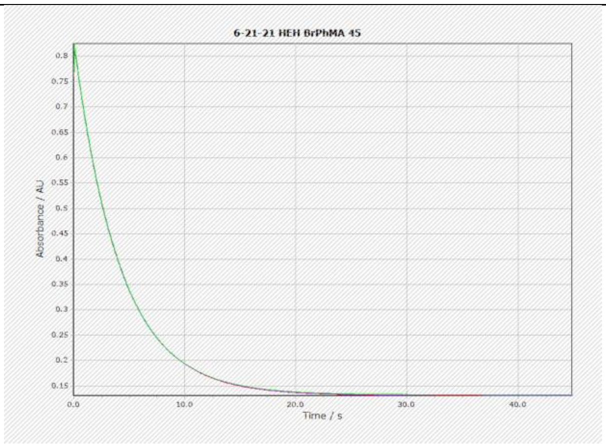
6-21-21 HEH BrPhMA 45
Absorbance / AU
Time / s

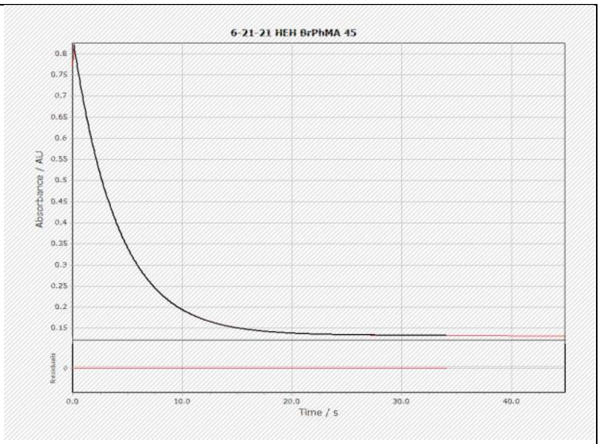
6-21-21 HEH BrPhMA 45
Absorbance / AU
Residuals
Time / s

45℃ H

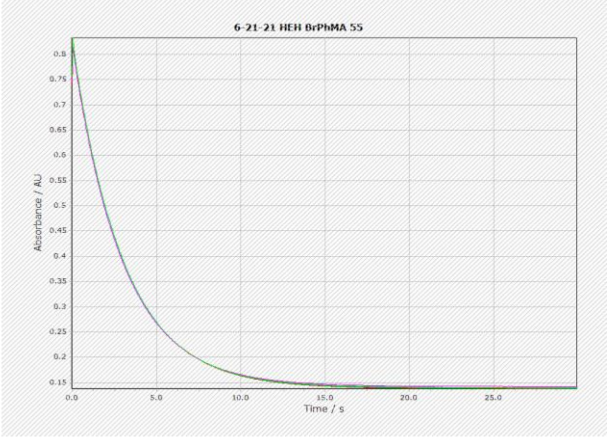
6-21-21 HEH BrPhMA 55
Absorbance / AU
Time / s

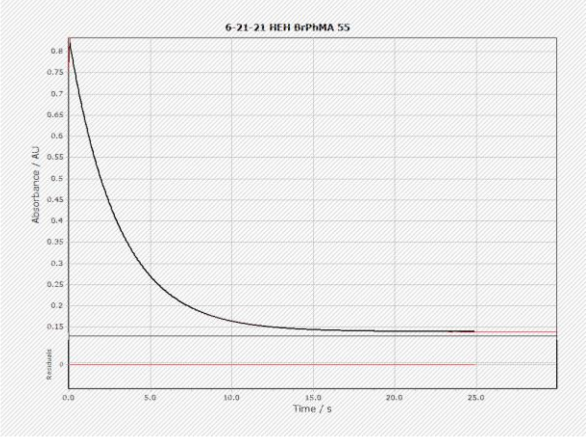
6-21-21 HEH BrPhMA 55
Absorbance / AU
Residuals
Time / s

55℃ H

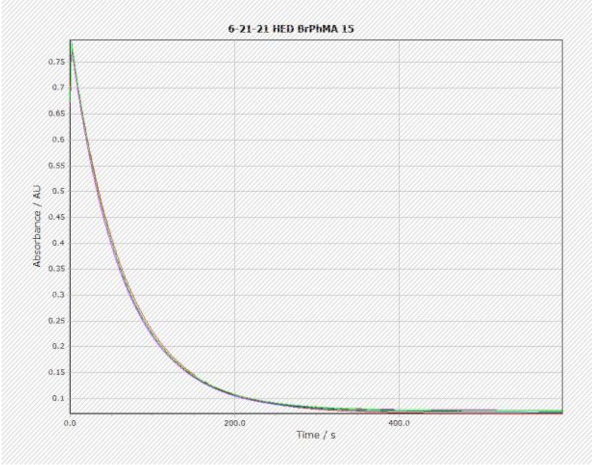
6-21-21 HED BrPhMA 15
Absorbance / AU
Time / s

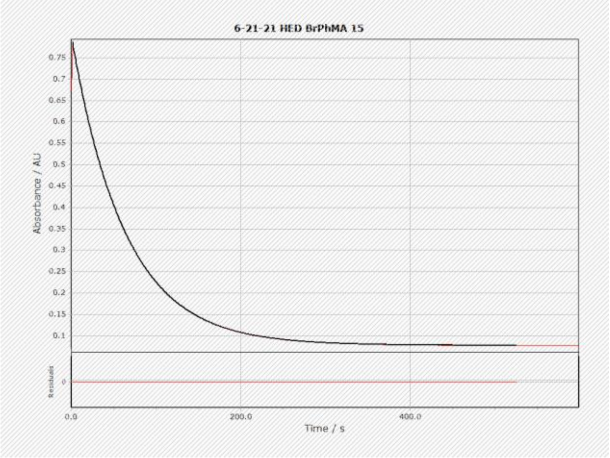
6-21-21 HED BrPhMA 15
Absorbance / AU
Residuals
Time / s

15℃ D

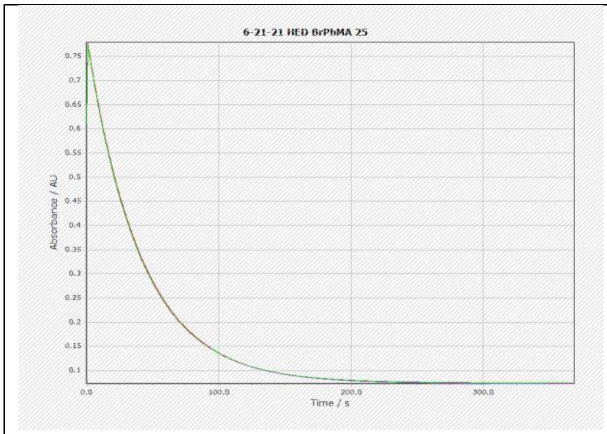

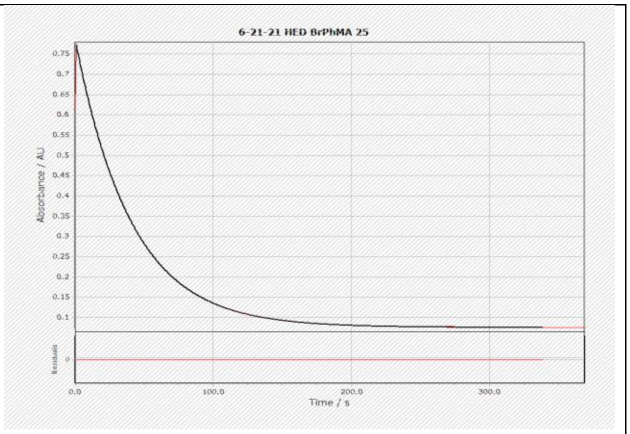

25°C D

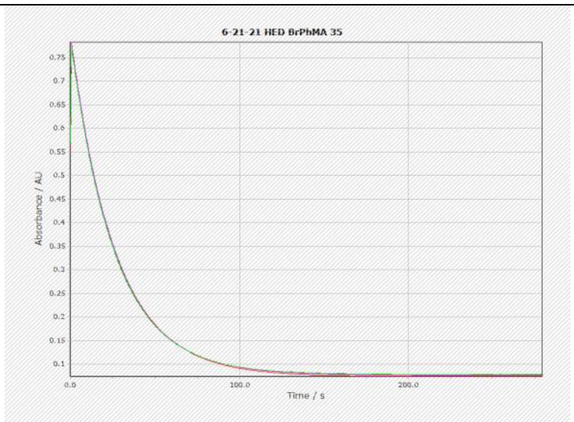

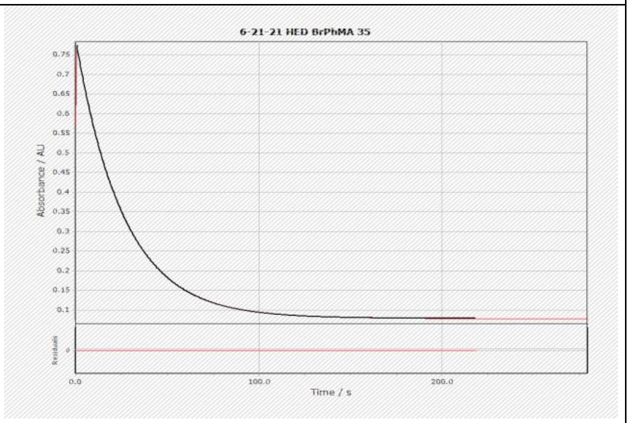

35°C D

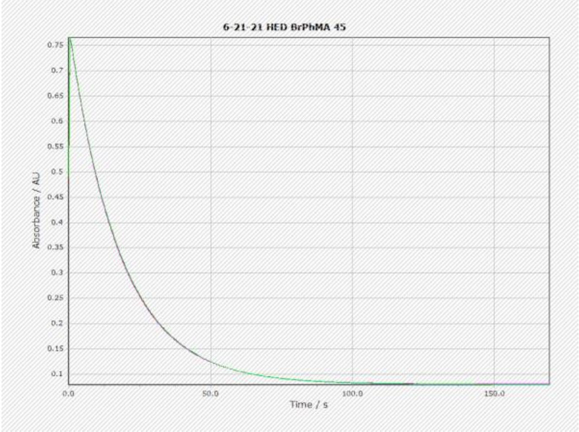

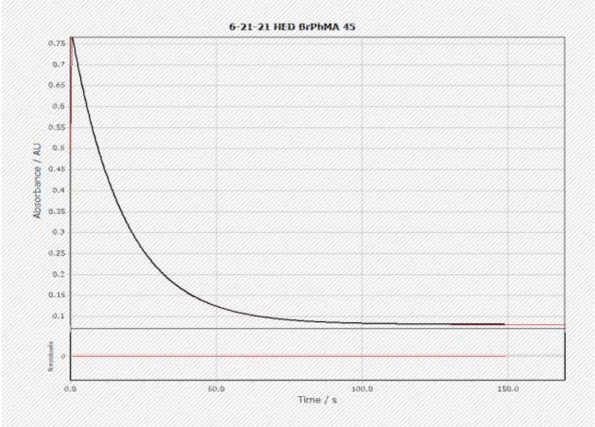

45°C D

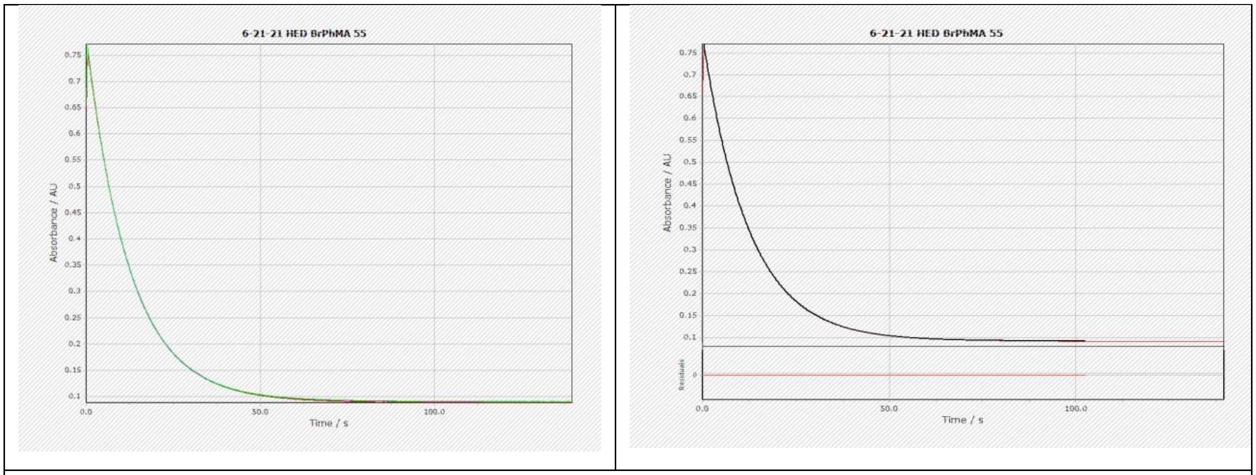

55°C D

Day 2 data (June 23, 2021)

	Pseudo-first-order rate constants k^{pfo} (s^{-1})									
**Temp (°C)**	**Trial H1**	**Trial H2**	**Trial H3**	**Trial H4**	**Trial H5**	**Trial H6**	**Average k_H^{pfo} (s^{-1})**	**Stdev**	**k_{2H} ($M^{-1}s^{-1}$)**	**Stdev [a]**
55	0.3367	0.3365	0.3380	0.3345	0.3359	0.3366	0.3364	0.001138	5.61E+01	0.18973
45	0.2462	0.2463	0.2461	0.2470	0.2459	0.2471	0.2464	0.000475	4.11E+01	0.07922
35	0.1798	0.1793	0.1780	0.1801	0.18343	0.18387	0.1808	0.00236	3.01E+01	0.39341
25	0.1213	0.1244	0.1258	0.1256	0.1259	0.1279	0.1251	0.002195	2.09E+01	0.36579
15	0.0847	0.0857	0.0856	0.0864	0.0861	0.0852	0.0856	0.000628	1.427E+01	0.10467

**Temp (°C)**	**Trial D1**	**Trial D2**	**Trial D3**	**Trial D4**	**Trial D5**	**Trial D6**	**Average k_D^{pfo} (s^{-1})**	**Stdev**	**k_{2D} ($M^{-1}s^{-1}$)**	**Stdev [a]**
55	0.0818	0.0817	0.0808	0.0812	0.0806	0.0803	0.0811	0.000614	1.342E+01	0.10163
45	0.0552	0.0562	0.0566	0.0565	0.0566	0.0567	0.0563	0.000572	9.32E+00	0.09463
35	0.0378	0.0381	0.0378	0.0380	0.0378	0.0377	0.0379	0.000136	6.26E+00	0.02255
25	0.0244	0.0242	0.0240	0.0246	0.0244	0.0243	0.0243	0.000212	4.02E+00	0.03511
15	0.0155	0.0158	0.0158	0.0158	0.0155	0.0156	0.0157	0.000147	2.59E+00	0.02440

[a] = (Stdev(for k^{pfo})/k^{pfo})*k_{2H}

<table>
<tr><td>Six kinetic runs together with the averaged data</td><td>First-order kinetic fit of the averaged data as an example</td></tr>
<tr><td>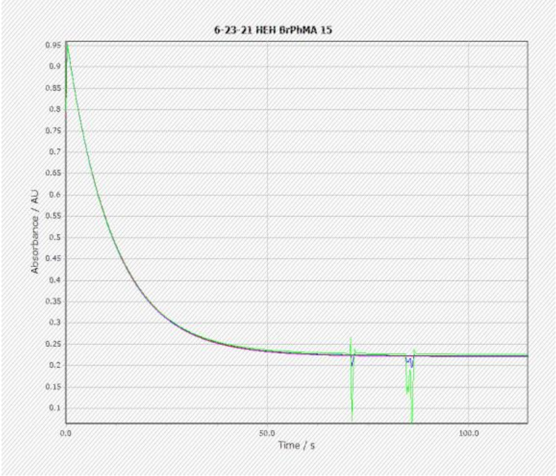
</td><td>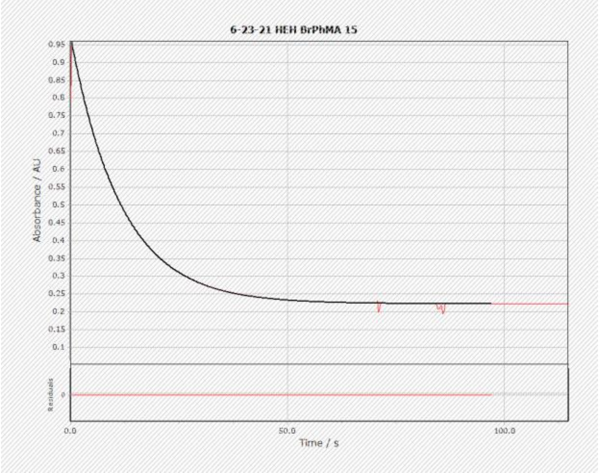
</td></tr>
<tr><td colspan="2">15℃ H</td></tr>
<tr><td>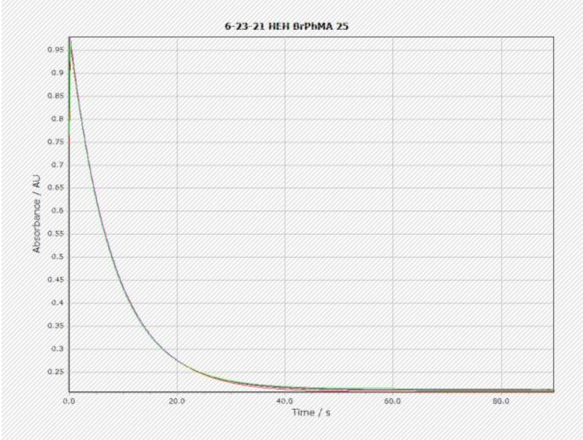
</td><td>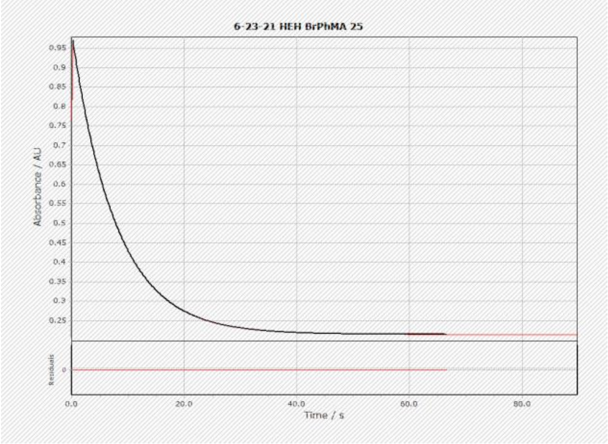
</td></tr>
<tr><td colspan="2">25℃ H</td></tr>
<tr><td>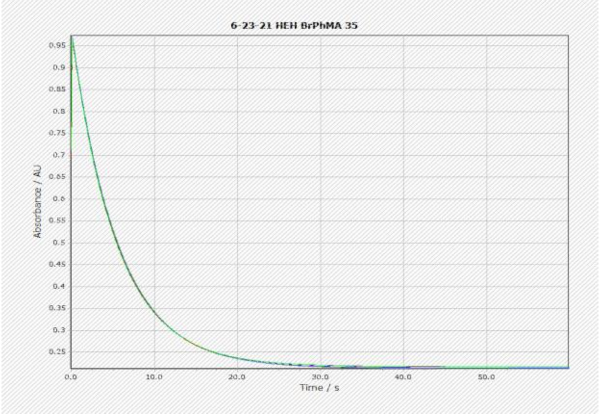
</td><td>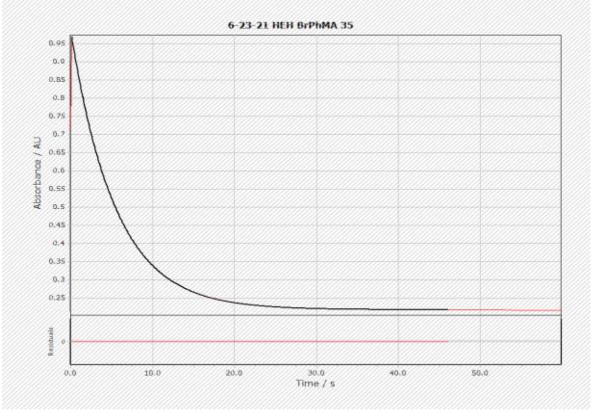
</td></tr>
<tr><td colspan="2">35℃ H</td></tr>
</table>

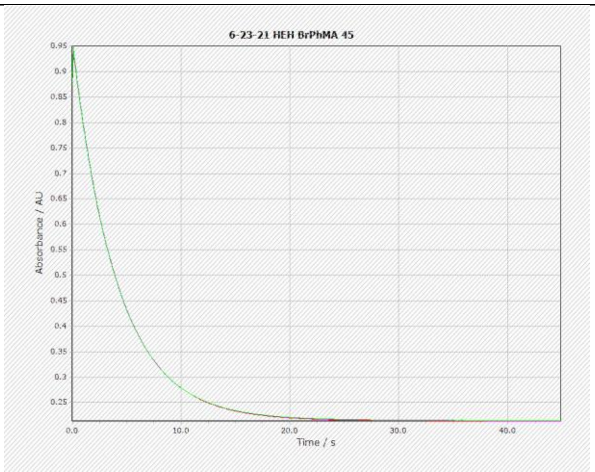

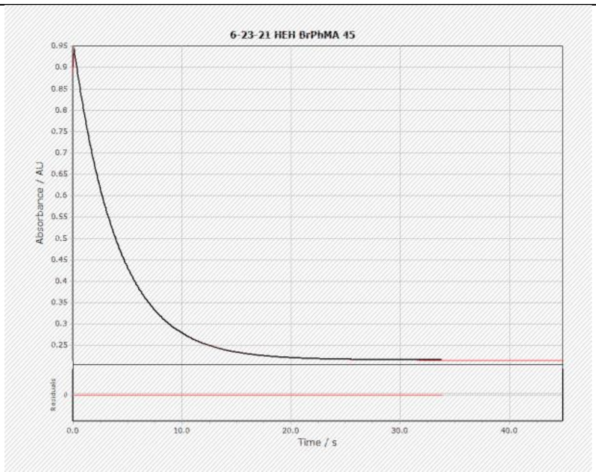

45°C H

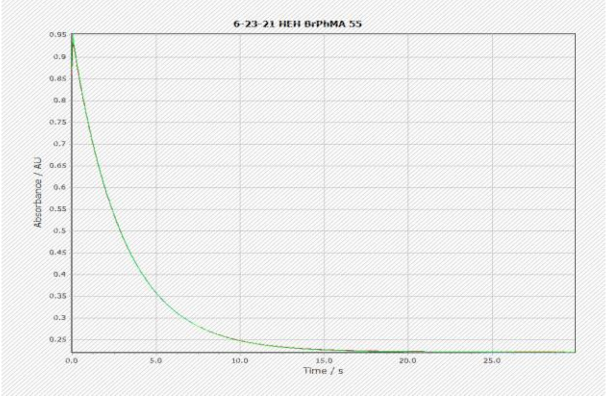

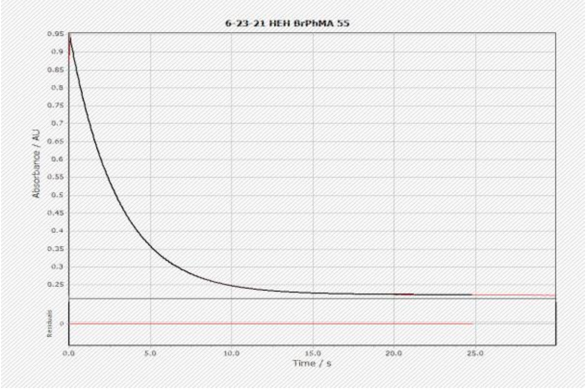

55°C H

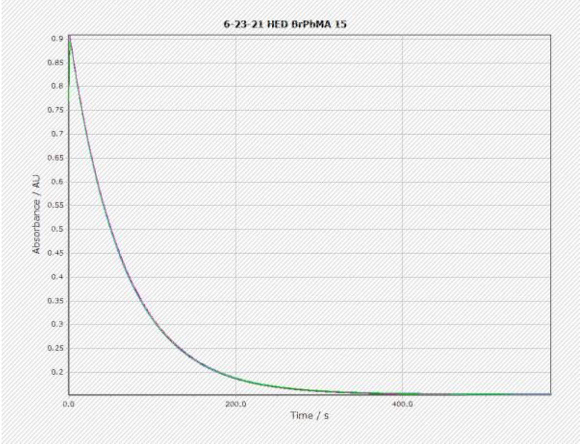

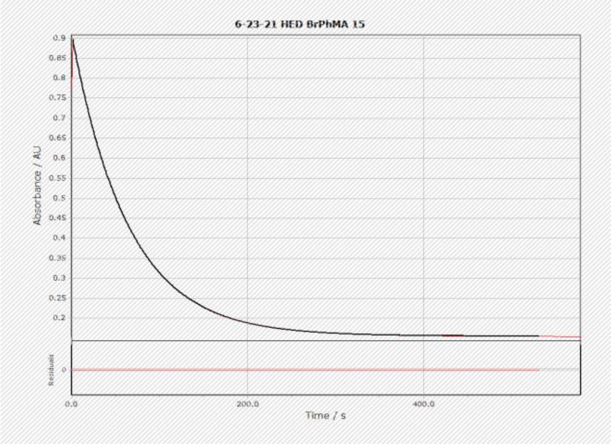

15°C D

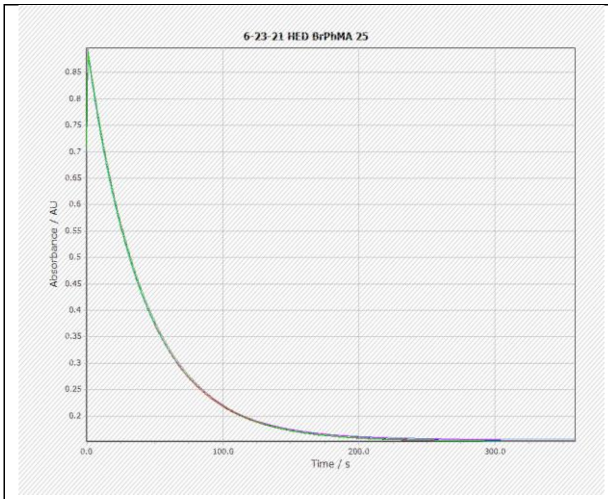
6-23-21 HED BrPhMA 25
Absorbance / AU
Time / s

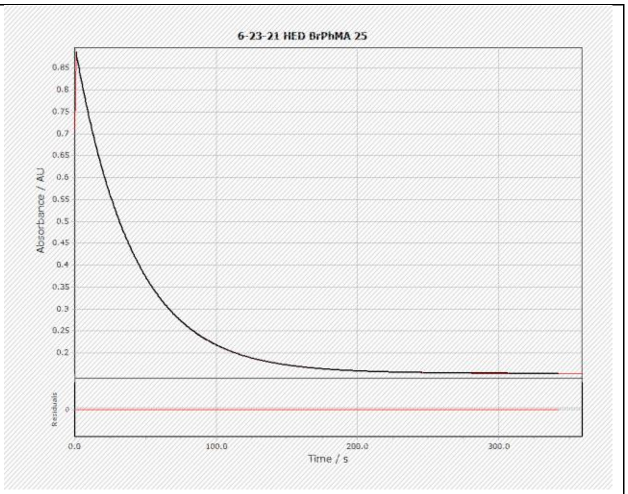
6-23-21 HED BrPhMA 25
Absorbance / AU
Residuals
Time / s

25℃ D

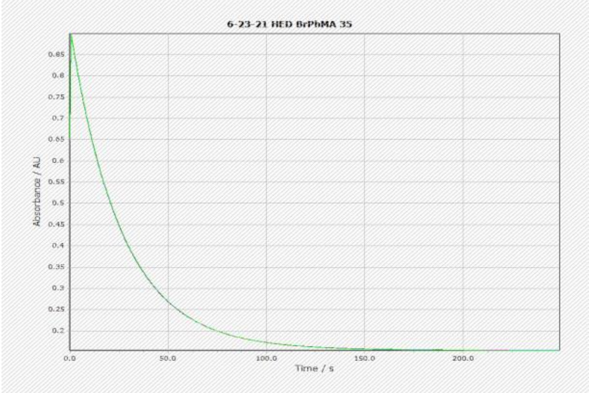
6-23-21 HED BrPhMA 35
Absorbance / AU
Time / s

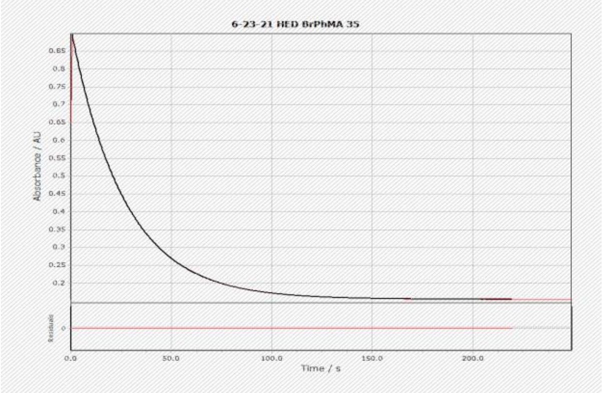
6-23-21 HED BrPhMA 35
Absorbance / AU
Residuals
Time / s

35℃ D

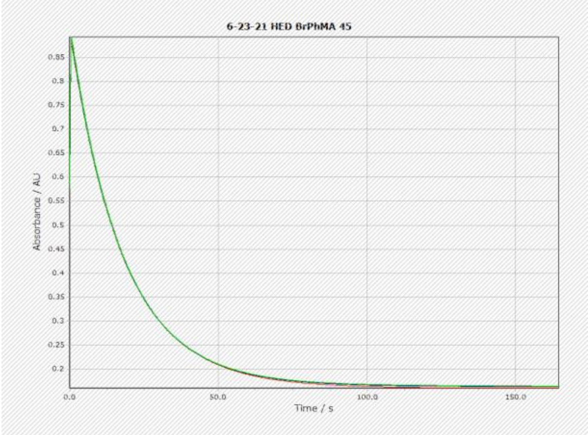
6-23-21 HED BrPhMA 45
Absorbance / AU
Time / s

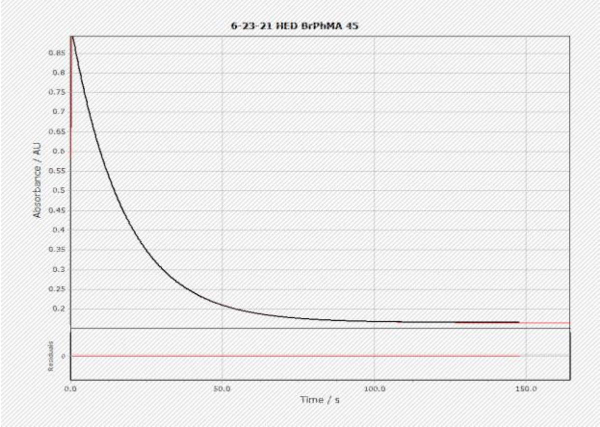
6-23-21 HED BrPhMA 45
Absorbance / AU
Residuals
Time / s

45℃ D

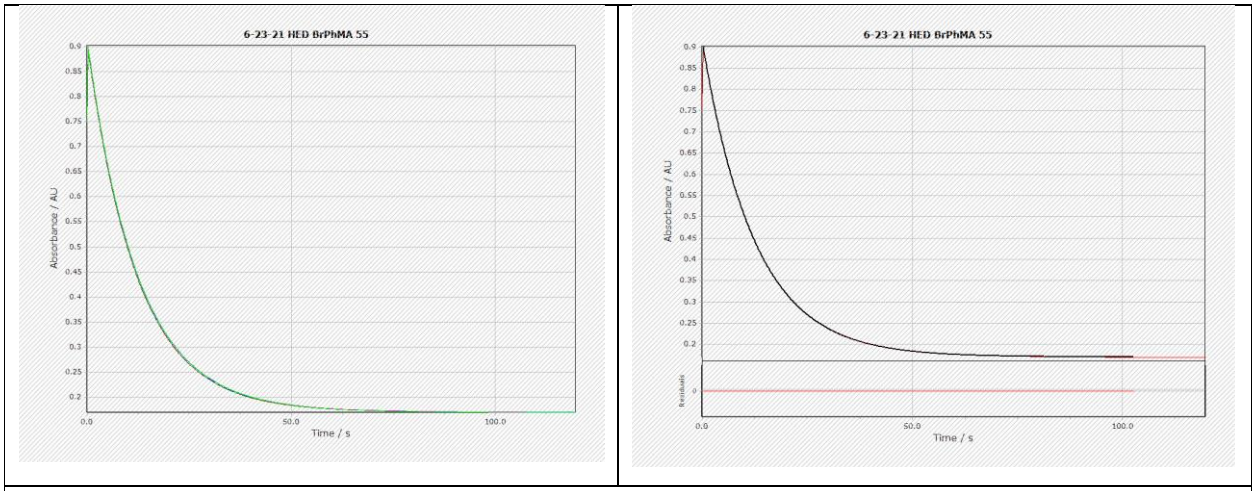

55°C D

Primary kinetic data for the rate constants in Table S11

Day 1 data (June 9, 2021)

	Pseudo-first-order rate constants k^{pfo} (s^{-1})									
**Temp (°C)**	**Trial H1**	**Trial H2**	**Trial H3**	**Trial H4**	**Trial H5**	**Trial H6**	**Average k_H^{pfo} (s^{-1})**	**Stdev**	**k_{2H} ($M^{-1}s^{-1}$)**	**Stdev [a]**
55	0.4276	0.4296	0.4306	0.4368	0.4439	0.4362	0.4341	0.006023	7.24E+01	1.00385
45	0.3138	0.3178	0.3140	0.3177	0.3170	0.3185	0.3165	0.00204	5.27E+01	0.34004
35	0.2350	0.2388	0.2390	0.2379	0.2393	0.2357	0.2376	0.001847	3.96E+01	0.30786
25	0.1625	0.1626	0.1665	0.1649	0.1663	0.1662	0.1648	0.001836	2.75E+01	0.30593
15	0.0973	0.1094	0.1092	0.1116	0.1112	0.1108	0.1083	0.005463	1.80E+01	0.91048

**Temp (°C)**	**Trial D1**	**Trial D2**	**Trial D3**	**Trial D4**	**Trial D5**	**Trial D6**	**Average k_D^{pfo} (s^{-1})**	**Stdev**	**k_{2D} ($M^{-1}s^{-1}$)**	**Stdev [a]**
55	0.1016	0.1027	0.1011	0.1011	0.1017	0.1019	0.1017	0.000597	1.66E+01	0.09755
45	0.0713	0.0713	0.0708	0.0708	0.0712	0.0710	0.0711	0.000241	1.16E+01	0.03942
35	0.0464	0.0489	0.0495	0.0495	0.0493	0.0484	0.0487	0.00116	7.95E+00	0.18943
25	0.0314	0.0317	0.0317	0.0317	0.0315	0.0316	0.0316	0.000124	5.16E+00	0.02024
15	0.0197	0.0204	0.0207	0.0207	0.0204	0.0206	0.0204	0.000393	3.34E+00	0.06420

[a] = (Stdev(for k^{pfo})/k^{pfo})*k_{2H}

<table>
<tr><td>Six kinetic runs together with the averaged data</td><td>First-order kinetic fit of the averaged data as an example</td></tr>
<tr><td>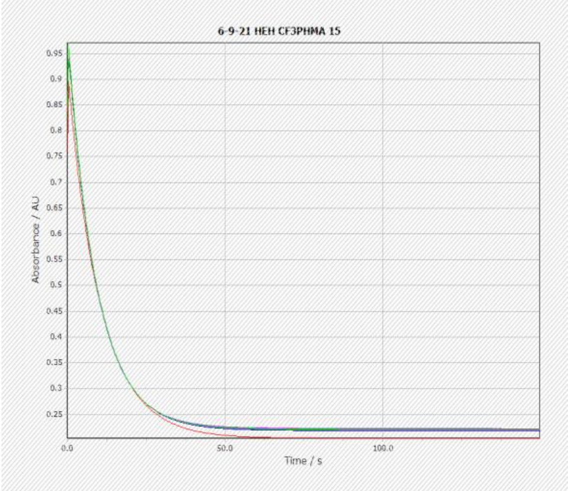
</td><td>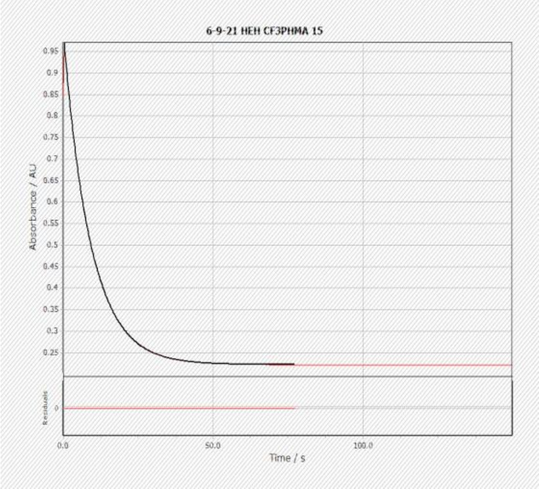
</td></tr>
<tr><td colspan="2">15℃ H</td></tr>
<tr><td>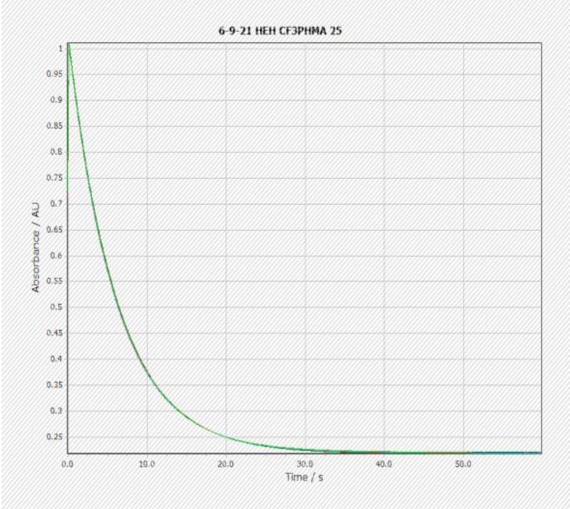
</td><td>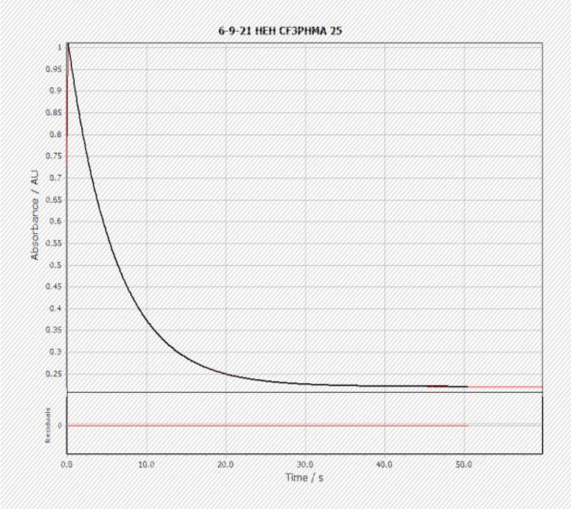
</td></tr>
<tr><td colspan="2">25℃ H</td></tr>
<tr><td>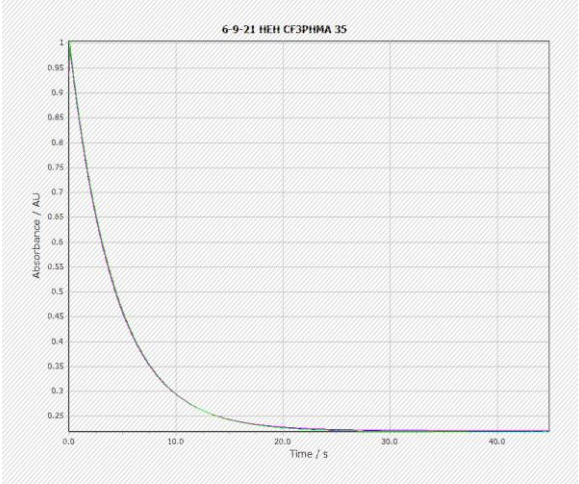
</td><td>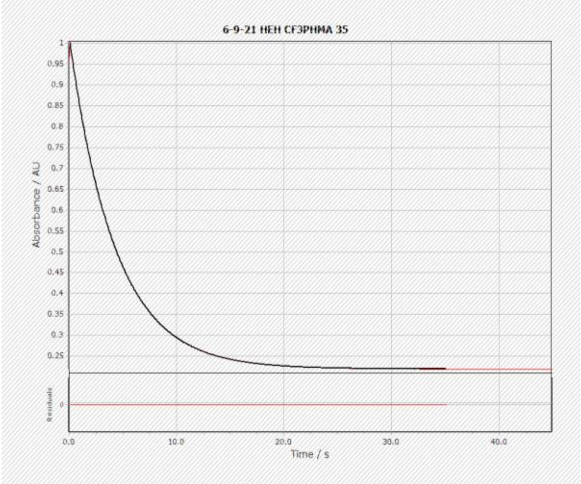
</td></tr>
<tr><td colspan="2">35℃ H</td></tr>
</table>

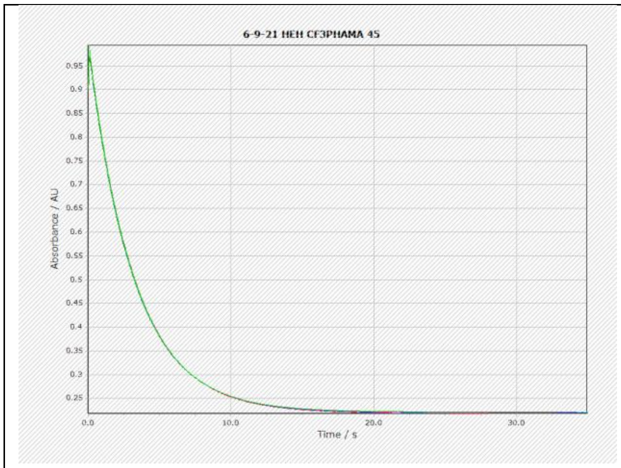

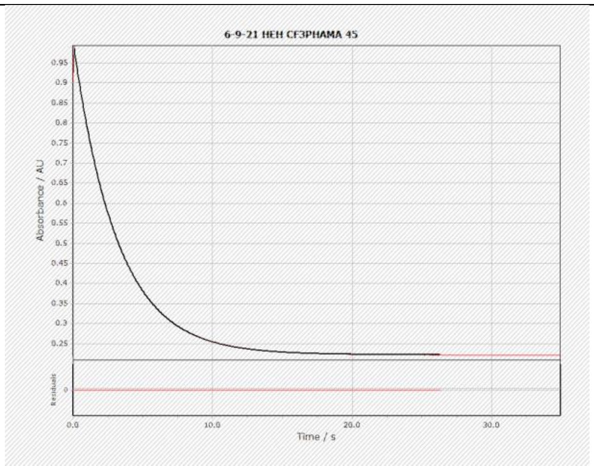

45°C H

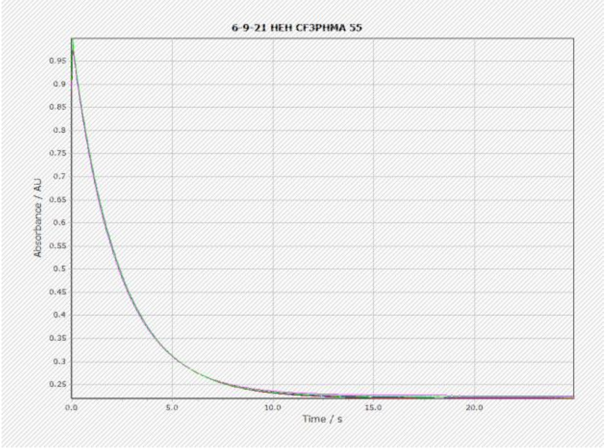

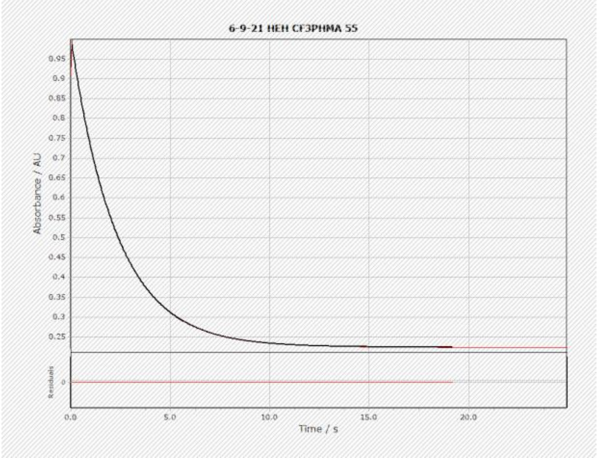

55°C H

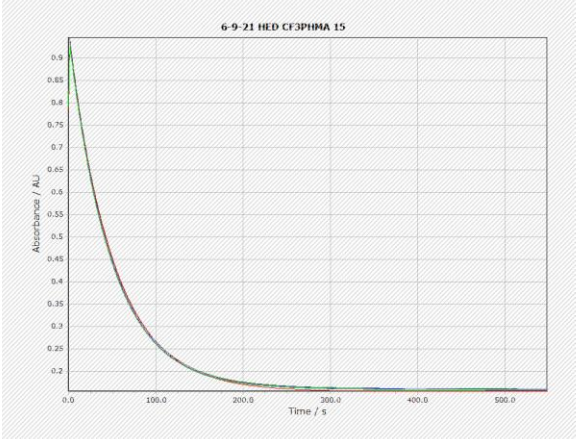

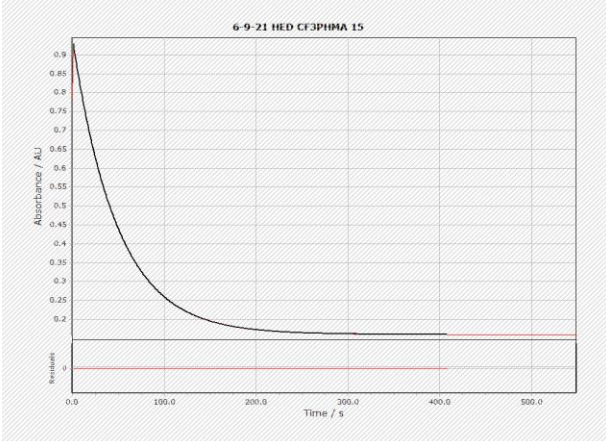

15°C D

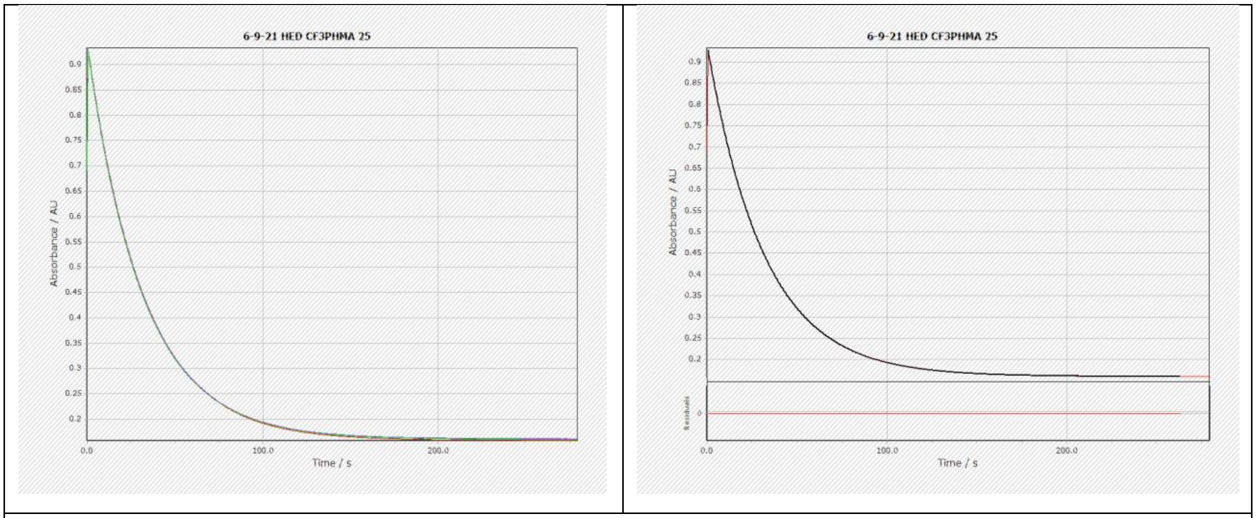

25°C D

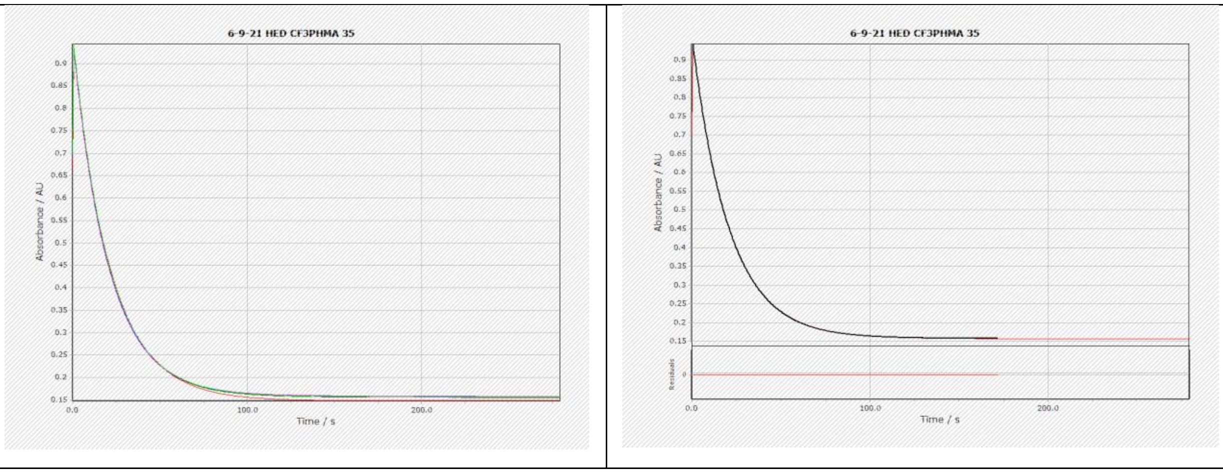

35°C D

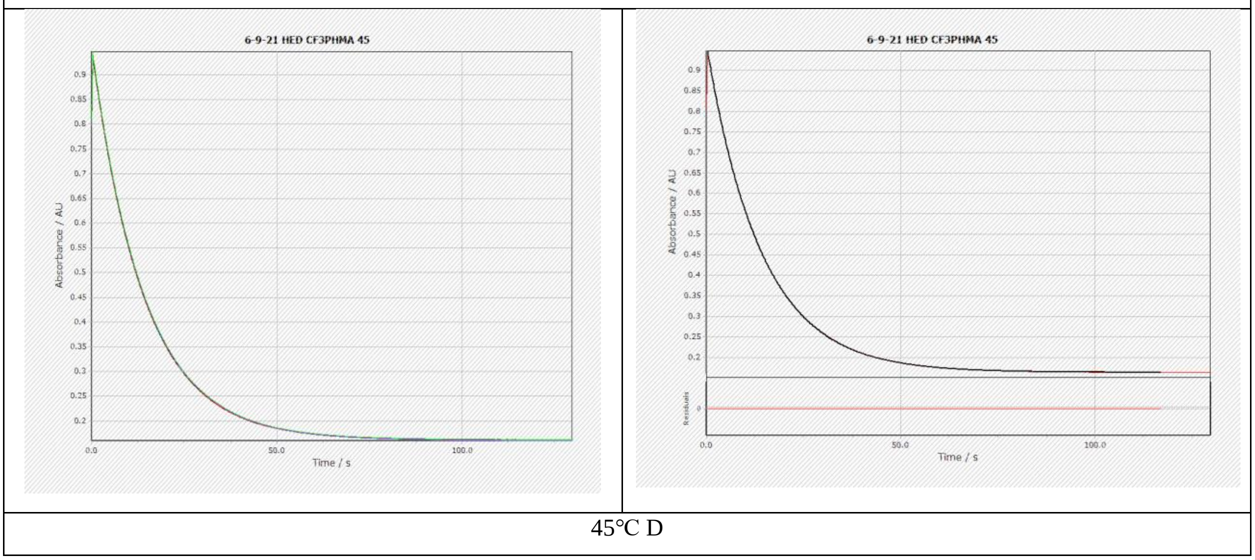

45°C D

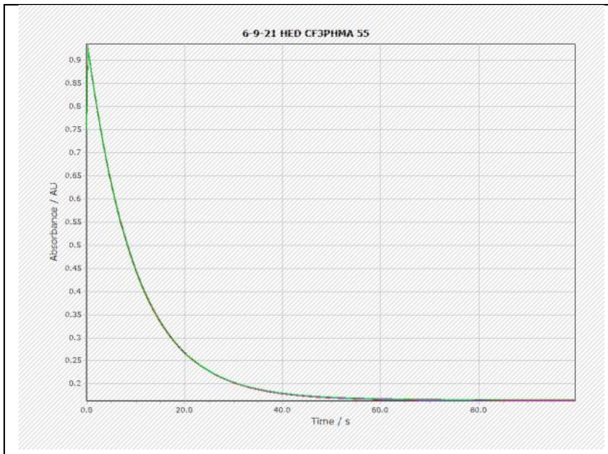

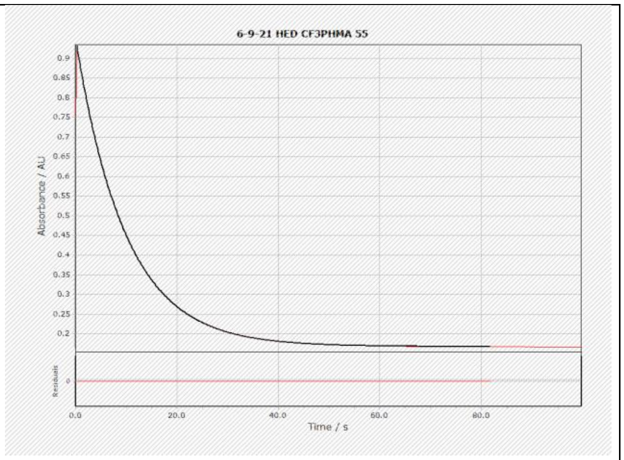

55°C D

Day 2 data (June 11, 2021)

	Pseudo-first-order rate constants k^{pfo} (s^{-1})									
**Temp (°C)**	**Trial H1**	**Trial H2**	**Trial H3**	**Trial H4**	**Trial H5**	**Trial H6**	**Average k_H^{pfo} (s^{-1})**	**Stdev**	**k_{2H} ($M^{-1}s^{-1}$)**	**Stdev [a]**
55	0.4335	0.4431	0.4369	0.4422	0.4358	0.4421	0.4389	0.0041	73.1542	0.6756
45	0.3142	0.3184	0.3190	0.3188	0.3214	0.3189	0.3185	0.0024	53.0753	0.3920
35	0.2326	0.2313	0.2321	0.23799	0.23554	0.23308	0.2338	0.0025	38.9608	0.4194
25	0.1613	0.1632	0.1629	0.1644	0.1647	0.1641	0.1634	0.0013	27.2369	0.2097
15	0.1090	0.1107	0.1114	0.1103	0.1116	0.1124	0.1109	0.0012	18.4806	0.1992

**Temp (°C)**	**Trial D1**	**Trial D2**	**Trial D3**	**Trial D4**	**Trial D5**	**Trial D6**	**Average k_D^{pfo} (s^{-1})**	**Stdev**	**k_{2D} ($M^{-1}s^{-1}$)**	**Stdev [a]**
55	0.1142	0.1132	0.1133	0.1125	0.1142	0.1134	0.1134	0.000639	1.88E+01	0.10583
45	0.0723	0.0720	0.0716	0.0720	0.0719	0.0723	0.0720	0.000255	1.192E+01	0.04221
35	0.0483	0.0495	0.0500	0.0493	0.0499	0.0492	0.0493	0.00061	8.17E+00	0.10090
25	0.0322	0.0319	0.0320	0.0320	0.0320	0.0320	0.0320	0.000107	5.30E+00	0.01775
15	0.0205	0.0209	0.0208	0.0210	0.0207	0.0208	0.0208	0.00018	3.44E+00	0.02979

[a] = (Stdev(for k^{pfo})/k^{pfo})*k_{2H}

<table>
<tr><td>Six kinetic runs together with the averaged data</td><td>First-order kinetic fit of the averaged data as an example</td></tr>
<tr><td>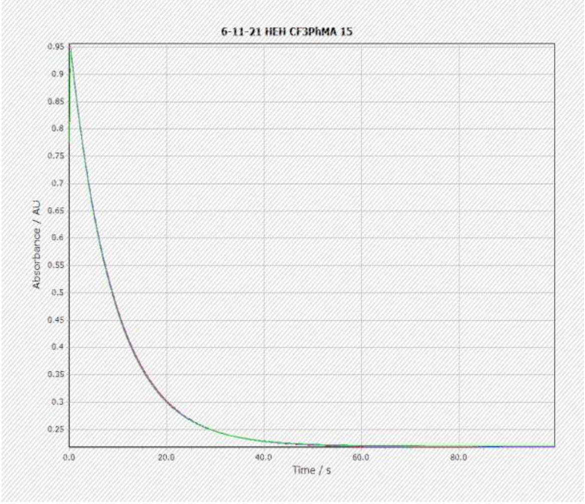
</td><td>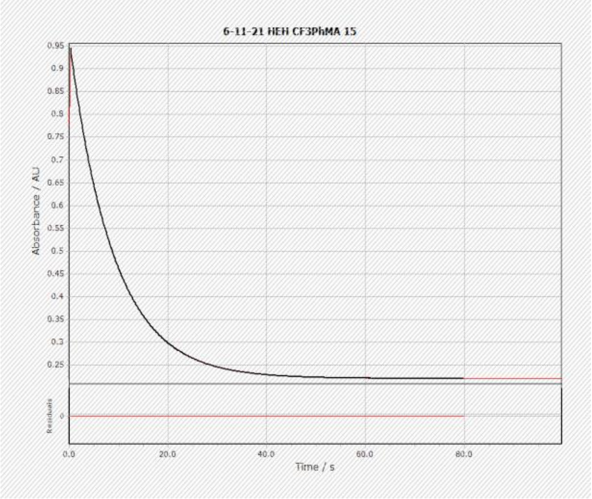
</td></tr>
<tr><td colspan="2">15°C H</td></tr>
<tr><td>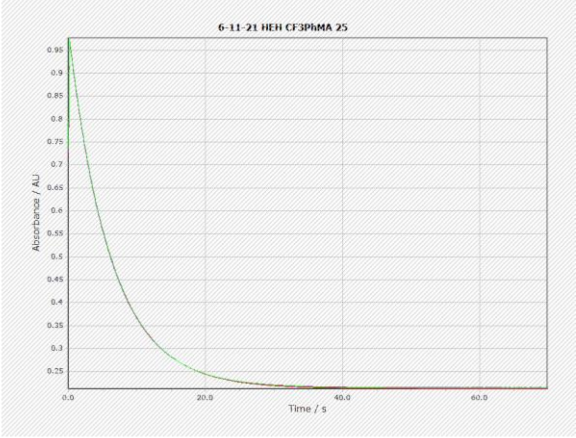
</td><td>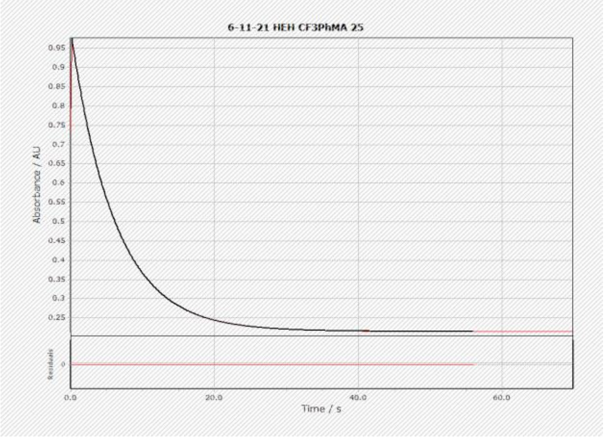
</td></tr>
<tr><td colspan="2">25°C H</td></tr>
<tr><td>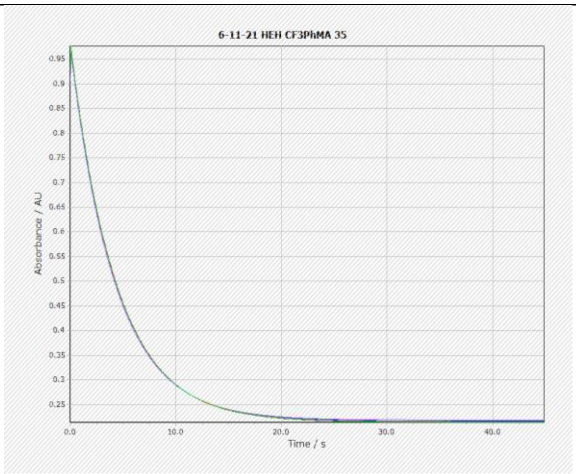
</td><td>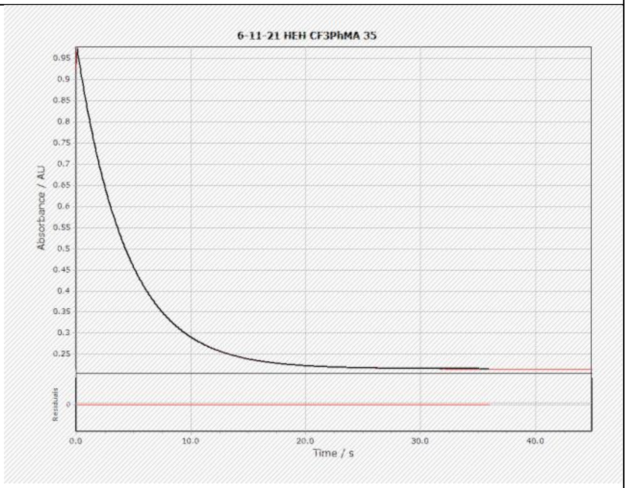
</td></tr>
<tr><td colspan="2">35°C H</td></tr>
</table>

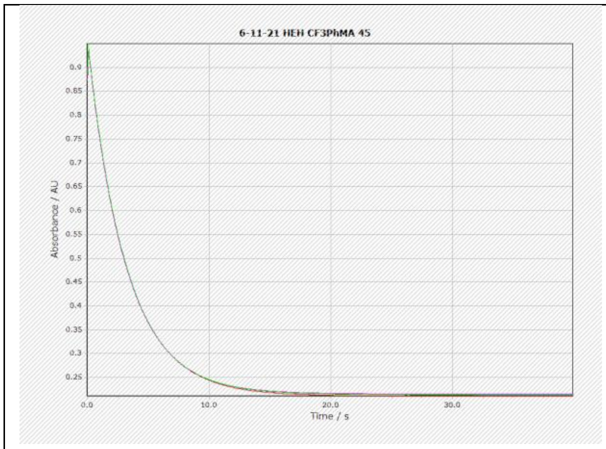
6-11-21 HEH CF3PhMA 45
Absorbance / AU
Time / s

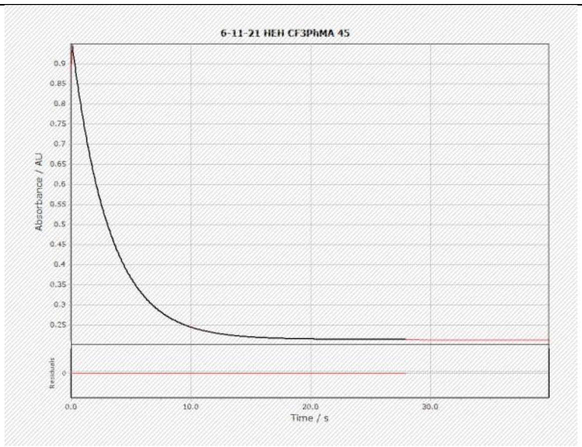
6-11-21 HEH CF3PhMA 45
Absorbance / AU
Residuals
Time / s

45℃ H

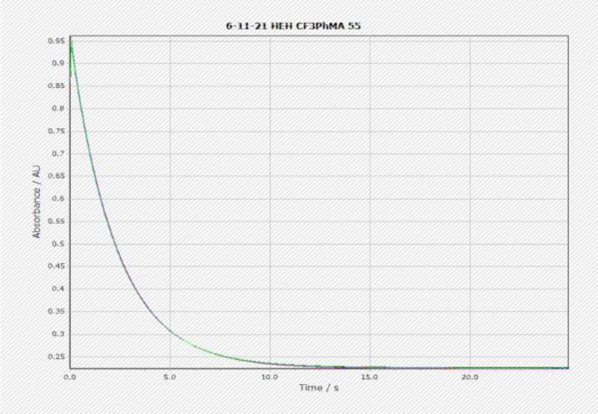
6-11-21 HEH CF3PhMA 55
Absorbance / AU
Time / s

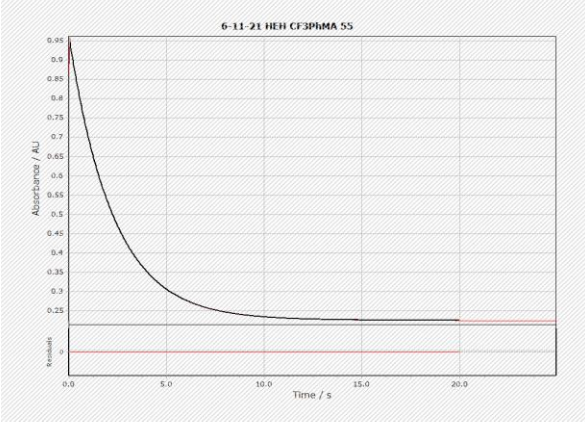
6-11-21 HEH CF3PhMA 55
Absorbance / AU
Residuals
Time / s

55℃ H

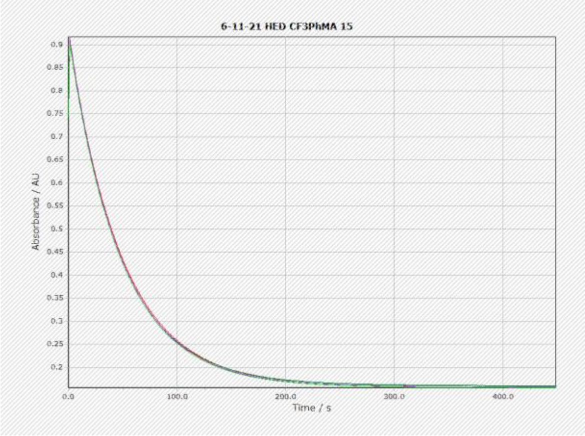
6-11-21 HED CF3PhMA 15
Absorbance / AU
Time / s

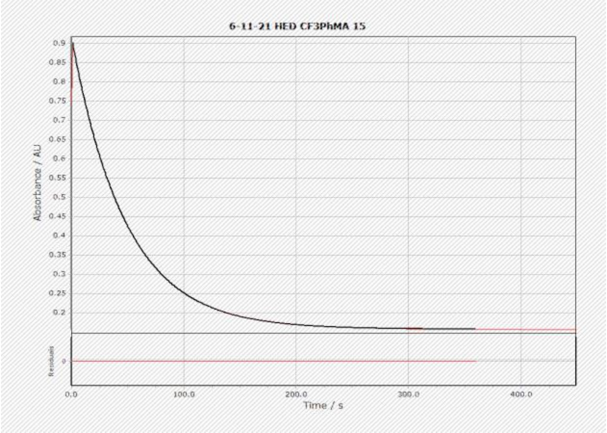
6-11-21 HED CF3PhMA 15
Absorbance / AU
Residuals
Time / s

15℃ D

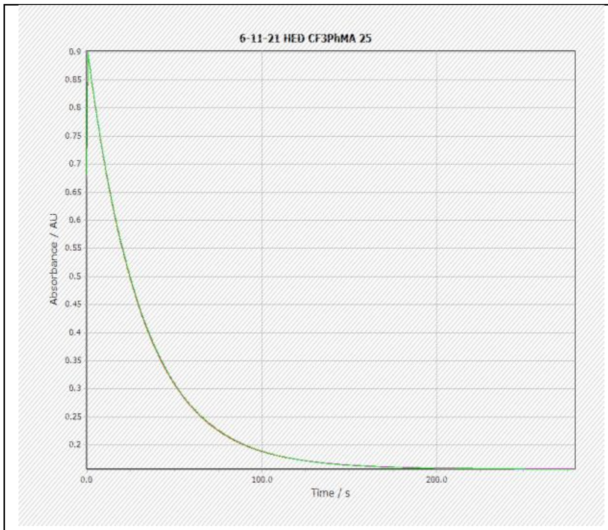
6-11-21 HED CF3PhMA 25
Absorbance / AU
Time / s
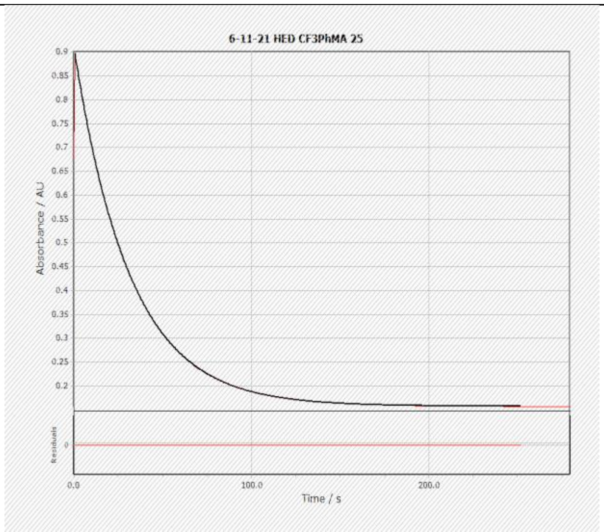
6-11-21 HED CF3PhMA 25
Absorbance / AU
Residuals
Time / s

25℃ D

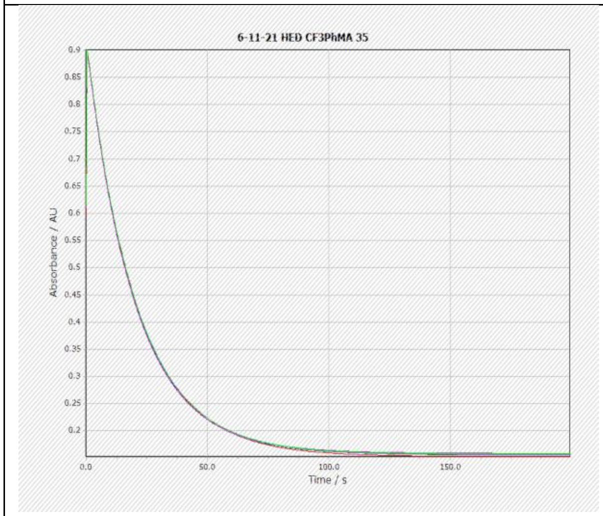
6-11-21 HED CF3PhMA 35
Absorbance / AU
Time / s
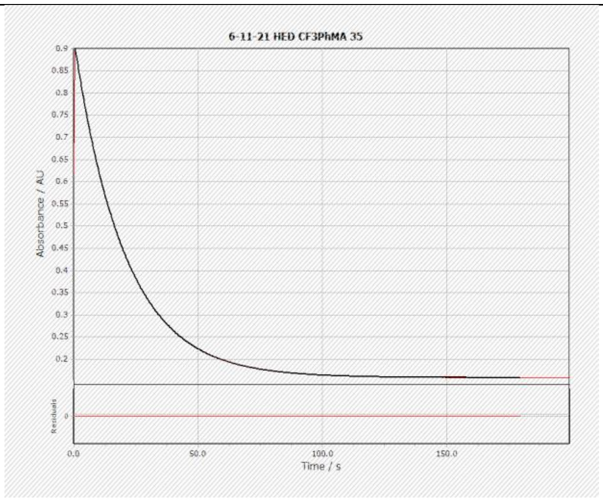
6-11-21 HED CF3PhMA 35
Absorbance / AU
Residuals
Time / s

35℃ D

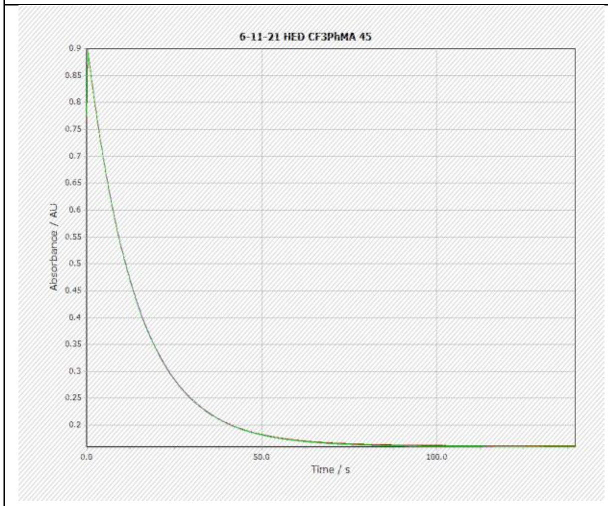
6-11-21 HED CF3PhMA 45
Absorbance / AU
Time / s
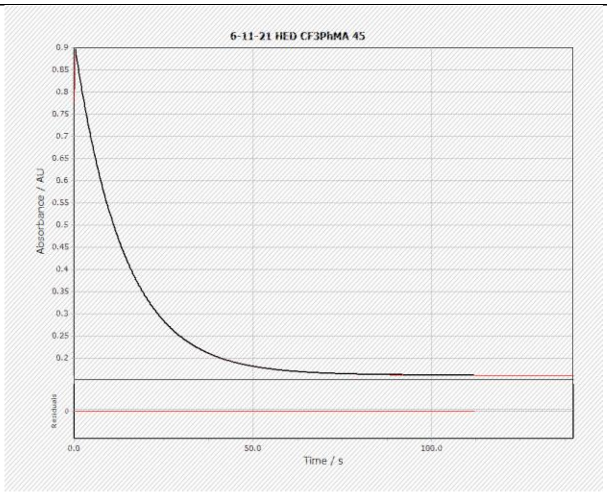
6-11-21 HED CF3PhMA 45
Absorbance / AU
Residuals
Time / s

45℃ D

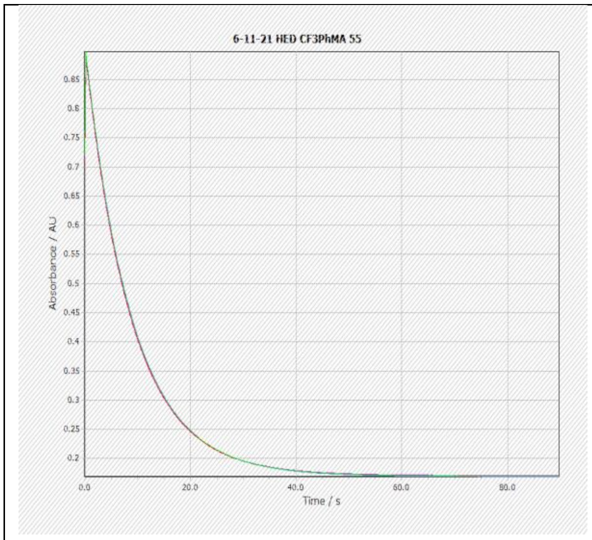	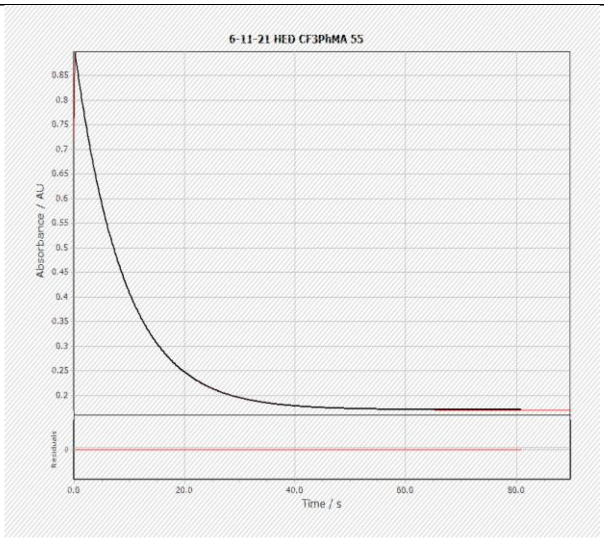
55°C D	

Primary kinetic data for the rate constants in Table S13

For the reaction of $PhXn^+$ (A_{343nm}(DMPBIH(γ,γ-2CH_3)) = 1.0936, A_{343nm}(DMPBIH(γ,γ-2CD_3)) = 1.13395)

Date	Runs	k^{pfo} DMPBIH(γ,γ-2CH_3) (s^{-1})	k^{pfo} DMPBIH(γ,γ-2CD_3) (s^{-1})	γ-2° KIE [a] ($k_2(CH_3)/k_2(CD_3)$)
Sept 19, 2023	1	56.520	66.293	
	2	57.178	66.233	
	3	57.634	65.886	
	4	56.753	66.471	
	5	57.335	65.465	
	6	57.985	66.847	
	Average	57.234	66.199	0.896
	SD	0.496	0.436	0.010

[a] Calculated from eqn (3) in the main paper.

Six kinetic runs together with the averaged data	First-order kinetic fit of the averaged data as an example
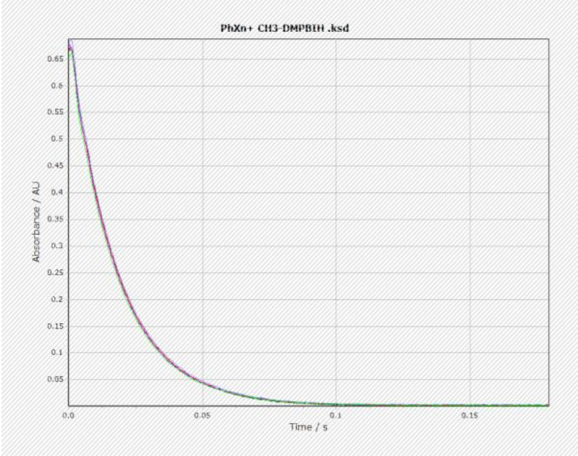	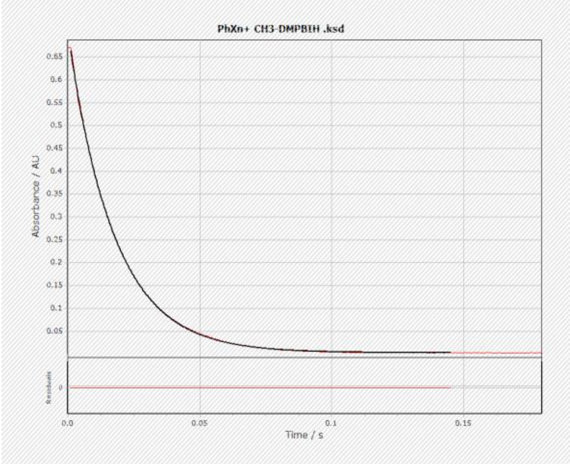
DMPBIH(γ,γ-2CH_3)	

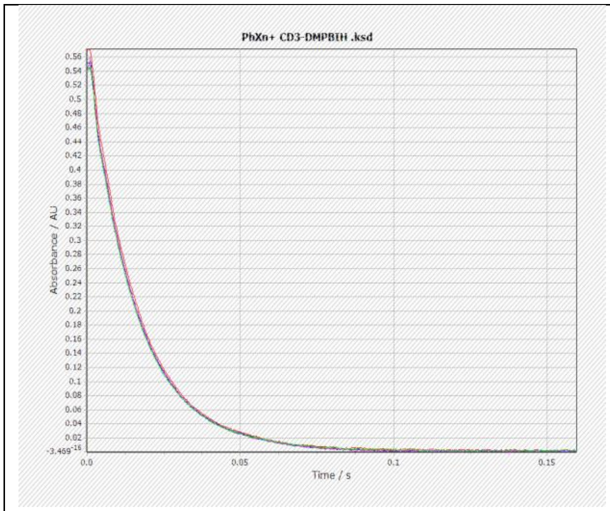

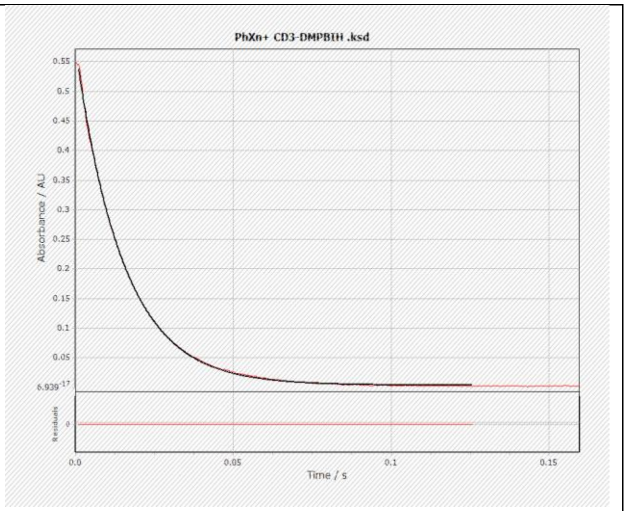

DMPBIH(γ,γ-2CD$_3$)

For the reaction of PhXn$^+$ (A_{343nm}(DMPBIH(γ,γ-2CH$_3$)) = 1.11598, A_{343nm}(DMPBIH(γ,γ-2CD$_3$)) = 1.04573)

Date	Runs	k^{pfo} DMPBIH(γ,γ-2CH$_3$) (s^{-1})	k^{pfo} DMPBIH(γ,γ-2CD$_3$) (s^{-1})	γ-2° KIE [a] (k_2(CH$_3$)/k_2(CD$_3$))
Sept 18, 2023(1)	1	57.420	60.463	
	2	56.798	61.150	
	3	56.498	59.911	
	4	57.024	60.489	
	5	57.008	60.747	
	6	57.008	60.982	
	Average	56.959	60.624	0.880
	SD	0.277	0.403	0.007

[a] Calculated from eqn (3) in the main paper.

Six kinetic runs together with the averaged data

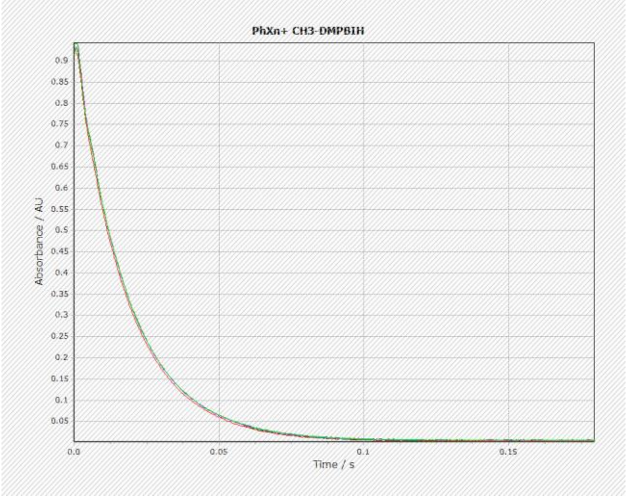

First-order kinetic fit of the averaged data as an example

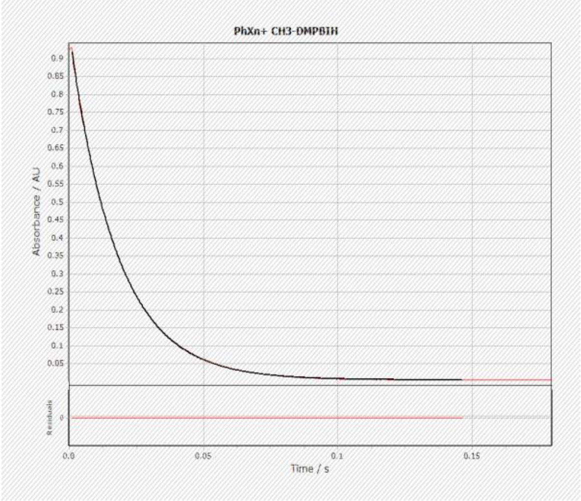

DMPBIH(γ,γ-2CH$_3$)

<table>
<tr><td>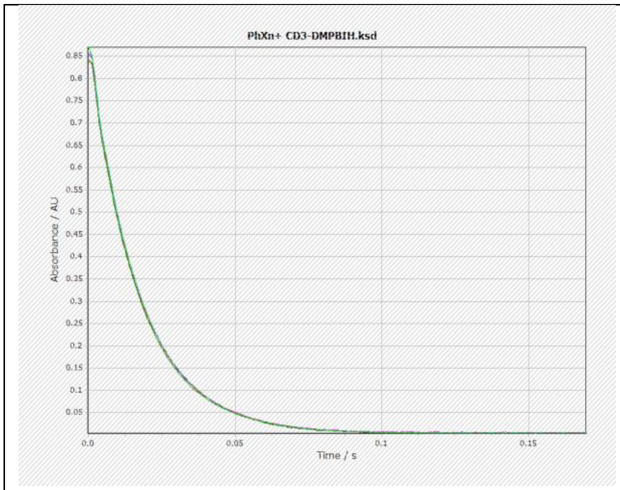
</td><td>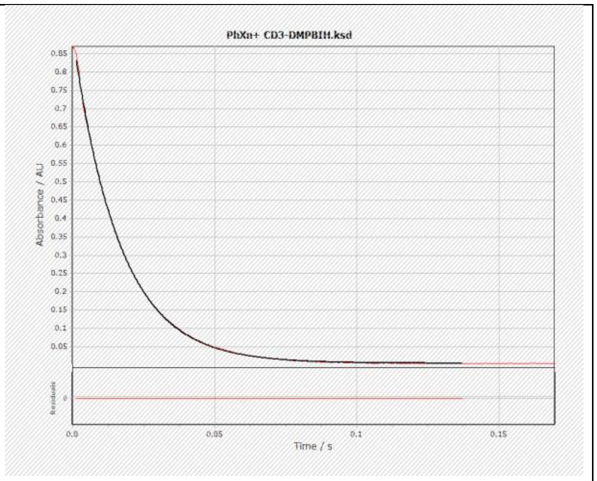
</td></tr>
<tr><td colspan="2">DMPBIH(γ,γ-2CD₃)</td></tr>
</table>

For the reaction of $PhXn^+$ (A_{343nm}(DMPBIH(γ,γ-2CH_3)) = 1.11598, A_{343nm}(DMPBIH(γ,γ-2CD_3)) = 1.04573)

Date	Runs	k^{pfo} DMPBIH(γ,γ-2CH_3) (s^{-1})	k^{pfo} DMPBIH(γ,γ-2CD_3) (s^{-1})	γ-2° KIE [a] ($k_2(CH_3)/k_2(CD_3)$)
Sept 18, 2023(2)	1	57.414	62.942	
	2	57.517	63.054	
	3	57.206	63.552	
	4	57.429	63.541	
	5	57.364	63.601	
	6	57.830	64.323	
	Average	57.460	63.502	0.848
	SD	0.190	0.447	0.007

<table>
<tr><td>Six kinetic runs together with the averaged data</td><td>First-order kinetic fit of the averaged data as an example</td></tr>
<tr><td>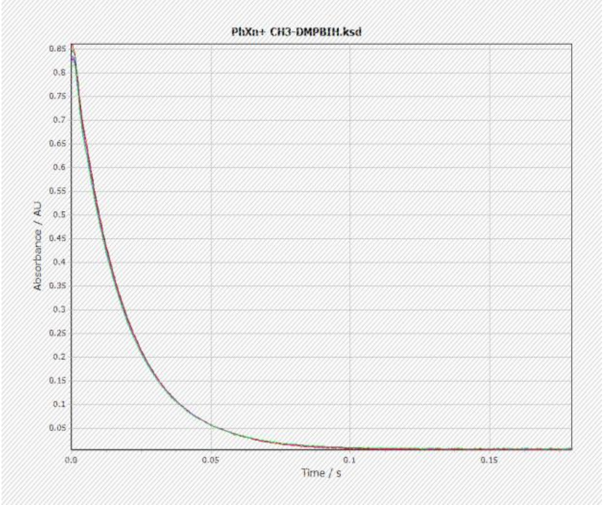
</td><td>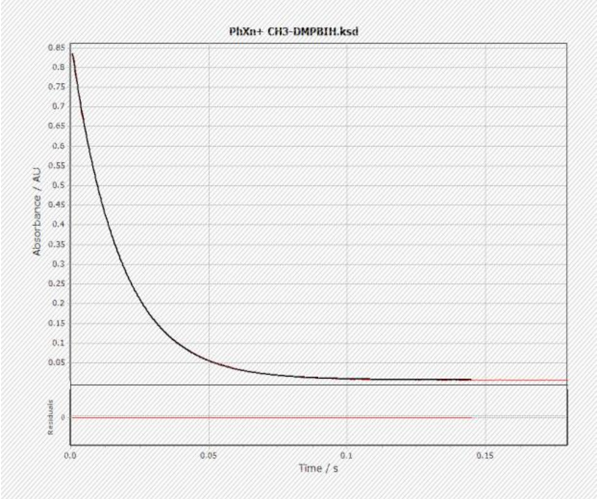
</td></tr>
<tr><td colspan="2">DMPBIH(γ,γ-2CH₃)</td></tr>
</table>

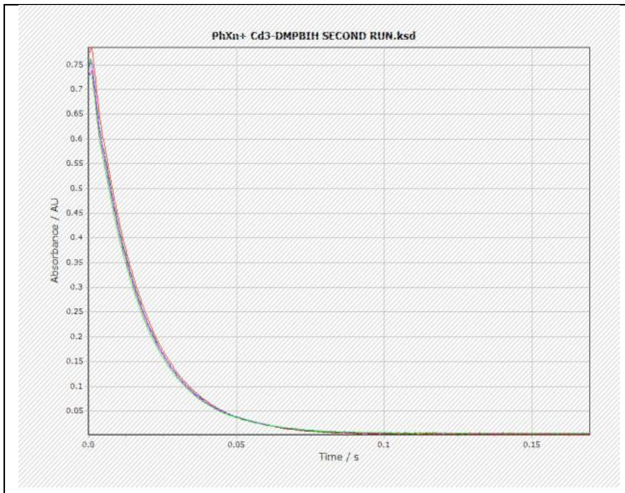	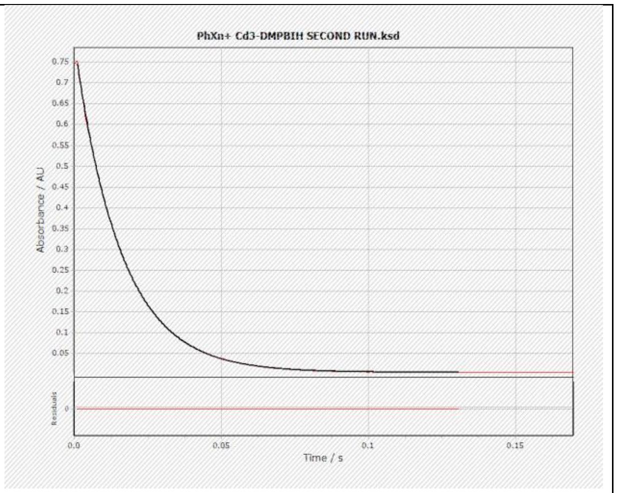
DMPBIH(γ,γ-2CD_3)	

For the reaction of $PhXn^+$ (A_{343nm}(DMPBIH(γ,γ-2CH_3)) = 1.08329, A_{343nm}(DMPBIH(γ,γ-2CD_3)) = 0.84115)

Date	Runs	k^{pfo} DMPBIH(γ,γ-2CH_3) (s^{-1})	k^{pfo} DMPBIH(γ,γ-2CD_3) (s^{-1})	γ-2° KIE [a] ($k_2(CH_3)/k_2(CD_3)$)
Sept 15, 2023	1	55.287	48.372	
	2	55.886	48.874	
	3	55.670	48.586	
	4	56.222	49.396	
	5	55.608	48.446	
	6	55.435	48.229	
	Average	55.685	48.650	0.889
	SD	0.304	0.389	0.009

[a] Calculated from eqn (3) in the main paper.

Six kinetic runs together with the averaged data	First-order kinetic fit of the averaged data as an example
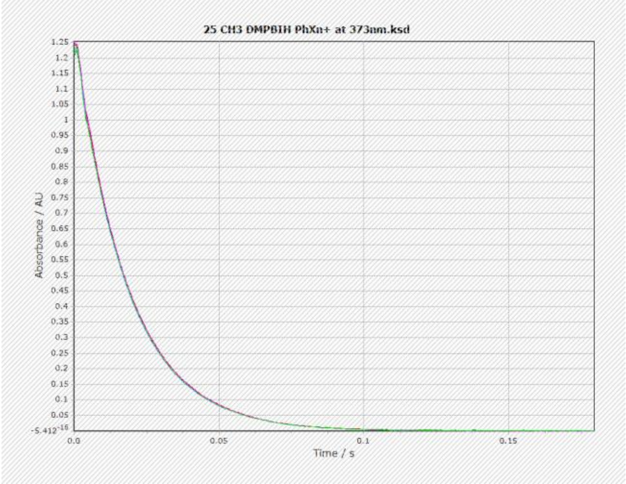	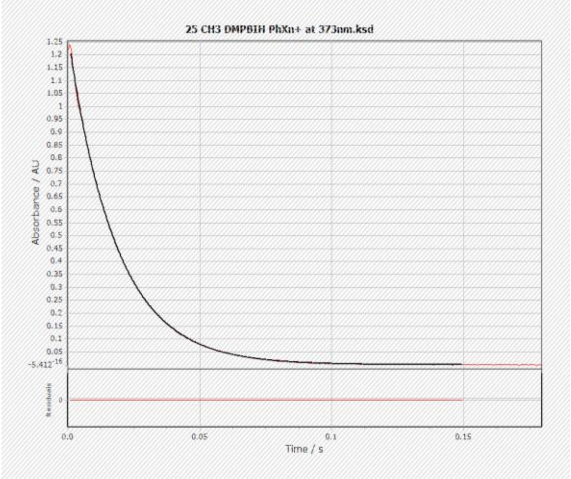
DMPBIH(γ,γ-2CH_3)	

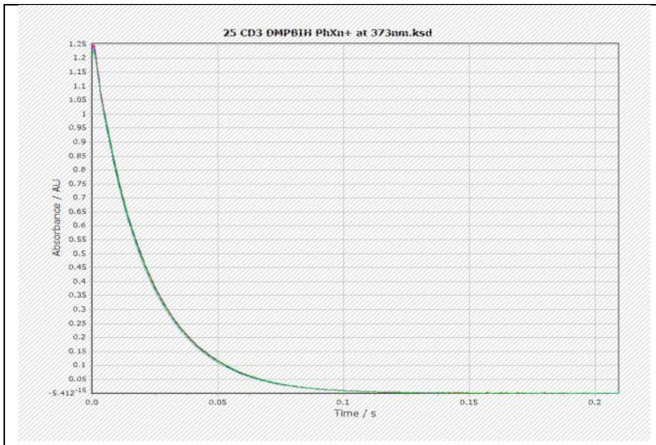

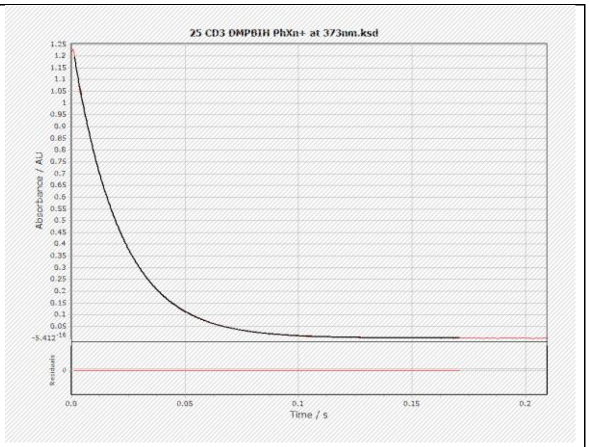

DMPBIH(γ,γ-2CD_3)

For the reaction of $PhTXn^+$ (A_{343nm}(DMPBIH(γ,γ-2CH_3)) = 1.0936, A_{343nm}(DMPBIH(γ,γ-2CD_3)) = 1.13395)

Date	Runs	k^{pfo} DMPBIH(γ,γ-2CH_3) (s^{-1})	k^{pfo} DMPBIH(γ,γ-2CD_3) (s^{-1})	γ-2° KIE [a] ($k_2(CH_3)/k_2(CD_3)$)
Sept 19, 2023	1	187.264	217.694	
	2	188.161	223.483	
	3	186.773	216.403	
	4	188.780	219.747	
	5	189.216	225.605	
	6	189.996	218.359	
	Average	188.365	220.215	0.887
	SD	2.158	3.273	0.014

[a] Calculated from eqn (3) in the main paper.

Six kinetic runs together with the averaged data

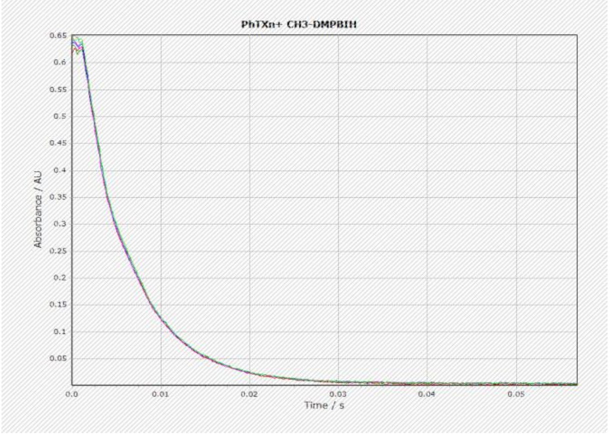

First-order kinetic fit of the averaged data as an example

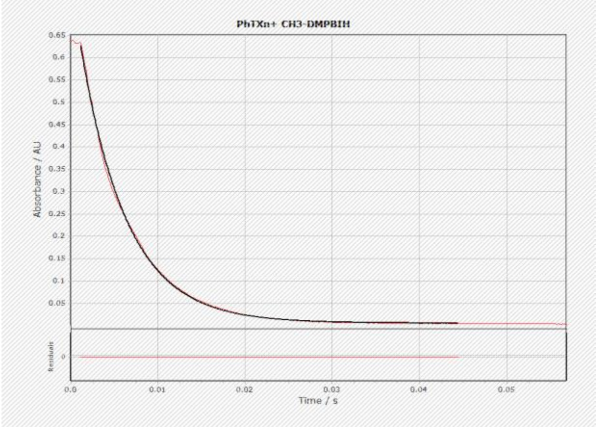

DMPBIH(γ,γ-2CH_3)

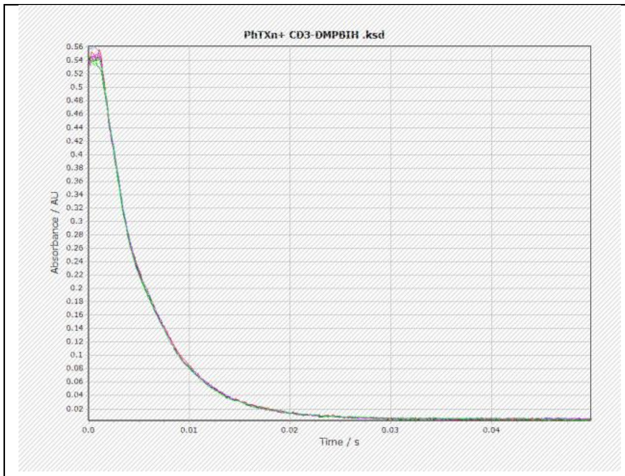

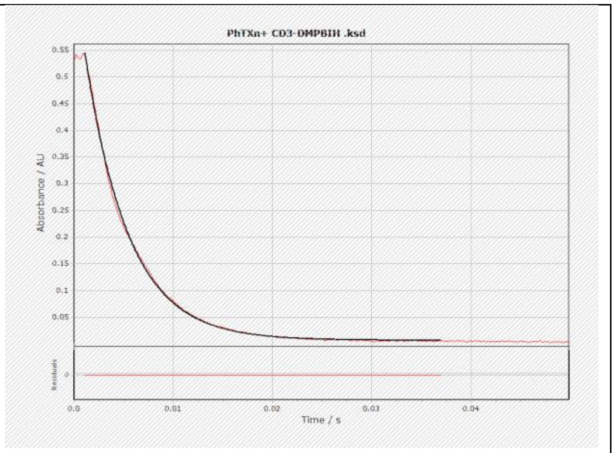

DMPBIH(γ,γ-2CD_3)

For the reaction of $PhTXn^+$ (A_{343nm}(DMPBIH(γ,γ-2CH_3)) = 1.11598, A_{343nm}(DMPBIH(γ,γ-2CD_3)) = 1.04573)

Date	Runs	k^{pfo} DMPBIH(γ,γ-2CH_3) (s^{-1})	k^{pfo} DMPBIH(γ,γ-2CD_3) (s^{-1})	γ-2° KIE [a] ($k_2(CH_3)/k_2(CD_3)$)
Sept 18, 2023(1)	1	186.025	196.517	
	2	189.244	203.932	
	3	184.941	199.804	
	4	186.344	203.728	
	5	190.353	202.344	
	6	190.234	202.440	
	Average	187.857	201.461	0.874
	SD	2.158	2.588	0.015

[a] Calculated using eqn (3) in the main paper.

Six kinetic runs together with the averaged data

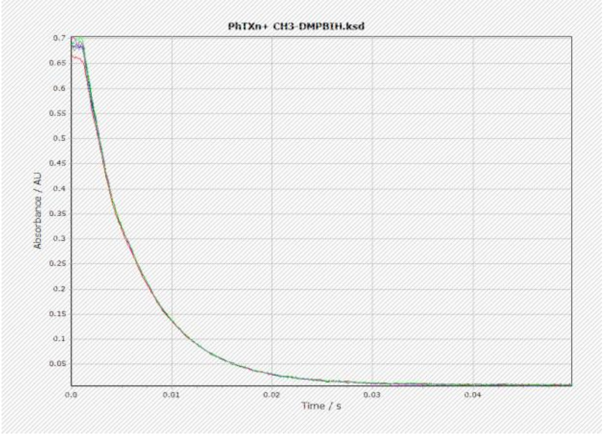

First-order kinetic fit of the averaged data as an example

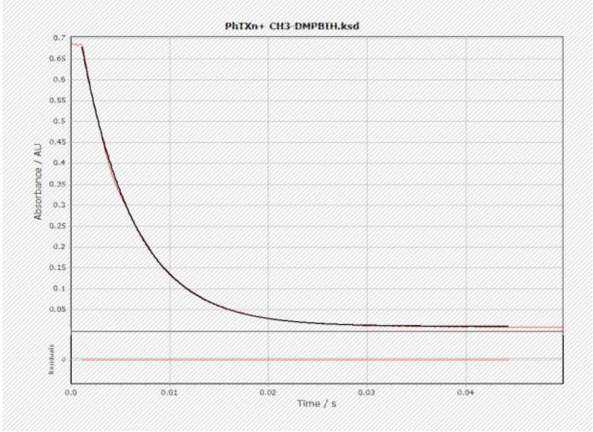

DMPBIH(γ,γ-2CH_3)

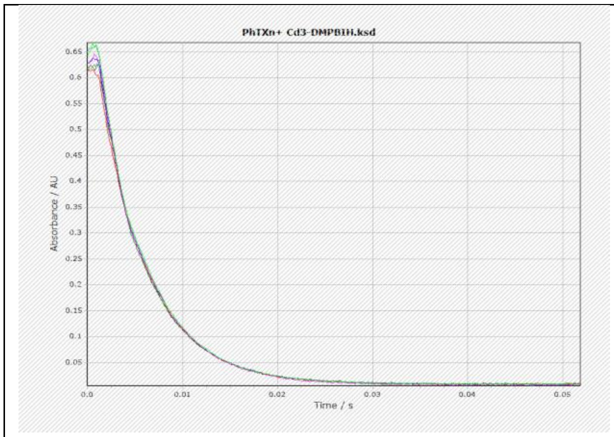

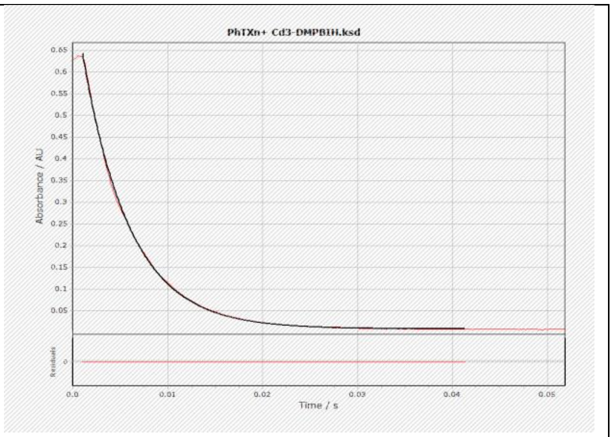

DMPBIH(γ,γ-2CD_3)

For the reaction of $PhTXn^+$ (A_{343nm}(DMPBIH(γ,γ-2CH_3)) = 1.11598, A_{343nm}(DMPBIH(γ,γ-2CD_3)) = 1.04573)

Date	Runs	k^{pfo} DMPBIH(γ,γ-2CH_3) (s^{-1})	k^{pfo} DMPBIH(γ,γ-2CD_3) (s^{-1})	γ-2° KIE [a] ($k_2(CH_3)/k_2(CD_3)$)
Sept 18, 2023(2)	1	194.220	205.205	
	2	188.277	205.787	
	3	197.307	204.754	
	4	191.081	209.576	
	5	196.238	205.533	
	6	195.981	206.931	
	Average	193.851	206.298	0.881
	SD	3.190	1.611	0.016

[a] Calculated using eqn (3) in the main paper.

Six kinetic runs together with the averaged data	First-order kinetic fit of the averaged data as an example
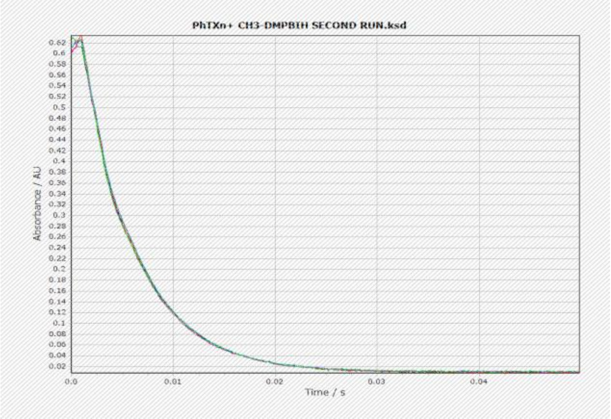	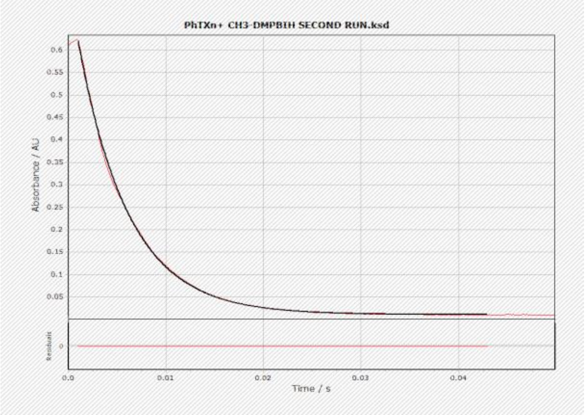

DMPBIH(γ,γ-2CH_3)

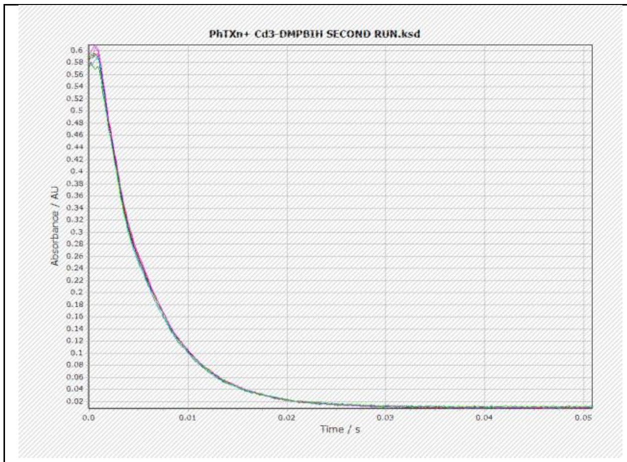

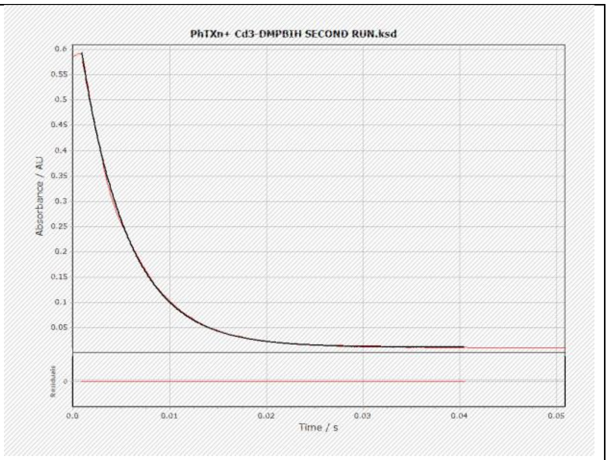

DMPBIH(γ,γ-2CD_3)

For the reaction of $PhTXn^+$ (A_{343nm}(DMPBIH(γ,γ-2CH_3)) = 1.08329, A_{343nm}(DMPBIH(γ,γ-2CD_3)) = 0.84115)

Date	Runs	k^{pfo} DMPBIH(γ,γ-2CH_3) (s^{-1})	k^{pfo} DMPBIH(γ,γ-2CD_3) (s^{-1})	γ-2° KIE [a] ($k_2(CH_3)/k_2(CD_3)$)
Sept 15, 2023	1	193.095	161.550	
	2	188.525	161.897	
	3	190.469	162.704	
	4	189.198	164.851	
	5	191.664	165.667	
	6	190.348	168.878	
	Average	190.550	164.258	0.901
	SD	1.511	2.550	0.016

[a] Calculated from eqn (3) in the main paper.

Six kinetic runs together with the averaged data	First-order kinetic fit of the averaged data as an example
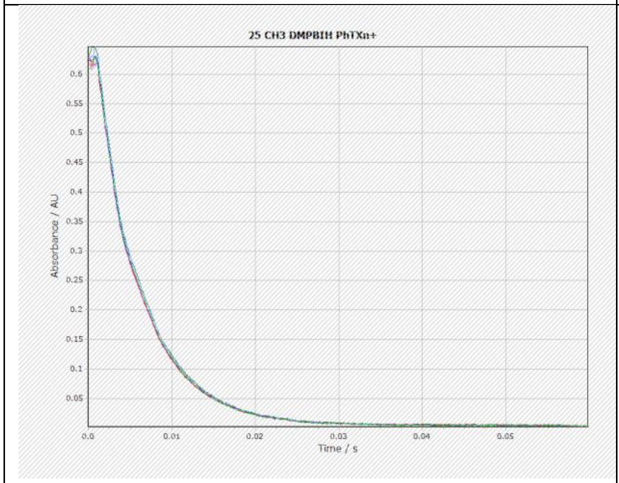	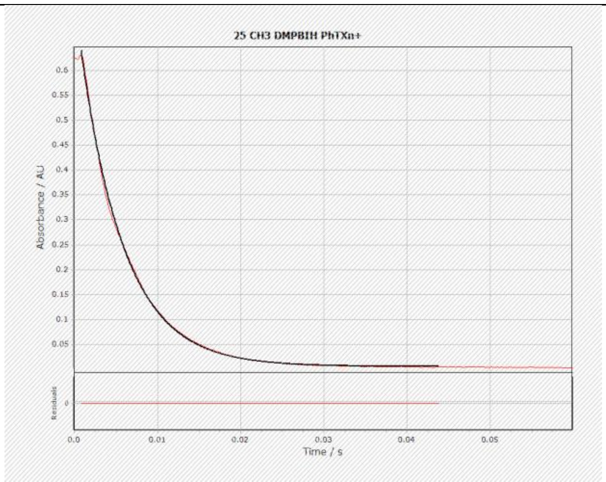

DMPBIH(γ,γ-2CH_3)

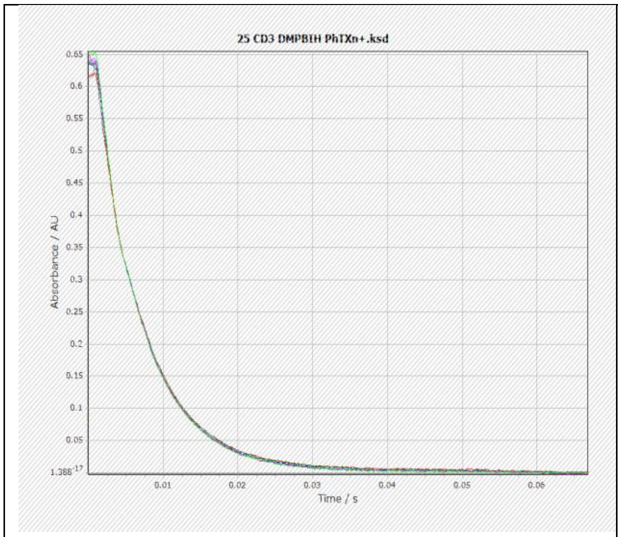	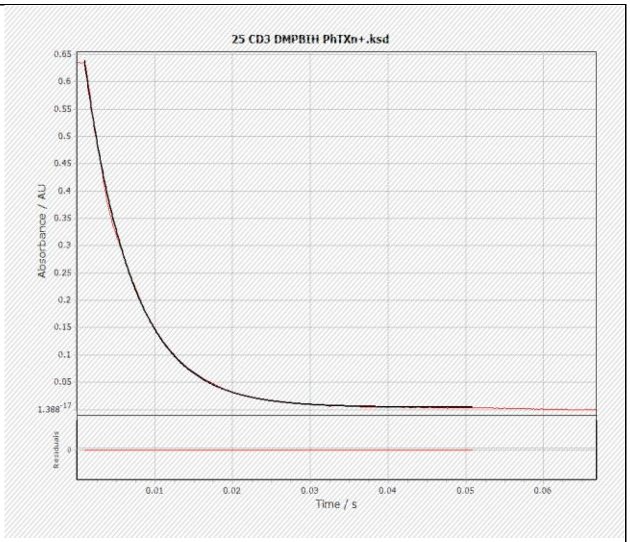
DMPBIH(γ,γ-2CD_3)	
